# The microRNA inhibitor CDR132L in patients with reduced left ventricular ejection fraction after myocardial infarction: a randomized phase 2 trial

**DOI:** 10.1038/s41591-026-04408-4

**Published:** 2026-05-10

**Authors:** Johann Bauersachs, Scott D. Solomon, Stefan D. Anker, Isabel Antorrena-Miranda, Rudolf A. de Boer, Gerasimos Filippatos, Tim Friede, Wilfried Hauke, Piotr Ponikowski, Orly Vardeny, Rebecca Altschul, Michael Gamborg, Kasper Vinther, Thomas Thum

**Affiliations:** 1 Department of Cardiology and Angiology, Hannover Medical School, Hannover, Germany; 2 Cardiovascular Division, Brigham and Women’s Hospital, Harvard Medical School, Boston, MA USA; 3 Department of Cardiology (CVK) of German Heart Center Charité; German Centre for Cardiovascular Research (DZHK), partner site Berlin, Charité Universitätsmedizin, Berlin, Germany; 4 Department of Cardiology, La Paz University Hospital, Research Institute IdiPAZ, Madrid, Spain; 5 Department of Cardiology, Erasmus University Medical Centre, Rotterdam, The Netherlands; 6 Department of Cardiology, Athens University Hospital Attikon, School of Medicine, National and Kapodistrian University of Athens, Athens, Greece; 7 Department of Medical Statistics, University Medical Centre Göttingen, Göttingen, Germany; 8 German Centre for Cardiovascular Research (DZHK), partner site Lower Saxony, Göttingen, Germany; 9 Cardior Pharmaceuticals GmbH, a Novo Nordisk company, Hannover, Germany; 10 Institute of Heart Diseases, University Hospital, Medical University Wroclaw, Wroclaw, Poland; 11 University of Minnesota Medical School, Minneapolis, MN USA; 12 Novo Nordisk A/S, Søborg, Denmark; 13 Institute of Molecular and Translational Therapeutic Strategies (IMTTS), Hannover Medical School, Hannover, Germany

**Keywords:** Heart failure, Heart failure, Antisense oligonucleotide therapy

## Abstract

MicroRNA-132 (miR-132) is a central regulator of adverse cardiac remodeling. Here we evaluated CDR132L, a synthetic antisense oligonucleotide miR-132 inhibitor, in a multinational, randomized, double-blind, placebo-controlled phase 2 trial (HF-REVERT) in patients with recent myocardial infarction (MI) and left ventricular (LV) systolic dysfunction. Within 3–14 days after MI, 294 patients were randomized to receive CDR132L 5 mg kg^−1^, CDR132L 10 mg kg^−1^ or placebo as three intravenous doses at 4-week intervals plus guideline-directed therapy. In total, 280 patients (245 men and 35 women) who received at least one dose of the study drug were included in the modified intention-to-treat population. CDR132L was well tolerated, with no hepatic, renal, hematologic or cardiac toxicity signals. The primary endpoint—the percentage change in LV end-systolic volume index at 6 months—improved in all groups but did not differ significantly between the CDR132L groups (5 mg kg^−1^ and 10 mg kg^−1^) and the placebo group. Secondary endpoints, including LV ejection fraction, global longitudinal strain and N-terminal pro B-type natriuretic peptide, were also not significantly different between the CDR132L and placebo groups. Prespecified exploratory analyses suggested potential benefits of CDR132L treatment in patients with advanced adverse remodeling at baseline, supporting further evaluation of CDR132L, including in chronic heart failure conditions. ClinicalTrials.gov: NCT05350969.

## Abstract (web summary)

As presented at the 2026 ESC Heart Failure Congress, in a phase 2 randomized trial in patients with heart failure with reduced left ventricular ejection fraction after myocardial infarction, treatment with CDR132L, an antisense oligonucleotide inhibitor of miR-132, was well tolerated but did not have a significant effect on measures of left ventricular structure or function.

## Main

Heart failure after MI remains a major cause of morbidity and mortality despite substantial advances in reperfusion strategies and guideline-directed medical therapy^1,2^. Adverse LV remodeling, characterized by ventricular dilatation, hypertrophy, fibrosis and contractile dysfunction, is the central pathophysiological process linking myocardial injury to progressive heart failure and death^3^. Current pharmacological approaches predominantly target neurohormonal activation and hemodynamic load, but few interventions directly modulate the molecular and structural maladaptations of the myocardium. Consequently, therapeutic options that effectively prevent or reverse post-infarction remodeling remain limited.

MicroRNAs (miRNAs) have emerged as key post-transcriptional regulators of cardiovascular biology. Among these, miR-132 is upregulated in response to myocardial stress and functions as a nodal driver of adverse remodeling^4,5^. Persistent miR-132 activation promotes cardiomyocyte hypertrophy, impaired calcium handling and contractility and myocardial fibrosis—all hallmarks of the transition from compensated injury to heart failure^5^. Inhibition of miR-132 in preclinical models halts and can partially reverse pathological remodeling, resulting in improved systolic and diastolic function^4,6^.

CDR132L is a synthetic, locked nucleic acid (LNA)-based antisense oligonucleotide specifically designed and optimized to inhibit miR-132. In extensive preclinical studies, CDR132L showed efficient myocardial uptake, dose-dependent miR-132 suppression and robust reversal of cardiac dysfunction in large animal models of ischemic and non-ischemic heart failure^6–8^. In a first-in-human phase 1b trial (NCT04045405), CDR132L was well tolerated at doses up to 10 mg kg^−1^, exhibited linear pharmacokinetics and achieved durable target engagement with dose-dependent reductions in circulating miR-132 (ref. ^9^).

On this basis, the HF-REVERT trial (NCT05350969, registered on 20 April 2022) was designed as an international, multicenter, randomized, double-blind, placebo-controlled phase 2 study to evaluate the efficacy and safety of CDR132L in patients with LV systolic dysfunction early after acute MI^10^. The trial enrolled patients with a left ventricular ejection fraction (LVEF) ≤45% within 14 days after the index event and elevated N-terminal pro B-type natriuretic peptide (NT-proBNP) and compared two intravenous dosing regimens of CDR132L (5 mg kg^−1^ and 10 mg kg^−1^ at weeks 0, 4 and 8) to placebo on top of contemporary standard of care. The primary endpoint was the percentage change in left ventricular end-systolic volume index (LVESVI) at 6 months, with secondary endpoints including additional echocardiographic measures, biomarkers, clinical events and patient-reported outcomes.

Here we report the results of HF-REVERT, the first randomized trial of a potentially disease-modifying antisense therapy directly targeting pathological cardiac remodeling after MI.

## Results

### Trial population

**Fig. 1:**
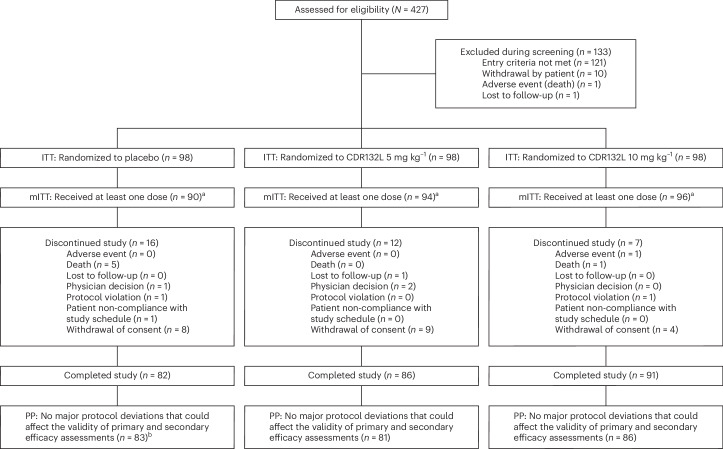
CONSORT diagram. ITT population: all patients who met the eligibility criteria and were randomized to study drug (CDR132L or placebo). mITT population: all randomized patients who received at least one dose of study drug (CDR132L or placebo). PP population: all patients from the ITT population who had completed treatments and the 6-month visit without any major protocol deviations that could affect the validity of primary and secondary efficacy assessments. Safety population: all randomized patients who received at least one dose of study drug (CDR132L or placebo) and had at least one post-dose safety assessment. Missing data for the primary endpoint were as follows: placebo, five missing; CDR132L 5 mg kg^−1^, nine missing; CDR132L 10 mg kg^−1^, seven missing. ^a^ The mITT population also served as the safety population, although one patient included in the CDR132L 10 mg kg^−1^ arm in the mITT population was included in the placebo arm in the safety population. ^b^ One patient included in the CDR132L 10 mg kg^−1^ arm in the mITT population was included in the placebo arm in the PP population.

Between July 2022 and March 2024, a total of 427 patients with acute MI were screened across 54 international sites. Of these, 294 patients met the eligibility criteria and were randomized to receive CDR132L 5 mg kg^−1^ (*n* = 98), CDR132L 10 mg kg^−1^ (*n* = 98) or placebo (*n* = 98) (Fig. 1).

The intention-to-treat (ITT) population comprised all randomized patients (*n* = 294). The modified intention-to-treat (mITT) population (*n* = 280), defined as all randomized patients who received at least one dose of study drug, was prespecified as the primary analysis population for efficacy endpoints. Fourteen randomized patients did not receive study treatment and were, therefore, excluded from the mITT population (Fig. 1).

The per-protocol (PP) population (*n* = 250) included patients from the ITT population who completed treatments and the 6-month visit without any major protocol deviations that could affect efficacy assessments. Exclusions were primarily due to treatment discontinuation, missing data or post-randomization events including cardiac resynchronization therapy (CRT) implantation, heart transplantation or death and were balanced across treatment groups; this cohort was used for prespecified supportive analyses (Fig. 1).

The safety population (*n* = 280) comprised all randomized patients who received at least one dose of study drug and had at least one post-dose safety assessment. This population was identical to the mITT population. However, one patient randomized to receive CDR132L 10 mg kg^−1^ erroneously received placebo and so was included in the placebo arm of the safety population, despite being in the CDR132L 10 mg kg^−1^ arm of the mITT population (Fig. 1).

Baseline demographic and clinical characteristics were generally well balanced across treatment groups in both the mITT and PP populations (Table 1 and Supplementary Tables 1 and 2). In the mITT population, the median age was 61.0 years (range, 35–80), and 13% were women based on sex at birth; gender data were not collected. A total of 81% of patients presented with ST-segment elevation myocardial infarction (STEMI). The median time from MI to randomization was 11.0 days (range, 4–15), consistent with the protocol-defined window of 3–14 days after the index event (with day of infarction counted as day 1).

**Table 1:** Baseline patient demographics and characteristics for the mITT population with data for each CDR132L dose analyzed separately

	CDR132L 5 mg kg^−1^ (*n* = 94)	CDR132L 10 mg kg^−1^ (*n* = 96)	Placebo (*n* = 90)	All patients (*n* = 280)
Median age^a^, years (min, max)	61.0 (35, 80)	62.0 (37, 79)	62.0 (36, 79)	61.0 (35, 80)
Sex at birth, *n* (%)				
Male	83 (88)	81 (84)	81 (90)	245 (88)
Female	11 (12)	15 (16)	9 (10)	35 (13)
Baseline NT-proBNP level, pg ml^−1^, *n* (%)^b^				
≤1,307	44 (47)	49 (51)	47 (52)	140 (50)
>1,307	50 (53)	47 (49)	43 (48)	140 (50)
Median days between index AMI and randomization^c^ (min, max)	11.0 (4, 15)	10.0 (4, 14)	11.0 (5, 15)	11.0 (4, 15)
Index AMI, *n* (%)				
STEMI	78 (83)	81 (84)	69 (77)	228 (81)
NSTEMI	16 (17)	15 (16)	21 (23)	52 (19)
Number of previous infarctions, *n* (%)				
0	81 (86)	86 (90)	72 (80)	239 (85)
≥1	13 (14)	10 (10)	18 (20)	41 (15)
Killip classification, *n* (%)				
Class I	72 (77)	79 (82)	68 (76)	219 (78)
Class II	18 (19)	15 (16)	19 (21)	52 (19)
Class III/IV	4 (4)	2 (2)	3 (3)	9 (3)
NYHA class, *n* (%)				
Class I	35 (37)	43 (45)	29 (32)	107 (38)
Class II	52 (55)	49 (51)	57 (63)	158 (56)
Class III	7 (7)	3 (3)	4 (4)	14 (5)
Class IV	0 (0)	0 (0)	0 (0)	0 (0)
Median LVEF, %^d^ (min, max)	36.49 (19.28, 54.97)	36.55 (13.11, 44.81)	34.64 (21.38, 45.00)	35.86 (13.11, 54.97)
LVEF, %, *n* (%)				
≤35.86	46 (49)	43 (45)	51 (57)	140 (50)
>35.86	48 (51)	53 (55)	39 (43)	140 (50)
Median LVESVI, ml m^−^^2^ (min, max)	41.90 (19.19, 106.81)	42.20 (17.87, 85.80)	43.61 (24.33, 85.33)	42.78 (17.87, 106.81)
Location of infarction at randomization, *n* (%)				
Anterior	67 (71)	68 (71)	64 (71)	199 (71)
Non-anterior	27 (29)	28 (29)	26 (29)	81 (29)
Troponin T (central), ng l^−1^, *n* (%)				
≤208.5	46 (49)	48 (50)	46 (51)	140 (50)
>208.5	48 (51)	48 (50)	44 (49)	140 (50)

^a^ Age at time of consent; patients enrolled were aged ≥30 to ≤80 years.

^b^ Baseline NT-proBNP values are based on central laboratory analyses and may differ from local screening values used for eligibility assessment.

^c^ Index AMI date to randomization date.

^d^ One patient had a baseline LVEF >45% due to an initial central echocardiography reading error identified during final QC; the patient was randomized based on the original eligibility confirmation and was retained in the PP and mITT populations. All other randomized patients had an LVEF ≤45%.

AMI, acute myocardial infarction; max, maximum; min, minimum; QC, quality control.

**Extended Data Fig. 1:**
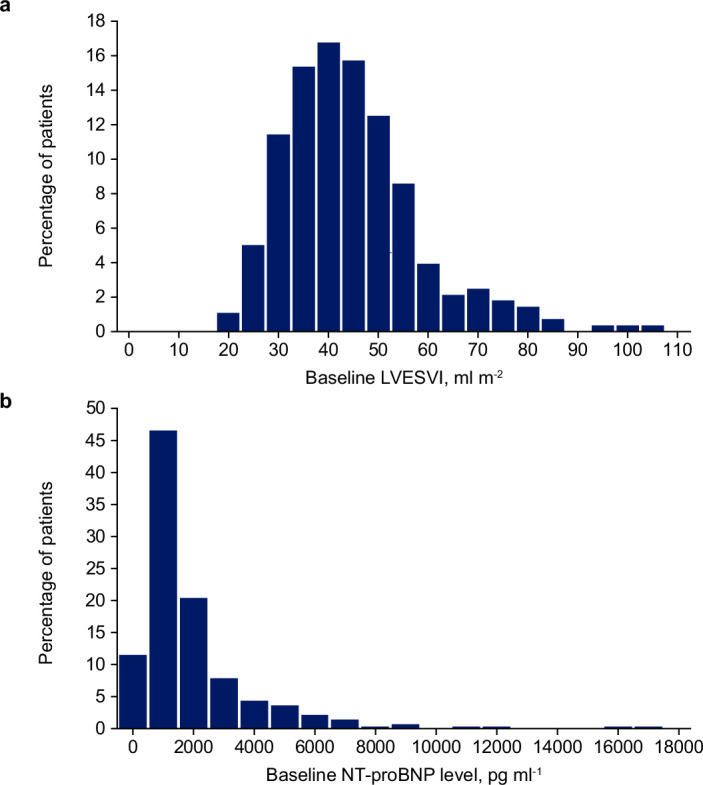
Baseline distributions of LVESVI (a) and NT-proBNP (b). Data are shown for the mITT population. LVESVI, left ventricular end-systolic volume index; mITT, modified intention-to-treat; NT-proBNP, N-terminal pro B-type natriuretic peptide.

Markers of cardiac dysfunction reflected a population with overall moderate impairment but substantial heterogeneity. Median baseline LVEF was 36% (range, 13.1–55.0; see legend of Table 1), and median NT-proBNP concentration was 1,307 pg ml^−1^ (range, 55.0–17,451.0). Baseline distributions of LVESVI and NT-proBNP are shown in Extended Data Fig. 1. Approximately half of the study population had LVESVI values within the normal to moderately elevated range, indicating that a substantial proportion of patients had not yet developed advanced ventricular remodeling at the time of randomization.

Background therapy was consistent with contemporary post-MI guideline-directed care and was balanced across groups, with more than 90% of patients receiving beta-blockers and more than 90% receiving angiotensin-converting enzyme inhibitors, angiotensin receptor blockers or angiotensin receptor–neprilysin inhibitors as well as 84% receiving mineralocorticoid receptor antagonists. A high number of patients (79%) additionally received sodium-glucose co-transporter-2 (SGLT2) inhibitors on top of current standard of care (Extended Data Table 1). Study drug adherence was high across treatment groups: 82 patients (84%) in the placebo group, 86 patients (88%) in the 5 mg kg^−1^ group and 91 patients (93%) in the 10 mg kg^−1^ group completed the study (Fig. 1).

### CDR132L target engagement

**Fig. 2:**
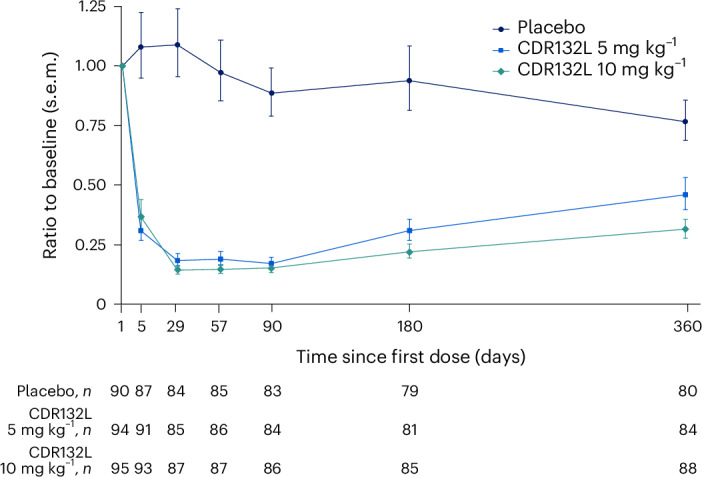
Concentration of miR-132 in plasma derived from patients treated with placebo, CDR132L 5 mg kg^−1^ or CDR132L 10 mg kg^−1^. Data are shown for the mITT population with each CDR132L dose analyzed separately.

Based on previous studies in pigs, plasma miR-132 concentrations may serve as a marker for target engagement in cardiac tissue^4,6^. Plasma miR-132 decreased in a dose-dependent manner after administration of CDR132L, with the greatest reductions observed in the 10 mg kg^−1^ group (Fig. 2). These changes were evident after the first infusion and persisted through month 12, suggesting sustained inhibition of the miR-132 pathway. Cardiac tissue miR-132 concentrations were not assessed. At months 6 and 12, plasma miR-132 concentrations increased compared to month 3, especially in the 5 mg kg^−1^ group. Interindividual variability was observed, but overall suppression of circulating miR-132 aligned with the expected pharmacodynamic profile. However, circulating miR-132 represents a systemic biomarker and may not directly reflect myocardial tissue levels, which are the biologically relevant compartment for ventricular remodeling.

### Primary and secondary endpoints

The primary endpoint was analyzed using a one-sided testing framework (*α* = 0.025) with hierarchical dose testing. All *P* values are nominal and not adjusted for multiplicity.

**Fig. 3:**
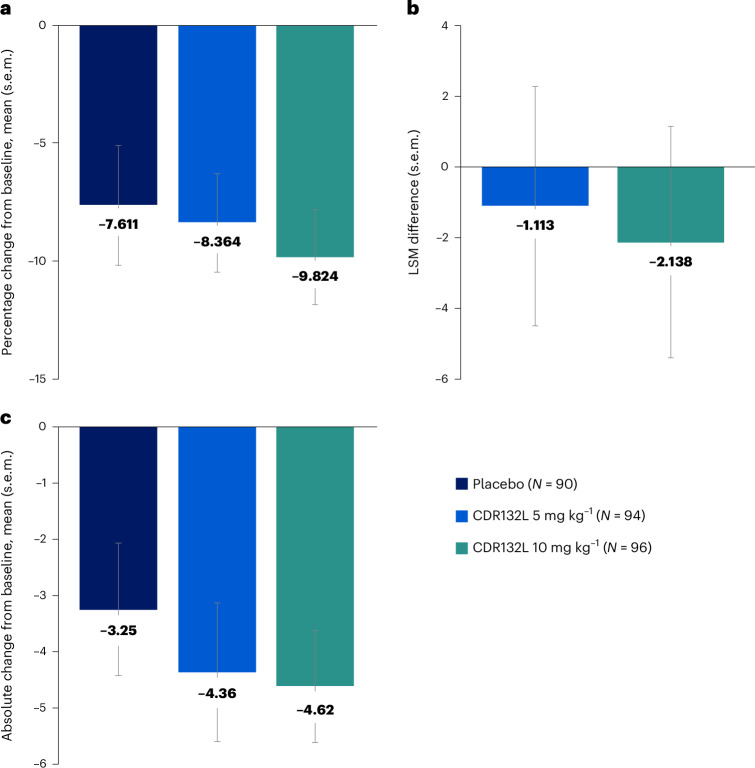
Changes in LVESVI in patients treated after MI with placebo, CDR132L 5 mg kg^−1^ or CDR132L 10 mg kg^−1^. Percentage change from baseline in LVESVI at month 6 (**a**), LSM difference versus placebo at month 6 (**b**) and absolute change from baseline in LVESVI at month 6 (**c**). Data are shown for the mITT population with each CDR132L dose analyzed separately. In **b**, LSM difference was determined using an ANCOVA model including treatment and center group, stratification factors of age group (<60 years, ≥60 years) and location of infarction (anterior, non-anterior) as fixed effects and baseline LVESVI as a covariate (comparisons shown versus placebo).

**Extended Data Fig. 2:**
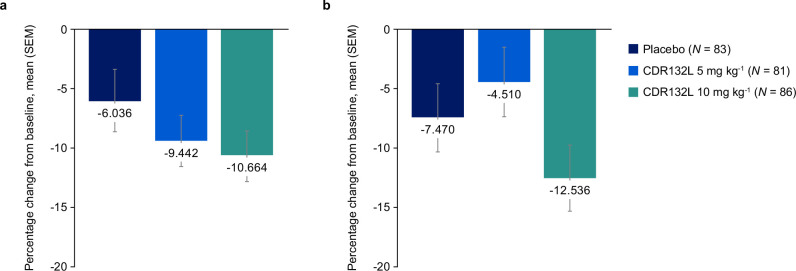
Percentage change from baseline in LVESVI at month 6 (a) and month 12 (b). Data are shown for the PP population with each CDR132L dose analyzed separately. LVESVI, left ventricular end-systolic volume index; PP, per protocol; SEM, standard error of the mean.

The percentage change in LVESVI from baseline to 6 months in the mITT population served as the primary endpoint. Across the three treatment groups, LVESVI decreased modestly over 6 months (Fig. 3a). The resulting placebo-adjusted least squares mean (LSM) differences were −1.11% (s.e.m. 3.37; *F*-test *P* = 0.37) for 5 mg kg^−1^ and −2.14% (s.e.m. 3.27; *F*-test *P* = 0.26) for 10 mg kg^−1^ (Fig. 3b). The reductions in LVESVI also remained stable after 12 months (Supplementary Table 3). A prespecified PP analysis yielded numerically larger and dose-dependent reductions in LVESVI for both CDR132L doses at month 6 compared to placebo (Extended Data Fig. 2a).

Absolute changes in LVESVI are depicted in Fig. 3c and Supplementary Table 3; findings were consistent across both metrics. Missing data for the primary endpoint were limited (placebo, five missing; CDR132L 5 mg kg^−1^, nine missing; CDR132L 10 mg kg^−1^, seven missing) and balanced across groups; sensitivity analyses using mixed models for repeated measures (MMRM) and multiple imputation produced consistent results and did not change the interpretation of the primary endpoint.

Prespecified confirmatory secondary endpoints included absolute changes from baseline to month 6 for LVEF, NT-proBNP, global longitudinal strain (GLS) and Kansas City Cardiomyopathy Questionnaire (KCCQ) overall summary score for pooled doses of CDR132L against placebo. No statistically significant differences were observed between study groups (Table 2). Supportive secondary endpoints of changes in LVEF, NT-proBNP, LVESVI and troponin T levels for individual doses of CDR132L are shown in Supplementary Table 3. GLS exhibited a greater numerical improvement in the CDR132L-treated groups compared to placebo at month 6 (absolute change, −2.62 versus −1.62), although the difference was not statistically significant (Table 2). LVEF increased from baseline to months 6 and 12 in all groups, with absolute LVEF changes at month 6 of 7.2%, 7.7% and 8.4% in the placebo, 5 mg kg^−1^ and 10 mg kg^−1^ groups, respectively (Supplementary Table 3). In the PP population, LVEF responses were larger and dose dependent, with absolute increases of approximately 7.0%, 8.4% and 8.9% at month 6 and 7.7%, 8.0% and 10.6% at month 12 for placebo, 5 mg kg^−1^ and 10 mg kg^−1^ groups, respectively (Extended Data Table 2). NT-proBNP decreased from baseline to months 6 and 12 in all treatment groups (Table 2 and Supplementary Table 3). High-sensitivity troponin levels showed post-MI declines over time with no significant differences between CDR132L treatment groups (Supplementary Table 3). KCCQ overall summary scores increased from baseline to months 6 and 12 across all treatment groups (Table 2 and Supplementary Table 3).

**Table 2:** Confirmatory secondary endpoints

	CDR132L (pooled) (*n* = 190)	Placebo (*n* = 90)
LVEF, %, absolute change from baseline to month 6 (LOCF)		
*n*	190	90
Mean (s.d.)	8.03 (8.86)	7.20 (8.98)
LSM difference, CDR132L versus placebo (95% CI; one-sided *P* value)	0.87 (−1.41, 3.15; *P* = 0.227)	
NT-proBNP, pg ml^−1^, logarithmic scale, absolute change from baseline to month 6 (LOCF)		
*n*	190	90
Mean (s.d.)	−1.046 (0.89)	−1.108 (0.87)
Ratio (CDR132L / placebo) (95% CI; one-sided *P* value)	1.11 (0.91, 1.37; *P* = 0.850)	
GLS, absolute change from baseline to month 6 (LOCF)		
* n*	190	90
Mean (s.d.)	−2.62 (3.68)	−1.62 (3.03)
LSM difference, CDR132L versus placebo (95% CI; one-sided *P* value)	−0.52 (−1.34, 0.30; *P* = 0.108)	
KCCQ overall summary score, absolute change from baseline to month 6 (LOCF)		
* n*	187	87
Mean (s.d.)	7.728 (20.30)	11.698 (18.35)
LSM difference, CDR132L versus placebo (95% CI; one-sided *P* value)	−4.01 (−8.10, 0.07; *P* = 0.973)	

Data are shown for the mITT population with CDR132L doses pooled.

CI, confidence interval.

### Subgroup analyses

**Fig. 4:**
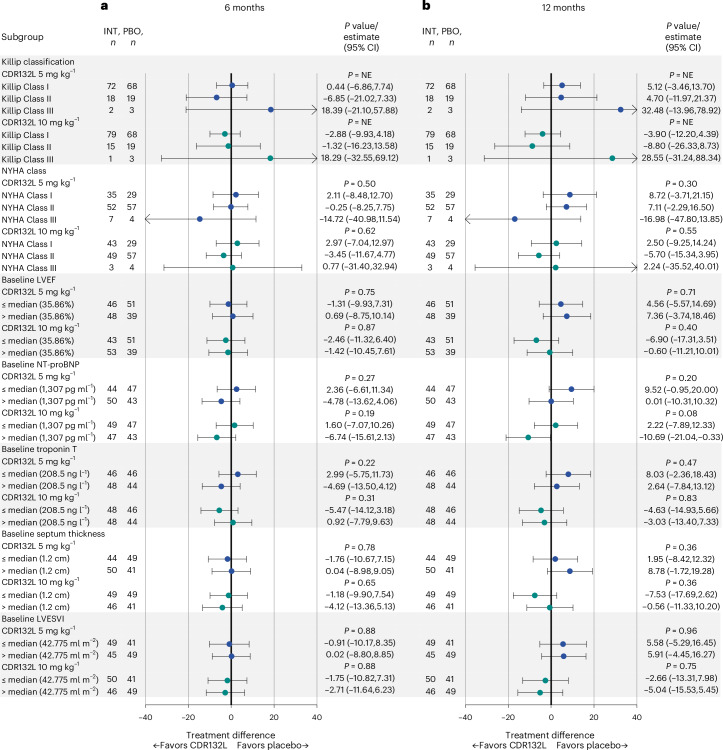
Subgroup analyses for percentage change from baseline in LVESVI. The difference in LSM (95% CI) between treatment arms at month 6 (**a**) and at month 12 (**b**) for selected subgroups is shown. Data are shown for the mITT population with each CDR132L dose analyzed separately. The ANCOVA model (used for all displayed subgroup analyses) includes treatment and center group, stratification factors of age group (<60 years, ≥60 years) and location of infarction (anterior, non-anterior), baseline LVESVI and subgroup and treatment-by-subgroup interaction. *P* values are two-sided. All comparisons shown are versus placebo. CI, confidence interval; INT, intervention; NE, not estimable; PBO, placebo.

**Extended Data Fig. 3:**
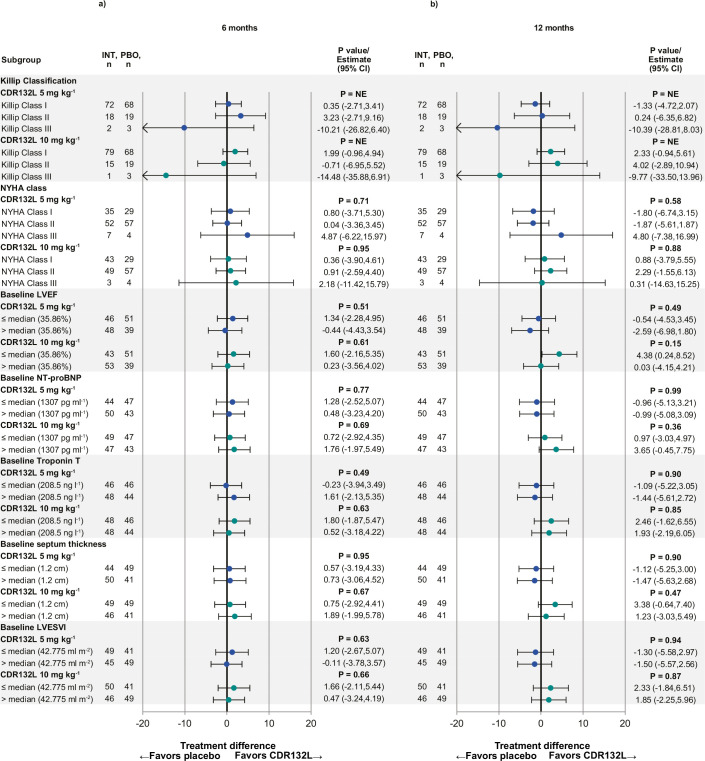
Subgroup analyses for change from baseline in LVEF. (**a, b**) The difference in LSM (95% CI) between treatment arms at month 6 (**a**) and month 12 (**b**) for selected subgroups is shown. Data are shown for the mITT population with each CDR132L dose analyzed separately. The ANCOVA model (used for all displayed subgroup analyses) includes treatment and center group, stratification factors of age group (<60 years, ≥60 years) and location of infarction (anterior, non-anterior), baseline LVEF, subgroup and treatment-by-subgroup interaction. *P* values are 2-sided. All comparisons shown are versus placebo. ANCOVA, analysis of covariance; CI, confidence interval; INT, intervention; LSM, least squares mean; LVEF, left ventricular ejection fraction; LVESVI, left ventricular end-systolic volume index; mITT, modified intention-to-treat; NE, not evaluable; NT-proBNP, N-terminal pro B-type natriuretic peptide; NYHA, New York Heart Association; PBO, placebo.

**Extended Data Fig. 4:**
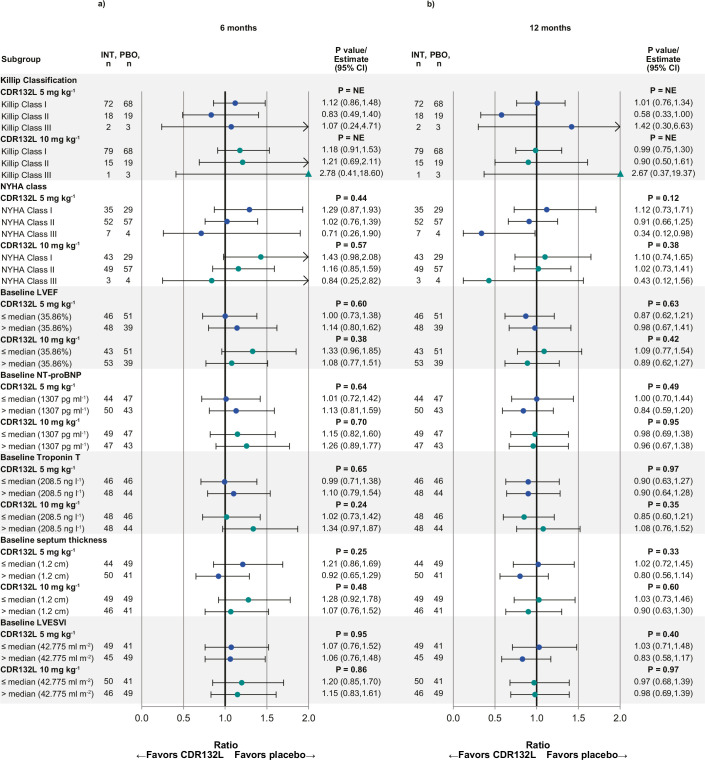
Subgroup analyses for NT-proBNP ratio to baseline. (**a,b**) The difference in LSM (95% CI) between treatment groups at month 6 (**a**) and month 12 (**b**) for selected subgroups is shown. Data are shown for the mITT population with each CDR132L dose analyzed separately. The ANCOVA model includes treatment and center group, stratification factors of age group (<60 years, ≥60 years) and location of infarction (anterior, non-anterior), baseline NT-proBNP (log transformed), subgroup and treatment-by-subgroup interaction. *P* values are 2-sided. All comparisons shown are versus placebo. Triangles represent estimates that fall outside the x axis range. ANCOVA, analysis of covariance; CI, confidence interval; INT, intervention; LSM, least squares mean; LVEF, left ventricular ejection fraction; mITT, modified intention-to-treat; NE, not evaluable; NT-proBNP, N-terminal pro B-type natriuretic peptide; NYHA, New York Heart Association; PBO, placebo.

Exploratory analyses in prespecified subgroups using similar methods to the primary endpoint analyses were performed to explore whether treatment effects on LVESVI as the primary endpoint varied according to baseline disease severity and other clinically relevant characteristics. These analyses, summarized in forest plots for the mITT population (Fig. 4 and Supplementary Fig. 1), evaluated treatment-by-subgroup interactions across various demographic and clinical categories. Two severity-defined and clinically important subgroups with higher anticipated mortality risk demonstrated directionally consistent numerical trends favoring CDR132L treatment. The analysis of an important post hoc-defined subgroup of patients (analogous to prespecified LVEF and NT-proBNP subgroups) who were post-MI with baseline LVESVI above the median showed that CDR132L produced larger reductions in LVESVI compared to placebo, with effect sizes favoring the 10 mg kg^−1^ dose (Fig. 4 and Supplementary Fig. 1). Similarly, patients with baseline NT-proBNP concentrations above the median showed improvements in LV remodeling (greater LVESVI reduction; Fig. 4 and Supplementary Fig. 1) and functional parameters (greater increase in LVEF; Extended Data Fig. 3 and Supplementary Fig. 2) after CDR132L treatment compared to placebo. In these high-risk subgroups, the magnitude of LVESVI reduction and LVEF increase was generally larger in the PP population than in the mITT population (Fig. 4, Extended Data Fig. 3 and Supplementary Figs. 1−4). The same trends on cardiac remodeling were observed in analyses to explore subgroup effects on NT-proBNP changes (Extended Data Fig. 4 and Supplementary Figs. 5 and 6). Given the limited sample size, multiple testing and the potential for selection bias, these analyses should be considered exploratory and hypothesis generating only and should not be interpreted as evidence of efficacy.

### Safety and tolerability of CDR132L treatment

Safety assessments were conducted in all patients who received at least one dose of study drug (*n* = 280). Two observation periods were defined: the in-trial period, from randomization to the date of last contact with the trial site, and the on-treatment period, from the first dose of study treatment to the last dose plus 30 days.

CDR132L was well tolerated at both dose levels, with an overall adverse event profile similar to placebo (Extended Data Table 3 and Supplementary Table 4). Most adverse events were mild or moderate in intensity. The incidence of treatment-emergent adverse events was 44%, 50% and 54% in the placebo, 5 mg kg^−1^ and 10 mg kg^−1^ groups, respectively. Serious adverse events occurred at similar rates across treatment arms, without evidence of dose dependency.

A total of six deaths occurred after treatment start: five in the placebo group (all cardiovascular related) and one in the 10 mg kg^−1^ group (lung cancer) (Extended Data Table 3). No cardiovascular deaths were reported in either CDR132L treatment group. Given the low number of events, the study was not powered to assess differences in clinical outcomes, and these findings should be interpreted descriptively. One additional patient died during the screening period.

The hazard ratio (95% confidence interval) of time to first event of heart failure for the mITT population was 0.874 (0.349, 2.184) for the 10 mg kg^−1^ and 0.938 (0.387, 2.274) for the 5 mg kg^−1^ collective.

There were no signals of organ-specific toxicity, including renal or hepatic function or hematologic parameters (Supplementary Table 4). No clinically significant arrhythmias were attributed to CDR132L. There were few infusion-related adverse events (Supplementary Table 4), mostly mild skin reactions, which occurred more frequently with CDR132L than with placebo across both mITT and PP populations. Discontinuation of study treatment due to treatment-emergent adverse events occurred in 2%, 7% and 4% of patients in the placebo, 5 mg kg^−1^ and 10 mg kg^−1^ groups, respectively (Extended Data Table 3), and discontinuations from the study due to adverse events were rare (<1% overall).

## Discussion

In this randomized, double-blind, placebo-controlled phase 2 trial in patients with LV systolic dysfunction early after MI, treatment with the antisense oligonucleotide CDR132L was well tolerated and was associated with dose-dependent reductions in circulating miR-132, indicating pharmacodynamic target engagement. The trial did not meet its primary endpoint: the percentage change in LVESVI at 6 months was not significantly different between either CDR132L dose and placebo. Secondary endpoints, including LVEF, NT-proBNP, LV volumes, GLS and patient-reported outcomes, showed modest improvements in all groups, without statistically significant treatment differences.

The overall neutral result should be interpreted in the context of trial design features that likely reduced sensitivity to detect an incremental treatment effect in the overall population. The study enrolled patients early after MI, at a time when a substantial proportion had not yet developed advanced ventricular remodeling. In addition, the population was exceptionally well treated with contemporary guideline-directed therapy, including frequent initiation of SGLT2 inhibitors, which are not formally guideline recommended in the immediate post-MI setting but are increasingly recognized for their favorable effects on ventricular remodeling^11^. This high level of background therapy likely reduced the residual modifiable risk and may have limited the ability to detect incremental structural benefit of CDR132L in the complete study population. Finally, CDR132L treatment stopped after the third dose, and, thus, treatment duration might have been too short to see significant reduction of pathological remodeling at the 6-month and 12-month timepoints.

Exploratory prespecified and post hoc subgroup analyses suggested numerical differences in LV remodeling parameters among CDR132L-treated patients with higher baseline disease severity, including those with elevated LVESVI or NT-proBNP. However, no statistically significant treatment-by-subgroup interactions were observed in the mITT populations. The larger apparent and partially significant effects in the PP population are interesting but should be considered exploratory and hypothesis generating and should not be interpreted as evidence of efficacy.

The biological rationale for targeting miR-132 in post-infarction remodeling is supported by preclinical studies demonstrating the role of miR-132 in regulating pathways involved in hypertrophy, fibrosis, calcium handling and autophagy^4–6,12^. Thus, patients with strong ongoing pathological cardiac remodeling may benefit most from this therapeutic concept. CDR132L treatment resulted in sustained, long-term, dose-dependent suppression of circulating miR-132, consistent with pharmacodynamic activity. However, circulating miR-132 represents a systemic biomarker and may not directly reflect myocardial tissue levels, which are the biologically relevant compartment for ventricular remodeling. Although correlations between plasma and cardiac tissue miR-132 levels have been demonstrated in preclinical models^4–6^, this relationship has not been established in humans. Thus, the observed target engagement does not necessarily imply sufficient modulation of myocardial remodeling processes.

Several additional factors may have influenced the ability to detect a significant treatment effect in the overall study population. First, although the study was powered based on expected effect sizes from earlier trials, the degree of reverse remodeling observed in the placebo group was greater than anticipated, consistent with the intensity of background therapy. Second, despite the inclusion criterion of LVEF ≤45%, approximately half of the study population had LVESVI values within the normal to moderately elevated range at baseline, reflecting early enrollment after MI before advanced remodeling had developed. This limited dynamic range may have reduced sensitivity to detect further improvements. Third, mean baseline NT-proBNP levels were numerically lower in the 10 mg kg^−1^ group, reflecting expected variability in a study of this size. The higher mean troponin T level at month 6 in the 5 mg kg^−1^ group was driven by a small number of outliers (which we interpret as likely reflecting random variability in the relatively small sample rather than a meaningful biological signal), with otherwise similar distributions across groups. Analyses were adjusted for baseline values, and findings should be interpreted with caution given the potential impact of baseline differences. Fourth, the timing and duration of treatment may not have been optimal. Treatment was initiated a median of 11 days after MI and completed after 8 weeks, leaving a limited period of sustained exposure before the 6-month endpoint. Fifth, only 13% of participants were women based on sex at birth. Data on gender were not collected. This limits the applicability of the results to female patients.

Preclinical data suggest that prolonged exposure to CDR132L may be required to achieve maximal effects on ventricular remodeling^6^. In large animal models, extended treatment duration was associated with more sustained myocardial drug levels and greater effects on cardiac structure and function^6^. In the present study, circulating miR-132 suppression was sustained but showed partial rebound after treatment cessation, which may be consistent with the pharmacokinetic profile of the compound. These findings raise the possibility that longer treatment duration or maintenance dosing could enhance biological effects and should be evaluated in future studies. The safety profile of CDR132L was favorable, with no evidence of dose-limiting toxicity or organ-specific safety concerns. Although a numerical imbalance in cardiovascular deaths was observed, with all events occurring in the placebo group, the total number of events was low, and the study was not powered to assess clinical outcomes. Therefore, these findings should be interpreted as descriptive only.

In conclusion, in this phase 2 trial in patients with LV systolic dysfunction after MI, CDR132L demonstrated favorable safety and pharmacodynamic target engagement but did not improve ventricular remodeling compared to placebo at 6 months. The subgroup findings are exploratory and hypothesis generating only. Together with the observations regarding patient selection, background therapy and treatment duration, these results support continued clinical investigation under optimized trial conditions rather than definitive conclusions regarding clinical efficacy. Ongoing studies are evaluating CDR132L in chronic heart failure populations (patients with heart failure with preserved LVEF (NCT06979362) and patients with heart failure with reduced LVEF (NCT06979375)).

## Methods

### Ethical approval statement

The study protocol, all study protocol amendments, written study participant information, informed consent form, Investigator’s Brochure and any other relevant documents were reviewed and approved by an independent ethics committee or institutional review board at each study site.

This study was conducted in accordance with the study protocol and all approved amendments; the International Council for Harmonisation (ICH) Guideline for Good Clinical Practice E6(R2); the ethical principles of the Declaration of Helsinki; the Council for International Organizations of Medical Sciences, International Ethical Guidelines; all applicable local laws and regulations; and ICH requirements for archiving and retention of essential documents.

### Study design and oversight

HF-REVERT was an international, multicenter, randomized, double-blind, placebo-controlled, parallel-group phase 2 trial evaluating two dose levels of CDR132L in patients with LV systolic dysfunction after MI. The trial was conducted at 54 study sites (that is, study sites that consented at least one patient) in eight countries (Czech Republic, Germany, Greece, Hungary, The Netherlands, Poland, Spain and the United Kingdom). Recruitment took place between July 2022 and March 2024.

Patients were assigned in a 1:1:1 ratio to CDR132L 5 mg kg^−1^, CDR132L 10 mg kg^−1^ or placebo, administered as three intravenous infusions 28 days apart, on top of contemporary standard of care. The trial consisted of a screening period (3–14 days after MI diagnosis), a 6-month double-blind treatment period and a 6-month follow-up, with the end-of-study visit at month 12.

The trial was conducted in accordance with the Declaration of Helsinki and Good Clinical Practice guidelines. All patients provided written informed consent. An independent data monitoring committee oversaw patient safety. Echocardiographic imaging was analyzed at a blinded core laboratory at Brigham and Women’s Hospital, Harvard Medical School, using standardized acquisition and interpretation protocols.

The trial protocol and the statistical analysis plan are available in Supplementary Note 1 and Supplementary Note 2, respectively.

### Patients

Eligible participants were adults aged 30–80 years with a spontaneous type 1 MI (STEMI or non-ST-segment elevation myocardial infarction (NSTEMI)), randomized 3–14 days after the index event. Patients were required to have an LVEF ≤45% confirmed on core laboratory echocardiography, NT-proBNP ≥125 pg ml^−1^ and <8,000 pg ml^−1^ and significantly elevated troponin levels (for NSTEMI, ≥5× the upper limit of normal). All patients underwent percutaneous coronary intervention or diagnostic angiography for the index event.

Major exclusion criteria included non-ischemic cardiomyopathy; prior LVEF <30% within 6 months; New York Heart Association (NYHA) Class IV; planned cardiac surgery; severe valvular disease; hemodynamic instability; significant renal impairment (estimated glomerular filtration rate <30 ml min^−1^ 1.73 m^−2^) or hepatic impairment (Child–Pugh B/C); active viral infections; recent major neurological events; thrombocytopenia; uncontrolled diabetes; epilepsy; unstable psychiatric or medical conditions; and clinically significant electrocardiogram abnormalities.

The complete inclusion and exclusion criteria are provided in Extended Data Table 4. Data on gender were not collected. Some further exploratory analyses were performed stratified by sex for key endpoints, including LVESVI and LVEF, but the small number of treated women does not allow a meaningful conclusion.

### Randomization and masking

Patients were randomized centrally using a computer-generated schedule stratified by age group (<60 years, ≥60 years) and location of infarction (anterior, non-anterior). Investigators, patients, core laboratory personnel and the sponsor remained blinded until all patients had completed month 6. The study drug was administered in a double-blind fashion whereby patients and clinical study site staff members were blinded to the study treatment. The pharmacy staff members who prepared the study drug were not blinded to study drug assignment.

### Echocardiographic assessment

Transthoracic echocardiograms were obtained at baseline and at months 3, 6 and 12 using a standardized imaging protocol. All study sonographers at study sites were sufficiently trained and had to be certified before study start. All echocardiograms obtained at study sites were transferred to the Brigham/Harvard core laboratory for blinded analysis.

LVESVI and LVEF were quantified using the Simpson’s rule algorithm.

Other echocardiographic parameters were assessed by the core laboratory’s subset of standard echocardiographic examinations.

### miRNA measurements in plasma

Plasma miR-132 levels were quantified at Biotype GmbH using the CardiorHealth miR-132 Plasma PCR Kit (Cardior Pharmaceuticals GmbH) in accordance with the manufacturer’s instructions. The assay is a CE-marked in vitro diagnostic based on quantitative real-time polymerase chain reaction (PCR) for specific detection of miR-132-3p in plasma from patients with established heart failure or at risk of heart failure after MI.

Total RNA was isolated from 150 µl of EDTA plasma spiked with cel-miR-39-3p using the miRNeasy Serum/Plasma Advanced Kit (Qiagen). Reverse transcription was performed in a single reaction using the TaqMan MicroRNA Reverse Transcription Kit (Thermo Fisher Scientific) with miR-132-3p and cel-miR-39-3p specific reverse transcription primers. Quantitative PCR was carried out on a LightCycler 480 II instrument (Roche) using unlabeled forward and reverse primers and a FAM-labeled TaqMan MGB probe, with each microRNA analyzed in separate reactions on the same plate. Results were reported as *C*_p_ values, and relative plasma miR-132 levels were calculated as 2 − Δ*C*_p_ (2 − (*C*_p_ miR-132 – *C*_p_ cel-miR-39)).

### Statistical analysis

The primary endpoint was the percentage change in LVESVI from baseline to month 6. The primary analysis used an analysis of covariance (ANCOVA) model that included treatment as a fixed effect and baseline LVESVI as a covariate. The analysis was also adjusted for center group and stratification factors of age group (<60 years, ≥60 years) and location of infarction (anterior, non-anterior). The LSM estimates for each treatment arm are displayed together with standard errors and their corresponding 95% confidence intervals. Treatment differences with 95% confidence intervals were also produced.

All observed data were included in the analysis regardless of post-randomization events such as treatment discontinuations or hospitalizations for CRT implants or heart transplantations. Missing post-baseline data through month 6 were imputed using the last observation (including baseline) carried forward (LOCF) approach. In addition, MMRM was used for sensitivity analyses. The model used treatment, timepoint, treatment-by-month interaction, center group, stratification factors of age group (<60 years, ≥60 years) and location of infarction (anterior, non-anterior) and baseline LVESVI.

The primary analysis set was the mITT population (all randomized patients who received at least one dose of study drug (CDR132L or placebo)). The safety population comprised all randomized patients who received at least one dose of study drug and had at least one post-dose safety assessment. Prespecified supportive analyses were performed in addition in the PP population, which included patients who completed treatment and 6-month visit without any major protocol deviations.

Supportive analyses were performed for the PP population in a similar manner to the primary efficacy analysis.

Predefined subgroup analyses of the primary endpoint were performed at month 6 and month 12 in the mITT and PP populations. Subgroup analyses of the secondary endpoints (that is, absolute and relative change from baseline in LVESVI, LVEF and NT-proBNP) were also performed at months 6 and 12 in the mITT and PP populations.

Predefined exploratory subgroup analyses based on age group, sex, MI type, time of first treatment after MI event, patients with and without treatment of SGLT2 inhibitors and disease severity, including patients with baseline LVEF values and NT-proBNP concentrations above/below the median, were performed in an exploratory fashion for secondary endpoints.

However, only one important post hoc-defined subgroup was analyzed: patients post-MI with baseline LVESVI above/below the median. This subgroup was analogous to predefined subgroups of LVEF and NT-proBNP (above/below median), and it was analyzed in a similar manner to the primary efficacy analysis. The subgroup analysis used an ANCOVA model that included treatment and center group, stratification factors of age group (<60 years, ≥60 years) and location of infarction (anterior, non-anterior), baseline LVESVI, subgroup and treatment-by-subgroup interaction.

All analyses performed used SAS version 9.4 software.

### Sample size determination

Sample size was determined using a one-sided two-sample *t*-test with a significance level of 2.5%. Based on assumed mean LVESVI changes of 1% for placebo, 5% for CDR132L 5 mg kg^−1^ and 6% for CDR132L 10 mg kg^−1^, each with a common s.d. of 9%, a sample size of 90 patients per group provided 96.0% power to detect a 5-percentage-point difference between 10 mg kg^−1^ and placebo (step 1) and 84.3% power to detect a 4-percentage-point difference between 5 mg kg^−1^ and placebo (step 2), within a hierarchical testing procedure. This resulted in an overall power of 80.9%. To account for early dropouts, the planned sample size was increased to 294 patients.

### Secondary endpoints

Secondary endpoints (changes from baseline to months 6 and 12 in LVEF, NT-proBNP, troponin T, GLS and KCCQ, and change from baseline to month 12 in LVESVI) were analyzed using ANCOVA, with change scores expressed as LSMs with standard errors and 95% confidence intervals. No adjustment for multiple comparisons was prespecified.

### Safety

The safety of CDR132L was assessed by continuous monitoring of adverse events and abnormalities in clinical laboratory assessments, vital signs, physical examination, electrocardiograms and urinalysis.

Adverse events were reported by the patient (or, when appropriate, by a caregiver, surrogate or the patient’s legally authorized representative).

The investigator was responsible for detecting, documenting and recording events that met the definition of an adverse event or a serious adverse event and remained responsible for following-up adverse events that were serious, that were considered related to the study treatment or study procedures or that caused the patient to discontinue the study treatment.

Adverse events were summarized with the number of patients, percentage of patients, number of events and the rate of events.

The rate of events was calculated as (number of events / patient years of exposure), where patient years of exposure = (last dose date – first dose date + 31) / 365.25.

At each level of summarization, a patient was counted once if he/she reported one or more events. The severity grade and relationship to study drug were summarized in a similar manner.

All safety analyses were based on the safety population. No formal statistical analysis of the safety data was performed.

### Reporting summary

Further information on research design is available in the Nature Portfolio Reporting Summary linked to this article.

## Online content

Any methods, additional references, Nature Portfolio reporting summaries, source data, extended data, supplementary information, acknowledgements, peer review information; details of author contributions and competing interests; and statements of data and code availability are available at 10.1038/s41591-026-04408-4.

## Supplementary information

Supplementary Information Supplementary Tables 1–4, Supplementary Figs. 1–6, Supplementary Note 1 (redacted protocol) and Supplementary Note 2 (redacted statistical analysis plan).

Reporting Summary

## Data Availability

Researchers can request access to clinical trial data by submitting a research proposal for review and approval by Novo Nordisk and an internal independent review panel. Requests are considered after the research is concluded or completed and the main results have been published. If the research supports a regulatory application, requests will be considered after the product and its intended use are approved in both the European Union and the United States. Participants’ clinical data will be anonymized, following an approved internal process, before data are shared to external third parties. For details on how to request access to clinical data, visit https://www.novonordisk-trials.com/.

## Extended data

**Extended Data Table 1:** Concomitant medication usage in the safety population with data for each CDR132L dose analyzed separately

Category	CDR132L 5 mg kg^−1^ (*n* = 94)	CDR132L 10 mg kg^−1^ (*n* = 95)	Placebo (*n* = 91)	All patients (*n* = 280)
Beta-blockers, selective, *n* (%)	88 (94)	88 (93)	88 (97)	264 (94)
Mineralocorticoid receptor antagonists, *n* (%)	80 (85)	79 (83)	75 (82)	234 (84)
ACE inhibitors and angiotensin receptor blockers, *n* (%)	91 (97)	92 (97)	87 (96)	270 (96)
ACE inhibitors, plain, *n* (%)	68 (72)	65 (68)	67 (74)	200 (71)
Angiotensin II receptor blockers, plain, *n* (%)	16 (17)	24 (25)	21 (23)	61 (22)
Angiotensin II receptor blockers, other combinations, *n* (%)	24 (26)	23 (24)	20 (22)	67 (24)
Sodium-glucose co-transporter 2 inhibitors, *n* (%)	75 (80)	76 (80)	69 (76)	220 (79)
Platelet aggregation inhibitors, *n* (%)	94 (100)	94 (99)	90 (99)	278 (99)
HMG CoA reductase inhibitors, *n* (%)	88 (94)	89 (94)	89 (98)	266 (95)

ACE, angiotensin-converting enzyme; HMG CoA, 3-hydroxy-3-methylglutaryl-coenzyme A.

**Extended Data Table 2:** Key secondary endpoints at 6 and 12 months. Data are shown for the PP population with each CDR132L dose analyzed separately

	CDR132L 5 mg kg^−1^ (*n* = 81)	CDR132L 10 mg kg^−1^ (*n* = 86)	Placebo (*n* = 83)
LVEF, %, absolute change from baseline to month 6 (LOCF)			
*n*	81	86	83
Mean (s.d.)	8.39 (9.04)	8.87 (9.22)	7.00 (8.86)
LSM difference, CDR132L versus placebo (95% CI; 1-sided *P* value)	1.42 (−1.47, 4.30; 0.167)	1.88 (−0.96, 4.73; 0.097)	
LVEF, %, absolute change from baseline to month 12 (LOCF)			
*n*	76	80	78
Mean (s.d.)	8.01 (9.64)	10.55 (10.49)	7.68 (10.35)
LSM difference, CDR132L versus placebo (95% CI; 1-sided *P* value)	0.15 (−3.15, 3.45; 0.464)	2.96 (−0.42, 6.35; 0.043)	
NT-proBNP, pg ml^−1^; logarithmic scale, absolute change from baseline to month 6 (LOCF)			
*n*	81	86	83
Mean (s.d.)	−1.22 (0.88)	−1.01 (0.90)	−1.17 (0.85)
Ratio (CDR132L/placebo) (95% CI; 1-sided *P* value)	0.98 (0.77, 1.25; 0.437)	1.17 (0.92, 1.50; 0.901)	
NT-proBNP, pg ml^−1^; logarithmic scale, absolute change from baseline to month 12 (LOCF)			
*n*	77	83	79
Mean (s.d.)	−1.44 (0.95)	−1.32 (0.89)	−1.22 (0.93)
Ratio (CDR132L/placebo) (95% CI; 1-sided *P* value)	0.83 (0.64, 1.07; 0.073)	0.90 (0.70, 1.16; 0.200)	

CI, confidence interval; LOCF, last observation carried forward; LS, least squares; LVEF, left ventricular ejection fraction; NT-proBNP, N-terminal pro B-type natriuretic peptide; PP, per protocol; SD, standard deviation.

**Extended Data Table 3:** Safety overview for the safety population with data for each CDR132L dose analyzed separately

	CDR132L 5 mg kg^−1^ (*n* = 94)		CDR132L 10 mg kg^−1^ (*n* = 95)		Placebo (*n* = 91)	
	*n* (%)	E (R)	*n* (%)	E (R)	*n* (%)	E (R)
Overview of adverse events						
Number of patients	94 (PYE, 21.61)		95 (PYE, 22.05)		91 (PYE, 21.37)	
TEAEs, OT	47 (50)	116 (537)	51 (54)	120 (544)	40 (44)	81 (379)
Adverse events, IT	62 (66)	217 (1,004)	68 (72)	212 (962)	57 (63)	174 (814)
Serious TEAEs, OT	9 (10)	13 (60)	8 (8)	10 (45)	9 (10)	10 (47)
Serious adverse events	23 (24)	42 (194)	25 (26)	34 (154)	21 (23)	28 (131)
Severe TEAEs, OT	5 (5)	8 (37)	5 (5)	7 (32)	5 (5)	6 (28)
Treatment-related TEAEs, OT	16 (17)	22 (102)	16 (17)	25 (113)	2 (2)	3 (14)
TEAEs leading to discontinuation of study treatment, OT	7 (7)	8 (37)	4 (4)	4 (18)	2 (2)	3 (14)
Adverse events leading to death, IT	0 (0)	0 (0)	1 (1)	1 (5)	5 (6)	6 (28)
Causes of death, IT						
Cardiovascular	0 (0)	−	0 (0)	−	6 (7)	6 (28)
Lung cancer	0 (0)	−	1 (1)	1 (5)	0 (0)	−
TEAEs occurring in ≥5% of patients in any treatment group						
Cardiac disorders	9 (10)	13 (60)	7 (7)	9 (41)	10 (11)	13 (61)
Gastrointestinal disorders	6 (6)	6 (28)	11 (12)	13 (59)	3 (3)	3 (14)
General disorders and administration site conditions	3 (3)	3 (14)	11 (12)	13 (59)	7 (8)	9 (42)
Infections and infestations	18 (19)	19 (88)	11 (12)	13 (59)	9 (10)	9 (42)
*Nasopharyngitis*	5 (5)	5 (23)	4 (4)	4 (18)	3 (3)	3 (14)
Injury, poisoning and procedural complications	3 (3)	3 (14)	5 (5)	7 (32)	2 (2)	2 (9)
Investigations	5 (5)	8 (37)	7 (7)	9 (41)	6 (7)	7 (33)
Metabolism and nutrition disorders	7 (7)	8 (37)	3 (3)	3 (14)	0 (0)	0 (0)
Nervous system disorders	8 (9)	11 (51)	13 (14)	14 (63)	4 (4)	4 (19)
Renal and urinary disorders	5 (5)	6 (28)	2 (2)	2 (9)	6 (7)	6 (28)
Respiratory, thoracic and mediastinal disorders	8 (9)	9 (42)	6 (6)	8 (36)	9 (10)	10 (47)
Skin and subcutaneous tissue disorders	8 (9)	9 (42)	7 (7)	8 (36)	1 (1)	1 (5)
Vascular disorders	6 (6)	6 (28)	6 (6)	6 (27)	5 (5)	5 (23)

PT is shown in italic text. %, proportion of patients with event(s); E, event; IT, in-trial (from randomization to last contact); n, number of patients with event(s); OT, on-treatment (from first dose to last dose plus 30 days); PT, preferred term; PYE, patient-years of exposure; R, events per 100 years of exposure; TEAE, treatment-emergent adverse event (adverse events that first occurred or worsened in severity after the first administration of study treatment and prior to 30 days after the last administration of study treatment).

**Extended Data Table 4:** Full inclusion and exclusion criteria for HF-REVERT

**Inclusion criteria**
• Male or female patients aged 30–80 years (inclusive)
• Body weight ≤120 kg
• Spontaneous acute MI (type I) according to the universal MI definition
• Randomization ≤14 days after index MI diagnosis
• LVEF ≤45% by core lab echocardiography following MI diagnosis
• STEMI or NSTEMI; NSTEMI required evidence of clinically significant myocardial necrosis (troponin T or troponin I ≥5 × ULN at MI diagnosis)
• NT-proBNP ≥125 pg ml^−1^ and <8,000 pg ml^−1^
• Patients with prior MI allowed
• Patients with STEMI/NSTEMI who underwent PCI or coronary angiography (if no indication for stent placement or balloon procedure) for the index event
• Ability to comply with study procedures and provide written informed consent
**Exclusion criteria**
• Heart failure of non-ischemic origin (for example myocarditis, alcoholic cardiomyopathy)
• Prior LVEF <30% within 6 months before the index MI
• NYHA class IV at screening or randomization
• Planned cardiac intervention or surgery during the study period (angiography without intervention permitted)
• Severe valvular heart disease
• Systolic BP <90 mmHg or >180 mmHg, diastolic BP <50 mmHg or >110 mmHg, or HR <50 or >100 bpm at screening/randomization
• Clinically significant arrhythmias, conduction abnormalities, family history of long-QT syndrome, recurrent unexplained syncope, or concerning structural abnormalities based on investigator judgment
• eGFR <30 ml min^−1^ 1.73 m^−2^ or dialysis
• Hepatic insufficiency (Child–Pugh B or C)
• Total bilirubin ≥2 × ULN and ALT ≥3 × ULN
• Known active infection with HIV, hepatitis B or hepatitis C
• Stroke within 6 months prior to screening
• Multiple sclerosis or other diseases affecting the blood–brain barrier
• Platelet count <100,000 μl^−1^
• Bleeding disorders
• Poorly controlled diabetes, based on investigator judgment
• Current treatment for epilepsy
• Relevant psychiatric or physical illness that is unstable or has required recent changes in therapy
• Any clinically significant abnormality on ECG judged by the investigator to pose risk
• Any condition that, in the opinion of the investigator, would interfere with participation or interpretation of study results

Full protocol-specified criteria are presented above. This table accompanies the summary provided in the Methods. ALT, alanine aminotransferase; BP, blood pressure; ECG, electrocardiogram; eGFR, estimated glomerular filtration rate; HIV, human immunodeficiency virus; HR, heart rate; LVEF, left ventricular ejection fraction; MI, myocardial infarction; NSTEMI, non-ST-segment elevation myocardial infarction; NT-proBNP, N-terminal pro B-type natriuretic peptide; NYHA, New York Heart Association; PCI, percutaneous coronary intervention; STEMI, ST-segment elevation myocardial infarction; ULN, upper limit of normal.
