# Supplementary material for: The microRNA inhibitor CDR132L in patients with reduced left ventricular ejection fraction after myocardial infarction: a randomized phase 2 trial
Source: Nat Med. 2026 May 10;32(7):2654–61. doi: 10.1038/s41591-026-04408-4 (PMC13375544; doi:10.1038/s41591-026-04408-4)
Supplement: Supplementary file 1 — Supplementary Tables 1–4, Supplementary Figs. 1–6, Supplementary Note 1 (redacted protocol) and Supplementary Note 2 (redacted statistical analysis plan). [file 41591_2026_4408_MOESM1_ESM.pdf]

nature medicine

Supplementary information https://doi.org/10.1038/s41591-026-04408-4

The microRNA inhibitor CDR132L in patients with reduced left ventricular ejection fraction after myocardial infarction: a randomized phase 2 trial

In the format provided by the authors and unedited

Table of Contents

Supplementary Table 1 2
Supplementary Table 2 5
Supplementary Table 3 9
Supplementary Table 4 14
Supplementary Figure 1 16
Supplementary Figure 2 20
Supplementary Figure 3 24
Supplementary Figure 4 28
Supplementary Figure 5 32
Supplementary Figure 6 36
Supplementary Note 1 (redacted protocol) 40
Supplementary Note 2 (redacted SAP) 118

Supplementary Table 1: Additional baseline patient demographics and characteristics for the mITT population with data for each CDR132L dose analyzed separately.

	**CDR132L 5 mg kg^{-1} (*N* = 94)**	**CDR132L 10 mg kg^{-1} (*N* = 96)**	**Placebo (*N* = 90)**	**All patients (*N* = 280)**
**Age[1], years**				
Mean (SD)	60.8 (10.12)	61.0 (10.11)	61.0 (10.44)	60.9 (10.18)
**Age[1] group, years, *n* (%)**				
<60	41 (44)	42 (44)	40 (44)	123 (44)
≥60	53 (56)	54 (56)	50 (56)	157 (56)
**Race[2], *n* (%)**				
White	90 (96)	91 (95)	88 (98)	269 (96)
Black or African American	1 (1)	0 (0)	2 (2)	3 (1)
Asian	2 (2)	1 (1)	1 (1)	4 (1)
Native Hawaiian or Other Pacific Islander	1 (1)	0 (0)	0 (0)	1 (<1)
Not reported	1 (1)	4 (4)	0 (0)	5 (2)
**Country, *n* (%)**				
Czech Republic	9 (10)	8 (8)	4 (4)	21 (8)
Germany	9 (10)	9 (9)	8 (9)	26 (9)
Greece	1 (1)	0 (0)	2 (2)	3 (1)
Hungary	1 (1)	0 (0)	4 (4)	5 (2)
Netherlands	8 (9)	12 (13)	12 (13)	32 (11)
Poland	37 (39)	27 (28)	26 (29)	90 (32)
Spain	22 (23)	27 (28)	24 (27)	73 (26)
United Kingdom	7 (7)	13 (14)	10 (11)	30 (11)
**Weight at screening, kg**				
Mean (SD)	81.88 (15.35)	79.85 (14.06)	82.73 (13.22)	81.46 (14.25)
Median (min, max)	80.00 (46.5, 119.0)	77.80 (45.0, 111.9)	81.10 (49.0, 118.0)	80.00 (45.0, 119.0)
**BMI at screening, kg m^{-2}**				
Mean (SD)	27.36 (4.25)	26.39 (3.99)	27.78 (4.23)	27.16 (4.18)
Median	27.10 (17.3, 36.7)	25.94 (17.1, 37.4)	26.83 (19.5, 40.0)	26.45 (17.1, 40.0)
**BMI group at screening, kg m^{-2}, *n* (%)**				
<25	32 (34)	36 (38)	23 (26)	91 (33)
≥25	62 (66)	60 (63)	67 (74)	189 (68)
**Baseline NT-proBNP level[3], pg ml^{-1}**				
Mean (SD)	2169.29 (2503.10)	1582.38 (1252.97)	2190.08 (2526.25)	1974.74 (2177.05)
Median (min, max)	1350.00	1286.50	1250.00	1307.00

	CDR132L 5 mg kg^{-1} (*N* = 94)	CDR132L 10 mg kg^{-1} (*N* = 96)	Placebo (*N* = 90)	All patients (*N* = 280)
	(163.0, 17451.0)	(55.0, 5579.0)	(107.0, 15603.0)	(55.0, 17451.0)
**Troponin T (local), ng l^{-1}**				
n	45	48	48	141
Mean (SD)	18835.6 (82895.31)	418945.9 (2546564.24)	4253.4 (6727.34)	150079.2 (1488922.71)
Median (min, max)	2309.0 (12, 542000)	2755.5 (11, 17585000)	1496.0 (17, 30958)	2305.0 (11, 17585000)
**Troponin I (local), ng l^{-1}**				
n	47	47	42	136
Mean (SD)	35820.33 (65772.42)	37956.21 (77673.79)	40847.75 (78313.85)	38111.05 (73461.62)
Median (min, max)	4861.40 (3.00, 335479.00)	11234.00 (10.00, 398398.80)	9701.35 (41.20, 352171.00)	10072.30 (3.00, 398398.80)
**Days between index AMI and randomization[4]**				
Mean (SD)	10.3 (3.08)	9.8 (2.94)	10.9 (2.56)	10.3 (2.90)
**Days between index AMI and troponin measurement[5]**				
n	92	95	90	277
Mean (SD)	5.1 (2.46)	4.9 (2.61)	5.5 (2.69)	5.2 (2.59)
Median (min, max)	4.0 (2, 13)	4.0 (2, 13)	5.0 (1, 12)	4.0 (1, 13)
**Diabetes status, *n* (%)**				
Diabetes	30 (32)	18 (19)	24 (27)	72 (26)
No diabetes	64 (68)	78 (81)	66 (73)	208 (74)
**Chronic HF status, *n* (%)**				
Patients with	9 (10)	7 (7)	10 (11)	26 (9)
Patients without	85 (90)	89 (93)	80 (89)	254 (91)
**eGFR, ml min^{-1} 1.72 m^{-2}, *n* (%)**				
≤45	6 (6)	3 (3)	5 (6)	14 (5)
>45 to ≤60	10 (11)	10 (10)	9 (10)	29 (10)
>60	78 (83)	83 (86)	76 (84)	237 (85)
**Atrial fibrillation status, *n* (%)**				
Patients with	4 (4)	5 (5)	3 (3)	12 (4)
Patients without	90 (96)	91 (95)	87 (97)	268 (96)
**Septum thickness, cm, *n* (%)**				
≤1.2	44 (47)	49 (51)	49 (54)	142 (51)
>1.2	50 (53)	46 (48)	41 (46)	137 (49)

	CDR132L 5 mg kg^{-1} (N = 94)	CDR132L 10 mg kg^{-1} (N = 96)	Placebo (N = 90)	All patients (N = 280)
**LVEF, %[6]**				
Mean (SD)	36.013 (6.80)	35.391 (7.25)	34.937 (5.72)	35.454 (6.63)
**LVESVI, ml m^{-2}**				
Mean (SD)	44.639 (15.06)	44.219 (14.49)	45.356 (12.04)	44.725 (13.91)

[1]Age at time of consent, patients enrolled were aged ≥30 to ≤80 years.

[2]More than one response could be provided and the percentage may total more than 100%.

[3]Baseline NT-proBNP values are based on central laboratory analyses and may differ from local screening values used for eligibility assessment.

[4]Index AMI date to randomization date.

[5]Index AMI date to date of troponin measurement.

[6]One patient had a baseline LVEF >45% due to an initial central echocardiography reading error identified during final QC; the patient was randomized based on the original eligibility confirmation and was retained in the PP and mITT populations. All other randomized patients had an LVEF ≤45%.

AMI, acute myocardial infarction; BMI, body mass index; eGFR, estimated glomerular filtration rate; HF, heart failure; LVEF, left ventricular ejection fraction; LVESVI, left ventricular end systolic volume index; mITT, modified intention-to-treat; NT-proBNP, N-terminal pro B-type natriuretic peptide; PP, per protocol; QC, quality control; SD, standard deviation.

Supplementary Table 2: Baseline patient demographics and characteristics for the PP population with data for each CDR132L dose analyzed separately.

	CDR132L 5 mg kg^{-1} (*N* = 81)	CDR132L 10 mg kg^{-1} (*N* = 86)	Placebo (*N* = 83)	All patients (*N* = 250)
**Age, years[1]**				
Mean (SD)	60.8 (10.41)	60.5 (10.18)	60.7 (10.50)	60.6 (10.32)
Median (min, max)	61.0 (35, 80)	61.0 (37, 79)	61.0 (36, 79)	61.0 (35, 80)
**Age[1] group, years, *n* (%)**				
<60	35 (43)	39 (45)	38 (46)	112 (45)
≥60	46 (57)	47 (55)	45 (54)	138 (55)
**Sex at birth, *n* (%)**				
Male	72 (89)	71 (83)	74 (89)	217 (87)
Female	9 (11)	15 (17)	9 (11)	33 (13)
**Race[2], *n* (%)**				
White	80 (99)	81 (94)	81 (98)	242 (97)
Black/African American	0 (0)	0 (0)	2 (2)	2 (1)
American Indian/Alaska Native	0 (0)	0 (0)	0 (0)	0 (0)
Asian	0 (0)	1 (1)	1 (1)	2 (1)
Native Hawaiian/other Pacific Islander	1 (1)	0 (0)	0 (0)	1 (<1)
Not reported	0 (0)	4 (5)	0 (0)	4 (2)
Missing	0 (0)	0 (0)	0 (0)	0 (0)
**Country, *n* (%)**				
Czech Republic	9 (11)	6 (7)	3 (4)	18 (7)
Germany	6 (7)	7 (8)	7 (8)	20 (8)
Greece	1 (1)	0 (0)	2 (2)	3 (1)
Hungary	1 (1)	0 (0)	4 (5)	5 (2)
Netherlands	7 (9)	10 (12)	13 (16)	30 (12)
Poland	32 (40)	26 (30)	23 (28)	81 (32)
Spain	20 (25)	26 (30)	23 (28)	69 (28)
United Kingdom	5 (6)	11 (13)	8 (10)	24 (10)
**Height at screening, cm**				
Mean (SD)	173.0 (7.98)	173.3 (8.98)	172.7 (9.17)	173.0 (8.70)
Median (min, max)	173.0 (150, 191)	173.0 (146, 195)	173.0 (150, 195)	173.0 (146, 195)
**Weight at screening, kg**				
Mean (SD)	82.27 (15.87)	79.64 (14.21)	82.58 (12.53)	81.47 (14.26)

	CDR132L 5 mg kg^{-1} (*N* = 81)	CDR132L 10 mg kg^{-1} (*N* = 86)	Placebo (*N* = 83)	All patients (*N* = 250)
Median (min, max)	80.50 (46.5, 119.0)	77.80 (45.0, 111.9)	81.00 (49.0, 118.0)	80.00 (45.0, 119.0)
**BMI at screening, kg m^{-2}**				
Mean (SD)	27.43 (4.52)	26.47 (4.02)	27.75 (4.14)	27.20 (4.25)
Median (min, max)	27.40 (17.3, 36.7)	25.98 (17.1, 37.4)	26.90 (19.5, 40.0)	26.66 (17.1, 40.0)
**BMI group at screening, kg m^{-2}, *n* (%)**				
<25	29 (36)	32 (37)	20 (24)	81 (32)
≥25	52 (64)	54 (63)	63 (76)	169 (68)
**Baseline NT-proBNP, pg ml^{-1}**				
Mean (SD)	1961.96 (1779.19)	1552.98 (1260.36)	2127.41 (2510.91)	1876.20 (1922.25)
Median (min, max)	1324.00 (163.0, 8911.0)	1256.00 (55.0, 5579.0)	1214.00 (107.0, 15603.0)	1286.00 (55.0, 15603.0)
**Baseline NT-proBNP, pg ml^{-1}**				
≤1286	39 (48)	44 (51)	43 (52)	126 (50)
>1286	42 (52)	42 (49)	40 (48)	124 (50)
**Days between index AMI and randomization**[3]				
Mean (SD)	10.5 (3.07)	9.9 (2.92)	10.9 (2.53)	10.4 (2.86)
Median (min, max)	11.0 (4, 15)	10.0 (4, 14)	11.0 (5, 14)	11.0 (4, 15)
**Location of MI, *n* (%)**[2]				
Anterior	61 (75)	62 (72)	63 (76)	186 (74)
Posterior	1 (1)	4 (5)	6 (7)	11 (4)
Lateral	9 (11)	17 (20)	14 (17)	40 (16)
Other	20 (25)	21 (24)	15 (18)	56 (22)
**Index AMI, *n* (%)**				
STEMI	67 (83)	73 (85)	69 (83)	209 (84)
NSTEMI	14 (17)	13 (15)	14 (17)	41 (16)
**Number of previous infarctions, *n* (%)**				
0	70 (86)	77 (90)	68 (82)	215 (86)
1	8 (10)	5 (6)	12 (14)	25 (10)
2	1 (1)	2 (2)	3 (4)	6 (2)
≥3	2 (2)	2 (2)	0 (0)	4 (2)
**Killip classification, *n* (%)**				
Class I	62 (77)	69 (80)	63 (76)	194 (78)

	CDR132L 5 mg kg^{-1} (*N* = 81)	CDR132L 10 mg kg^{-1} (*N* = 86)	Placebo (*N* = 83)	All patients (*N* = 250)
Class II	16 (20)	15 (17)	18 (22)	49 (20)
Class III	1 (1)	1 (1)	2 (2)	4 (2)
Class IV	2 (2)	1 (1)	0 (0)	3 (1)
**NYHA class, *n* (%)**				
Class I	30 (37)	40 (47)	28 (34)	98 (39)
Class II	45 (56)	42 (49)	52 (63)	139 (56)
Class III	6 (7)	3 (3)	3 (4)	12 (5)
Class IV	0 (0)	0 (0)	0 (0)	0 (0)
**LVEF, %**				
Mean (SD)	36.15 (6.92)	35.22 (7.51)	35.04 (5.37)	35.46 (6.66)
Median (min, max)	36.68 (19.28, 54.97)	36.48 (13.11, 44.76)	34.65 (21.38, 45.00)	35.79 (13.11, 54.97)
**LVEF, %**				
≤35.79	39 (48)	40 (47)	47 (57)	126 (50)
>35.79	42 (52)	46 (53)	36 (43)	124 (50)
**Age group at randomization, years, *n* (%)**				
<60	36 (44)	38 (44)	38 (46)	112 (45)
≥60	45 (56)	48 (56)	45 (54)	138 (55)
**Location of infarction at randomization, *n* (%)**				
Anterior	57 (70)	62 (72)	63 (76)	182 (73)
Non-anterior	24 (30)	24 (28)	20 (24)	68 (27)
**Troponin T (local), ng l^{-1}**				
n	39	43	46	128
Mean (SD)	21432.4 (88906.27)	459257.9 (2690469.66)	4427.6 (6820.83)	162403.3 (1562445.82)
Median (min, max)	2370.0 (12, 542000)	3190.0 (11, 17585000)	1883.0 (17, 30958)	2364.0 (11, 17585000)
**Troponin I (local), ng l^{-1}**				
n	42	42	37	121
Mean (SD)	36172.74 (68769.86)	39344.59 (81838.13)	45523.92 (82346.98)	40133.16 (77156.48)
Median (min, max)	4729.70 (3.00, 335479.00)	10758.45 (10.00, 398398.80)	12356.90 (41.20, 352171.00)	10282.90 (3.00, 398398.80)
**Days between index AMI and troponin measurement[4]**				
Mean (SD)	5.1 (2.49)	5.0 (2.68)	5.5 (2.67)	5.2 (2.61)

	**CDR132L 5 mg kg^{-1} (*N* = 81)**	**CDR132L 10 mg kg^{-1} (*N* = 86)**	**Placebo (*N* = 83)**	**All patients (*N* = 250)**
Median (min, max)	4.0 (2, 13)	4.0 (2, 13)	5.0 (1, 12)	4.0 (1, 13)
**Diabetes status, *n* (%)**				
Diabetes	27 (33)	14 (16)	18 (22)	59 (24)
No diabetes	54 (67)	72 (84)	65 (78)	191 (76)
**Troponin T (central), ng l^{-1}**				
≤198	42 (52)	45 (52)	38 (46)	125 (50)
>198	39 (48)	41 (48)	45 (54)	125 (50)
**Chronic HF status, *n* (%)**				
Patients with	8 (10)	7 (8)	8 (10)	23 (9)
Patients without	73 (90)	79 (92)	75 (90)	227 (91)
**eGFR, ml min^{-1} 1.72 m^{-2}, *n* (%)**				
≤45	4 (5)	3 (3)	4 (5)	11 (4)
>45 to ≤60	9 (11)	9 (10)	8 10)	26 (10)
>60	68 (84)	74 (86)	71 (86)	213 (85)
**Atrial fibrillation status, *n* (%)**				
Patients with	3 (4)	5 (6)	2 (2)	10 (4)
Patients without	78 (96)	81 (94)	81 (98)	240 (96)
**Septum thickness, cm, *n* (%)**				
≤1.2	38 (47)	47 (55)	43 (52)	128 (51)
>1.2	43 (53)	38 (44)	40 (48)	121 (48)

[1]Age at time of consent, patients enrolled are aged ≥30 to ≤80 years.

[2]More than one response could be provided and the percentage may total more than 100%.

[3]Index AMI date to randomization date.

[4]Index AMI date to date of troponin measurement.

AMI, acute myocardial infarction; BMI, body mass index; eGFR, estimated glomerular filtration rate; HF, heart failure; LVEF, left ventricular ejection fraction; MI, myocardial infarction; NSTEMI, non-ST-segment elevation myocardial infarction; NT-proBNP, N-terminal pro B-type natriuretic peptide; NYHA, New York Heart Association; PP, per protocol; SD, standard deviation; STEMI, ST-segment elevation myocardial infarction.

Supplementary Table 3: Supportive secondary endpoints. Data are shown for the mITT population with CDR132L doses pooled.

	**CDR132L 5 mg kg^{-1} (*N* = 94)**	**CDR132L 10 mg kg^{-1} (*N* = 96)**	**Placebo (*N* = 90)**
**LVEF, %**			
**Baseline**			
n	94	96	90
Mean (SD)	36.01 (6.80)	35.39 (7.25)	34.94 (5.72)
**Month 6 (LOCF)**			
n	94	96	90
Mean (SD)	43.72 (10.30)	43.74 (10.60)	42.14 (11.17)
**Absolute change from baseline**			
n	94	96	90
Mean (SD)	7.70 (8.79)	8.35 (8.96)	7.20 (8.98)
LS mean difference, CDR132L vs placebo (95% CI)	0.556 (-2.142, 3.254)	1.157 (-1.506, 3.820)	
1-sided P value	0.342	0.196	
**Relative change from baseline**			
n	94	96	90
Mean (SD)	23.36 (30.45)	27.22 (45.29)	20.70 (26.41)
LS mean difference, CDR132L vs placebo (95% CI)	3.77 (-4.67, 12.21)	7.89 (-2.89, 18.67)	
1-sided P value	0.190	0.075	
**Month 12 (observed cases)**			
n	83	87	80
Mean (SD)	43.62 (10.10)	45.30 (10.98)	43.30 (11.39)
**Absolute change from baseline**			
n	83	87	80
Mean (SD)	7.24 (9.65)	10.00 (10.31)	7.83 (10.21)
LS mean difference, CDR132L vs placebo (95% CI)	-0.71 (-3.88, 2.46)	2.16 (-1.07, 5.39)	
1-sided P value	0.671	0.094	
**Relative change from baseline**			
n	83	87	80
Mean (SD)	22.75 (33.46)	33.30 (51.73)	22.56 (30.27)
LS mean difference, CDR132L vs placebo (95% CI)	0.56 (-9.15, 10.28)	11.23 (-1.43, 23.88)	
1-sided P value	0.454	0.041	
**NT-proBNP, pg ml^{-1}; logarithmic scale**			

	CDR132L 5 mg kg^{-1} (*N* = 94)	CDR132L 10 mg kg^{-1} (*N* = 96)	Placebo (*N* = 90)
**Baseline**			
n	94	96	90
Mean (SD)	7.27 (0.88)	7.04 (0.91)	7.23 (0.96)
**Month 6 (LOCF)**			
n	94	96	90
Mean (SD)	6.15 (1.13)	6.06 (1.02)	6.12 (1.21)
**Absolute change from baseline**			
n	94	96	90
Mean (SD)	-1.12 (0.91)	-0.97 (0.87)	-1.11 (0.87)
Ratio (CDR132L/placebo) (95% CI)	1.04 (0.82, 1.32)	1.17 (0.93, 1.49)	
1-sided P value	0.620	0.908	
**Relative change from baseline**			
n	94	96	90
Mean (SD)	-15.33 (12.20)	-13.35 (12.90)	-15.33 (11.69)
Ratio (CDR132L/placebo) (95% CI)	2.213 (0.085, 57.678)	12.791 (0.427, 382.772)	
1-sided P value	0.684	0.930	
**Month 12 (observed cases)**			
n	85	89	81
Mean (SD)	5.80 (0.93)	5.75 (1.02)	5.97 (1.16)
**Absolute change from baseline**			
n	85	89	81
Mean (SD)	-1.45 (1.00)	-1.29 (0.87)	-1.22 (0.94)
Ratio (CDR132L/placebo) (95% CI)	0.84 (0.63, 1.08)	0.94 (0.73, 1.20)	
1-sided P value	0.084	0.298	
**Relative change from baseline**			
n	85	89	81
Mean (SD)	-19.49 (12.52)	-17.98 (11.89)	-16.72 (12.92)
Ratio (CDR132L/placebo) (95% CI)	0.15 (0.01, 4.18)	0.43 (0.01, 13.41)	
1-sided P value	0.129	0.313	
**LVESVI, ml m^{-2}**			
**Baseline**			
n	94	96	90
Mean (SD)	44.64 (15.06)	44.2 (14.49)	45.36 (12.04)

	CDR132L 5 mg kg^{-1} (*N* = 94)	CDR132L 10 mg kg^{-1} (*N* = 96)	Placebo (*N* = 90)
**Month 6 (LOCF)**			
n	94	96	90
Mean (SD)	40.27 (14.54)	39.60 (14.94)	42.11 (16.17)
**Absolute change from baseline**			
n	94	96	90
Mean (SD)	-4.36 (11.97)	-4.62 (9.7)	-3.25 (11.17)
LS mean difference, CDR132L vs placebo (95% CI)	-1.34 (-4.72, 2.04)	-1.59 (-4.65, 1.48)	
1-sided P value	0.218	0.154	
**Relative change from baseline**			
n	94	96	90
Mean (SD)	-8.36 (19.84)	-9.82 (19.60)	-7.61 (23.87)
LS mean difference, CDR132L vs placebo (95% CI)	-1.11 (-7.77, 5.54)	-2.14 (-8.60, 4.32)	
1-sided P value	0.371	0.257	
**Month 12 (observed cases)**			
n	83	87	80
Mean (SD)	42.09 (15.68)	38.40 (15.21)	41.37 (15.56)
**Absolute change from baseline**			
n	83	87	80
Mean (SD)	-2.93 (13.81)	-5.84 (12.78)	-3.56 (12.92)
LS mean difference, CDR132L vs placebo (95% CI)	1.20 (-2.83, 5.24)	-2.87 (-6.65, 0.90)	
1-sided P value	0.722	0.067	
**Relative change from baseline**			
n	83	87	80
Mean (SD)	-3.98 (26.20)	-11.34 (25.21)	-7.60 (27.12)
LS mean difference, CDR132L vs placebo (95% CI)	4.644 (-3.833, 13.120)	-4.546 (-12.526, 3.434)	
1-sided P value	0.860	0.131	
**Troponin T, ng l^{-1}**			
**Baseline**			
n	94	96	90
Mean (SD)	665.92 (1212.01)	667.03 (1060.89)	515.00 (1049.19)
**Month 6 (LOCF)**			
n	94	96	90
Mean (SD)	49.21 (168.43)	18.07 (49.39)	19.22 (22.49)

	CDR132L 5 mg kg^{-1} (*N* = 94)	CDR132L 10 mg kg^{-1} (*N* = 96)	Placebo (*N* = 90)
**Absolute change from baseline**			
n	94	96	90
Mean (SD)	-616.71 (1192.96)	-648.96 (1059.82)	-495.78 (1049.75)
LS mean difference, CDR132L vs placebo (95% CI)	29.144 (-7.49, 65.78)	-1.442 (-12.86, 9.98)	
1-sided P value	0.941	0.402	
**Relative change from baseline**			
n	94	96	90
Mean (SD)	-67.93 (101.66)	-82.39 (26.25)	-73.43 (38.54)
LS mean difference, CDR132L vs placebo (95% CI)	6.74 (-15.29, 28.76)	-3.76 (-10.74, 3.22)	
1-sided P value	0.727	0.145	
**Month 12 (observed cases)**			
n	86	90	82
Mean (SD)	14.02 (8.15)	11.52 (4.74)	14.26 (11.96)
**Absolute change from baseline**			
n	86	90	82
Mean (SD)	-597.29 (1202.66)	-669.20 (1089.26)	-532.98 (1092.31)
LS mean difference, CDR132L vs placebo (95% CI)	0.241 (-2.86, 3.34)	-2.30 (-4.95, 0.35)	
1-sided P value	0.561	0.044	
**Relative change from baseline**			
n	86	90	82
Mean (SD)	-78.67 (32.72)	-83.66 (26.10)	-78.75 (35.19)
LS mean difference, CDR132L vs placebo (95% CI)	0.47 (-5.89, 6.82)	-0.59 (-7.15, 5.98)	
1-sided P value	0.558	0.430	
**KCCQ overall summary score**			
**Baseline**			
n	92	95	87
Mean (SD)	73.27 (22.37)	74.37 (19.22)	74.86 (19.35)
**Month 6 (LOCF)**			
n	94	96	90
Mean (SD)	80.96 (20.58)	82.05 (17.61)	86.81 (15.91)
**Absolute change from baseline**			
n	92	95	87
Mean (SD)	7.82 (21.50)	7.64 (19.18)	11.70 (18.35)

	CDR132L 5 mg kg^{-1} (*N* = 94)	CDR132L 10 mg kg^{-1} (*N* = 96)	Placebo (*N* = 90)
LS mean difference, CDR132L vs placebo (95% CI)	-4.30 (-9.29, 0.68)	-3.93 (-8.29, 0.42)	
1-sided P value	0.955	0.962	
**Relative change from baseline**			
n	92	95	87
Mean (SD)	19.60 (45.19)	17.78 (42.55)	22.32 (37.04)
LS mean difference, CDR132L vs placebo (95% CI)	-3.93 (-13.34, 5.48)	-5.42 (-14.02, 3.18)	
1-sided P value	0.794	0.892	
**Month 12 (observed cases)**			
n	87	90	82
Mean (SD)	84.03 (16.37)	82.84 (19.76)	87.96 (18.02)
**Absolute change from baseline**			
n	85	89	80
Mean (SD)	10.26 (20.73)	8.26 (19.39)	12.35 (20.95)
LS mean difference, CDR132L vs placebo (95% CI)	-3.11 (-8.08, 1.86)	-4.14 (-9.29, 1.00)	
1-sided P value	0.891	0.943	
**Relative change from baseline**			
n	85	89	80
Mean (SD)	24.47 (47.29)	17.78 (42.34)	24.05 (42.35)
LS mean difference, CDR132L vs placebo (95% CI)	-2.00 (-11.28, 7.27)	-7.71 (-17.59, 2.18)	
1-sided P value	0.665	0.937	

CI, confidence interval; KCCQ, Kansas City Cardiomyopathy Questionnaire; LOCF, last observation carried forward; LS, least squares; LVEF, left ventricular ejection fraction; LVESVI, left ventricular end-systolic volume index; mITT, modified intent-to-treat; NT-proBNP, N-terminal pro B-type natriuretic peptide; SD, standard deviation.

Supplementary Table 4: Additional safety findings for the safety population with data for each CDR132L dose analyzed separately.

	CDR132L 5 mg kg^{-1} (*N* = 94)		CDR132L 10 mg kg^{-1} (*N* = 95)		Placebo (*N* = 91)	
	***N* (%)**	**E (R)**	***N* (%)**	**E (R)**	***N* (%)**	**E (R)**
**Incidence of TEAEs by maximum severity**						
Patients with any TEAEs	47 (50)	116 (537)	51 (54)	120 (544)	40 (44)	81 (379)
Mild	28 (30)	83 (384)	29 (31)	89 (404)	21 (23)	49 (229)
Moderate	14 (15)	25 (116)	17 (18)	24 (109)	14 (15)	26 (122)
Severe	5 (5)	8 (37)	5 (5)	7 (32)	5 (5)	6 (28)
Missing	0 (0)	0 (0)	0 (0)	0 (0)	0 (0)	0 (0)
**Renal and urinary disorders SOC - most frequently reported PTs**						
Renal failure	1 (1)	1 (5)	1 (1)	1 (5)	2 (2)	2 (9)
Renal impairment	1 (1)	1 (5)	0 (0)	0 (0)	3 (3)	3 (14)
Chronic kidney disease	2 (2)	2 (9)	0 (0)	0 (0)	0 (0)	0 (0)
**Hepatobiliary disorders SOC - most frequently reported PTs**						
Hepatic function abnormal	2 (2)	2 (9)	1 (1)	1 (5)	0 (0)	0 (0)
Hepatic steatosis	0 (0)	0 (0)	1 (1)	1 (5)	0 (0)	0 (0)
**Immune system disorders SOC - most frequently reported PTs**						
Drug hypersensitivity	1 (1)	1 (5)	0 (0)	0 (0)	0 (0)	0 (0)
Hypersensitivity	0 (0)	0 (0)	1 (1)	1 (5)	0 (0)	0 (0)
Seasonal allergy	0 (0)	0 (0)	0 (0)	0 (0)	1 (1)	1 (5)
**Injury, poisoning and procedural complications & skin and subcutaneous tissue disorders SOCs - most frequently reported PTs**						
Dermatitis allergic	1 (1)	1 (5)	3 (3)	4 (18)	0 (0)	0 (0)
Pruritus	1 (1)	1 (5)	2 (2)	2 (9)	1 (1)	1 (5)
Infusion-related reaction	1 (1)	1 (5)	1 (1)	3 (14)	0 (0)	0 (0)
**Thrombocytopenia, heparin-induced thrombocytopenia & platelet count decreased PTs**						
Thrombocytopenia	2 (2)	2 (9)	0 (0)	0 (0)	0 (0)	0 (0)
Heparin-induced thrombocytopenia	0 (0)	0 (0)	0 (0)	0 (0)	0 (0)	0 (0)
Platelet count decreased	0 (0)	0 (0)	1 (1)	1 (5)	0 (0)	0 (0)

Safety population: all randomized patients who received at least 1 dose of study drug (CDR132L or placebo) and have at least 1 post-dose safety assessment. PTs are shown in italic text.

%, proportion of patients with event(s); E, event; IT, in-trial (from randomization to last contact); N, number of patients with event(s); OT, on-treatment (from first dose to last dose plus 30 days); PT, preferred term; PYE, patient-years of exposure; R, events per 100 years of exposure; SOC, system organ class; TEAE, treatment-emergent adverse event (adverse events that first occurred or worsened in severity after the first administration of study treatment and prior to 30 days after the last administration of study treatment).

Supplementary Fig. 1: All subgroups for percentage change from baseline in LVESVI at a) month 6 and b) month 12. Data are shown for the mITT population with each CDR132L dose analyzed separately.

a)

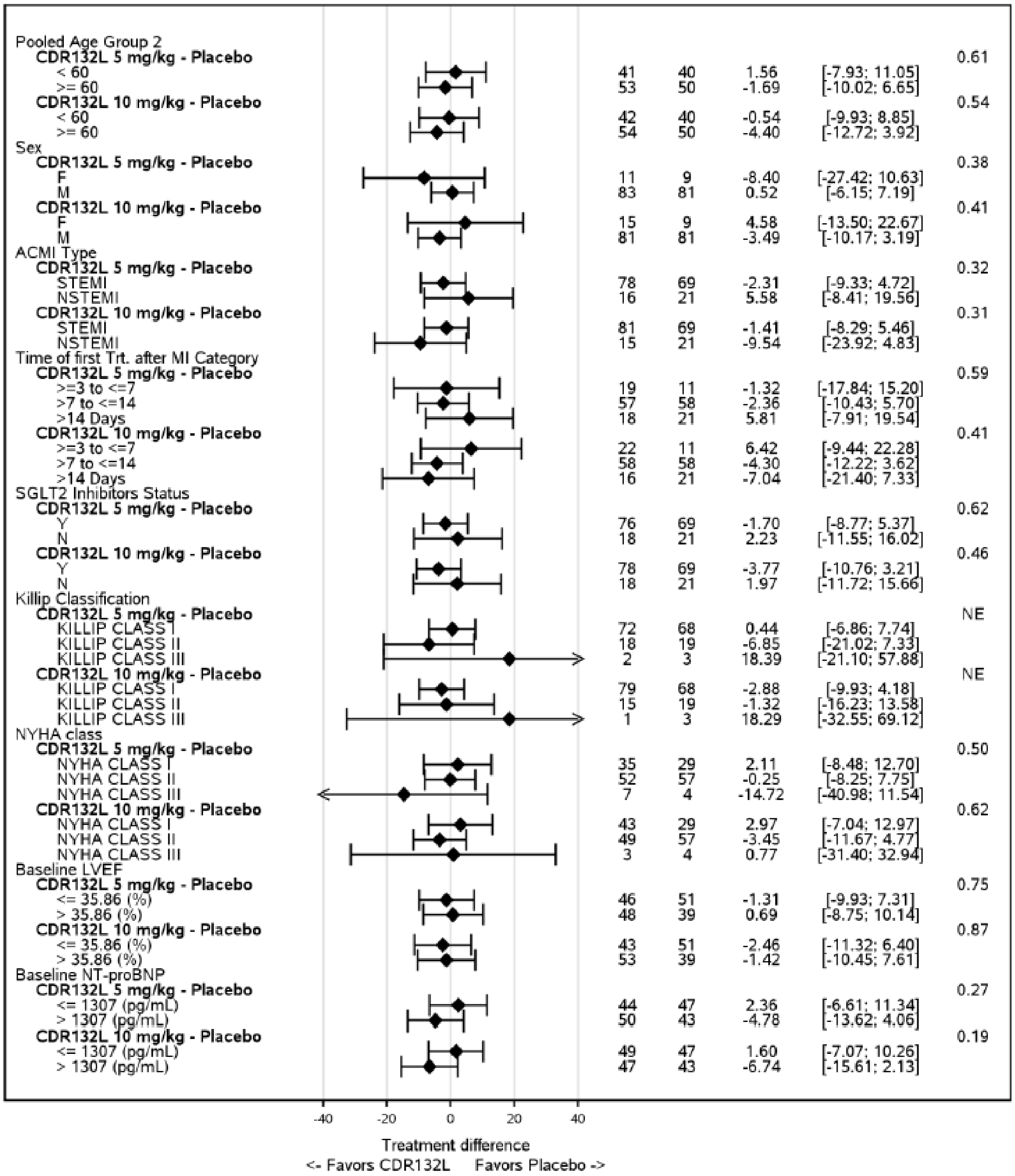

Subgroup	Nact	Npbo	Estimate	[95% CI]	p-value
Pooled Age Group 2					
**CDR132L 5 mg/kg - Placebo**					0.61
< 60	41	40	1.56	[-7.93; 11.05]	
>= 60	53	50	-1.69	[-10.02; 6.65]	
**CDR132L 10 mg/kg - Placebo**					0.54
< 60	42	40	-0.54	[-9.93; 8.85]	
>= 60	54	50	-4.40	[-12.72; 3.92]	
Sex					
**CDR132L 5 mg/kg - Placebo**					0.38
F	11	9	-8.40	[-27.42; 10.63]	
M	83	81	0.52	[-6.15; 7.19]	
**CDR132L 10 mg/kg - Placebo**					0.41
F	15	9	4.58	[-13.50; 22.67]	
M	81	81	-3.49	[-10.17; 3.19]	
ACMI Type					
**CDR132L 5 mg/kg - Placebo**					0.32
STEMI	78	69	-2.31	[-9.33; 4.72]	
NSTEMI	16	21	5.58	[-8.41; 19.56]	
**CDR132L 10 mg/kg - Placebo**					0.31
STEMI	81	69	-1.41	[-8.29; 5.46]	
NSTEMI	15	21	-9.54	[-23.92; 4.83]	
Time of first Trt. after MI Category					
**CDR132L 5 mg/kg - Placebo**					0.59
>=3 to <=7	19	11	-1.32	[-17.84; 15.20]	
>7 to <=14	57	58	-2.36	[-10.43; 5.70]	
>14 Days	18	21	5.81	[-7.91; 19.54]	
**CDR132L 10 mg/kg - Placebo**					0.41
>=3 to <=7	22	11	6.42	[-9.44; 22.28]	
>7 to <=14	58	58	-4.30	[-12.22; 3.62]	
>14 Days	16	21	-7.04	[-21.40; 7.33]	
SGLT2 Inhibitors Status					
**CDR132L 5 mg/kg - Placebo**					0.62
Y	76	69	-1.70	[-8.77; 5.37]	
N	18	21	2.23	[-11.55; 16.02]	
**CDR132L 10 mg/kg - Placebo**					0.46
Y	78	69	-3.77	[-10.76; 3.21]	
N	18	21	1.97	[-11.72; 15.66]	
Killip Classification					
**CDR132L 5 mg/kg - Placebo**					NE
KILLIP CLASS I	72	68	0.44	[-6.86; 7.74]	
KILLIP CLASS II	18	19	-6.85	[-21.02; 7.33]	
KILLIP CLASS III	2	3	18.39	[-21.10; 57.88]	
**CDR132L 10 mg/kg - Placebo**					NE
KILLIP CLASS I	79	68	-2.88	[-9.93; 4.18]	
KILLIP CLASS II	15	19	-1.32	[-16.23; 13.58]	
KILLIP CLASS III	1	3	18.29	[-32.55; 69.12]	
NYHA class					
**CDR132L 5 mg/kg - Placebo**					0.50
NYHA CLASS I	35	29	2.11	[-8.48; 12.70]	
NYHA CLASS II	52	57	-0.25	[-8.25; 7.75]	
NYHA CLASS III	7	4	-14.72	[-40.98; 11.54]	
**CDR132L 10 mg/kg - Placebo**					0.62
NYHA CLASS I	43	29	2.97	[-7.04; 12.97]	
NYHA CLASS II	49	57	-3.45	[-11.67; 4.77]	
NYHA CLASS III	3	4	0.77	[-31.40; 32.94]	
Baseline LVEF					
**CDR132L 5 mg/kg - Placebo**					0.75
<= 35.86 (%)	46	51	-1.31	[-9.93; 7.31]	
> 35.86 (%)	48	39	0.69	[-8.75; 10.14]	
**CDR132L 10 mg/kg - Placebo**					0.87
<= 35.86 (%)	43	51	-2.46	[-11.32; 6.40]	
> 35.86 (%)	53	39	-1.42	[-10.45; 7.61]	
Baseline NT-proBNP					
**CDR132L 5 mg/kg - Placebo**					0.27
<= 1307 (pg/mL)	44	47	2.36	[-6.61; 11.34]	
> 1307 (pg/mL)	50	43	-4.78	[-13.62; 4.06]	
**CDR132L 10 mg/kg - Placebo**					0.19
<= 1307 (pg/mL)	49	47	1.60	[-7.07; 10.26]	
> 1307 (pg/mL)	47	43	-6.74	[-15.61; 2.13]	

Subgroup	Nact	Npbo	Estimate	[95% CI]	p-value
Location of infarction at Randomization					
**CDR132L 5 mg/kg - Placebo**					0.74
Anterior Infarction	67	64	-1.24	[-8.80; 6.31]	
Non-Anterior Infarction	27	26	1.06	[-10.61; 12.73]	
**CDR132L 10 mg/kg - Placebo**					0.88
Anterior Infarction	68	64	-2.01	[-9.40; 5.39]	
Non-Anterior Infarction	28	26	-3.06	[-14.75; 8.63]	
Diabetes Status					
**CDR132L 5 mg/kg - Placebo**					0.29
Diabetes	30	24	4.66	[-7.07; 16.39]	
Non-Diabetes	64	66	-2.70	[-10.21; 4.80]	
**CDR132L 10 mg/kg - Placebo**					0.66
Diabetes	18	24	0.13	[-13.30; 13.57]	
Non-Diabetes	78	66	-3.21	[-10.28; 3.86]	
Baseline Troponin T					
**CDR132L 5 mg/kg - Placebo**					0.22
<= 208.5 (ng/L)	46	46	2.99	[-5.75; 11.73]	
> 208.5 (ng/L)	48	44	-4.69	[-13.50; 4.12]	
**CDR132L 10 mg/kg - Placebo**					0.31
<= 208.5 (ng/L)	48	46	-5.47	[-14.12; 3.18]	
> 208.5 (ng/L)	48	44	0.92	[-7.79; 9.63]	
Chronic HF status					
**CDR132L 5 mg/kg - Placebo**					0.82
Patients with	9	10	-2.70	[-22.38; 16.97]	
Patients without	85	80	-0.38	[-7.06; 6.30]	
**CDR132L 10 mg/kg - Placebo**					0.31
Patients with	7	10	-13.29	[-35.36; 8.78]	
Patients without	89	80	-1.48	[-8.01; 5.04]	
eGFR Baseline Category					
**CDR132L 5 mg/kg - Placebo**					0.03
<=45	6	5	23.70	[-1.80; 49.20]	
>45, <=60	10	9	-18.93	[-38.42; 0.57]	
>60	78	76	-0.21	[-7.08; 6.65]	
**CDR132L 10 mg/kg - Placebo**					0.07
<=45	3	5	7.18	[-24.14; 38.50]	
>45, <=60	10	9	-23.13	[-42.49; -3.76]	
>60	83	76	-0.38	[-7.05; 6.29]	
Atrial fibrillation status					
**CDR132L 5 mg/kg - Placebo**					0.61
Patients with	4	3	7.39	[-25.20; 39.99]	
Patients without	90	87	-1.10	[-7.52; 5.31]	
**CDR132L 10 mg/kg - Placebo**					0.43
Patients with	5	3	-15.20	[-47.80; 17.40]	
Patients without	91	87	-2.08	[-8.40; 4.24]	
Baseline septum thickness					
**CDR132L 5 mg/kg - Placebo**					0.78
<= 1.2 (cm)	44	49	-1.76	[-10.67; 7.15]	
> 1.2 (cm)	50	41	0.04	[-8.98; 9.05]	
**CDR132L 10 mg/kg - Placebo**					0.65
<= 1.2 (cm)	49	49	-1.18	[-9.90; 7.54]	
> 1.2 (cm)	46	41	-4.12	[-13.36; 5.13]	
Baseline LVESVi					
**CDR132L 5 mg/kg - Placebo**					0.88
<= 42.775	49	41	-0.91	[-10.17; 8.35]	
> 42.775	45	49	0.02	[-8.80; 8.85]	
**CDR132L 10 mg/kg - Placebo**					0.88
<= 42.775	50	41	-1.75	[-10.82; 7.31]	
> 42.775	46	49	-2.71	[-11.64; 6.23]	

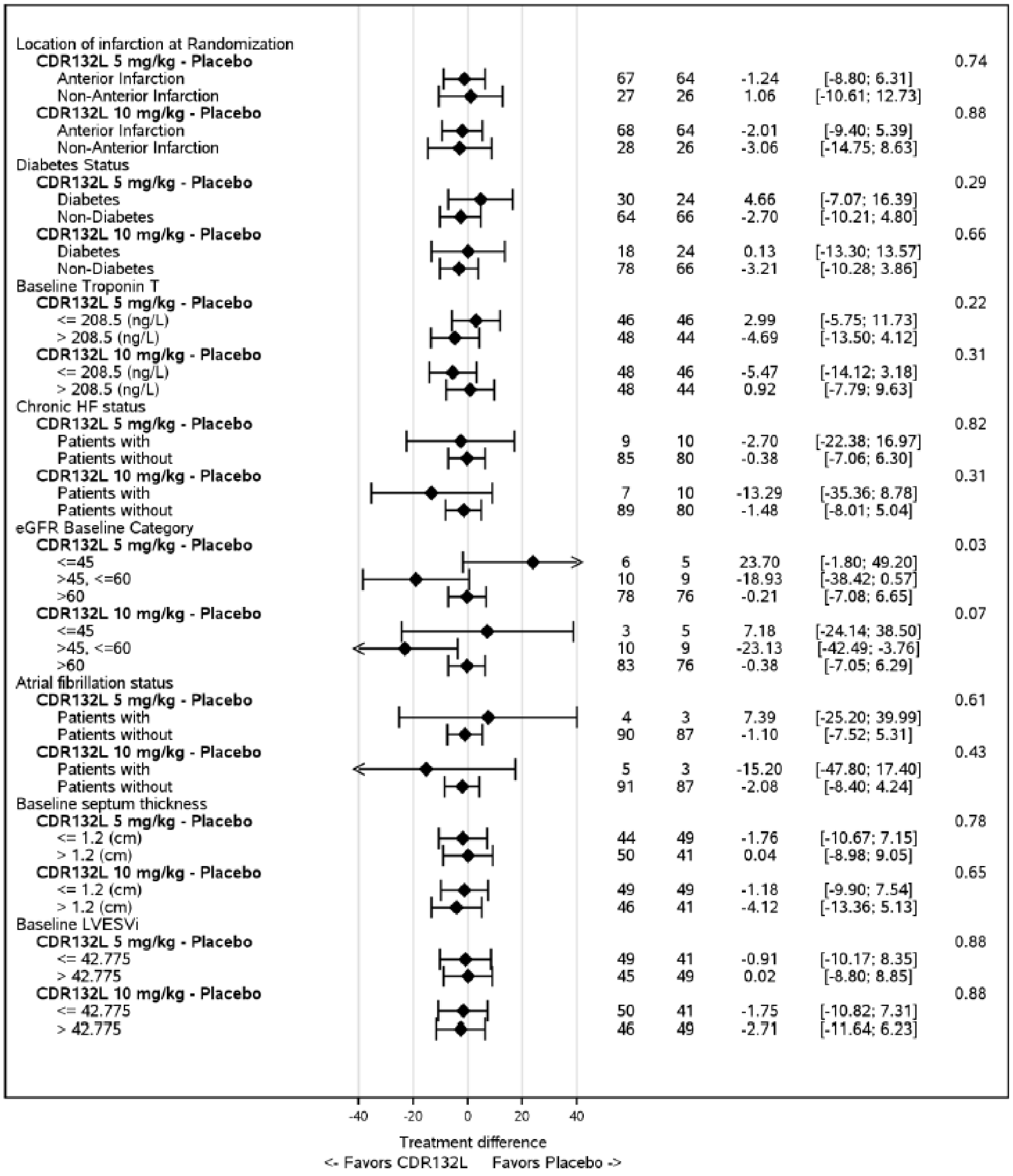

b)

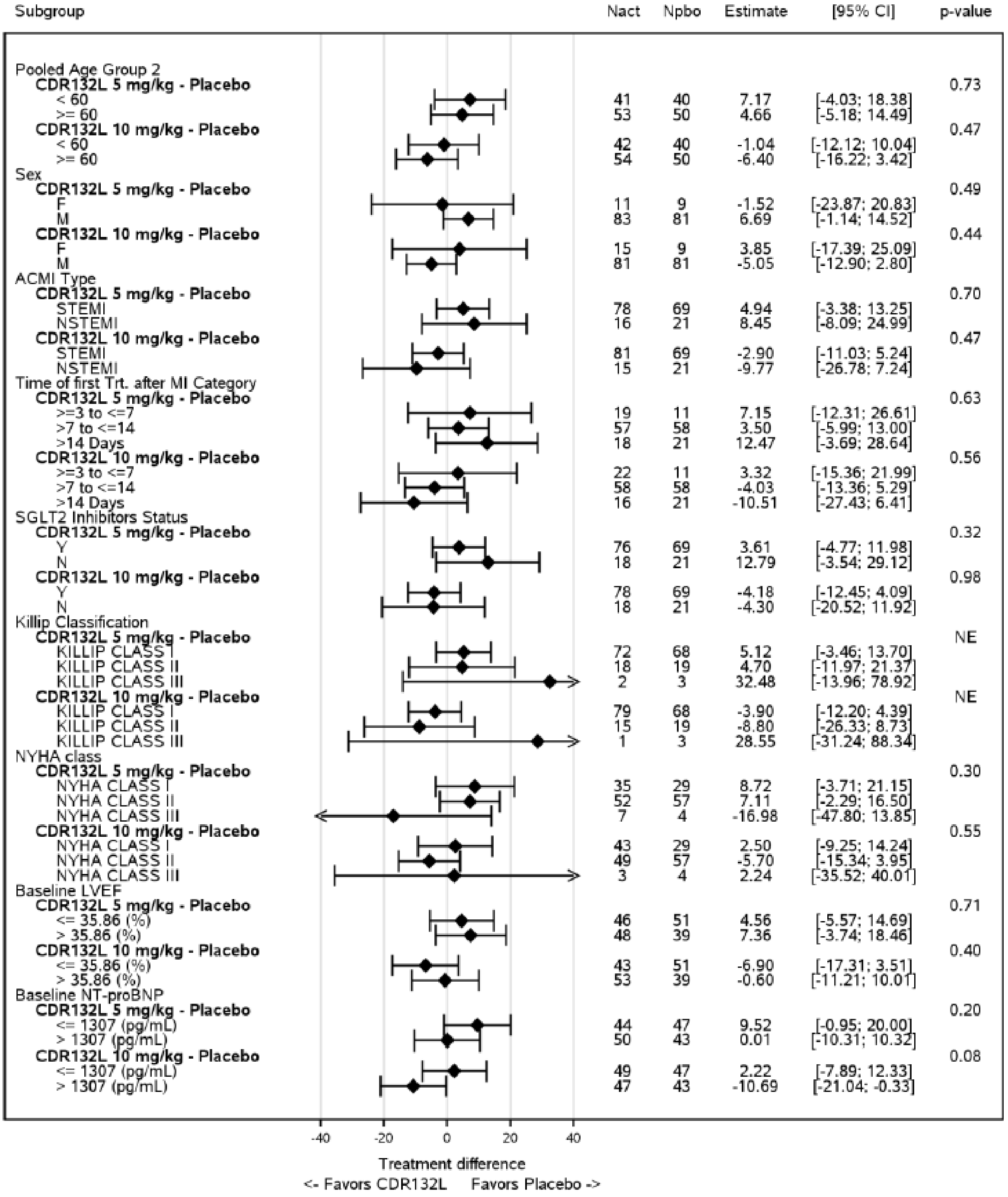
Subgroup
Nact
Npbo
Estimate
[95% CI]
p-value
Pooled Age Group 2
CDR132L 5 mg/kg - Placebo 0.73
< 60 41 40 7.17 [-4.03; 18.38]
>= 60 53 50 4.66 [-5.18; 14.49]
CDR132L 10 mg/kg - Placebo 0.47
< 60 42 40 -1.04 [-12.12; 10.04]
>= 60 54 50 -6.40 [-16.22; 3.42]
Sex
CDR132L 5 mg/kg - Placebo 0.49
F 11 9 -1.52 [-23.87; 20.83]
M 83 81 6.69 [-1.14; 14.52]
CDR132L 10 mg/kg - Placebo 0.44
F 15 9 3.85 [-17.39; 25.09]
M 81 81 -5.05 [-12.90; 2.80]
ACMI Type
CDR132L 5 mg/kg - Placebo 0.70
STEMI 78 69 4.94 [-3.38; 13.25]
NSTEMI 16 21 8.45 [-8.09; 24.99]
CDR132L 10 mg/kg - Placebo 0.47
STEMI 81 69 -2.90 [-11.03; 5.24]
NSTEMI 15 21 -9.77 [-26.78; 7.24]
Time of first Trt. after MI Category
CDR132L 5 mg/kg - Placebo 0.63
>=3 to <=7 19 11 7.15 [-12.31; 26.61]
>7 to <=14 57 58 3.50 [-5.99; 13.00]
>14 Days 18 21 12.47 [-3.69; 28.64]
CDR132L 10 mg/kg - Placebo 0.56
>=3 to <=7 22 11 3.32 [-15.36; 21.99]
>7 to <=14 58 58 -4.03 [-13.36; 5.29]
>14 Days 16 21 -10.51 [-27.43; 6.41]
SGLT2 Inhibitors Status
CDR132L 5 mg/kg - Placebo 0.32
Y 76 69 3.61 [-4.77; 11.98]
N 18 21 12.79 [-3.54; 29.12]
CDR132L 10 mg/kg - Placebo 0.98
Y 78 69 -4.18 [-12.45; 4.09]
N 18 21 -4.30 [-20.52; 11.92]
Killip Classification
CDR132L 5 mg/kg - Placebo NE
KILLIP CLASS I 72 68 5.12 [-3.46; 13.70]
KILLIP CLASS II 18 19 4.70 [-11.97; 21.37]
KILLIP CLASS III 2 3 32.48 [-13.96; 78.92]
CDR132L 10 mg/kg - Placebo NE
KILLIP CLASS I 79 68 -3.90 [-12.20; 4.39]
KILLIP CLASS II 15 19 -8.80 [-26.33; 8.73]
KILLIP CLASS III 1 3 28.55 [-31.24; 88.34]
NYHA class
CDR132L 5 mg/kg - Placebo 0.30
NYHA CLASS I 35 29 8.72 [-3.71; 21.15]
NYHA CLASS II 52 57 7.11 [-2.29; 16.50]
NYHA CLASS III 7 4 -16.98 [-47.80; 13.85]
CDR132L 10 mg/kg - Placebo 0.55
NYHA CLASS I 43 29 2.50 [-9.25; 14.24]
NYHA CLASS II 49 57 -5.70 [-15.34; 3.95]
NYHA CLASS III 3 4 2.24 [-35.52; 40.01]
Baseline LVEF
CDR132L 5 mg/kg - Placebo 0.71
<= 35.86 (%) 46 51 4.56 [-5.57; 14.69]
> 35.86 (%) 48 39 7.36 [-3.74; 18.46]
CDR132L 10 mg/kg - Placebo 0.40
<= 35.86 (%) 43 51 -6.90 [-17.31; 3.51]
> 35.86 (%) 53 39 -0.60 [-11.21; 10.01]
Baseline NT-proBNP
CDR132L 5 mg/kg - Placebo 0.20
<= 1307 (pg/mL) 44 47 9.52 [-0.95; 20.00]
> 1307 (pg/mL) 50 43 0.01 [-10.31; 10.32]
CDR132L 10 mg/kg - Placebo 0.08
<= 1307 (pg/mL) 49 47 2.22 [-7.89; 12.33]
> 1307 (pg/mL) 47 43 -10.69 [-21.04; -0.33]
-40
-20
0
20
40
Treatment difference
<- Favors CDR132L
Favors Placebo ->

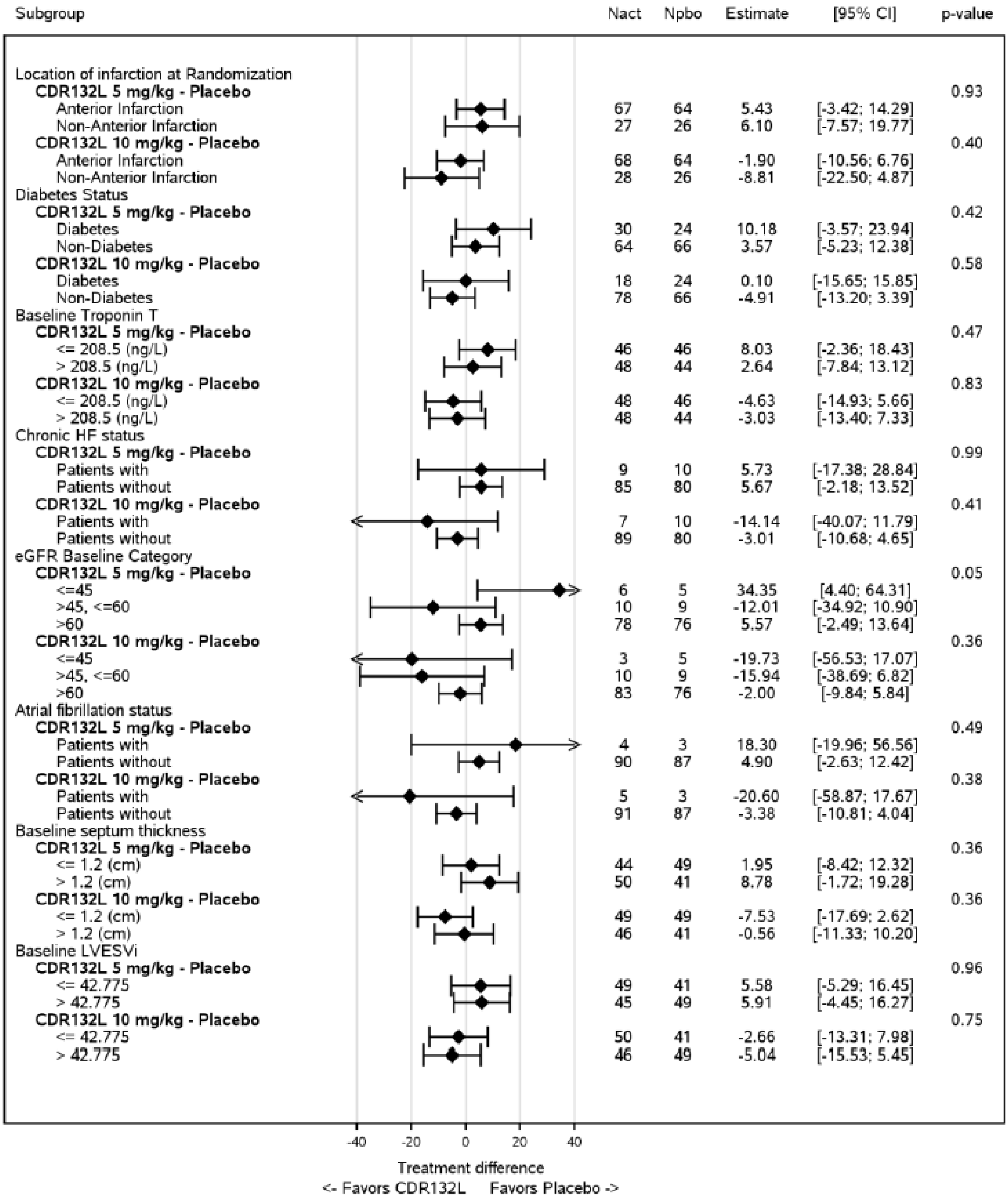

Triangles represent estimates that fall outside the x axis range.
The ANCOVA model includes treatment and center group, stratification factors of age group (<60 years, ≥60 years) and location of infarction (anterior, non-anterior), baseline LVESVI and treatment-by-subgroup interaction. P values are 2-sided. The mITT population includes all randomized patients who received at least 1 dose of study drug (CDR132L or placebo). Missing post-baseline data were imputed using the last observation (including baseline) carried forward approach. All comparisons shown are versus placebo. ACMI, acute myocardial infarction; ANCOVA, analysis of covariance; Ca, category; CI, confidence interval; eGFR, estimated glomerular filtration rate; HF, heart failure; LVEF, left ventricular ejection fraction; LVESVI, left ventricular end-systolic volume index; MI, myocardial infarction; mITT, modified intention-to-treat; Nact, number of patients receiving active treatment; NE, not estimable; Npbo, number of patients receiving placebo; NSTEMI, non-ST-segment elevation myocardial infarction; NT-proBNP, N-terminal pro B-type natriuretic peptide; NYHA, New York Heart Association; SGLT2, sodium-glucose co-transporter 2; STEMI, ST-segment elevation myocardial infarction; Trt, treatment.

Supplementary Fig. 2: All subgroups for change from baseline in LVEF at a) month 6 and b) month 12. Data are shown for the mITT population with each CDR132L dose analyzed separately.

a)

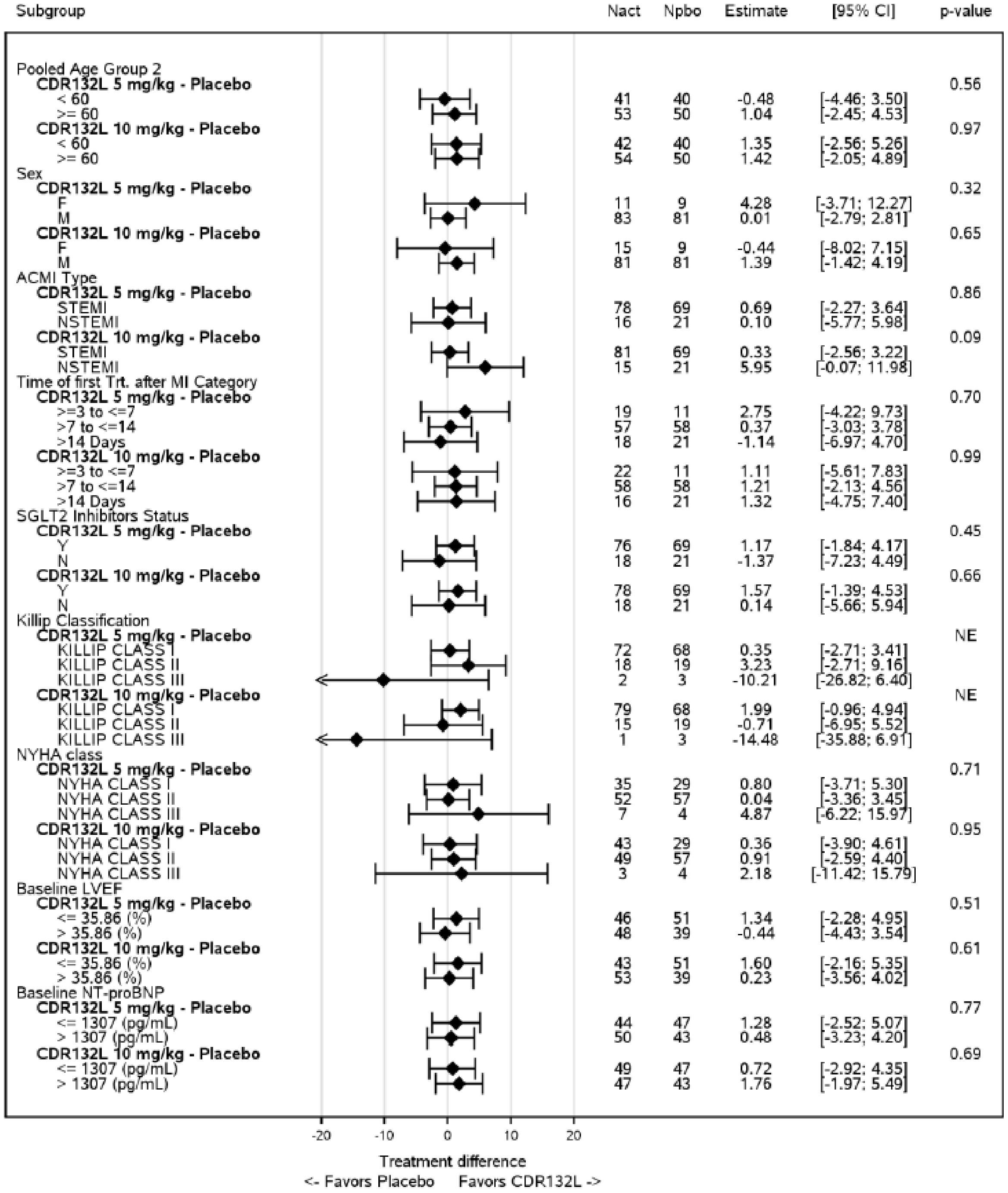

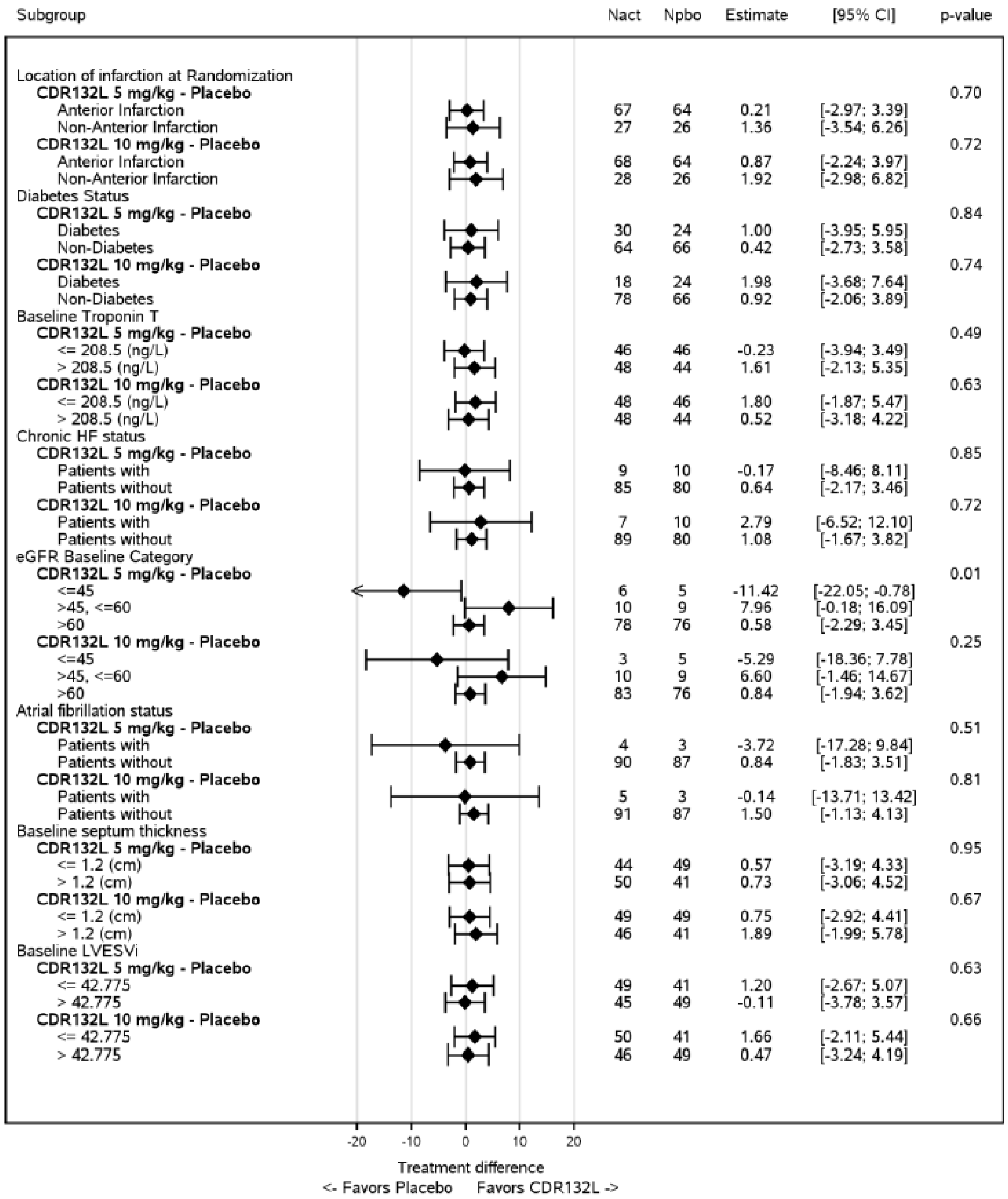
Subgroup Nact Npbo Estimate [95% CI] p-value
Location of infarction at Randomization
CDR132L 5 mg/kg - Placebo 0.70
Anterior Infarction 67 64 0.21 [-2.97; 3.39]
Non-Anterior Infarction 27 26 1.36 [-3.54; 6.26]
CDR132L 10 mg/kg - Placebo 0.72
Anterior Infarction 68 64 0.87 [-2.24; 3.97]
Non-Anterior Infarction 28 26 1.92 [-2.98; 6.82]
Diabetes Status
CDR132L 5 mg/kg - Placebo 0.84
Diabetes 30 24 1.00 [-3.95; 5.95]
Non-Diabetes 64 66 0.42 [-2.73; 3.58]
CDR132L 10 mg/kg - Placebo 0.74
Diabetes 18 24 1.98 [-3.68; 7.64]
Non-Diabetes 78 66 0.92 [-2.06; 3.89]
Baseline Troponin T
CDR132L 5 mg/kg - Placebo 0.49
<= 208.5 (ng/L) 46 46 -0.23 [-3.94; 3.49]
> 208.5 (ng/L) 48 44 1.61 [-2.13; 5.35]
CDR132L 10 mg/kg - Placebo 0.63
<= 208.5 (ng/L) 48 46 1.80 [-1.87; 5.47]
> 208.5 (ng/L) 48 44 0.52 [-3.18; 4.22]
Chronic HF status
CDR132L 5 mg/kg - Placebo 0.85
Patients with 9 10 -0.17 [-8.46; 8.11]
Patients without 85 80 0.64 [-2.17; 3.46]
CDR132L 10 mg/kg - Placebo 0.72
Patients with 7 10 2.79 [-6.52; 12.10]
Patients without 89 80 1.08 [-1.67; 3.82]
eGFR Baseline Category
CDR132L 5 mg/kg - Placebo 0.01
<=45 6 5 -11.42 [-22.05; -0.78]
>45, <=60 10 9 7.96 [-0.18; 16.09]
>60 78 76 0.58 [-2.29; 3.45]
CDR132L 10 mg/kg - Placebo 0.25
<=45 3 5 -5.29 [-18.36; 7.78]
>45, <=60 10 9 6.60 [-1.46; 14.67]
>60 83 76 0.84 [-1.94; 3.62]
Atrial fibrillation status
CDR132L 5 mg/kg - Placebo 0.51
Patients with 4 3 -3.72 [-17.28; 9.84]
Patients without 90 87 0.84 [-1.83; 3.51]
CDR132L 10 mg/kg - Placebo 0.81
Patients with 5 3 -0.14 [-13.71; 13.42]
Patients without 91 87 1.50 [-1.13; 4.13]
Baseline septum thickness
CDR132L 5 mg/kg - Placebo 0.95
<= 1.2 (cm) 44 49 0.57 [-3.19; 4.33]
> 1.2 (cm) 50 41 0.73 [-3.06; 4.52]
CDR132L 10 mg/kg - Placebo 0.67
<= 1.2 (cm) 49 49 0.75 [-2.92; 4.41]
> 1.2 (cm) 46 41 1.89 [-1.99; 5.78]
Baseline LVESVi
CDR132L 5 mg/kg - Placebo 0.63
<= 42.775 49 41 1.20 [-2.67; 5.07]
> 42.775 45 49 -0.11 [-3.78; 3.57]
CDR132L 10 mg/kg - Placebo 0.66
<= 42.775 50 41 1.66 [-2.11; 5.44]
> 42.775 46 49 0.47 [-3.24; 4.19]
-20 -10 0 10 20
Treatment difference
<- Favors Placebo Favors CDR132L ->

b)

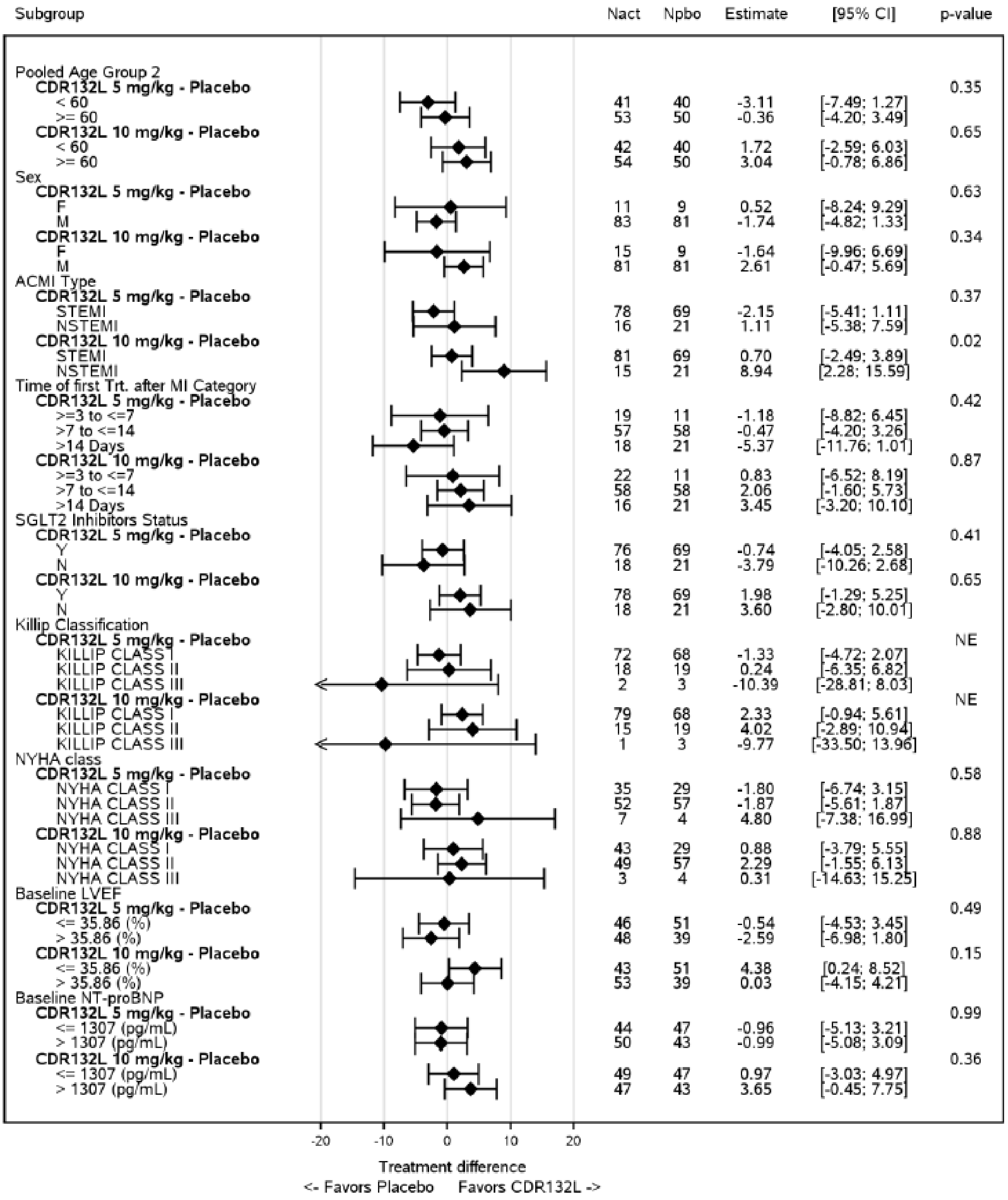
Subgroup
Nact
Npbo
Estimate
[95% CI]
p-value
Pooled Age Group 2
CDR132L 5 mg/kg - Placebo 0.35
< 60 41 40 -3.11 [-7.49; 1.27]
>= 60 53 50 -0.36 [-4.20; 3.49]
CDR132L 10 mg/kg - Placebo 0.65
< 60 42 40 1.72 [-2.59; 6.03]
>= 60 54 50 3.04 [-0.78; 6.86]
Sex
CDR132L 5 mg/kg - Placebo 0.63
F 11 9 0.52 [-8.24; 9.29]
M 83 81 -1.74 [-4.82; 1.33]
CDR132L 10 mg/kg - Placebo 0.34
F 15 9 -1.64 [-9.96; 6.69]
M 81 81 2.61 [-0.47; 5.69]
ACMI Type
CDR132L 5 mg/kg - Placebo 0.37
STEMI 78 69 -2.15 [-5.41; 1.11]
NSTEMI 16 21 1.11 [-5.38; 7.59]
CDR132L 10 mg/kg - Placebo 0.02
STEMI 81 69 0.70 [-2.49; 3.89]
NSTEMI 15 21 8.94 [2.28; 15.59]
Time of first Trt. after MI Category
CDR132L 5 mg/kg - Placebo 0.42
>=3 to <=7 19 11 -1.18 [-8.82; 6.45]
>7 to <=14 57 58 -0.47 [-4.20; 3.26]
>14 Days 18 21 -5.37 [-11.76; 1.01]
CDR132L 10 mg/kg - Placebo 0.87
>=3 to <=7 22 11 0.83 [-6.52; 8.19]
>7 to <=14 58 58 2.06 [-1.60; 5.73]
>14 Days 16 21 3.45 [-3.20; 10.10]
SGLT2 Inhibitors Status
CDR132L 5 mg/kg - Placebo 0.41
Y 76 69 -0.74 [-4.05; 2.58]
N 18 21 -3.79 [-10.26; 2.68]
CDR132L 10 mg/kg - Placebo 0.65
Y 78 69 1.98 [-1.29; 5.25]
N 18 21 3.60 [-2.80; 10.01]
Killip Classification
CDR132L 5 mg/kg - Placebo NE
KILLIP CLASS I 72 68 -1.33 [-4.72; 2.07]
KILLIP CLASS II 18 19 0.24 [-6.35; 6.82]
KILLIP CLASS III 2 3 -10.39 [-28.81; 8.03]
CDR132L 10 mg/kg - Placebo NE
KILLIP CLASS I 79 68 2.33 [-0.94; 5.61]
KILLIP CLASS II 15 19 4.02 [-2.89; 10.94]
KILLIP CLASS III 1 3 -9.77 [-33.50; 13.96]
NYHA class
CDR132L 5 mg/kg - Placebo 0.58
NYHA CLASS I 35 29 -1.80 [-6.74; 3.15]
NYHA CLASS II 52 57 -1.87 [-5.61; 1.87]
NYHA CLASS III 7 4 4.80 [-7.38; 16.99]
CDR132L 10 mg/kg - Placebo 0.88
NYHA CLASS I 43 29 0.88 [-3.79; 5.55]
NYHA CLASS II 49 57 2.29 [-1.55; 6.13]
NYHA CLASS III 3 4 0.31 [-14.63; 15.25]
Baseline LVEF
CDR132L 5 mg/kg - Placebo 0.49
<= 35.86 (%) 46 51 -0.54 [-4.53; 3.45]
> 35.86 (%) 48 39 -2.59 [-6.98; 1.80]
CDR132L 10 mg/kg - Placebo 0.15
<= 35.86 (%) 43 51 4.38 [0.24; 8.52]
> 35.86 (%) 53 39 0.03 [-4.15; 4.21]
Baseline NT-proBNP
CDR132L 5 mg/kg - Placebo 0.99
<= 1307 (pg/mL) 44 47 -0.96 [-5.13; 3.21]
> 1307 (pg/mL) 50 43 -0.99 [-5.08; 3.09]
CDR132L 10 mg/kg - Placebo 0.36
<= 1307 (pg/mL) 49 47 0.97 [-3.03; 4.97]
> 1307 (pg/mL) 47 43 3.65 [-0.45; 7.75]
-20
-10
0
10
20
Treatment difference
<- Favors Placebo
Favors CDR132L ->

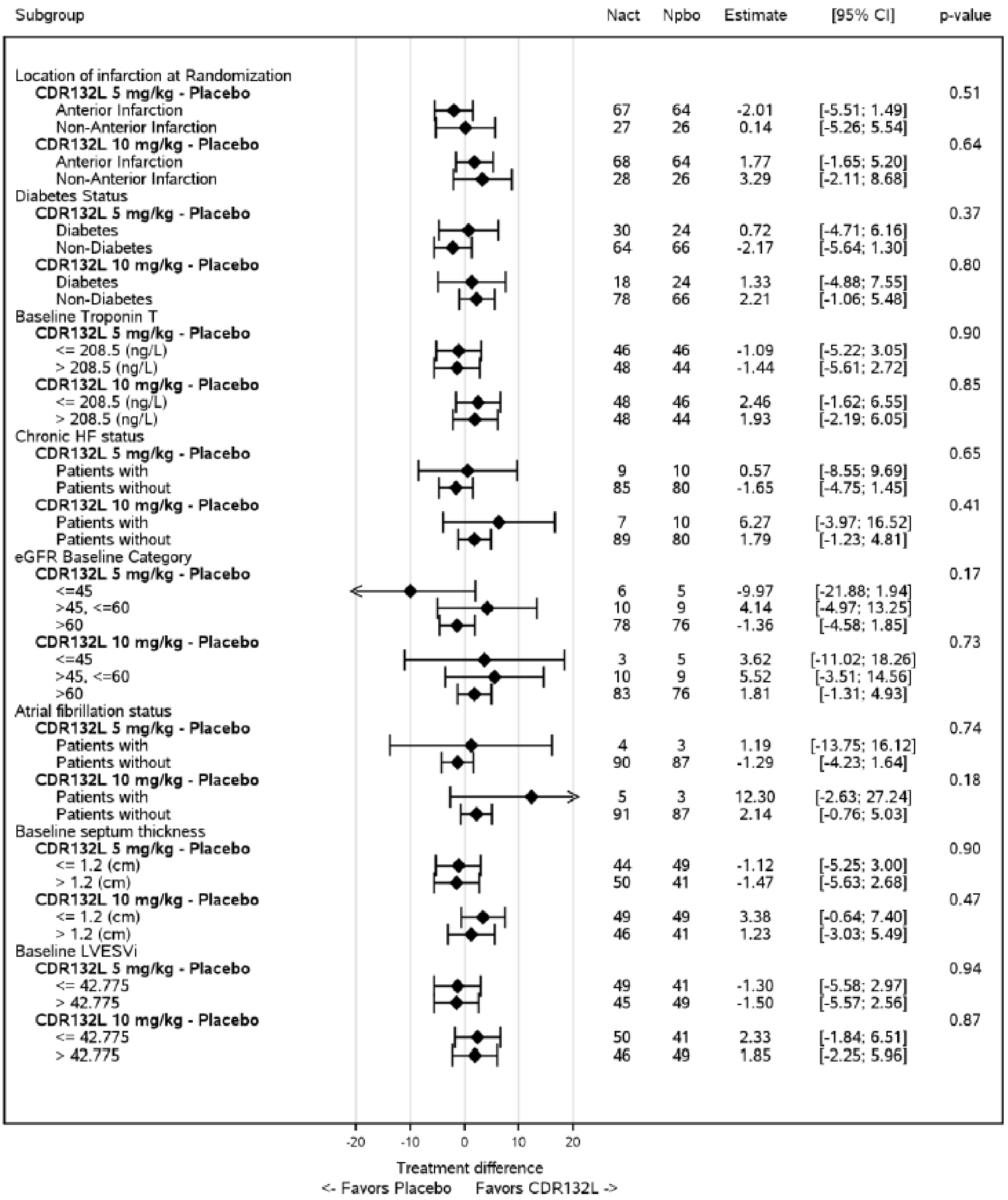

Triangles represent estimates that fall outside the x axis range.
The ANCOVA model includes treatment and center group, stratification factors of age group (<60 years, ≥60 years) and location of infarction (anterior, non-anterior), baseline LVEF and treatment-by-subgroup interaction. P values are 2 sided. The mITT population includes all randomized patients who received at least 1 dose of study drug (CDR132L or placebo). Missing post-baseline data were imputed using the last observation (including baseline) carried forward approach. All comparisons shown are versus placebo.
ACMI, acute myocardial infarction; ANCOVA, analysis of covariance; Ca, category; CI, confidence interval; eGFR, estimate glomerular filtration rate; HF, heart failure; LVEF, left ventricular ejection fraction; LVESVI, left ventricular end-systolic volume index; MI, myocardial infarction; mITT, modified intention-to-treat; Nact, number of patients receiving active treatment; NE, not estimable; Npbo, number of patients receiving placebo; NSTEMI, non-ST-segment elevation myocardial infarction; NT-proBNP, N-terminal pro B-type natriuretic peptide; NYHA, New York Heart Association; PP, per protocol; Rand, randomization; SGLT2, sodium-glucose co-transporter 2; STEMI, ST-segment elevation myocardial infarction; Trt, treatment.

Supplementary Fig. 3: All subgroups for percentage change from baseline in LVESVI at a) month 6 and b) month 12. Data are shown for the PP population with each CDR132L dose analyzed separately.

a)

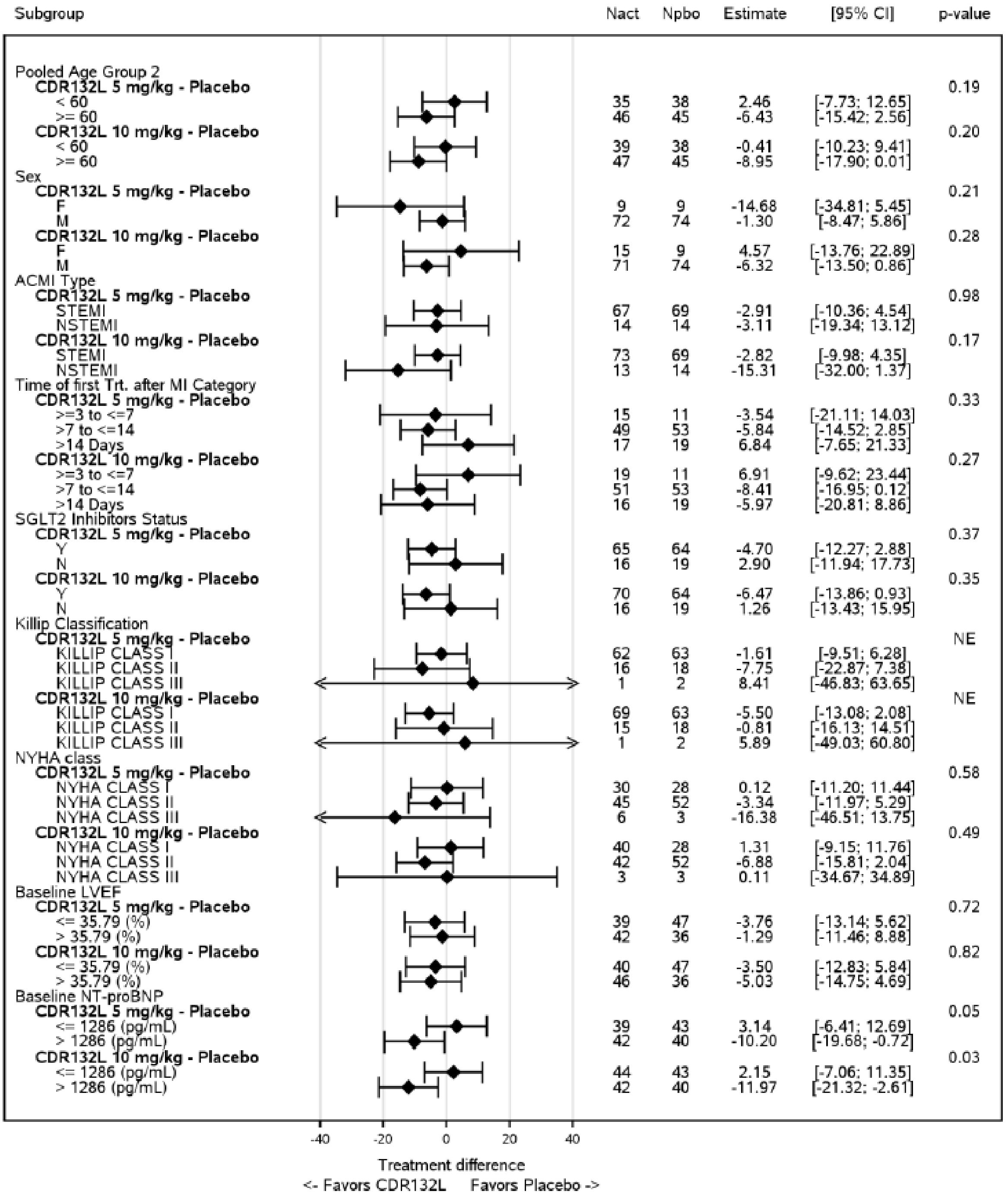

Subgroup	Nact	Npbo	Estimate	[95% CI]	p-value
Location of infarction at Randomization					
**CDR132L 5 mg/kg - Placebo**					0.90
Anterior Infarction	57	63	-2.56	[-10.59; 5.47]	
Non-Anterior Infarction	24	20	-3.49	[-16.64; 9.65]	
**CDR132L 10 mg/kg - Placebo**					0.70
Anterior Infarction	62	63	-3.86	[-11.56; 3.84]	
Non-Anterior Infarction	24	20	-6.77	[-20.06; 6.53]	
Diabetes Status					
**CDR132L 5 mg/kg - Placebo**					0.78
Diabetes	27	18	-1.29	[-14.63; 12.06]	
Non-Diabetes	54	65	-3.43	[-11.47; 4.61]	
**CDR132L 10 mg/kg - Placebo**					0.89
Diabetes	14	18	-5.60	[-21.28; 10.08]	
Non-Diabetes	72	65	-4.46	[-11.81; 2.89]	
Baseline Troponin T					
**CDR132L 5 mg/kg - Placebo**					0.27
<= 198 (ng/L)	42	38	1.03	[-8.49; 10.56]	
> 198 (ng/L)	39	45	-6.43	[-15.75; 2.88]	
**CDR132L 10 mg/kg - Placebo**					0.27
<= 198 (ng/L)	45	38	-7.50	[-16.84; 1.84]	
> 198 (ng/L)	41	45	-0.16	[-9.33; 9.02]	
Chronic HF status					
**CDR132L 5 mg/kg - Placebo**					0.80
Patients with	8	8	-5.48	[-27.33; 16.38]	
Patients without	73	75	-2.53	[-9.75; 4.68]	
**CDR132L 10 mg/kg - Placebo**					0.36
Patients with	7	8	-15.04	[-38.72; 8.65]	
Patients without	79	75	-3.74	[-10.70; 3.21]	
eGFR Baseline Category					
**CDR132L 5 mg/kg - Placebo**					0.23
<=45	4	4	7.47	[-23.16; 38.10]	
>45, <=60	9	8	-19.17	[-40.30; 1.96]	
>60	68	71	-1.42	[-8.88; 6.03]	
**CDR132L 10 mg/kg - Placebo**					0.14
<=45	3	4	2.20	[-31.64; 36.05]	
>45, <=60	9	8	-24.29	[-45.23; -3.35]	
>60	74	71	-2.60	[-9.79; 4.59]	
Atrial fibrillation status					
**CDR132L 5 mg/kg - Placebo**					0.89
Patients with	3	2	-5.48	[-45.15; 34.18]	
Patients without	78	81	-2.84	[-9.78; 4.10]	
**CDR132L 10 mg/kg - Placebo**					0.53
Patients with	5	2	-16.55	[-54.26; 21.16]	
Patients without	81	81	-4.45	[-11.24; 2.33]	
Baseline septum thickness					
**CDR132L 5 mg/kg - Placebo**					0.91
<= 1.2 (cm)	38	43	-3.39	[-13.10; 6.32]	
> 1.2 (cm)	43	40	-2.63	[-12.18; 6.92]	
**CDR132L 10 mg/kg - Placebo**					0.58
<= 1.2 (cm)	47	43	-3.08	[-12.36; 6.20]	
> 1.2 (cm)	38	40	-6.97	[-16.94; 3.00]	
Baseline LVESVi					
**CDR132L 5 mg/kg - Placebo**					0.75
<= 42.45	42	36	-3.99	[-14.18; 6.20]	
> 42.45	39	47	-1.75	[-11.31; 7.81]	
**CDR132L 10 mg/kg - Placebo**					0.67
<= 42.45	47	36	-6.12	[-15.97; 3.73]	
> 42.45	39	47	-3.02	[-12.85; 6.82]	

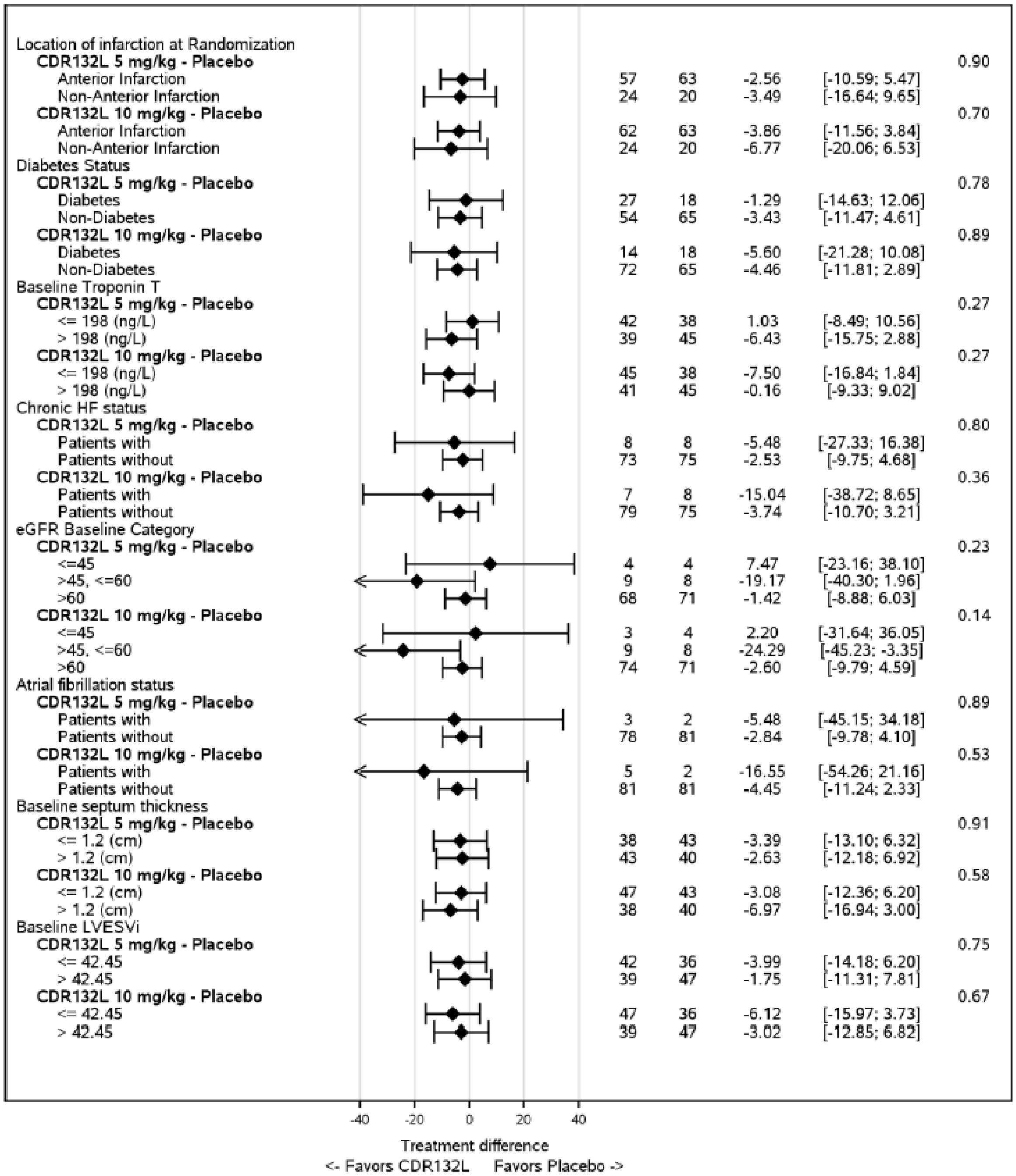

b)

Subgroup	Nact	Npbo	Estimate	[95% CI]	p-value
Pooled Age Group 2					
**CDR132L 5 mg/kg - Placebo**					0.51
< 60	35	38	7.52	[-4.66; 19.70]	
>= 60	46	45	2.21	[-8.53; 12.95]	
**CDR132L 10 mg/kg - Placebo**					0.37
< 60	39	38	-1.87	[-13.61; 9.86]	
>= 60	47	45	-9.11	[-19.81; 1.59]	
Sex					
**CDR132L 5 mg/kg - Placebo**					0.28
F	9	9	-7.72	[-31.66; 16.21]	
M	72	74	5.99	[-2.53; 14.51]	
**CDR132L 10 mg/kg - Placebo**					0.37
F	15	9	3.34	[-18.45; 25.12]	
M	71	74	-7.25	[-15.79; 1.28]	
ACMI Type					
**CDR132L 5 mg/kg - Placebo**					0.92
STEMI	67	69	4.14	[-4.73; 13.02]	
NSTEMI	14	14	5.12	[-14.22; 24.45]	
**CDR132L 10 mg/kg - Placebo**					0.57
STEMI	73	69	-4.79	[-13.33; 3.75]	
NSTEMI	13	14	-10.98	[-30.86; 8.89]	
Time of first Trt. after MI Category					
**CDR132L 5 mg/kg - Placebo**					0.46
>=3 to <=7	15	11	5.01	[-15.95; 25.98]	
>7 to <=14	49	53	1.27	[-9.09; 11.63]	
>14 Days	17	19	13.87	[-3.42; 31.17]	
**CDR132L 10 mg/kg - Placebo**					0.67
>=3 to <=7	19	11	1.79	[-17.93; 21.52]	
>7 to <=14	51	53	-6.64	[-16.82; 3.55]	
>14 Days	16	19	-9.64	[-27.34; 8.05]	
SGLT2 Inhibitors Status					
**CDR132L 5 mg/kg - Placebo**					0.31
Y	65	64	2.24	[-6.84; 11.32]	
N	16	19	12.55	[-5.23; 30.33]	
**CDR132L 10 mg/kg - Placebo**					0.78
Y	70	64	-5.41	[-14.28; 3.46]	
N	16	19	-8.17	[-25.78; 9.44]	
Killip Classification					
**CDR132L 5 mg/kg - Placebo**					NE
KILLIP CLASS I	62	63	3.70	[-5.68; 13.08]	
KILLIP CLASS II	16	18	5.99	[-11.97; 23.96]	
KILLIP CLASS III	1	2	33.53	[-32.09; 99.14]	
**CDR132L 10 mg/kg - Placebo**					NE
KILLIP CLASS I	69	63	-5.94	[-14.94; 3.07]	
KILLIP CLASS II	15	18	-8.25	[-26.45; 9.94]	
KILLIP CLASS III	1	2	21.95	[-43.28; 87.18]	
NYHA class					
**CDR132L 5 mg/kg - Placebo**					0.47
NYHA CLASS I	30	28	8.09	[-5.38; 21.56]	
NYHA CLASS II	45	52	4.54	[-5.72; 14.80]	
NYHA CLASS III	6	3	-15.78	[-51.61; 20.05]	
**CDR132L 10 mg/kg - Placebo**					0.46
NYHA CLASS I	40	28	1.09	[-11.34; 13.53]	
NYHA CLASS II	42	52	-8.73	[-19.35; 1.88]	
NYHA CLASS III	3	3	4.41	[-36.96; 45.77]	
Baseline LVEF					
**CDR132L 5 mg/kg - Placebo**					0.82
<= 35.79 (%)	39	47	3.76	[-7.39; 14.91]	
> 35.79 (%)	42	36	5.64	[-6.45; 17.72]	
**CDR132L 10 mg/kg - Placebo**					0.72
<= 35.79 (%)	40	47	-6.89	[-17.98; 4.21]	
> 35.79 (%)	46	36	-4.05	[-15.60; 7.51]	
Baseline NT-proBNP					
**CDR132L 5 mg/kg - Placebo**					0.13
<= 1286 (pg/mL)	39	43	9.65	[-1.69; 20.99]	
> 1286 (pg/mL)	42	40	-2.56	[-13.81; 8.70]	
**CDR132L 10 mg/kg - Placebo**					0.03
<= 1286 (pg/mL)	44	43	2.74	[-8.19; 13.67]	
> 1286 (pg/mL)	42	40	-14.68	[-25.78; -3.57]	

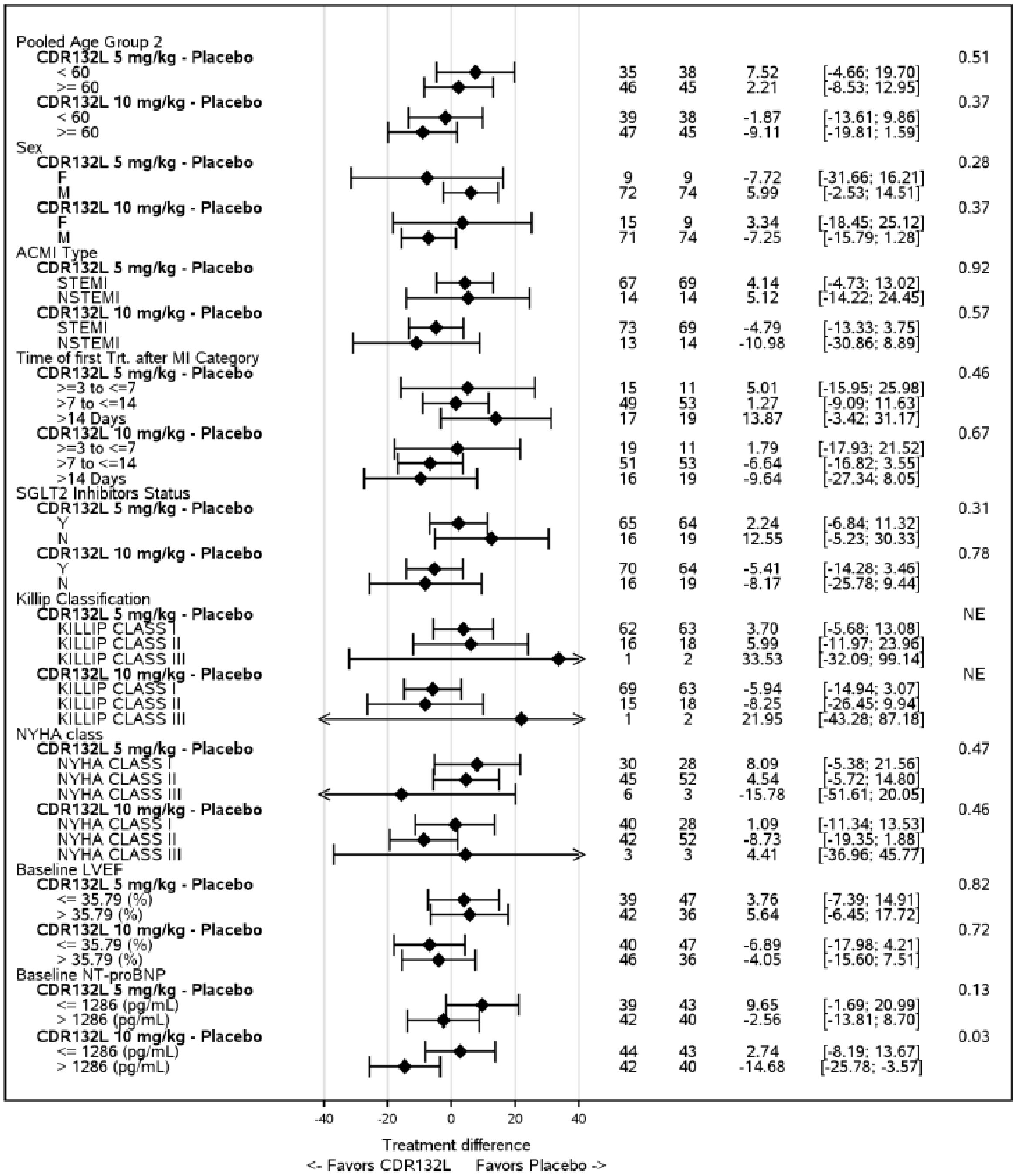

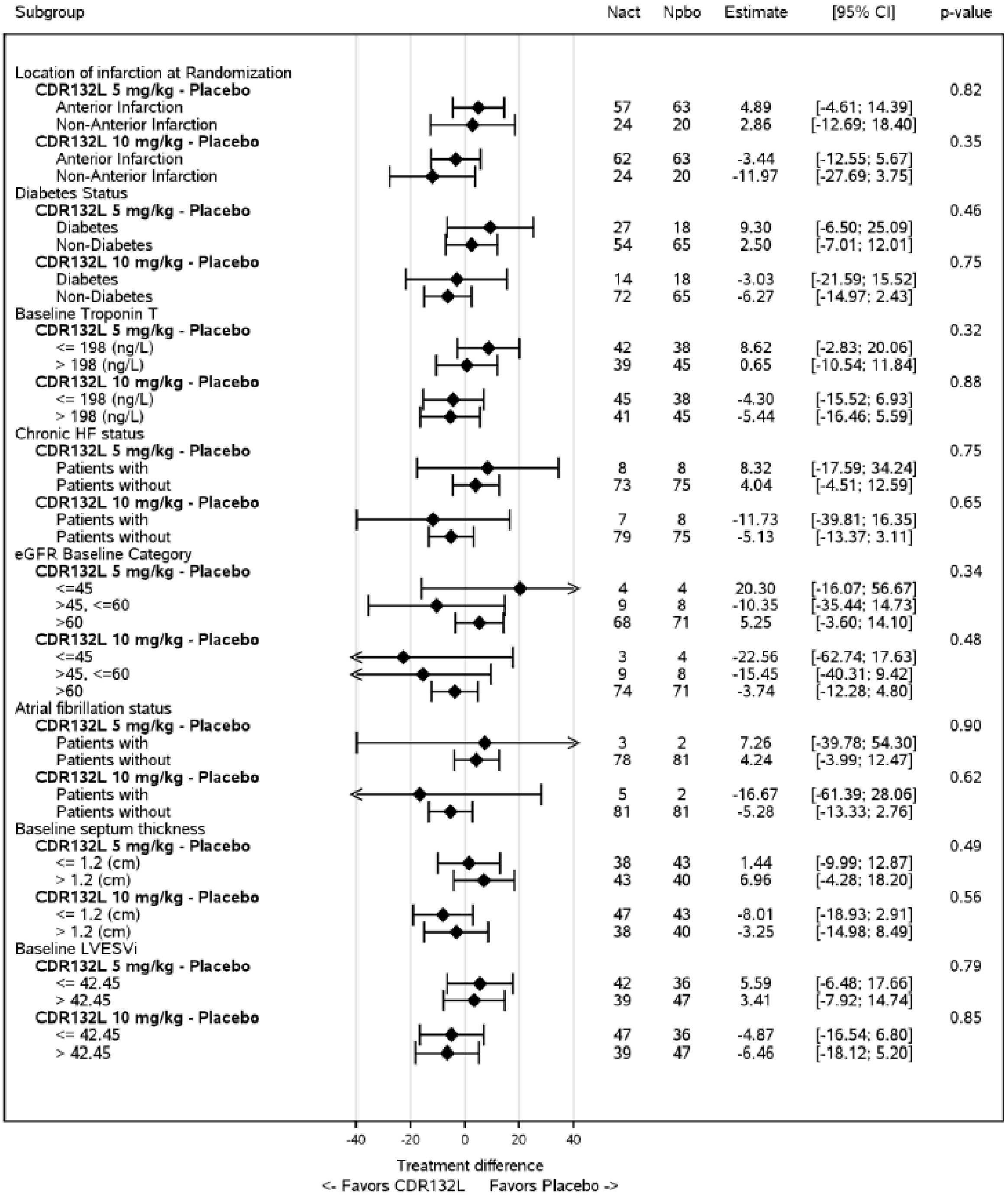

Triangles represent estimates that fall outside the x axis range.

The ANCOVA model includes treatment and center group, stratification factors of age group (<60 years, ≥60 years) and location of infarction (anterior, non-anterior), baseline LVESVI and treatment-by-subgroup interaction. P values are 2-sided. The PP population includes all patients from the ITT population who have completed treatment and the 6-month visit without any major protocol deviations that could affect the validity of primary and secondary efficacy assessments. Missing post-baseline data were imputed using the last observation (including baseline) carried forward approach. All comparisons shown are versus placebo.

ACMI, acute myocardial infarction; ANCOVA, analysis of covariance; Ca, category; CI, confidence interval; eGFR, estimated glomerular filtration rate; HF, heart failure; ITT, intention-to-treat; LVEF, left ventricular ejection fraction; LVESVI, left ventricular end-systolic volume index; MI, myocardial infarction; Nact, number of patients receiving active treatment; NE, not estimable; Npbo, number of patients receiving placebo; NSTEMI, non-ST-segment elevation myocardial infarction; NT-proBNP, N-terminal pro B-type natriuretic peptide; NYHA, New York Heart Association; PP, per protocol; SGLT2, sodium-glucose co-transporter 2; STEMI, ST-segment elevation myocardial infarction; Trt, treatment.

Supplementary Fig. 4: All subgroups for change from baseline in LVEF at a) month 6 and b) month 12. Data are shown for the PP population with each CDR132L dose analyzed separately.

a)

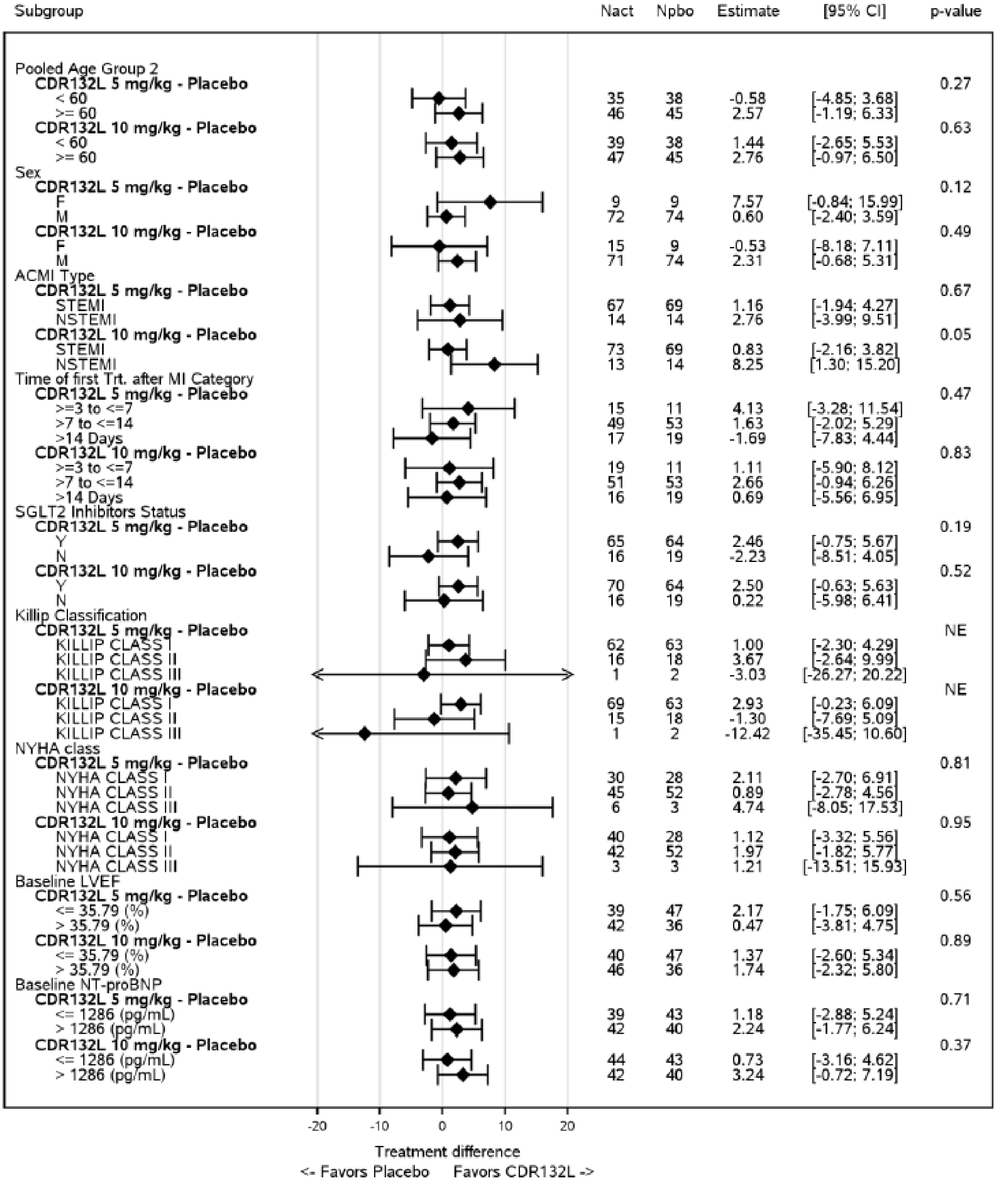

Subgroup	Nact	Npbo	Estimate	[95% CI]	p-value
Location of infarction at Randomization					
**CDR132L 5 mg/kg - Placebo**					0.61
Anterior Infarction	57	63	0.92	[-2.44; 4.29]	
Non-Anterior Infarction	24	20	2.58	[-2.90; 8.07]	
**CDR132L 10 mg/kg - Placebo**					0.76
Anterior Infarction	62	63	1.67	[-1.55; 4.90]	
Non-Anterior Infarction	24	20	2.66	[-2.91; 8.22]	
Diabetes Status					
**CDR132L 5 mg/kg - Placebo**					0.42
Diabetes	27	18	3.44	[-2.15; 9.02]	
Non-Diabetes	54	65	0.78	[-2.58; 4.13]	
**CDR132L 10 mg/kg - Placebo**					0.33
Diabetes	14	18	4.75	[-1.81; 11.30]	
Non-Diabetes	72	65	1.25	[-1.83; 4.32]	
Baseline Troponin T					
**CDR132L 5 mg/kg - Placebo**					0.41
<= 198 (ng/L)	42	38	0.21	[-3.82; 4.24]	
> 198 (ng/L)	39	45	2.54	[-1.40; 6.48]	
**CDR132L 10 mg/kg - Placebo**					0.61
<= 198 (ng/L)	45	38	2.33	[-1.62; 6.27]	
> 198 (ng/L)	41	45	0.91	[-2.97; 4.79]	
Chronic HF status					
**CDR132L 5 mg/kg - Placebo**					0.87
Patients with	8	8	0.71	[-8.46; 9.89]	
Patients without	73	75	1.46	[-1.57; 4.49]	
**CDR132L 10 mg/kg - Placebo**					0.86
Patients with	7	8	2.72	[-7.23; 12.67]	
Patients without	79	75	1.86	[-1.06; 4.77]	
eGFR Baseline Category					
**CDR132L 5 mg/kg - Placebo**					0.12
<=45	4	4	-7.56	[-20.30; 5.18]	
>45, <=60	9	8	8.12	[-0.68; 16.91]	
>60	68	71	1.10	[-2.01; 4.20]	
**CDR132L 10 mg/kg - Placebo**					0.35
<=45	3	4	-4.34	[-18.40; 9.72]	
>45, <=60	9	8	6.91	[-1.79; 15.61]	
>60	74	71	1.61	[-1.38; 4.60]	
Atrial fibrillation status					
**CDR132L 5 mg/kg - Placebo**					0.62
Patients with	3	2	-2.58	[-18.91; 13.75]	
Patients without	78	81	1.58	[-1.29; 4.44]	
**CDR132L 10 mg/kg - Placebo**					0.57
Patients with	5	2	-2.08	[-17.68; 13.52]	
Patients without	81	81	2.38	[-0.43; 5.18]	
Baseline septum thickness					
**CDR132L 5 mg/kg - Placebo**					0.90
<= 1.2 (cm)	38	43	1.30	[-2.78; 5.38]	
> 1.2 (cm)	43	40	1.64	[-2.35; 5.64]	
**CDR132L 10 mg/kg - Placebo**					0.47
<= 1.2 (cm)	47	43	1.05	[-2.83; 4.94]	
> 1.2 (cm)	38	40	3.16	[-1.02; 7.33]	
Baseline LVESVi					
**CDR132L 5 mg/kg - Placebo**					0.48
<= 42.45	42	36	2.47	[-1.76; 6.71]	
> 42.45	39	47	0.41	[-3.54; 4.36]	
**CDR132L 10 mg/kg - Placebo**					0.36
<= 42.45	47	36	3.04	[-1.04; 7.13]	
> 42.45	39	47	0.32	[-3.73; 4.36]	

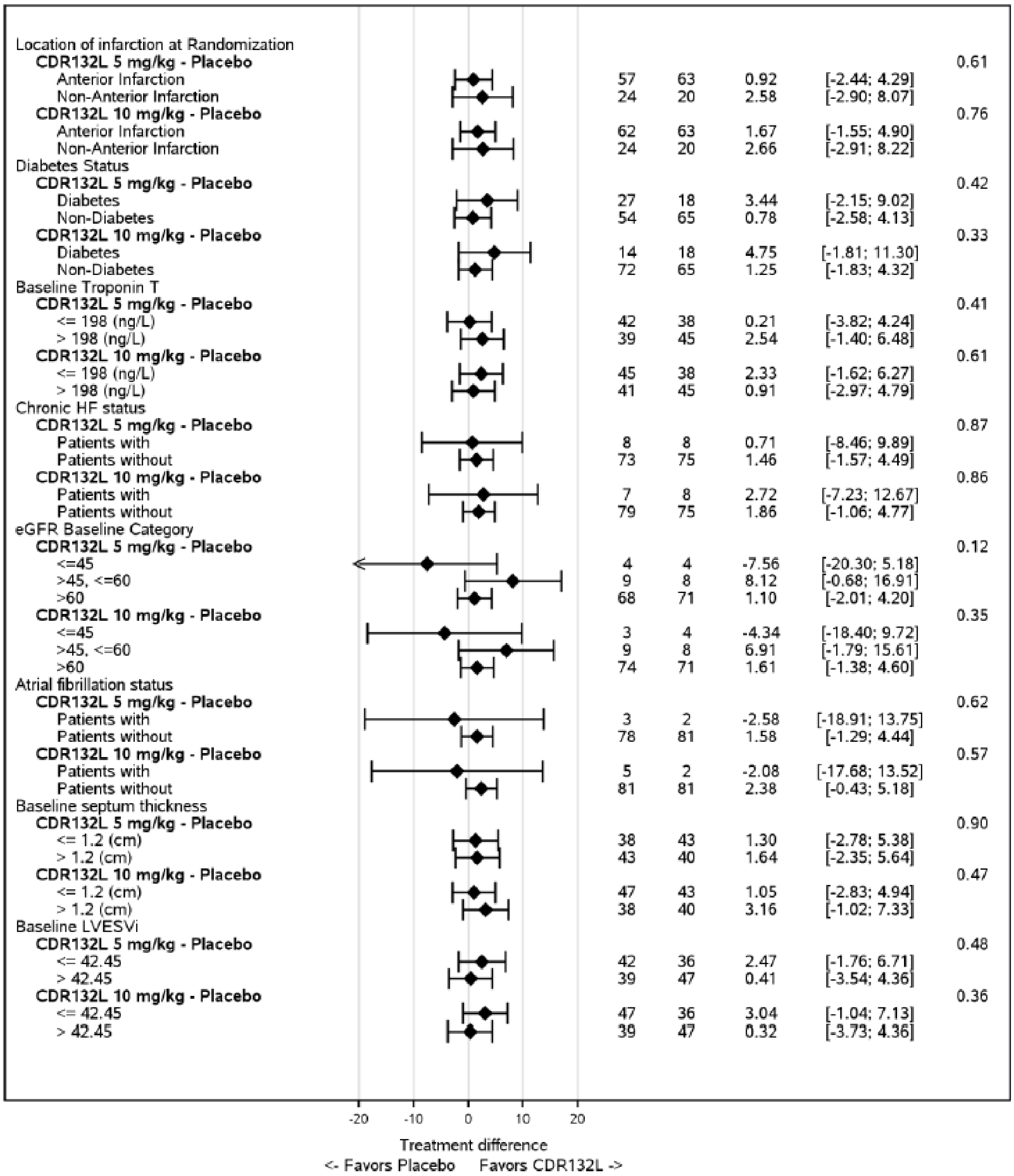

b)

Subgroup	Nact	Npbo	Estimate	[95% CI]	p-value
Pooled Age Group 2					
**CDR132L 5 mg/kg - Placebo**					0.26
< 60	35	38	-2.76	[-7.49; 1.96]	
>= 60	46	45	0.81	[-3.36; 4.98]	
**CDR132L 10 mg/kg - Placebo**					0.45
< 60	39	38	1.88	[-2.65; 6.42]	
>= 60	47	45	4.21	[0.07; 8.35]	
Sex					
**CDR132L 5 mg/kg - Placebo**					0.29
F	9	9	4.09	[-5.21; 13.40]	
M	72	74	-1.15	[-4.46; 2.17]	
**CDR132L 10 mg/kg - Placebo**					0.27
F	15	9	-1.59	[-10.05; 6.87]	
M	71	74	3.45	[0.13; 6.76]	
ACMI Type					
**CDR132L 5 mg/kg - Placebo**					0.33
STEMI	67	69	-1.23	[-4.68; 2.22]	
NSTEMI	14	14	2.85	[-4.66; 10.36]	
**CDR132L 10 mg/kg - Placebo**					0.02
STEMI	73	69	1.42	[-1.91; 4.74]	
NSTEMI	13	14	11.03	[3.29; 18.76]	
Time of first Trt. after MI Category					
**CDR132L 5 mg/kg - Placebo**					0.22
>=3 to <=7	15	11	0.40	[-7.76; 8.56]	
>7 to <=14	49	53	0.87	[-3.16; 4.89]	
>14 Days	17	19	-5.98	[-12.73; 0.77]	
**CDR132L 10 mg/kg - Placebo**					0.90
>=3 to <=7	19	11	1.30	[-6.41; 9.02]	
>7 to <=14	51	53	3.25	[-0.71; 7.22]	
>14 Days	16	19	2.80	[-4.09; 9.68]	
SGLT2 Inhibitors Status					
**CDR132L 5 mg/kg - Placebo**					0.36
Y	65	64	0.21	[-3.36; 3.78]	
N	16	19	-3.46	[-10.45; 3.54]	
**CDR132L 10 mg/kg - Placebo**					0.57
Y	70	64	2.53	[-0.96; 6.01]	
N	16	19	4.77	[-2.13; 11.68]	
Killip Classification					
**CDR132L 5 mg/kg - Placebo**					NE
KILLIP CLASS I	62	63	-0.61	[-4.30; 3.08]	
KILLIP CLASS II	16	18	0.25	[-6.81; 7.32]	
KILLIP CLASS III	1	2	-1.85	[-27.86; 24.16]	
**CDR132L 10 mg/kg - Placebo**					NE
KILLIP CLASS I	69	63	3.13	[-0.41; 6.66]	
KILLIP CLASS II	15	18	3.31	[-3.83; 10.46]	
KILLIP CLASS III	1	2	-6.81	[-32.57; 18.95]	
NYHA class					
**CDR132L 5 mg/kg - Placebo**					0.79
NYHA CLASS I	30	28	-0.86	[-6.18; 4.47]	
NYHA CLASS II	45	52	-0.68	[-4.74; 3.38]	
NYHA CLASS III	6	3	4.20	[-9.97; 18.37]	
**CDR132L 10 mg/kg - Placebo**					0.74
NYHA CLASS I	40	28	1.46	[-3.46; 6.37]	
NYHA CLASS II	42	52	3.48	[-0.72; 7.69]	
NYHA CLASS III	3	3	-1.22	[-17.52; 15.09]	
Baseline LVEF					
**CDR132L 5 mg/kg - Placebo**					0.66
<= 35.79 (%)	39	47	0.03	[-4.34; 4.41]	
> 35.79 (%)	42	36	-1.42	[-6.20; 3.36]	
**CDR132L 10 mg/kg - Placebo**					0.42
<= 35.79 (%)	40	47	4.06	[-0.37; 8.49]	
> 35.79 (%)	46	36	1.48	[-3.06; 6.01]	
Baseline NT-proBNP					
**CDR132L 5 mg/kg - Placebo**					0.86
<= 1286 (pg/mL)	39	43	-0.44	[-4.94; 4.06]	
> 1286 (pg/mL)	42	40	0.09	[-4.34; 4.53]	
**CDR132L 10 mg/kg - Placebo**					0.20
<= 1286 (pg/mL)	44	43	0.95	[-3.35; 5.26]	
> 1286 (pg/mL)	42	40	4.95	[0.57; 9.33]	

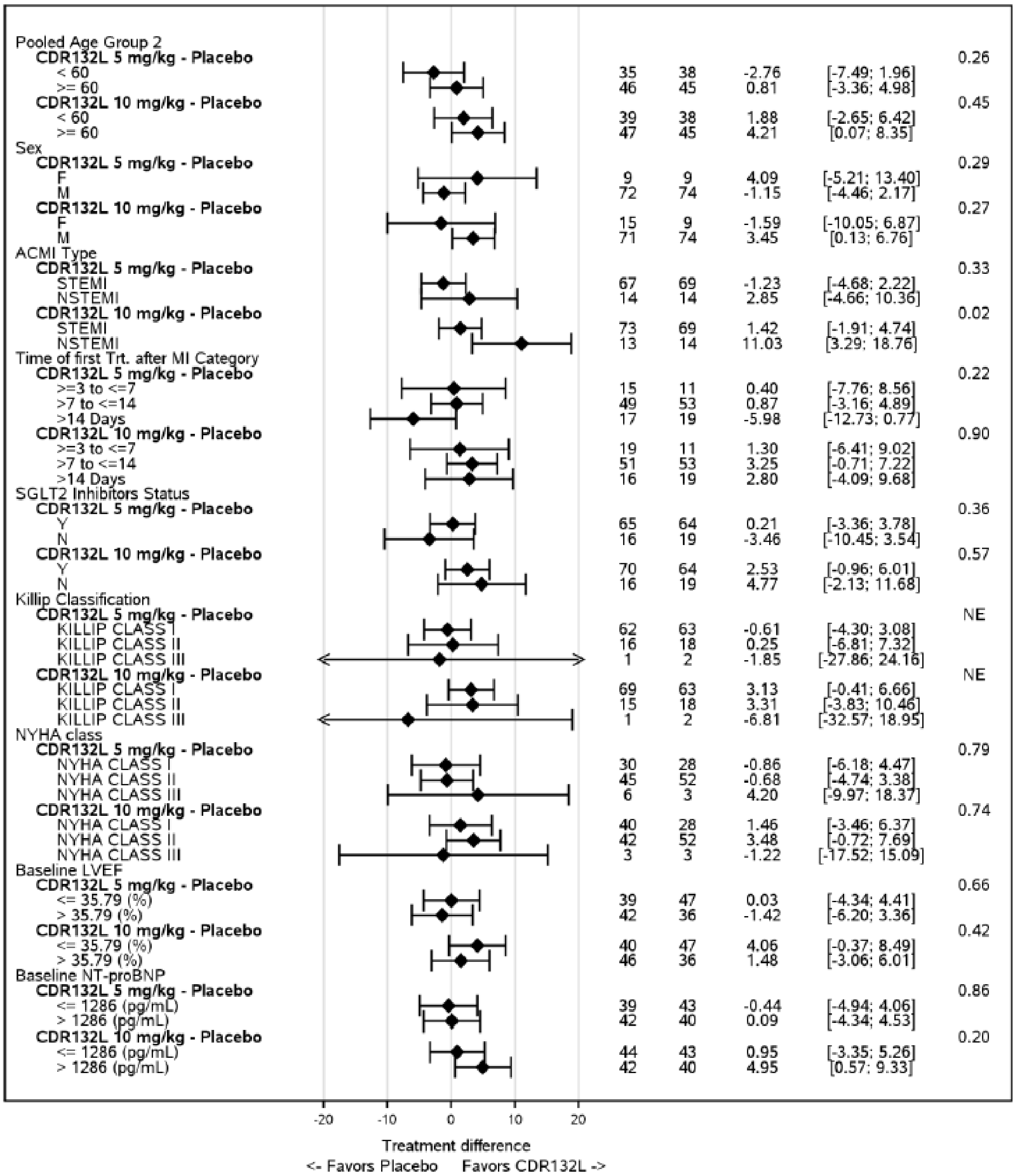

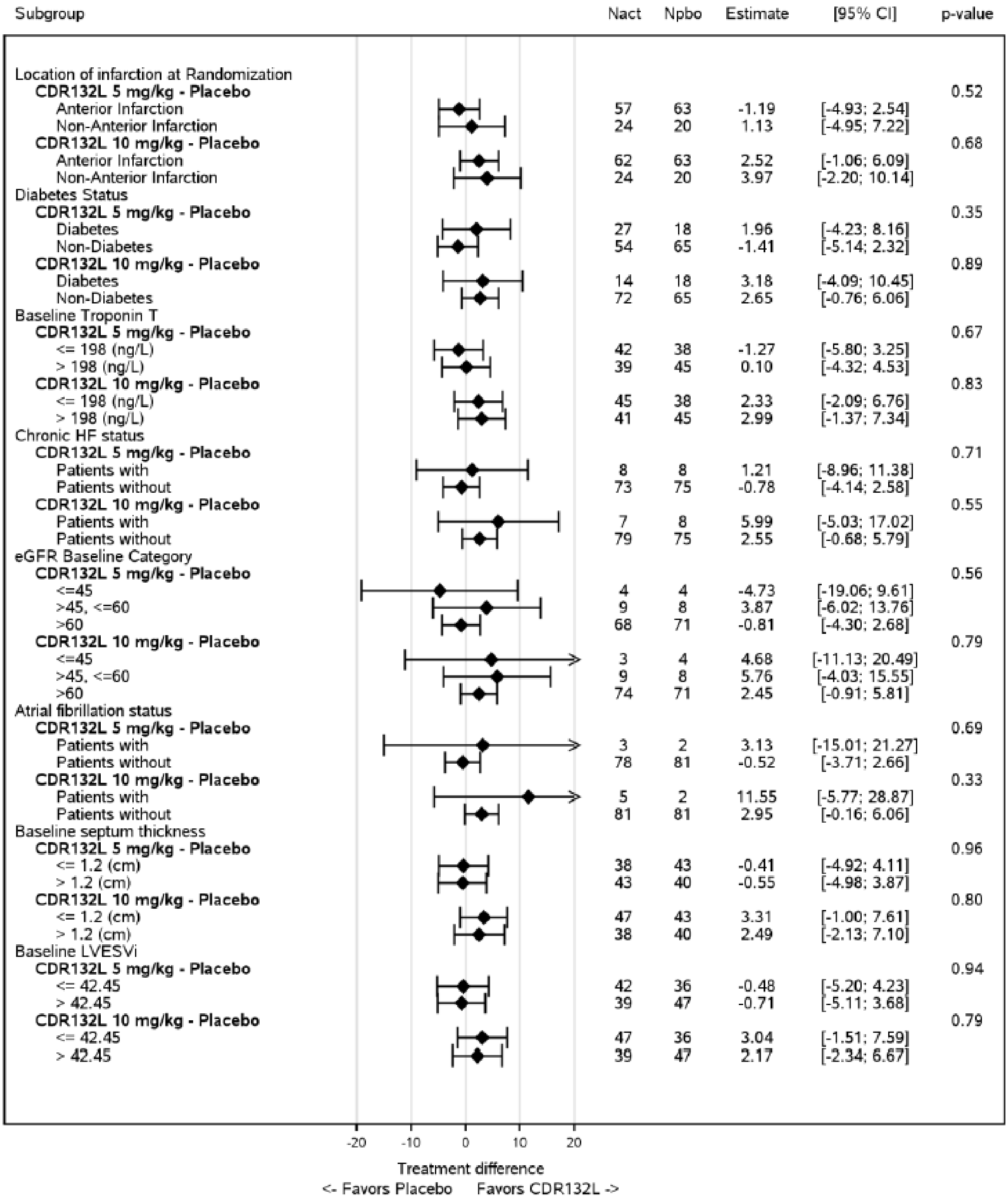

Triangles represent estimates that fall outside the x axis range.
The ANCOVA model includes treatment and center group, stratification factors of age group (<60 years, ≥60 years) and location of infarction (anterior, non-anterior), baseline LVEF and treatment-by-subgroup interaction. P values are 2-sided. The PP population includes all patients from the ITT population who have completed treatment and the 6-month visit without any major protocol deviations that could affect the validity of primary and secondary efficacy assessments. Missing post-baseline data were imputed using the last observation (including baseline) carried forward approach. All comparisons shown are versus placebo.
ACMI, acute myocardial infarction; ANCOVA, analysis of covariance; Ca, category; CI, confidence interval; eGFR, estimated glomerular filtration rate; HF, heart failure; ITT, intention-to-treat; LVEF, left ventricular ejection fraction; LVESVI, left ventricular end-systolic volume index; MI, myocardial infarction; Nact, number of patients receiving active treatment; NE, not estimable; Npbo, number of patients receiving placebo; NSTEMI, non-ST-segment elevation myocardial infarction; NT-proBNP, N-terminal pro B-type natriuretic peptide; NYHA, New York Heart Association; PP, per protocol; Rand, randomization; SGLT2, sodium-glucose co-transporter 2; STEMI, ST-segment elevation myocardial infarction; Trt, treatment.

Supplementary Fig. 5: All subgroups for NT-proBNP ratio to baseline at a) month 6 and b) month 12. Data are shown for the mITT population with each CDR132L dose analyzed separately.

a)

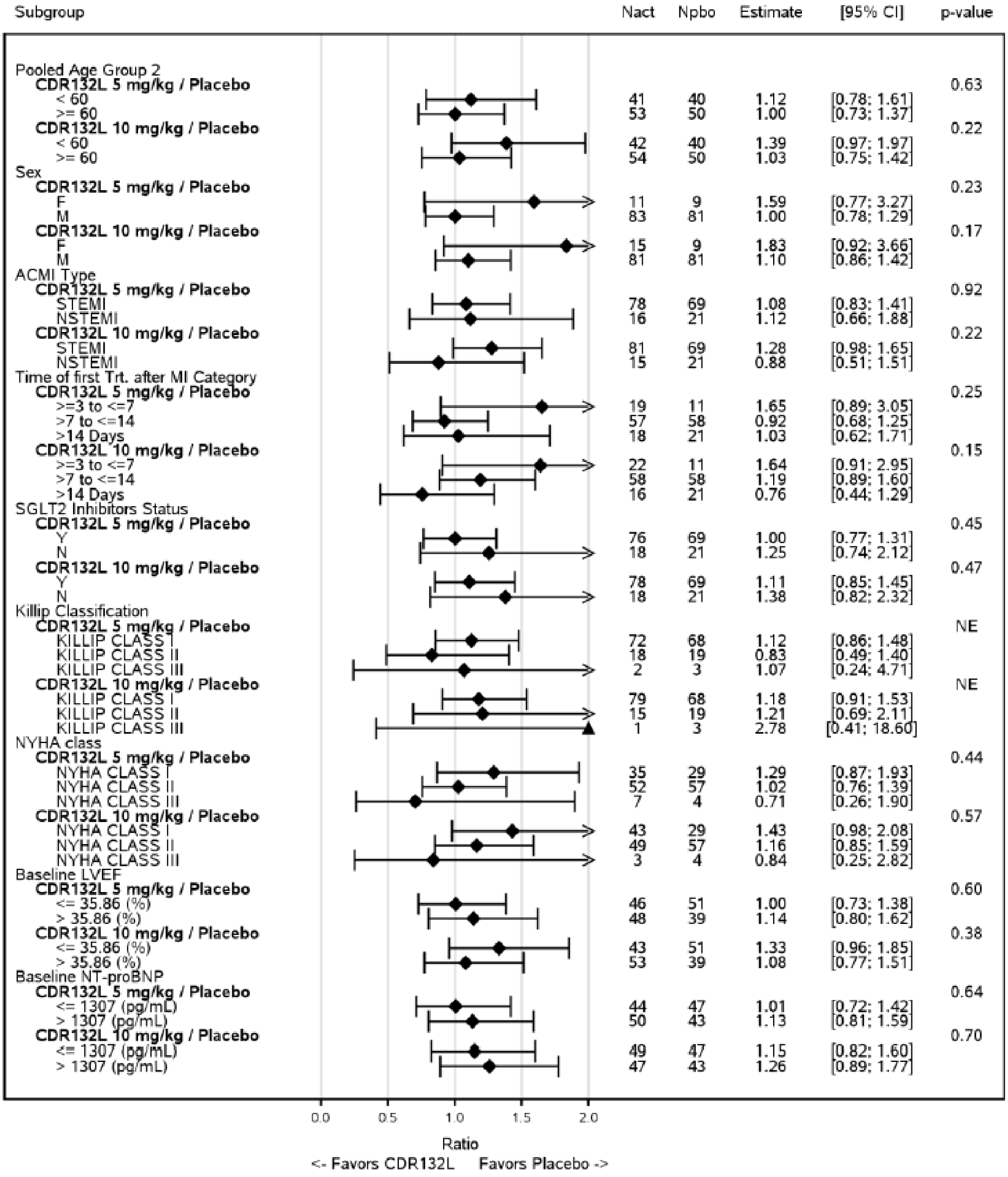

Subgroup	Nact	Npbo	Estimate	[95% CI]	p-value
Location of infarction at Randomization					
**CDR132L 5 mg/kg / Placebo**					0.31
Anterior Infarction	67	64	0.97	[0.73; 1.29]	
Non-Anterior Infarction	27	26	1.27	[0.82; 1.97]	
**CDR132L 10 mg/kg / Placebo**					0.48
Anterior Infarction	68	64	1.23	[0.93; 1.63]	
Non-Anterior Infarction	28	26	1.03	[0.66; 1.59]	
Diabetes Status					
**CDR132L 5 mg/kg / Placebo**					0.23
Diabetes	30	24	0.83	[0.53; 1.28]	
Non-Diabetes	64	66	1.13	[0.86; 1.50]	
**CDR132L 10 mg/kg / Placebo**					0.07
Diabetes	18	24	0.80	[0.48; 1.32]	
Non-Diabetes	78	66	1.33	[1.02; 1.73]	
Baseline Troponin T					
**CDR132L 5 mg/kg / Placebo**					0.65
<= 208.5 (ng/L)	46	46	0.99	[0.71; 1.38]	
> 208.5 (ng/L)	48	44	1.10	[0.79; 1.54]	
**CDR132L 10 mg/kg / Placebo**					0.24
<= 208.5 (ng/L)	48	46	1.02	[0.73; 1.42]	
> 208.5 (ng/L)	48	44	1.34	[0.97; 1.87]	
Chronic HF status					
**CDR132L 5 mg/kg / Placebo**					0.11
Patients with	9	10	0.60	[0.29; 1.26]	
Patients without	85	80	1.13	[0.88; 1.46]	
**CDR132L 10 mg/kg / Placebo**					0.53
Patients with	7	10	0.93	[0.40; 2.12]	
Patients without	89	80	1.22	[0.95; 1.56]	
eGFR Baseline Category					
**CDR132L 5 mg/kg / Placebo**					0.37
<=45	6	5	0.74	[0.29; 1.93]	
>45, <=60	10	9	0.71	[0.35; 1.48]	
>60	78	76	1.14	[0.88; 1.47]	
**CDR132L 10 mg/kg / Placebo**					0.19
<=45	3	5	0.50	[0.16; 1.59]	
>45, <=60	10	9	0.87	[0.42; 1.78]	
>60	83	76	1.29	[1.00; 1.65]	
Atrial fibrillation status					
**CDR132L 5 mg/kg / Placebo**					0.33
Patients with	4	3	1.88	[0.55; 6.37]	
Patients without	90	87	1.02	[0.80; 1.30]	
**CDR132L 10 mg/kg / Placebo**					0.81
Patients with	5	3	0.99	[0.29; 3.36]	
Patients without	91	87	1.15	[0.91; 1.46]	
Baseline septum thickness					
**CDR132L 5 mg/kg / Placebo**					0.25
<= 1.2 (cm)	44	49	1.21	[0.86; 1.69]	
> 1.2 (cm)	50	41	0.92	[0.65; 1.29]	
**CDR132L 10 mg/kg / Placebo**					0.48
<= 1.2 (cm)	49	49	1.28	[0.92; 1.78]	
> 1.2 (cm)	46	41	1.07	[0.76; 1.52]	
Baseline LVESVi					
**CDR132L 5 mg/kg / Placebo**					0.95
<= 42.775	49	41	1.07	[0.76; 1.52]	
> 42.775	45	49	1.06	[0.76; 1.48]	
**CDR132L 10 mg/kg / Placebo**					0.86
<= 42.775	50	41	1.20	[0.85; 1.70]	
> 42.775	46	49	1.15	[0.83; 1.61]	

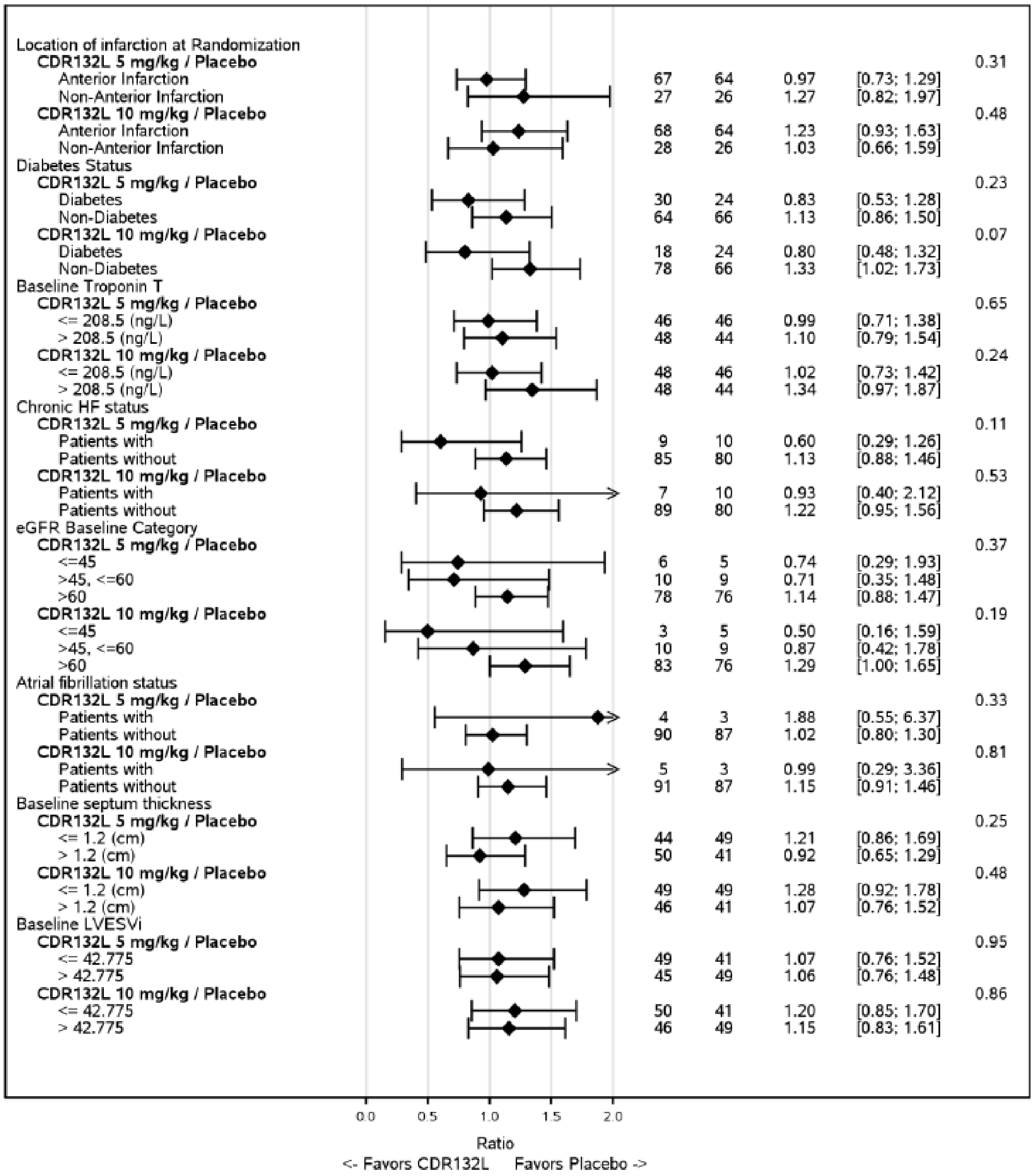

b)

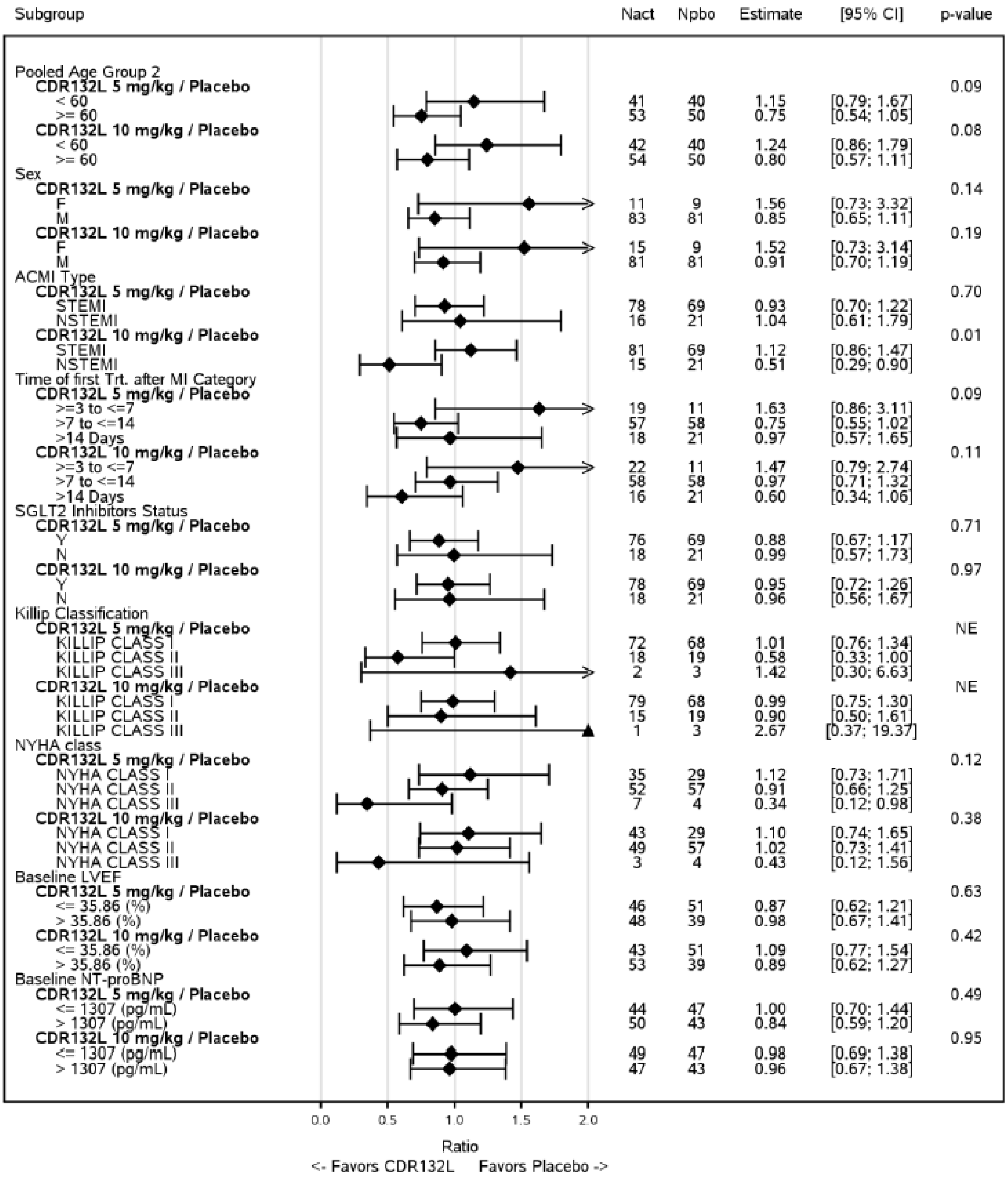
Subgroup
Nact
Npbo
Estimate
[95% CI]
p-value
Pooled Age Group 2
CDR132L 5 mg/kg / Placebo 0.09
< 60 41 40 1.15 [0.79; 1.67]
>= 60 53 50 0.75 [0.54; 1.05]
CDR132L 10 mg/kg / Placebo 0.08
< 60 42 40 1.24 [0.86; 1.79]
>= 60 54 50 0.80 [0.57; 1.11]
Sex
CDR132L 5 mg/kg / Placebo 0.14
F 11 9 1.56 [0.73; 3.32]
M 83 81 0.85 [0.65; 1.11]
CDR132L 10 mg/kg / Placebo 0.19
F 15 9 1.52 [0.73; 3.14]
M 81 81 0.91 [0.70; 1.19]
ACMI Type
CDR132L 5 mg/kg / Placebo 0.70
STEMI 78 69 0.93 [0.70; 1.22]
NSTEMI 16 21 1.04 [0.61; 1.79]
CDR132L 10 mg/kg / Placebo 0.01
STEMI 81 69 1.12 [0.86; 1.47]
NSTEMI 15 21 0.51 [0.29; 0.90]
Time of first Trt. after MI Category
CDR132L 5 mg/kg / Placebo 0.09
>=3 to <=7 19 11 1.63 [0.86; 3.11]
>7 to <=14 57 58 0.75 [0.55; 1.02]
>14 Days 18 21 0.97 [0.57; 1.65]
CDR132L 10 mg/kg / Placebo 0.11
>=3 to <=7 22 11 1.47 [0.79; 2.74]
>7 to <=14 58 58 0.97 [0.71; 1.32]
>14 Days 16 21 0.60 [0.34; 1.06]
SGLT2 Inhibitors Status
CDR132L 5 mg/kg / Placebo 0.71
Y 76 69 0.88 [0.67; 1.17]
N 18 21 0.99 [0.57; 1.73]
CDR132L 10 mg/kg / Placebo 0.97
Y 78 69 0.95 [0.72; 1.26]
N 18 21 0.96 [0.56; 1.67]
Killip Classification
CDR132L 5 mg/kg / Placebo NE
KILLIP CLASS I 72 68 1.01 [0.76; 1.34]
KILLIP CLASS II 18 19 0.58 [0.33; 1.00]
KILLIP CLASS III 2 3 1.42 [0.30; 6.63]
CDR132L 10 mg/kg / Placebo NE
KILLIP CLASS I 79 68 0.99 [0.75; 1.30]
KILLIP CLASS II 15 19 0.90 [0.50; 1.61]
KILLIP CLASS III 1 3 2.67 [0.37; 19.37]
NYHA class
CDR132L 5 mg/kg / Placebo 0.12
NYHA CLASS I 35 29 1.12 [0.73; 1.71]
NYHA CLASS II 52 57 0.91 [0.66; 1.25]
NYHA CLASS III 7 4 0.34 [0.12; 0.98]
CDR132L 10 mg/kg / Placebo 0.38
NYHA CLASS I 43 29 1.10 [0.74; 1.65]
NYHA CLASS II 49 57 1.02 [0.73; 1.41]
NYHA CLASS III 3 4 0.43 [0.12; 1.56]
Baseline LVEF
CDR132L 5 mg/kg / Placebo 0.63
<= 35.86 (%) 46 51 0.87 [0.62; 1.21]
> 35.86 (%) 48 39 0.98 [0.67; 1.41]
CDR132L 10 mg/kg / Placebo 0.42
<= 35.86 (%) 43 51 1.09 [0.77; 1.54]
> 35.86 (%) 53 39 0.89 [0.62; 1.27]
Baseline NT-proBNP
CDR132L 5 mg/kg / Placebo 0.49
<= 1307 (pg/mL) 44 47 1.00 [0.70; 1.44]
> 1307 (pg/mL) 50 43 0.84 [0.59; 1.20]
CDR132L 10 mg/kg / Placebo 0.95
<= 1307 (pg/mL) 49 47 0.98 [0.69; 1.38]
> 1307 (pg/mL) 47 43 0.96 [0.67; 1.38]
0.0
0.5
1.0
1.5
2.0
Ratio
<- Favors CDR132L
Favors Placebo ->

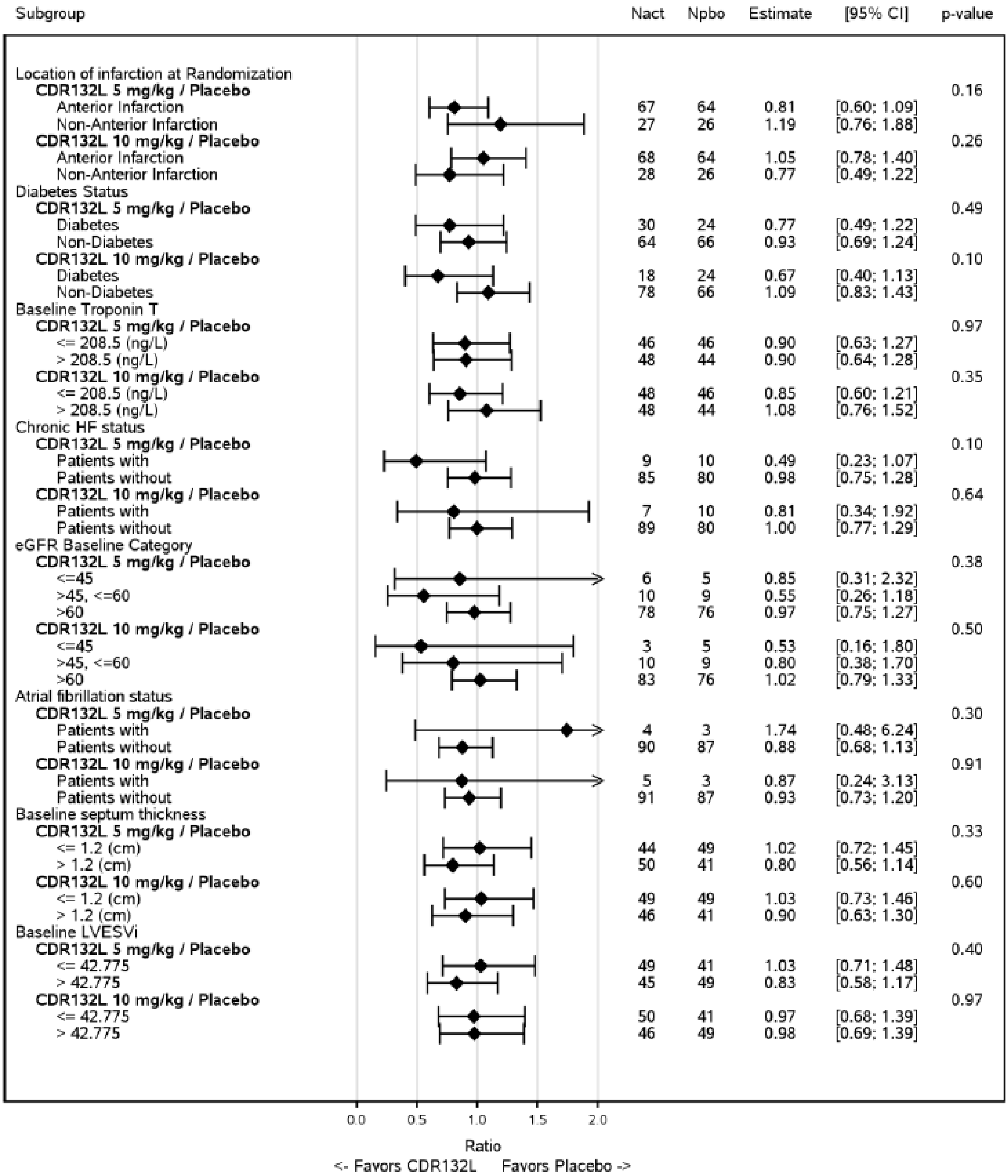

Triangles represent estimates that fall outside the x axis range.
The ANCOVA model includes treatment and center group, stratification factors of age group (<60 years, ≥60 years) and location of infarction (anterior, non-anterior), baseline NT-proBNP (log transformed) and treatment-by-subgroup interaction. P values are 2-sided. The mITT population includes all randomized patients who received at least 1 dose of study drug (CDR132L or placebo). All comparisons shown are versus placebo.
ACMI, acute myocardial infarction; ANCOVA, analysis of covariance; Ca, category; CI, confidence interval; eGFR, estimated glomerular filtration rate; HF, heart failure; LVEF, left ventricular ejection fraction; LVESVI, left ventricular end-systolic volume index; MI, myocardial infarction; mITT, modified intention-to-treat; Nact, number of patients receiving active treatment; NE, not estimable; Npbo, number of patients receiving placebo; NSTEMI, non-ST-segment elevation myocardial infarction; NT-proBNP, N-terminal pro B-type natriuretic peptide; NYHA, New York Heart Association; Rand, randomization; SGLT2, sodium-glucose co-transporter 2; STEMI, ST-segment elevation myocardial infarction; Trt, treatment.

Supplementary Fig. 6: All subgroups for NT-proBNP ratio to baseline at a) month 6 and b) month 12. Data are shown for the PP population with each CDR132L dose analyzed separately.

a)

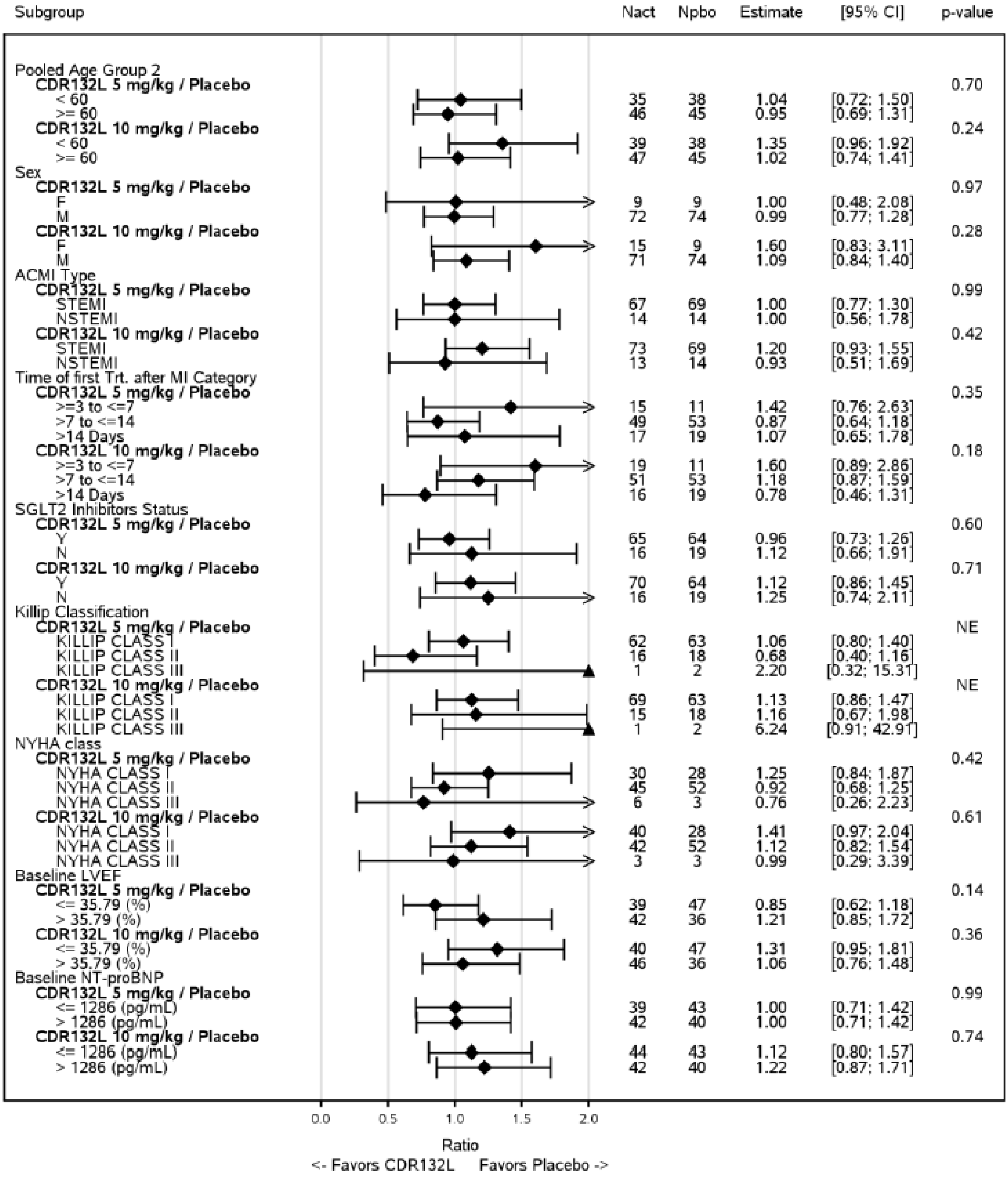

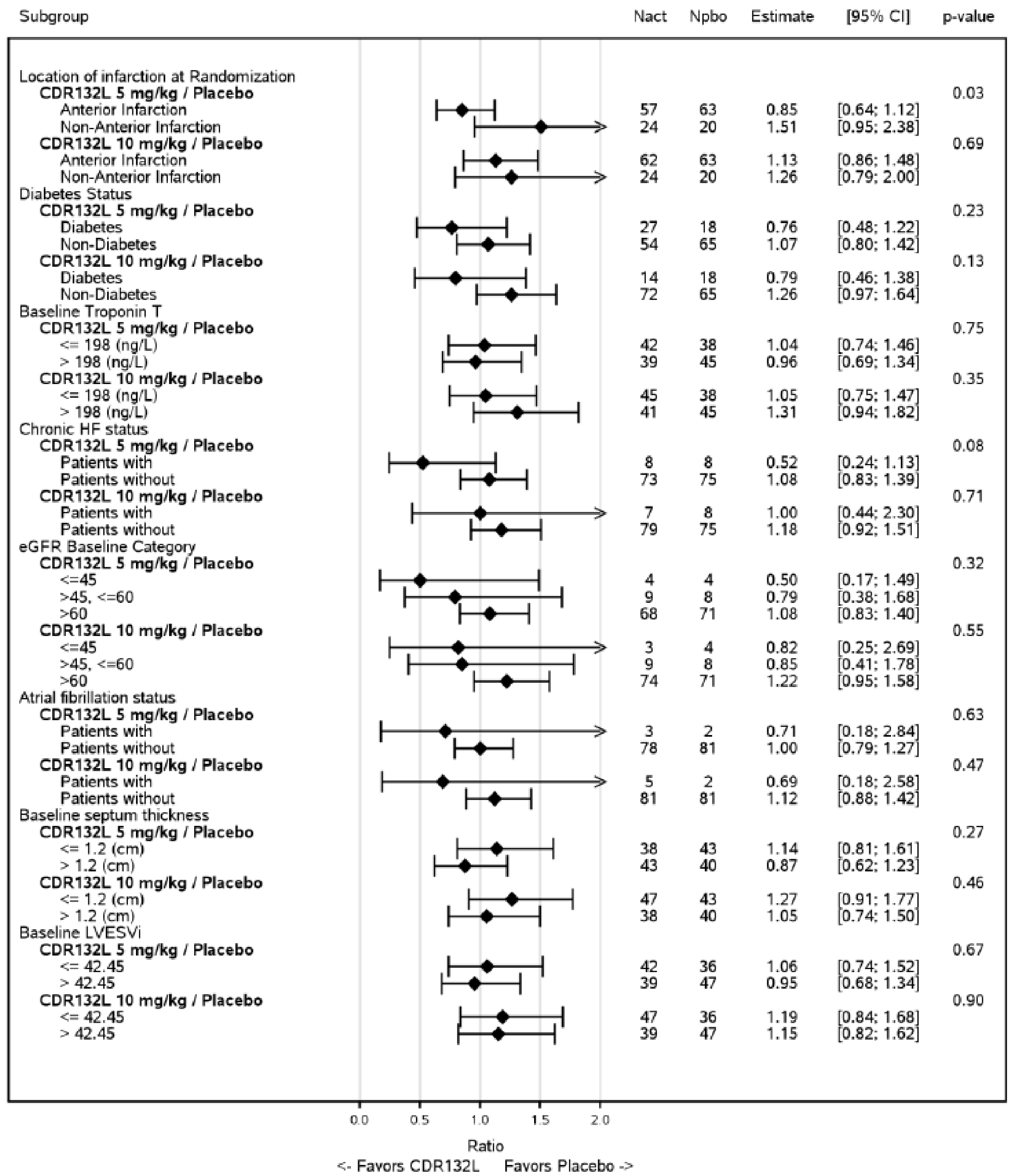
Subgroup
Nact
Npbo
Estimate
[95% CI]
p-value
Location of infarction at Randomization
CDR132L 5 mg/kg / Placebo 0.03
Anterior Infarction 57 63 0.85 [0.64; 1.12]
Non-Anterior Infarction 24 20 1.51 [0.95; 2.38]
CDR132L 10 mg/kg / Placebo 0.69
Anterior Infarction 62 63 1.13 [0.86; 1.48]
Non-Anterior Infarction 24 20 1.26 [0.79; 2.00]
Diabetes Status
CDR132L 5 mg/kg / Placebo 0.23
Diabetes 27 18 0.76 [0.48; 1.22]
Non-Diabetes 54 65 1.07 [0.80; 1.42]
CDR132L 10 mg/kg / Placebo 0.13
Diabetes 14 18 0.79 [0.46; 1.38]
Non-Diabetes 72 65 1.26 [0.97; 1.64]
Baseline Troponin T
CDR132L 5 mg/kg / Placebo 0.75
<= 198 (ng/L) 42 38 1.04 [0.74; 1.46]
> 198 (ng/L) 39 45 0.96 [0.69; 1.34]
CDR132L 10 mg/kg / Placebo 0.35
<= 198 (ng/L) 45 38 1.05 [0.75; 1.47]
> 198 (ng/L) 41 45 1.31 [0.94; 1.82]
Chronic HF status
CDR132L 5 mg/kg / Placebo 0.08
Patients with 8 8 0.52 [0.24; 1.13]
Patients without 73 75 1.08 [0.83; 1.39]
CDR132L 10 mg/kg / Placebo 0.71
Patients with 7 8 1.00 [0.44; 2.30]
Patients without 79 75 1.18 [0.92; 1.51]
eGFR Baseline Category
CDR132L 5 mg/kg / Placebo 0.32
<=45 4 4 0.50 [0.17; 1.49]
>45, <=60 9 8 0.79 [0.38; 1.68]
>60 68 71 1.08 [0.83; 1.40]
CDR132L 10 mg/kg / Placebo 0.55
<=45 3 4 0.82 [0.25; 2.69]
>45, <=60 9 8 0.85 [0.41; 1.78]
>60 74 71 1.22 [0.95; 1.58]
Atrial fibrillation status
CDR132L 5 mg/kg / Placebo 0.63
Patients with 3 2 0.71 [0.18; 2.84]
Patients without 78 81 1.00 [0.79; 1.27]
CDR132L 10 mg/kg / Placebo 0.47
Patients with 5 2 0.69 [0.18; 2.58]
Patients without 81 81 1.12 [0.88; 1.42]
Baseline septum thickness
CDR132L 5 mg/kg / Placebo 0.27
<= 1.2 (cm) 38 43 1.14 [0.81; 1.61]
> 1.2 (cm) 43 40 0.87 [0.62; 1.23]
CDR132L 10 mg/kg / Placebo 0.46
<= 1.2 (cm) 47 43 1.27 [0.91; 1.77]
> 1.2 (cm) 38 40 1.05 [0.74; 1.50]
Baseline LVESVi
CDR132L 5 mg/kg / Placebo 0.67
<= 42.45 42 36 1.06 [0.74; 1.52]
> 42.45 39 47 0.95 [0.68; 1.34]
CDR132L 10 mg/kg / Placebo 0.90
<= 42.45 47 36 1.19 [0.84; 1.68]
> 42.45 39 47 1.15 [0.82; 1.62]
0.0
0.5
1.0
1.5
2.0
Ratio
<- Favors CDR132L
Favors Placebo ->

b)

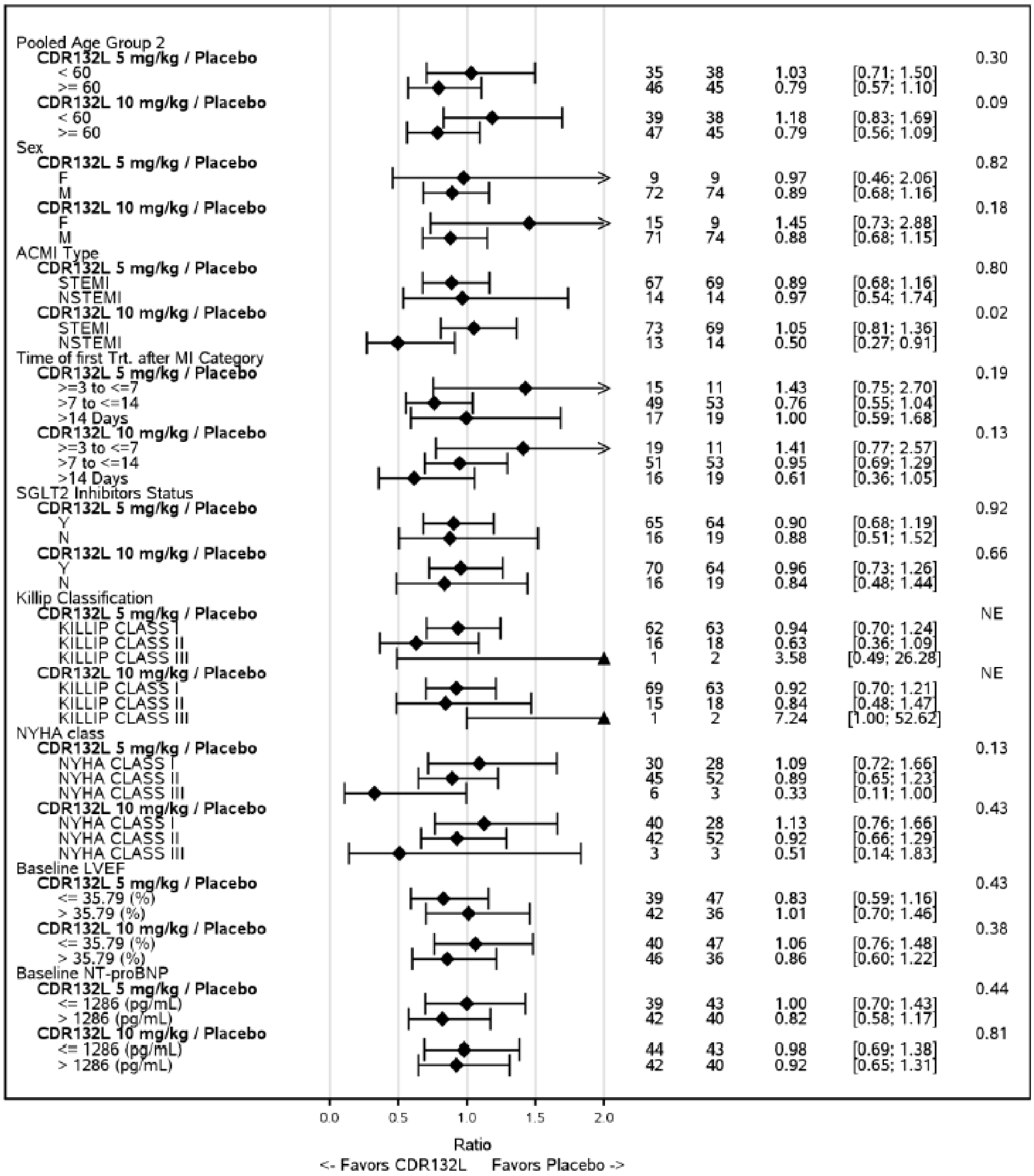

Subgroup	Nact	Npbo	Estimate	[95% CI]	p-value
Pooled Age Group 2					
**CDR132L 5 mg/kg / Placebo**					0.30
< 60	35	38	1.03	[0.71; 1.50]	
>= 60	46	45	0.79	[0.57; 1.10]	
**CDR132L 10 mg/kg / Placebo**					0.09
< 60	39	38	1.18	[0.83; 1.69]	
>= 60	47	45	0.79	[0.56; 1.09]	
Sex					
**CDR132L 5 mg/kg / Placebo**					0.82
F	9	9	0.97	[0.46; 2.06]	
M	72	74	0.89	[0.68; 1.16]	
**CDR132L 10 mg/kg / Placebo**					0.18
F	15	9	1.45	[0.73; 2.88]	
M	71	74	0.88	[0.68; 1.15]	
ACMI Type					
**CDR132L 5 mg/kg / Placebo**					0.80
STEMI	67	69	0.89	[0.68; 1.16]	
NSTEMI	14	14	0.97	[0.54; 1.74]	
**CDR132L 10 mg/kg / Placebo**					0.02
STEMI	73	69	1.05	[0.81; 1.36]	
NSTEMI	13	14	0.50	[0.27; 0.91]	
Time of first Trt. after MI Category					
**CDR132L 5 mg/kg / Placebo**					0.19
>=3 to <=7	15	11	1.43	[0.75; 2.70]	
>7 to <=14	49	53	0.76	[0.55; 1.04]	
>14 Days	17	19	1.00	[0.59; 1.68]	
**CDR132L 10 mg/kg / Placebo**					0.13
>=3 to <=7	19	11	1.41	[0.77; 2.57]	
>7 to <=14	51	53	0.95	[0.69; 1.29]	
>14 Days	16	19	0.61	[0.36; 1.05]	
SGLT2 Inhibitors Status					
**CDR132L 5 mg/kg / Placebo**					0.92
Y	65	64	0.90	[0.68; 1.19]	
N	16	19	0.88	[0.51; 1.52]	
**CDR132L 10 mg/kg / Placebo**					0.66
Y	70	64	0.96	[0.73; 1.26]	
N	16	19	0.84	[0.48; 1.44]	
Killip Classification					
**CDR132L 5 mg/kg / Placebo**					NE
KILLIP CLASS I	62	63	0.94	[0.70; 1.24]	
KILLIP CLASS II	16	18	0.63	[0.36; 1.09]	
KILLIP CLASS III	1	2	3.58	[0.49; 26.28]	
**CDR132L 10 mg/kg / Placebo**					NE
KILLIP CLASS I	69	63	0.92	[0.70; 1.21]	
KILLIP CLASS II	15	18	0.84	[0.48; 1.47]	
KILLIP CLASS III	1	2	7.24	[1.00; 52.62]	
NYHA class					
**CDR132L 5 mg/kg / Placebo**					0.13
NYHA CLASS I	30	28	1.09	[0.72; 1.66]	
NYHA CLASS II	45	52	0.89	[0.65; 1.23]	
NYHA CLASS III	6	3	0.33	[0.11; 1.00]	
**CDR132L 10 mg/kg / Placebo**					0.43
NYHA CLASS I	40	28	1.13	[0.76; 1.66]	
NYHA CLASS II	42	52	0.92	[0.66; 1.29]	
NYHA CLASS III	3	3	0.51	[0.14; 1.83]	
Baseline LVEF					
**CDR132L 5 mg/kg / Placebo**					0.43
<= 35.79 (%)	39	47	0.83	[0.59; 1.16]	
> 35.79 (%)	42	36	1.01	[0.70; 1.46]	
**CDR132L 10 mg/kg / Placebo**					0.38
<= 35.79 (%)	40	47	1.06	[0.76; 1.48]	
> 35.79 (%)	46	36	0.86	[0.60; 1.22]	
Baseline NT-proBNP					
**CDR132L 5 mg/kg / Placebo**					0.44
<= 1286 (pg/mL)	39	43	1.00	[0.70; 1.43]	
> 1286 (pg/mL)	42	40	0.82	[0.58; 1.17]	
**CDR132L 10 mg/kg / Placebo**					0.81
<= 1286 (pg/mL)	44	43	0.98	[0.69; 1.38]	
> 1286 (pg/mL)	42	40	0.92	[0.65; 1.31]	

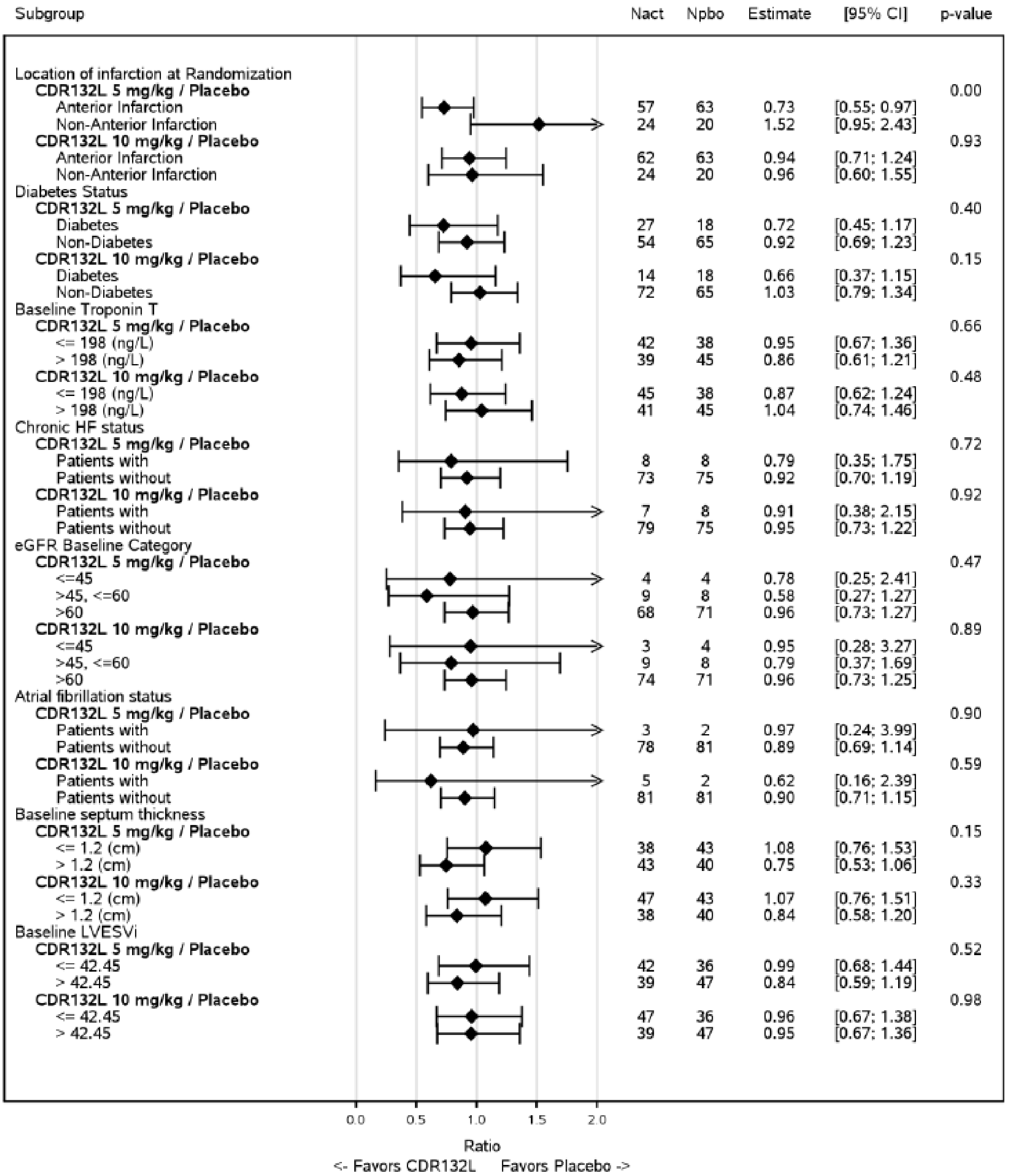

Triangles represent estimates that fall outside the x axis range.
The ANCOVA model includes treatment and center group, stratification factors of age group (<60 years, ≥60 years) and location of infarction (anterior, non-anterior), baseline NT-proBNP (log transformed) and treatment-by-subgroup interaction. P values are 2-sided. The PP population includes all patients from the ITT population who have completed treatment and the 6-month visit without any major protocol deviations that could affect the validity of primary and secondary efficacy assessments. All comparisons shown are versus placebo.
ACMI, acute myocardial infarction; ANCOVA, analysis of covariance; Ca, category; CI, confidence interval; eGFR, estimated glomerular filtration rate; HF, heart failure; ITT, intention-to-treat; LVEF, left ventricular ejection fraction; LVESVI, left ventricular end-systolic volume index; MI, myocardial infarction; Nact, number of patients receiving active treatment; NE, not estimable; Npbo, number of patients receiving placebo; NSTEMI, non-ST-segment elevation myocardial infarction; NT-proBNP, N-terminal pro B-type natriuretic peptide; NYHA, New York Heart Association; PP, per protocol; Rand, randomization; SGLT2, ST-segment elevation myocardial infarction; STEMI, ST-segment elevation myocardial infarction; Trt, treatment.

TITLE PAGE

Protocol Title: P**h**ase 2, Multicenter, Randomized, Parallel, 3-arm, Placebo-controlled Study to Assess E**f**ficacy and Safety of CD**R**132L in Pati**e**nts with Reduced Left **V**entricular **E**jection F**r**ac**t**ion (≤ 45%) After Myocardial Infarction (**HF-REVERT**)

Protocol Number: CDR132L-P2-01

Amendment Number: Original Protocol

Product: CDR132L

Short Title: P**h**ase 2 Study to Assess E**f**ficacy and Safety of CD**R**132L in Pati**e**nts with Reduced Left **V**entricular **E**jection F**r**ac**t**ion (≤ 45%) After Myocardial Infarction (**HF-REVERT**)

Study Phase: 2

Sponsor Name: Cardior Pharmaceuticals GmbH

Sponsor Address: Hollerithallee 20
D-30419 Hannover
Germany

Regulatory Agency Identifying Number(s): EudraCT Number: 2021-006040-27

Date of Protocol: 07 December 2021

Redacted protocol
includes redaction of personal identifiable and company confidential information.

.

Sponsor Signatory:

I have read this protocol in its entirety and agree to conduct the study accordingly:

[redacted] ______________ [redacted]
Date

[redacted] ______________ [redacted]
Date

[redacted] ______________ [redacted]
Date

Investigator Agreement Page is provided in Appendix 6. The Investigator should retain the original in the study center study files and return a copy to the Sponsor or Contract Research Organization for archiving.

Each Investigator should be sent a copy of the Investigator Agreement Page for completion. Signatures are obtained after the Sponsor has finalized and approved the protocol.

TABLE OF CONTENTS

TABLE OF TABLES **6**

TABLE OF FIGURES **7**

1.0 PROTOCOL SUMMARY **8**
1.1 Synopsis **8**
1.2 Schema **12**
1.3 Schedule of Activities **13**

2.0 INTRODUCTION **16**
2.1 Study Rationale **16**
2.2 Background **17**
2.2.1 Overview of Non-clinical Studies 17
2.2.2 Overview of Clinical Studies 21
2.3 Benefit/Risk Assessment **21**

3.0 OBJECTIVES AND ENDPOINTS **23**

4.0 STUDY DESIGN **25**
4.1 Overall Design **25**
4.2 Scientific Rationale for Study Design **25**
4.2.1 Rationale for Patient Selection 26
4.3 Justification for Dose **27**
4.3.1 Justification for Selection of Planned CDR132L Doses 27
4.3.2 Timing of Dose for Each Patient 32
4.4 End of Study Definition **32**

5.0 STUDY POPULATION **33**
5.1 Inclusion Criteria **33**
5.2 Exclusion Criteria **33**
5.3 Lifestyle Recommendations **35**
5.3.1 Diet Recommendations 35
5.3.2 Caffeine, Alcohol, and Tobacco 35
5.3.3 Activity 35
5.4 Screen Failures **36**

6.0 STUDY TREATMENT **37**
6.1 Study Treatment(s) Administered **37**
6.2 Preparation/Handling/Storage/Accountability **38**
6.3 Measures to Minimize Bias: Randomization and Blinding **39**
6.4 Study Treatment Compliance **39**
6.5 Concomitant Therapy **40**

6.5.1 Prohibited Therapies....40
6.6 Dose Modification 41
6.7 Treatment After the End of the Study 41

7.0 DISCONTINUATION OF STUDY TREATMENT AND PATIENT DISCONTINUATION/WITHDRAWAL 42
7.1 Discontinuation of Study Treatment....42
7.2 Patient Discontinuation/Withdrawal from the Study....43
7.3 Lost to Follow-up 43

8.0 STUDY ASSESSMENTS AND PROCEDURES 45
8.1 Efficacy Assessments 45
8.1.1 Echocardiography Central Laboratory 45
8.1.2 Patient Well-being....46
8.1.3 Efficacy-related Biomarkers....46
8.1.4 Exploratory Assessments of Efficacy....46
8.2 Safety Assessments....49
8.2.1 Physical Examinations....49
8.2.2 Vital Signs 49
8.2.3 Electrocardiograms....49
8.2.4 Clinical Safety Laboratory Assessments....49
8.3 Adverse Events....50
8.3.1 Time Period and Frequency for Collecting AE and SAE Information 50
8.3.2 Method of Detecting AEs and SAEs....51
8.3.3 Follow-up of AEs and SAEs 51
8.3.4 Regulatory Reporting Requirements for SAEs 51
8.3.5 Pregnancy 52
8.3.6 Adverse Events of Special Interest....52
8.4 Treatment of Overdose....52
8.5 Pharmacokinetics....52
8.6 Pharmacodynamics....53
8.7 Biomarkers 53
8.8 Genetics....53
8.9 Health Economics OR Medical Resource Utilization and Health Economics....53

9.0 STATISTICAL CONSIDERATIONS 53
9.1 Sample Size Determination....53
9.2 Populations for Analyses....54
9.3 Statistical Analyses....55
9.3.1 Efficacy Analyses....55
9.3.2 Safety Analyses 56
9.3.3 Missing Data....57
9.4 Interim Analyses 57
9.5 Data Safety Monitoring Board 57

10.0 REFERENCES....59

11.0 APPENDICES....61
Appendix 1 Abbreviations....62
Appendix 2 Regulatory, Ethical, and Study Oversight Considerations....64
Regulatory and Ethical Considerations....64
Financial Disclosure....64
Insurance....65
Informed Consent Process....65
Data Protection....65
Dissemination of Clinical Study Data....65
Data Quality Assurance....66
Source Documents....66
Study and Study Center Closure....67
Publication Policy....67
Appendix 3 Clinical Laboratory Tests....68
Appendix 4 Adverse Events: Definitions and Procedures for Recording, Evaluating, Follow-up, and Reporting....70
Appendix 5 Contraceptive Guidance and Collection of Pregnancy Information....75
Appendix 6 Signature of Investigator....78

TABLE OF TABLES

Table 1 Schedule of Activities..13
Table 2 Study Objectives and Endpoints..23
Table 3 Study Treatment Details 37
Table 4 Analysis Sets...55
Table 5 Protocol-required Safety Laboratory Assessments..68
Table 6 Highly Effective Contraceptive Methods 76

TABLE OF FIGURES

Figure 1 Study Schema..........12
Figure 2 CDR132L Pharmacokinetics in Human and Pig..........29
Figure 3 Circulating miR-132 Plasma Level in Human versus Tissue CDR132 Level in Pigs Both Normalized to Baseline Showing Highly Similar Time Course..........32

1.0 PROTOCOL SUMMARY

1.1 Synopsis

Protocol Title: P**h**ase 2, Multicenter, Randomized, Parallel, 3-arm, Placebo-controlled Study to Assess E**f**ficacy and Safety of CD**R**132L in Pati**e**nts with Reduced Left **V**entricular **E**jection F**r**ac**t**ion (≤ 45%) After Myocardial Infarction (**HF-REVERT**)

Short Title: P**h**ase 2 Study to Assess E**f**ficacy and Safety of CD**R**132L in Pati**e**nts with Reduced Left **V**entricular **E**jection F**r**ac**t**ion (≤ 45%) After Myocardial Infarction (**HF-REVERT**)

Rationale:

Several completed pre-clinical studies assessing the pharmacokinetics (PK), pharmacology, safety pharmacology, and toxicological profile of CDR132L have demonstrated that the safety profile is adequate to progress with clinical development. The non-clinical data supported the clinical drug regime in the recently-completed first-in-human (FIH) study in 28 patients with stable heart failure (HF) (New York Heart Association [NYHA] class I to III) of ischemic origin. This study determined the effects of [redacted] doses levels: [redacted] mg/kg of CDR132L administered intravenously (IV) on Days 1 and 28 as an add-on to Standard of Care (SoC). All dose levels were found to be safe and well tolerated with minimal related-treatment-emergent adverse events (TEAEs) and no clinically relevant changes in laboratory parameters, physical examination findings, vital signs, echocardiography (ECHO), or electrocardiogram (ECG). Pharmacokinetic analysis supported a good safety profile, with no accumulation, in line with a [redacted] half-life ($T_{1/2}$) of around [redacted], a restricted volume of distribution, and a high degree of dose linearity with regards to maximum plasma concentration (C_{max}). Dose-normalized area under the curve suggested a greater than dose-proportional increase in exposure with increasing dose. The Phase 1b FIH study therefore supports ongoing clinical assessment of CDR132L in the planned Phase 2, proof-of-concept study. [redacted]

This Phase 2 study aims to assess the efficacy and safety of CDR132L [redacted] mg/kg and [redacted] mg/kg, given as 3 single IV doses administered 28 days apart as an add-on to SoC therapy, in a larger cohort of patients (N = 280) with HF and reduced left ventricular ejection fraction (LVEF) (≤ 45%) after myocardial infarction (MI) with a follow-up of 10 months.

Objectives and Endpoints

Objectives	Endpoints
**Primary**	
• To assess the efficacy of [redacted] dose levels ([redacted] and [redacted] mg/kg) of CDR132L compared with placebo administered in 3 single IV doses given 28-days apart in patients with reduced LVEF ≤ 45% after MI (STEMI or NSTEMI) as add-on therapy to SoC treatment	• Percent change from baseline (screening to occur at least 3 days after MI diagnosis as measured by ECHO [central laboratory]) in LVESVI at Month 6

Objectives	Endpoints
**Secondary**	
• To assess the safety of [redacted] dose levels ([redacted] and [redacted] mg/kg) of CDR132L compared with placebo	• Frequency of adverse events and abnormalities in clinical laboratory assessments, vital signs, physical examination, ECGs, and urinalysis
• To assess the effects of CDR132L compared with placebo on cardiac function	• Change from baseline LVEF (absolute/relative) at Months 3, 6, and 12<br>• Change from baseline LVESVI at Month 3 (absolute/relative), Month 6 (absolute), and Month 12 (absolute/relative)
• To assess the effects of CDR132L compared with placebo on efficacy-related biomarkers	• Change from baseline in absolute/relative troponin T (ng/L) at Months 3, 6, and 12<br>• Change from baseline in absolute/relative values over time for the following efficacy-related biomarkers:<br>o N-terminal pro B-type natriuretic peptide (NT-proBNP)
• To assess the effects of CDR132L compared with placebo on patient well-being	• Well-being as evaluated by change from baseline at Months 6 and 12 in the following parameters:<br>o Mean KCCQ score and mean scores of subdomains (symptom burden, physical limitation, and quality of life)
**Exploratory**	
[redacted]	

Abbreviations: ECG = electrocardiogram; ECHO = echocardiography; HF = heart failure; IV = intravenously; KCCQ = Kansas City Cardiomyopathy Questionnaire; LVEF = left ventricular ejection fraction; LVESVI = left ventricular end-systolic volume index; MI = myocardial infarction; [redacted]; NSTEMI = non-ST-segment elevation myocardial infarction; [redacted]; SoC = Standard of Care; STEMI = ST-segment elevation myocardial infarction.

Overall Design:

This is a Phase 2, multicenter, randomized, parallel, 3-arm, placebo-controlled study to assess efficacy and safety of CDR132L in patients with reduced LVEF (≤ 45%) after MI. This study consists of a Screening Period (to occur at least 3 days after MI diagnosis), a 6-month Double-blind Period, and a 6-month Extension Period with the End of Study (EOS) Visit at Day 360/Month 12.

Patients will be screened to determine eligibility at least 3 days after MI diagnosis; all eligibility criteria must be confirmed no later than 14 days after MI diagnosis. Screening and dosing can be done in an inpatient (in case patients are hospitalized due to MI or HF) or outpatient setting. A total of approximately 280 unique individual patients will be randomly assigned to the 3 treatment groups in 1:1:1 ratio, with approximately 90 patients in each treatment group. Groups 1 and 2 will include patients who will receive CDR132L [redacted] mg/kg or [redacted] mg/kg IV, respectively. Patients in the placebo group (Group 3) are included as a comparator group for evaluation of efficacy and safety.

On Day 1 (preferably within one day after randomization but no later than 4 days after randomization), patients will receive CDR132L [redacted] mg/kg, CDR132L [redacted] mg/kg, or placebo IV. The second IV dose of the patient's assigned treatment will be administered on Day 29 ± 2 days, and the third IV dose on Day 57 ± 2 days. For doses administered in an outpatient setting, the patient should be observed for at least 30 minutes after dosing.

All patients will be required to attend the study visits as described in the Schedule of Activities. All patients will receive SoC therapy after MI and eventually for HF. CDR132L or placebo will be administered as add-on therapy to the SoC treatment. All patients will be followed for 10 months for efficacy and safety assessments. Overall, study duration for each patient will be approximately 12 months. A Data Safety Monitoring Board will closely supervise all data to monitor the safety of all patients.

Number of Investigators and Study Centers:

Approximately 60 Investigators and study centers are expected to participate in this study.

Number of Patients:

It is planned that approximately 280 patients will be enrolled/randomized into the study in a 1:1:1 ratio (approximately 90 patients each, in [redacted] mg/kg, [redacted] mg/kg, and placebo groups). Assuming 10 patients will not be evaluable, it is anticipated that 270 patients will be evaluable (90 patients each, in [redacted] mg/kg, [redacted] mg/kg, and placebo groups).

Treatment Groups and Duration:

Eligible patients will be randomized to receive CDR132L [redacted] mg/kg (Group 1), CDR132L [redacted] mg/kg (Group 2), or placebo (Group 3) IV; N = 90 patients per group. Patients will receive CDR132L [redacted] mg/kg, CDR132L [redacted] mg/kg, or placebo on Day 1. The second IV dose of the patient's assigned treatment will be administered on Day 29 ± 2 days, and the third IV dose on Day 57 ± 2 days.

Statistical Methods:

The primary endpoint is defined as percent (%) change from baseline left ventricular end-systolic volume index (LVESVI) at Month 6 compared with baseline (after reperfusion, before treatment start) on top of SoC as measured by ECHO (central laboratory).

For the sample size calculation, the two-sample t-test is used. The test level is set to 2.5% (one-sided). Assumptions are displayed below.

Left ventricular end-systolic volume index	Mean (change in %)	Standard deviation (%)
Placebo response	1	9
[redacted] mg/kg	6	9
[redacted] mg/kg	5	9
N = 90 per group	Step 1	96.0%
	Step 2	84.3%
	Overall	80.9%

Step 1 ([redacted] mg/kg versus placebo):

A sample size of 90 in each group will have 96.0% power to detect a difference in means of 5 (the difference between a Group 1 mean, μ_1, of 6 and a Group 2 mean, μ_2, of 1) assuming that the common standard deviation is 9 using a 2-group t-test with a 2.5% one-sided significance level.

Step 2 ([redacted] mg/kg versus placebo):

A sample size of 90 in each group will have 84.33% power to detect a difference in means of 4 (the difference between a Group 1 mean, μ_1, of 5 and a Group 2 mean, μ_2, of 1) assuming that the common standard deviation is 9 using a 2-group t-test with a 2.5% one-sided significance level.

Including 90 patients per group (270 total) will lead to a power of 96.0% for the first step ([redacted] mg/kg vs placebo, difference in means of 5) and 84.3% for the second step ([redacted] mg/kg vs placebo, difference in means of 4) of the hierarchical test procedure, resulting in an overall power of 80.9%. Ten additional patients should be included to compensate for early dropouts.

Data Safety Monitoring Board: Yes

1.2 Schema

Figure 1 Study Schema

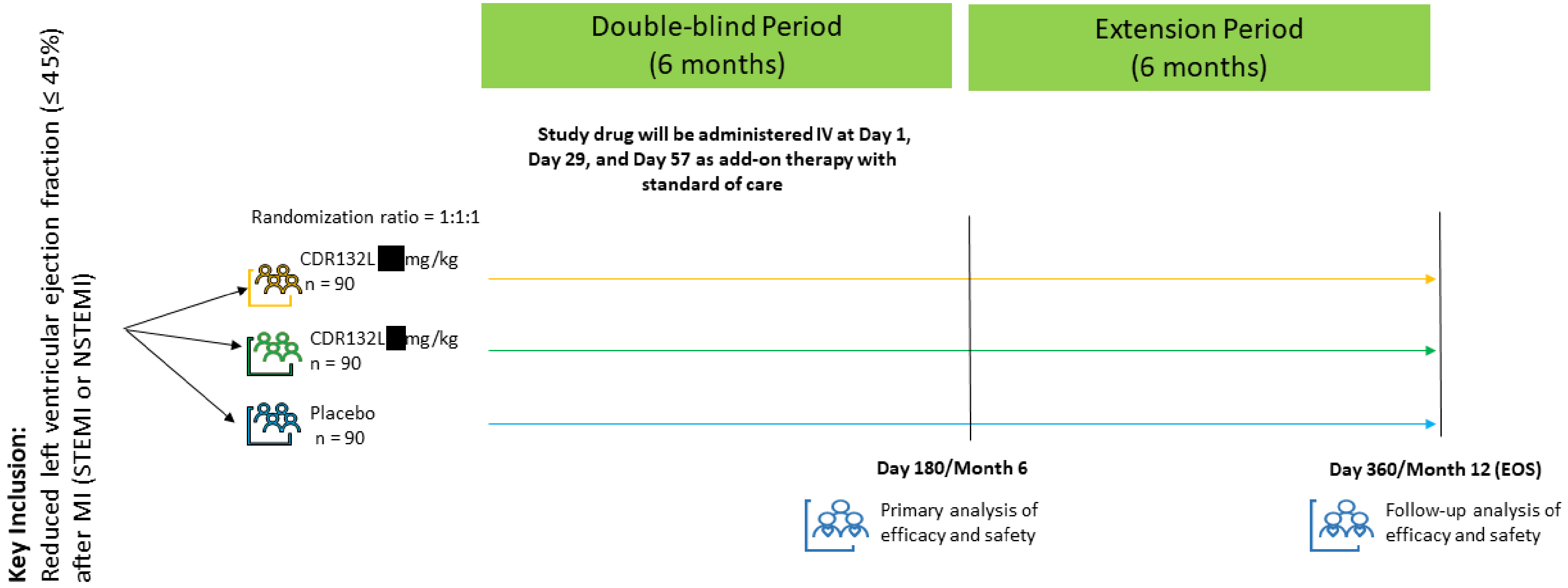

- ECHO = echocardiography; EOS = end of study; IV = intravenous; LVEF = left ventricular ejection fraction; MI = myocardial infarction; n = number of patients; NSTEMI = non-ST-segment elevation myocardial infarction; STEMI = ST-segment elevation myocardial infarction.

Note: To be eligible, patients must have LVEF ≤ 45% as measured by ECHO performed at least 3 days after diagnosis of MI (STEMI or NSTEMI). Eligible patients who receive study drug will also have an ECHO performed at Day 90/Month 3, Day 180/Month 6, and Day 360/Month 12.

1.3 Schedule of Activities

Table 1 Schedule of Activities

Procedures	Screening Period (To occur at least 3 days after MI diagnosis)	Double-blind Period (Day 1 to Day 180/Month 6)							Extension Period (6 months)
		Study Drug Treatment (Day 1, Day 29, and Day 57)					Follow-up		
**Visit**		**Randomization (To occur no later than 14 days after MI diagnosis)**	**1**	**2**	**3**	**4**	**5**	**6**	**EOS/ET**
**Study Day**			**Day 1**	**Days 2 to 5**	**Day 29**	**Day 57**	**Day 90**	**Day 180**	**Day 360**
**Window (days)**					**± 2**	**± 2**	**± 7**	**± 7**	**± 7**
**Month**					**Month 1**	**Month 2**	**Month 3**	**Month 6**	**Month 12**
Informed consent	X								
Demographic data and medical history	X								
Inclusion/exclusion criteria	X								
Randomization		X							
Study drug administration[a]			X[a]		X	X			
Prior/concomitant medications	X		X	X	X	X	X	X	X
**EFFICACY**									
Echocardiography[b]	X[b]						X	X	X
KCCQ	X						X	X	X
hs-cTn	X		X	X	X	X	X	X	X
NT-proBNP	X[c]		X	X	X	X	X	X	X
**SAFETY**									
Adverse event assessments	X		X	X	X	X	X	X	X
Injection site reaction monitoring			X		X	X			
Physical examination	X		X	X	X	X	X	X	X

**Procedures**	**Screening Period (To occur at least 3 days after MI diagnosis)**	**Double-blind Period (Day 1 to Day 180/Month 6)**							**Extension Period (6 months)**
		**Study Drug Treatment (Day 1, Day 29, and Day 57)**					**Follow-up**		
**Visit**		**Randomization (To occur no later than 14 days after MI diagnosis)**	**1**	**2**	**3**	**4**	**5**	**6**	**EOS/ET**
**Study Day**			**Day 1**	**Days 2 to 5**	**Day 29**	**Day 57**	**Day 90**	**Day 180**	**Day 360**
**Window (days)**					**± 2**	**± 2**	**± 7**	**± 7**	**± 7**
**Month**					**Month 1**	**Month 2**	**Month 3**	**Month 6**	**Month 12**
Vital signs	X		X	X	X	X	X	X	X
12-lead electrocardiogram	X		X		X	X		X	X
Body weight	X			X	X		X	X	X
Hematology, biochemistry, coagulation[d]	X		X	X	X	X	X	X	
Urinalysis[d]	X		X		X		X	X	X
**EXPLORATORY EFFICACY**									

[redacted]

Abbreviations: ECHO = echocardiography; ED = emergency department; EOS = end of study; ET = early termination; hs-cTn=high-sensitivity cardiac troponin; IV = intravenous; KCCQ = Kansas City Cardiomyopathy Questionnaire; NT-proBNP = N-terminal pro B-type natriuretic peptide; [redacted]; MI = myocardial infarction; SoC = Standard of Care.

Note: All blood samples should be collected pre-dose at dosing days.

a. On Day 1 (preferably within one day after randomization but no later than 4 days after randomization) patients will receive CDR132L 5 mg/kg, CDR132L 10 mg/kg, or placebo IV. The second IV dose of the patient's assigned treatment will be administered on Day 29 ± 2 days, and the third IV dose on Day 57 ± 2 days. For doses administered in an outpatient setting, the patient should be observed for at least 30 minutes after dosing.
b. The screening ECHO will be performed at least 3 days after MI diagnosis; all eligibility criteria must be confirmed no later than 14 days after MI diagnosis.
c. At screening, sample is to be collected as early as possible preferably on the same day as the ECHO. NT-proBNP values required for eligibility confirmation may be collected at any time post MI either through medical history (e.g., site has collected it as SoC once the patient came to the hospital), through a local laboratory assessment, or by sending a sample to the central laboratory (if sites use the SoC sample or the local laboratory sample for eligibility confirmation, no additional sample for central laboratory assessment is needed).
d. See Appendix 3 for more information.

2.0 INTRODUCTION

CDR132L is a synthetic antisense oligonucleotide (ASO) and a selective inhibitor of microRNA-132-3p (miR-132). miR-132 in cardiomyocytes is a central switch affecting the expression of genes that are crucially involved in maladaptive cardiac remodeling, transformation, and pathological cardiac growth (hypertrophy), contributing to adverse cardiac remodeling and heart failure (HF).[1-5] Aberrant expression of miR-132 in cardiac cells is causally associated with cardiac remodeling and HF progression.[1-6]

The characteristic common pathological feature in HF after myocardial infarction (MI) is adverse structural remodeling of the left ventricle. Dysfunctional left ventricular remodeling that occurs in response to mechanical stress and neurohormonal activation after an MI was initially considered irreversible, with only a decrease in heart size possible by therapies such as angiotensin-converting enzyme inhibitors, beta-blockers, and cardiac synchronization therapy. However, reverse remodeling, a process by which the failing myocardium normalizes chamber geometry and function with correction of molecular and transcriptional abnormalities, is now being recognised.[7] Blocking aberrant miR-132 expression by the selective ASO inhibitor CDR132L is suited to reverse MI-related adverse cardiac effects and restore normal cellular function in cardiomyocytes, contributing to improved cardiac function in stable ischemic HF patients. [2,3,8]

The global burden of HF is significant, with an estimated prevalence of 64.34 million cases contributing to 9.91 million years lost to disability.[9] Heart failure complicating acute MI is common and is a powerful predictor of death.[10] The incidence of HF among patients hospitalized for MI varies between 14% and 36%.[11,12] Inhibition of miR-132 effectively prevents progression of HF in an animal model of the disease,[13] and translational in vitro and in vivo studies have demonstrated the safety, tolerability, and efficacy of CDR132L in HF. [3,2] In addition, a recent first-in-human (FIH) study exploring a range of doses (0.32 to 10 mg/kg) reported CDR132L to be safe and well tolerated in patients with stable ischemic HF as an add-on therapy to Standard of Care (SoC), and supports the progression to the next stage of clinical development.[4]

2.1 Study Rationale

Several completed pre-clinical studies assessing the pharmacokinetics (PK), pharmacology, safety pharmacology, and toxicological profile of CDR132L have demonstrated that the safety profile is adequate to progress with clinical development. The non-clinical data supported the clinical drug regimen in the recently-completed FIH study in 28 patients with stable HF (New York Heart Association [NYHA] class I to III) of ischemic origin.[4] This study determined the effects of [redacted] dose levels: [redacted] mg/kg of CDR132L administered intravenously (IV) on Days 1 and 28 as an add-on to SoC. All dose levels were found to be safe and well tolerated with minimal treatment-related treatment-emergent adverse events (TEAEs) and no clinically

relevant changes in laboratory parameters, physical examination findings, vital signs, echocardiography (ECHO), or electrocardiogram (ECG). Pharmacokinetic analysis supported a good safety profile, with no accumulation, in line with a [redacted] half-life ($T_{1/2}$) of around [redacted], a restricted volume of distribution, and a high degree of dose linearity with regards to maximum plasma concentration (C_{max}). Dose-normalized area under the curve suggested a greater than dose-proportional increase in exposure with increasing dose. The Phase 1b FIH study therefore supports ongoing clinical assessment of CDR132L in the planned Phase 2, proof-of-concept study. [redacted].

This Phase 2 study aims to assess the efficacy and safety of CDR132L [redacted] mg/kg and [redacted] mg/kg, given as 3 single IV doses administered 28 days apart as an add-on to SoC therapy, in a larger cohort of patients (N = 280) with HF and reduced LVEF (≤ 45%) after MI with a follow-up of 10 months.

2.2 Background

Current state-of-the-art HF pharmacotherapy is largely focused on symptomatic management; that is, mainly on reducing cardiac load by reducing neurohormonal overdrive and volume retention. The recommended guideline-directed medical therapy (GDMT) includes diuretics, angiotensin-converting enzyme inhibitors, angiotensin II receptor blockers, beta-blockers, aldosterone antagonists, angiotensin receptor neprilysin inhibitors, and ivabradine. Mechanical circulatory support and heart transplant is reserved for the treatment of patients with severe HF with reduced ejection fraction (EF) who have failed GDMT.[14] Novel, efficient, disease-halting therapeutics that reduce mortality and hospitalization are urgently needed to offer curative hope for these patients. CDR132L is a unique miRNA-based next generation drug in HF, with the potential to improve patient care and reduce the financial burden of HF care.

The mechanism of action (MoA) of CDR132L has the following key elements forming the basis of its role as a novel drug in HF: a) normalization of aberrant cardiac miR-132 levels, b) normalization of calcium signaling and contractility as well as cardiac function, c) improvement in cardiac autophagy and homeostasis, and d) attenuation of maladaptive cardiac remodeling (reducing pathological cardiomyocyte growth and cardiac fibrosis).[1-5]

2.2.1 Overview of Non-clinical Studies

CDR132L has undergone an extensive non-clinical safety evaluation including safety pharmacology and repeat-dose toxicity studies. Additional safety information is available from a large proof-of-concept study in Mangalica pigs in a clinically relevant model of post MI HF.

CDR132L was well tolerated at [redacted] mg/kg in the proof-of concept study in Mangalica pigs. No acute effects on the central nervous system (CNS), CV, and respiratory system have been observed in Good Laboratory Practices (GLP)-compliant safety pharmacology studies in

rats and minipigs. In a GLP-compliant 4-week toxicity study in rats, the animals received 2 slow intravenous bolus injections of 0 (vehicle), [redacted] mg/kg CDR132L on Days 1 and 28. Additional animals from the control and high-dose group were subjected to a 4-week recovery period after completion of the treatment period. In the main groups, there were slightly decreased red blood cell (decreased hemoglobin, mean corpuscular volume, mean corpuscular hemoglobin) and coagulation parameters (decreased prothrombin time and partial thromboplastin time) at [redacted] mg/kg CDR132L, which may be treatment-related. Mildly but significantly increased alanine aminotransferase (ALT) and aspartate aminotransferase levels at [redacted] mg/kg may suggest an adverse effect of CDR132L on the liver, however, there was no morphological correlate, and the effects reversed during a 4-week recovery period. Slightly increased creatinine values indicating impairment of renal function were found in the [redacted] mg/kg dose group. Macroscopic findings were noted in the kidney in animals from the [redacted] mg/kg dose group. The kidneys were slightly paler compared with kidneys from control animals showing a light brown parenchyma. Relative kidney weights were increased at the end of the treatment period in female animals and at the end of the recovery period in male animals treated with [redacted] mg/kg. Relative liver weights were increased at the end of the recovery period in animals treated with [redacted] mg/kg. Increased kidney and liver weights likely represent the presence of high concentrations of the test item and the water that is required to keep it hydrated. Histological examination revealed treatment-related findings in the kidney in the form of basophilic granules in the cytoplasm of the tubular epithelium, degeneration of the tubular epithelium, dilation of tubules, and regeneration of tubules. The basophilic granules within the cytoplasm of the tubular epithelium were dose-dependent and occurred at [redacted] and [redacted] mg/kg and were still observable in the high-dose animals at the end of the recovery period. Very slight to slight degeneration of the tubular epithelium, very slight dilation and regeneration of tubules were only detected in the high-dose group. The incidence of very slight to slight degeneration of the tubular epithelium was interpreted as adverse. The very slight dilation and regeneration of tubules were considered to be the consequence of the degenerative effect on the tubular epithelium observed. The no-observed-adverse-effect-level (NOAEL) in this study was considered to be [redacted] mg/kg, due to effects observed in clinical chemistry and histopathological effects on the kidneys at [redacted] mg/kg.

In a GLP-compliant 6-month toxicity study in rats, the animals received 6 slow IV bolus injections of 0 (vehicle), [redacted] mg/kg CDR132L every [redacted]. Additional animals from the control and high-dose group were maintained for a 3-month recovery period after completion of the treatment period. Six animals from the high-dose group ([redacted] mg/kg) were found dead or were killed moribund in the course of the study. Body weights were significantly lower in animals from the high-dose group and bod weight gains were reduced in males from the high-dose group. Food consumption was decreased in males and females from the high-dose group. Reduced red blood cell counts observed in males and females at [redacted] mg/kg were suggestive of anemia. Increased laboratory parameters reflecting renal function i.e., creatinine and urea, plasma electrolytes, indicated a treatment-related effect on the kidney. Pronounced

hemolysis, which was observed primarily in the males from the high-dose group, was considered to be secondary to the renal impairment, as increased urea levels lead to hemolysis during the cause of renal anemia. In addition, hyperkalemia, hyperphosphatemia, hypocalcemia, decreased, albumin, increased globulin as well as strongly increased cholesterol and triglyceride levels were observed, which were considered secondary to the kidney effects. Quantitative urinalysis revealed decreased osmolality in the high-dose group and semiquantitative urinalysis results revealed increased leukocytes and urinary blood in rats of either sex, which again suggested a treatment-related effect on the kidney. The thymus was slightly to moderately smaller in some females from the high-dose group. The kidneys were slightly to moderately enlarged in animals from the high-dose group. Histopathologically, treatment-related effects were observed in the kidney, liver, and bone marrow. Kidney findings consisted of basophilic granules within the cytoplasm of the tubular epithelium, which were dose-dependent and occurred in male and female animals from the low-, mid-, and high-dose and were still observable in male and female animals from the high-dose recovery group. In the absence of any degenerative findings, basophilic granules in the kidneys were regarded as non-adverse. Findings of degeneration of the tubular epithelium, increased infiltration of mixed inflammatory cells and interstitial fibrosis, which were significantly increased in males and partially in females from the high-dose main study group and in male and female animals from the high-dose recovery group, were interpreted as adverse. Dilatation of tubules, intraluminal hyaline casts in the tubules and regeneration of tubules were interpreted to be the consequence of the degeneration of the tubular epithelium. These changes were significantly increased in the male and female animals from the high-dose main study and recovery groups. The findings of the kidneys in the animals from the high-dose group progressed during the recovery period developing into more severe changes. Treatment-related finding within the liver consisted of basophilic granules within cellular cytoplasm. This finding was regarded as an adaptive change. Treatment-related finding within the bone marrow consisted of decreased erythroid cellularity in few animals. This change was interpreted to be secondary to the kidney changes most likely due to decreased erythropoietin production. The NOAEL in this study was considered to be the mid-dose ([redacted] mg/kg).

In a GLP-compliant 4-week toxicity study in minipigs, the animals received 0 (vehicle), [redacted] mg/kg CDR132L on Days [redacted] by a 15-minute IV infusion. No signs of toxicity were observed in this study and thus the NOAEL was determined to be [redacted] mg/kg, the highest dose tested. Adequate systemic exposure to CDR132L was confirmed by toxicokinetic assessments.

In a GLP-compliant 9-month toxicity study in minipigs, the animals received nine 15-minute IV infusions at dose levels of 0 (vehicle) [redacted] mg/kg every [redacted] weeks. Additional animals from the control and high-dose group were maintained for a 3-month recovery period after completion of the treatment period. The following findings were noted:

- At macroscopic examination, enlarged and discolored kidneys were observed in 1 out of 4 male and 2 out of 4 female animals treated with [redacted] mg/kg CDR132L. Histopathologically,

there were test item-related microscopic changes in the kidneys in the form of basophilic epithelial swelling, tubular dilatation, tubular hyaline casts, tubular neutrophilic infiltration, tubular fatty infiltration, and in in cervical and mesenteric lymph nodes (foamy macrophage) in male and female animals treated with [redacted] mg/kg.

- Microscopic changes were also noted in the testes in the form of increased severity of tubular atrophy, correlating with the decreased relative and absolute testes weights, and epididymides in the form of oligospermia, immature germ cells, sperm granuloma and/or aspermia in male animals treated with [redacted] mg/kg.
- Histopathological examination of recovery animals previously treated with [redacted] mg/kg still revealed microscopic changes in the kidneys in the form of minimal tubular epithelial basophilic swelling, in the cervical and mesenteric lymph nodes in the form of foamy macrophages, in the testes and the epididymides in the form of tubular atrophy and the presence of immature germ cells.

However, a trend towards full recovery was noted for the findings observed in the kidneys, epididymides, and testes at the end of the treatment period as a clear decrease was noted for both the incidence and the severity of the changes. A trend towards recovery was also noted for the microscopic changes in the cervical and mesenteric lymph nodes as the severity of the finding declined compared with the main study animals. At [redacted] mg/kg, microscopic changes were confined to mild to moderate tubular basophilic epithelial swelling in the kidneys and foamy macrophages in the cervical and mesenteric lymph nodes, which, in the absence of any degenerative findings, were regarded as non-adverse. The changes in the kidney and lymph nodes observed at [redacted] mg/kg CDR132L are likely attributable to the presence of the test item as described in the literature for minipigs and other species following treatment with antisense oligonucleotides.[15,16] Microscopic findings noted in the testes in the form of tubular atrophy at [redacted] mg/kg CDR132L and correlating findings in the epididymides are known to be common spontaneous findings in Göttingen minipigs which are often accompanied by decreased testes weights. For example, it has been reported that tubular atrophy is observed in more than 70% of naïve Göttingen minipigs with similar findings observed in the epididymides in more than 20% of naïve Göttingen minipigs.[17]

Although the number of animals examined at the end of the treatment period was low (4 males per group), which hampers the interpretation of the findings, the severity of the testes atrophy and the incidence and severity of the accompanying changes in the epididymides at [redacted] and [redacted] mg/kg CDR132L revealed a slight dose-dependency which correlated with decreased testes weights. This suggested a relationship to treatment with CDR132L. However, the focal or multifocal distribution of the tubular atrophy, as well as the absence of microscopic changes that may indicate a test item induced testicular toxicity, such as degenerate or apoptotic germ cells, multinucleated giant cells or an adaptive hyperplasia of Leydig cells, may also suggest that the findings are incidental. Due to the potential reversibility of the observed testes atrophy, the findings are regarded as non-adverse. Due to the absence of degenerative findings in the kidneys

and lymph nodes, the minimally increased severity of potentially reversible testes findings, and the minimally increased number and severity of accompanying findings in the epididymides at [redacted] mg/kg, the NOAEL was assigned to [redacted] mg/kg.

2.2.2 Overview of Clinical Studies

The FIH study explored 4 dose levels that ranged from [redacted] mg/kg. All doses were found to be safe and well tolerated. There were no deaths or serious adverse events (SAEs) during the study, and no TEAE led to treatment or study discontinuation. A total of 53 TEAEs were reported in 22 patients (78.6% of all) including placebo-treated patients. Of the 53 TEAEs, 4 were considered related to CDR132L. All TEAEs were mild in severity and all resolved. According to adverse reaction rules, no safety limits were exceeded across all cohorts of the study. The most common CDR132L-related TEAE was dizziness, with 2 patients reporting 1 event each. The frequency of TEAEs did not increase with dose escalation, so that no dose-response relationship was demonstrated. The TEAEs reported were consistent with the underlying patient population and their concomitant medication. There were no adverse events of special interest (AESIs) (i.e., thrombocytopenia) and even though there was 1 episode of paravasal infusion, no injection site reactions have been recorded in this study. No telemetry, Holter ECG, or ECG parameter (blood pressure [BP] and heart rate) were considered to represent clinically significant changes from baseline at the time of recording by the reviewing cardiologist. There were no notable, treatment-related, or clinically significant changes in liver function, renal function, or other laboratory results (N-terminal pro B-type natriuretic peptide [NT-proBNP] or high-sensitivity cardiac troponin [hs-cTn]), vital signs (BP and heart rate), or physical examinations from baseline in any CDR132L group or placebo. Although this study was designed primarily to assess safety and PK of 4 ascending dosages of CDR132L, exploratory analysis of pre-planned PD endpoints revealed positive and encouraging effects demonstrating the translation of findings from various animal HF models into HF patients.

This proof-of-concept Phase 2 study will evaluate the efficacy of CDR132L in improving cardiac function and safety in patients with reduced left ventricular ejection fraction (LVEF) for 12 months post MI. Though safety has been proven in the pre-clinical studies and in a FIH study, the safety needs to be confirmed in a larger cohort of patients, which will be further assessed in this study.

2.3 Benefit/Risk Assessment

Heart failure is a serious condition with high mortality and frequent episodes of worsening that often requires hospitalization or emergency department (ED) care. CDR132L offers a novel therapeutic option in HF, by reversing the adverse cardiac remodeling and improving cardiac function. CDR132L is a synthetic and chemically modified ASO that selectively inhibits miR-132. A considerable number of ASOs containing identical or similar chemical modifications have been tested in clinical studies and some ASOs have been approved as medicinal products in

the European Union (EU) and the United States (US), e.g., Spinraza (nusinersen), Tegsedi (inotersen). Miravirsen, another PS-LNA modified ASO targeting the liver expressed miRNA-122 is currently in Phase 2 clinical study for treatment of Hepatitis C virus infection.[18]

The favorable PK, safety, tolerability, dose-dependent PK/PD relationship and high clinical potential for CDR132L has been demonstrated in vitro as well as in vivo in rats and in a highly clinically relevant pig model of HF. No potential CNS effects of CDR132L were reported in a 4-week toxicity study in rats. The potential cardiovascular (CV) effects were determined by telemetry in minipigs and demonstrated no deaths, no signs of local intolerance at infusion sites, and no signs of systemic toxicity at any dose level. Additionally, no influence was noted on physical activity, BP, heart rate, or ECG parameters. Similarly, exposure of CDR132L in rats had no demonstrable adverse effects on lung function parameters. The FIH study explored 4 dose levels that ranged from [redacted] mg/kg. All doses were found to be safe and well tolerated. There were no deaths or SAEs during the study, and no TEAE led to treatment or study discontinuation.

Potential adverse effects of CDR132L may relate to "on-target" (sequence-specific miR-132 inhibition) and "off-target" (sequence unspecific, class-effects of chemically modified oligonucleotides) effects. The safety profile of CDR132L has been evaluated in GLP-compliant safety pharmacology and repeat-dose toxicity studies using up to 9 IV applications of CDR132L given each 4 weeks apart, which covered a significantly longer exposure, than the 3 administrations planned in this Phase 2 study. There is clinical information from only 1 FIH study to predict adverse effects beyond those observed in safety pharmacology and toxicity studies. However, there is comprehensive clinical experience with other locked nucleic acid-phosphorothioate oligonucleotides and these data suggest a low risk of class-specific side effects and/or off-target effects with these types of compounds. Since adverse effects on the liver and kidneys may be anticipated from findings in the repeat-dose toxicity study at high dose tested ([redacted] mg/kg), liver and kidney values will be closely monitored during the clinical study.

Although not observed in the toxicity studies in rats and minipigs, special attention will be paid to potential side effects of CDR132L due to the abundant expression of miR-132 in healthy tissues, particularly in the CNS which shows the highest expression of miR-132. Due to its relatively high molecular weight, CDR132L is too large to cross the blood-brain-barrier by diffusion and reach an effective concentration in the CNS.[19] This has been confirmed in a tissue distribution study in healthy pigs and our GLP-compliant 9-month toxicity study in minipigs with monthly administrations. Therefore, the risk of neurological side effects due to inhibition of miR-132 in the CNS is very low. Severe thrombocytopenia has recently been reported in some clinical studies with ASO compounds.[20] However, there was no indication of reduced platelet counts in the repeat-dose toxicity studies or in the FIH study with CDR132L.

More detailed information about the known and expected benefits and risks and reasonably expected adverse events (AEs) of CDR132L may be found in the Investigator's Brochure (IB).[8]

3.0 OBJECTIVES AND ENDPOINTS

This is a Phase 2, multicenter, randomized, parallel, 3-arm, placebo-controlled study to assess the efficacy and safety of CDR132L in patients with reduced LVEF (≤ 45%) after MI. The objectives and endpoints are outlined in Table 2.

Table 2 Study Objectives and Endpoints

**Objectives**	**Endpoints**
**Primary**	
• To assess the efficacy of [redacted] dose levels ([redacted] and [redacted] mg/kg) of CDR132L compared with placebo administered in 3 single IV doses given 28-days apart in patients with reduced LVEF ≤ 45% after MI (STEMI or NSTEMI) as add-on therapy to SoC treatment	• Percent change from baseline in LVESVI (screening to occur at least 3 days after MI diagnosis as measured by ECHO [central laboratory]) at Month 6
**Secondary**	
• To assess the safety of [redacted] dose levels ([redacted] and [redacted] mg/kg) of CDR132L compared with placebo	• Frequency of adverse events and abnormalities in clinical laboratory assessments, vital signs, physical examination, ECGs, and urinalysis
• To assess the effects of CDR132L compared with placebo on cardiac function	• Change from baseline LVEF (absolute/relative) at Months 3, 6, and 12<br>• Change from baseline LVESVI at Month 3 (absolute/relative), Month 6 (absolute), and Month 12 (absolute/relative)
• To assess the effects of CDR132L compared with placebo on efficacy-related biomarkers	• Change from baseline in absolute/relative troponin T (ng/L) at Months 3, 6, and 12<br>• Change from baseline in absolute/relative values over time for the following efficacy-related biomarkers:<br>o N-terminal pro B-type natriuretic peptide (NT-proBNP)
• To assess the effects of CDR132L compared with placebo on patient well-being	• Well-being as evaluated by change from baseline at Months 6 and 12 in the following parameters:<br>o Mean KCCQ score and mean scores of subdomains (symptom burden, physical limitation, and quality of life)
**Exploratory**	
[redacted]	

Objectives	Endpoints
[redacted]	

Abbreviations: ECG = electrocardiogram; ECHO = echocardiography; HF = heart failure; IV = intravenously; KCCQ = Kansas City Cardiomyopathy Questionnaire; LVEF = left ventricular ejection fraction; LVESVI = left ventricular end-systolic volume index; MI = myocardial infarction; [redacted]; NSTEMI = non-ST-segment elevation myocardial infarction; [redacted]; SoC = Standard of Care; STEMI = ST-segment elevation myocardial infarction.

4.0 STUDY DESIGN

4.1 Overall Design

This is a Phase 2, multicenter, randomized, parallel, 3-arm, placebo-controlled study to assess efficacy and safety of CDR132L in patients with reduced LVEF (≤ 45%) after MI. As shown in Figure 1, this study consists of a Screening Period (to occur at least 3 days after MI diagnosis), a 6-month Double-blind Period, and a 6-month Extension Period with the End of Study (EOS) Visit at Day 360/Month 12.

Patients will be screened to determine eligibility at least 3 days after MI diagnosis; all eligibility criteria must be confirmed no later than 14 days after MI diagnosis. Screening and dosing can be done in an inpatient (in case patients are hospitalized due to MI or HF) or outpatient setting. A total of approximately 280 unique individual patients will be randomly assigned to the 3 treatment groups in 1:1:1 ratio, with approximately 90 patients in each treatment group. Groups 1 and 2 will include patients who will receive CDR132L [redacted] mg/kg or [redacted] mg/kg, respectively. Patients in the placebo group (Group 3) are included as a comparator group for evaluation of efficacy and safety.

On Day 1 (preferably within one day after randomization but no later than 4 days after randomization), patients will receive CDR132L [redacted] mg/kg, CDR132L [redacted] mg/kg, or placebo IV. The second IV dose of the patient's assigned treatment will be administered on Day 29 ± 2 days, and the third IV dose on Day 57 ± 2 days. For doses administered in an outpatient setting, the patient should be observed for at least 30 minutes after dosing.

All patients will be required to attend the study visits as described in the Schedule of Activities (SoA; Section 1.3). All patients will receive SoC therapy post MI and eventually for HF. CDR132L or placebo will be administered as add-on therapy to the SoC treatment. All patients will be followed for 10 months for efficacy and safety assessments. Overall, study duration for each patient will be approximately 12 months.

A Data Safety Monitoring Board (DSMB) will closely supervise all data to monitor the safety of all patients. The DSMB Charter will describe the membership, roles, responsibilities, and operating guidelines of the DSMB.

Approximately 60 European study centers will be selected according to their expertise in performing complex, early phase clinical studies as well as their ability to follow guidelines on established SoC treatment of post MI and HF patients. The study centers must have sufficiently trained study personnel to guarantee adherence to this study protocol.

4.2 Scientific Rationale for Study Design

This study will assess the efficacy and safety of CDR132L in patients with reduced LVEF (≤ 45%) after MI. Patients will be administered either [redacted] mg/kg or [redacted] mg/kg of CDR132L or

placebo on Days 1, 29 ± 2 days, and 57 ± 2 days. Patients who will receive placebo will constitute the control group that will help to determine which effects will pertain to the study drug (CDR132L) and which will not. Patients in this study will be assigned to a treatment regimen according to a randomization schedule and will enter the Double-blind Period whereby patients and clinical study site staff will be blinded to the active or placebo study treatment. CDR132L and placebo will be identical in appearance. In this study, patients who are prematurely discontinued will still be followed further for efficacy and safety assessments.

4.2.1 Rationale for Patient Selection

Due to its unique MoA, the main effect of CDR132L is to halt and reverse pathological cardiac ventricular remodeling. Adverse remodeling is the hallmark of post MI HF development irrespective of the clinical presentation of MI. In clinical practice, a combination of criteria is required to meet the diagnosis of acute myocardial infarction (AMI), which includes the detection of an increase of the cardiac biomarker troponin (preferably hs-cTn) T or I, and at least one of the following:[21]

- Symptoms of myocardial ischemia.
- New ischemic ECG changes.
- Development of pathological Q waves on ECG.
- Imaging evidence of loss of viable myocardium or new regional wall motion abnormality in a pattern consistent with an ischemic etiology.

Based on ECG evaluation, most patients will ultimately develop ST-segment elevation myocardial infarction (STEMI), but some patients will exhibit acute chest discomfort without persistent ST-segment elevation (non-ST-segment elevation myocardial infarction [NSTEMI]; including transient ST-segment elevation, persistent or transient ST-segment depression, T-wave inversion, flat T waves, or pseudo-normalization of T waves; or the ECG may be normal).

The proportion of patients with NSTEMI increased from approximately 30% in 1995 to approximately > 50% in 2015, mainly accounted for by a refinement in the operational diagnosis of NSTEMI.[22]

For both STEMI and NSTEMI patients, the pathological correlate at the myocardial level is cardiomyocyte necrosis in the infarcted area as indicated by the increase in hs-cTn T or I levels. The ensuing changes after tissue necrosis involve complex interactions in the myocardium between the cellular and extracellular components, under neurohormonal regulation. Due to increased load on the non-infarcted myocardium, the process of cardiac remodeling including pathological growth, impaired contractility, and increases in cardiac fibrosis is initiated.

We have shown that miR-132 induces all aforementioned hallmarks of cardiac remodeling. More importantly, this goes in hand with increased cardiac and plasma miR-132 levels in patients with

various forms of HF. Finally, cardiac remodeling processes lead to progressive left ventricle (LV) dilatation (measured by increased LV end-systolic and end-diastolic volumes).

In ischemic as well as non-ischemic HF models, the beneficial anti-remodeling effects of CDR132L treatment are postulated to be attributed to its anti-hypertrophic, pro-contractile, and anti-fibrotic effects. The miR-132, which is the direct target of CDR132L, is a central switch in the molecular mechanism of HF progression as evidenced by genetic and pharmacological animal data. CDR132L blocks aberrant cardiac miR-132 levels, and thereby:

- normalizes aberrant functional miR-132 levels in the myocardium,[1-3]
- normalizes calcium signaling and contractility,[2,3]
- reduces pro-hypertrophic nuclear factor of activated T cells (NFAT) signaling leading to lower B-type natriuretic peptide levels,[1-4] and
- attenuates maladaptive cardiac remodeling by additional anti-fibrotic effects.[1-4]

The results in relevant pre-clinical studies in post MI HF provide clear evidence that CDR132L treatment provides persistent and clinically relevant therapeutic effects on cardiac function in both STEMI and NSTEMI patients given that:

- acute ischemia is not the only trigger for increased cardiac miR-132 expression and
- the anti-remodeling MoA of CDR132L is expected to positively benefit both ischemic and non-ischemic HF conditions (as outlined above).

In summary, due to CDR132L's specific MoA, both patients with either STEMI or NSTEMI will be included in this study.

4.3 Justification for Dose

4.3.1 Justification for Selection of Planned CDR132L Doses

Justification for the selected CDR132L doses ([redacted] mg/kg and [redacted] mg/kg) in the present Phase 2 study considers the results from the following: non-clinical safety, tolerability, PK, and pharmacodynamic studies, the toxicology studies in rats and minipigs (see the IB[8]), as well as the Phase 1b study in otherwise healthy HF patients. The approach was further guided by an elaborate PK/PD modeling of the anticipated CDR132L exposures in humans. Based on the recommendation by the regulatory authority, Medicines Evaluation Board of the Netherlands, on the selection of an appropriate time window for the safety follow-up in the Phase 2 study, an additional PD modeling was performed to project the changes in the miR-132 target plasma levels over time in patient cohorts of the Phase 1b study beyond the study endpoint at Day 112. The aim of the clinical Phase 2 study dosing strategy is to demonstrate efficacy and successful target engagement.

Dose Selection

Since in the Phase 1b study, no dose-limiting toxicity was observed in human subjects up to a dose of [redacted] mg/kg in a once per month administration scheme, this dose was considered safe for the planned Phase 2 study (CDR132L-P2-01), which comprises the same administration regimen with regard to the dosing intervals.

Pre-clinical assessment of CDR132L in GLP-like studies such as early post MI ([redacted])[2] and chronic post MI HF pig model ([redacted])[3] including more than 180 pigs demonstrated clinically relevant improvement in cardiac function, thereby reversing pathological cardiac remodeling, inhibition of cardiac fibrosis, and pathological cardiomyocyte growth post MI. Simultaneously, CDR132L doses of [redacted] mg/kg and [redacted] mg/kg also improved systolic and diastolic function applying.

It was demonstrated that CDR132L tissue concentrations positively correlated with improvement of cardiac function, as measured by delta LVEF (change in LVEF between study endpoint and the day of first administration) in both early post MI and chronic HF pig models. Additionally, CDR132L levels correlated with the reduction of functional tissue levels of its target gene, miR-132, in a linear manner. Importantly, CDR132L administrations reduced circulating miR-132 in plasma in early post MI (data available up to 6 months of treatment; [redacted]) and in chronic post MI HF pig model ([redacted]).

In addition, assessment of CDR132L PK characteristics in humans, compared with the large animal pig model, confirmed a high level of inter-species translatability (see Figure 2). The CDR132L PK profiles determined in humans and pigs show inter-species consistency with no signs for drug accumulation. The PK profiles allow translation from pigs to humans with allometric scaling factor of 1.02 to 1.24 (of note, 1.1 was assumed before study start of the Phase 1b and is noted by the Food and Drug Administration for selection of a starting dose in first-in-human [FIH] studies). CDR132L shows only a slightly prolonged initial half-life in human when considering a bi-phasic kinetic of the compound (1 h at [redacted] mg/kg to 2.49 h at [redacted] mg/kg).

Figure 2 CDR132L Pharmacokinetics in Human and Pig

Human PK ([redacted])

	Cohort 1 (CDR132L, 0.32 mg/kg)	Cohort 2 (CDR132L, 1 mg/kg)	Cohort 3 (CDR132L, 3 mg/kg)	Cohort 4 (CDR132L, 10 mg/kg)
Day 1				
C_{max} (ng/mL)	4357.075 (473.306)	13209.16 (1457.641)	45535.94 (7286.316)	126963.14 (46410.337)
t_{max} (h)	0.404 (0.008)	0.403 (0.007)	0.407 (0.015)	0.57 (0.38)
$t_{½}$ (h)	1.038 (0.069)	5.207 (4.474)	4.244 (0.239)	4.346 (0.229)
λ_z (1/h)	0.67 (0.044)	0.198 (0.107)	0.164 (0.009)	0.16 (0.008)
$AUC_{0\text{-}inf}$ (h*ng/mL)	5403.536 (741.727)	25289.533 (5889.116)	116628.451 (32046.812)	424609.461 (71319.249)
V_z (L)	7.054 (1.267)	24.166 (21.972)	14.201 (2.979)	11.753 (1.316)
CL (L/h)	4.724 (0.925)	3.217 (0.725)	2.334 (0.558)	1.875 (0.2)
Day 28				
Cmax (ng/mL)	4257.68 (726.876)	12386.86 (1942.225)	43398.65 (15517.698)	127426.36 (35483.565)
tmax (h)	0.4 (0)	0.403 (0.007)	0.508 (0.142)	0.4 (0)
t½ (h)	1.056 (0.056)	5.095 (5.15)	4.594 (0.344)	4.57 (0.178)
λz (1/h)	0.658 (0.037)	0.223 (0.116)	0.152 (0.011)	0.152 (0.006)
AUC0-inf (h*ng/mL)	5280.611 (987.861)	24514.76 (5178.426)	109585.811 (32112.526)	408010.193 (58116.759)
Vz (L)	7.349 (1.301)	24.682 (26.064)	16.746 (5.13)	12.87 (1.49)
CL (L/h)	4.828 (0.81)	3.282 (0.412)	2.528 (0.726)	1.955 (0.238)

Pig PK ([redacted])

PK parameter	unit	PK-IV-L value	PK-IC-L value	PK-IV-M value	PK-IC-M value	PK-IV-H value	PK-IC-H value
Dose (total)	[mg/kg b.w.]	1	1	5	5	10	10
est. Body wt	[kg]	30	30	30	30	30	30
Cmax	[ng/mL]	17363	12399	87328	81280	159802	145330
Tmax	[min]	3	3	3	3	3	9
T1/2	[h]	0,88	0,88	0,98	1,00	1,08	1,12
λ	[1/h]	0,79	0,79	0,71	0,69	0,64	0,62
AUC(0-t)	[h x ng/mL]	20401,21	15154,64	212552,66	205013,91	418379,36	385403,36
AUC(0-inf)	[h x ng/mL]	20407,01	15154,64	212583,02	205046,22	418430,27	385461,02
Vd(ss)	[L/kg]	0,06	0,07	0,05	0,04	0,05	0,05
CL	[L/h]	1,38	1,66	0,98	0,92	0,88	0,96
CL	[L/h/kg]	0,05	0,06	0,03	0,03	0,03	0,03

In HF patients included in the Phase 1b study, tissue sampling by invasive myocardial biopsy for PK or miR-132 quantification was not regarded as ethical. Thus, direct heart tissue concentration data was not available. For this reason, data for exposure and reduction in heart tissue level of miR-132 were generated by modeling and extrapolation of the pig PK/PD data to HF patients based on experience with the pig studies. This was supported by data from a comparative measurement study ([redacted]) of circulating miR-132 in plasma samples derived from the Phase 1b study and the large animal pig PD studies in HF models ([redacted]), which showed that plasma levels and treatment response of miR-132 are highly comparable between pigs and humans. Thus, these results provided a solid basis for using the clinical miR-132 plasma data for the modeling of tissue exposure.

For the modeling, corresponding tissue and circulating plasma concentrations of miR-132 assessed in pigs were used as a bridge to translate human miR-132 plasma data to anticipated tissue levels and thereby expected PD activity of the compound.

PK/PD modeling was successfully performed based on the comparison of the human and pig PK as well as of miR-132 data. Based on the calculation using the "best fit" model, in a monthly dosing regimen in both post MI and chronic HF settings, doses in the range of [redacted] to [redacted] mg/kg could provide effective target engagement in cardiac tissue and cardiac improvement in the Phase 2 study.

Although this modeling approach provided a reasonable dose range that fits to the results from the current set of data, the small sample size and level of variability of the Phase 1b study data had to be considered for mitigating the risk of administrating an ineffective dose. From a mathematical point of view, the dose should not be chosen close to the lower limit of 1 mg/kg,

but rather at the higher limit to consider the limitations in the data model adequately. Based on these PK/PD considerations, [redacted] mg/kg was selected as the low dose and by applying an additional margin factor of [redacted] mg/kg was selected as the high dose in the present Phase 2 study, CDR132L-P2-01. Both doses would be adequately covered by the safety data, as CDR132L was well tolerated in the efficacy studies in pigs as well as in toxicological studies in rats and minipigs with monthly administrations for up to 9 months tested ([redacted]). Also, PD studies in the chronic post MI HF pig model, starting treatment was 1 month post MI, showed beneficial effects with 3 monthly doses and 5 monthly doses of [redacted] mg/kg with no safety signals. Thus, Cardior proposes 3 single IV doses of CDR132L, 28 days apart (Day 1, Day 29, and Day 57) from each other to prolong the therapeutic effect by an additional month of CDR132L cardiac tissue exposure and to ensure that patients are not underdosed.

Safety Follow-up Window

Regarding the selection of an appropriate time frame for the safety follow-up in the Phase 2 study, an additional PD modeling was performed to project the changes in the miR-132 target plasma levels for the months after the last CDR132L administration in the Phase 1b study. With CDR132L treatment achieving cardiac improvement, Cardior envisages to achieve a normalization of miR-132 levels on the long run. However, the Phase 1b data generated so far comprised data of 3 months after treatment, and the present Phase 2 study aims to provide data regarding miR-132 levels for a longer period of time, i.e., 10 months after the last dose of CDR132L.

In the Phase 1b study, miR-132 plasma assessments were only performed up to the endpoint of the study at Day 112 (3 months after the last CDR132L administration), wherein miR-132 plasma levels were still markedly reduced due to the sustained pharmacological activity of CDR132L. To ensure that the planned duration of safety follow-up in the planned Phase 2 study will be sufficient to cover the time span of CDR132L pharmacologic activity in the heart and other organs of the patients, a modeling of the miR-132 plasma levels was performed to project the time course of the return of miR-132 plasma levels to baseline.

Reduced miR-132 levels in plasma are due to the effect of CDR132L on plasma and tissue miR-132 as part of the MoA of CDR132L. The level of CDR132L in plasma remains very low but constant over time during the late part of the elimination phase, which is supported by the Phase 1b plasma PK data, where the levels reached the lower limit of quantification after 48 hours. After CDR132L administration and the initial phase of distribution, the source of plasma CDR132L in the circulation is released from tissue, which is balanced by its elimination. Rebound of the miR-132 level in plasma happens when plasma and tissue levels of CDR132L decrease over time, due to the depletion of CDR132L in tissues.

Based on these assumptions and the projection of the Phase 1b miR-132 study data, it is expected that miR-132 plasma levels after treatment with [redacted] mg/kg in humans will reach baseline levels approximately 6.5 months after the last dose.

[redacted]

Based on this data, a 10-month follow-up after the last dosing is sufficient to provide an adequate time window for evaluating safety of CDR132L at the selected doses of [redacted] mg/kg and [redacted] mg/kg.

[redacted]

4.3.2 Timing of Dose for Each Patient

Patients will receive a single IV infusion treatment for [redacted] of CDR132L [redacted] mg/kg, CDR132L [redacted] mg/kg, or placebo on Day 1, Day 29 ± 2 days, and Day 57 ± 2 days, according to the randomization list.

4.4 End of Study Definition

A patient is considered to have completed the study if he/she has completed all phases of the study including the last scheduled procedure shown in the SoA.

The end of the study is defined as the date of the last visit of the last patient in the study or last scheduled procedure shown in the SoA for the last patient in the study globally.

5.0 STUDY POPULATION

Prospective approval of protocol deviations to recruitment and enrollment criteria, also known as protocol waivers or exemptions, is not permitted.

5.1 Inclusion Criteria

Patients are eligible to be included in the study only if all of the following criteria apply:

1. Male or female patients, aged ≥ 30 to ≤ 80 years at the date of signing informed consent which is defined as the beginning of the Screening Period.
2. Spontaneous AMI (type I) based on the universal MI definition with randomization to occur no later than 14 days after index event diagnosis.[23]
3. Patient with a LVEF ≤ 45% as measured by ECHO after MI diagnosis (STEMI or NSTEMI).
4. Patient with NSTEMI with evidence of significant myocardial necrosis, evidenced through a troponin T or troponin I increase to at least 5 times the upper limit of normal (ULN) at MI index event diagnosis.
5. Patient with previous MI events in history can be included.
6. A male patient must agree to use contraception as detailed in Appendix 5 of this protocol during the treatment period and for at least 30 days after the last dose of study treatment and refrain from donating sperm during this period.
7. Patient with body weight of ≤ 120 kg.
8. N-terminal pro B-type natriuretic peptide level ≥ 125 pg/ml and < 8000 pg/ml at screening.

   Note: NT-proBNP values required for eligibility confirmation may be collected at any time post MI either through medical history (e.g., site has collected it as SoC once the patient came to the hospital), through a local laboratory assessment, or by sending a sample to the central laboratory (if sites use the SoC sample or the local laboratory sample for eligibility confirmation, no additional sample for central laboratory assessment is needed).
9. Patient with STEMI/NSTEMI who underwent percutaneous coronary intervention for this event.
10. Capable of giving signed informed consent as described in Appendix 2, which includes compliance with the requirements and restrictions listed in the informed consent form (ICF) and in this protocol.

5.2 Exclusion Criteria

Patients are excluded from the study if any of the following criteria apply:

1. A woman of childbearing potential (WOCBP) as defined in Appendix 5.
2. Patient with HF of non-ischemic origin; e.g., myocarditis, alcoholic cardiomyopathy.
3. Patient with history of decompensated HF or a history of LVEF < 30% within 6 months prior to the Screening Period.

4. Patient with NYHA class IV at screening or randomization.
5. Patient has any planned cardiac intervention (angiogram without angioplasty is acceptable) or any other planned surgery after the Screening Period.
6. Patient has severe valvular heart disease.
7. Patient has systolic BP < 90 mmHg or > 180 mmHg, diastolic BP < 50 mmHg or > 110 mmHg, and/or heart rate < 50 or > 100 beats/minute at screening or randomization.
8. Patient with an estimated glomerular filtration rate < 30 mL/min/1.73 m^2 or on dialysis.
9. Patient with hepatic insufficiency classified as Child-Pugh B or C.
10. Patient with known active human immunodeficiency virus, Hepatitis B, or Hepatitis C infection at screening.
11. Impaired hepatic function defined by a total bilirubin level of ≥ 2 × the ULN and ALT levels of ≥ 3 × ULN.
12. Patient has medical history of disease(s) affecting the blood-brain-barrier, e.g., stroke within 6 months or multiple sclerosis.
13. Patient has medical history of bleeding disorders or has thrombocytopenia (platelets < 100,000/µL).
14. Patient has poorly controlled diabetes as determined by the Investigator.
15. Patient is currently on treatment for epilepsy.
16. Patient has a current or relevant history of physical or psychiatric illness that is/are not stable or may require a change in treatment, use of prohibited therapies during the study, or cause the patient to be unlikely to fully comply with the requirements of the study or complete the study, or any condition that presents undue risk from the study drug or study procedures.
17. Patient has a history or presence of any of the following cardiac conditions: known structural cardiac abnormalities beyond HF, family history of long QT syndrome, cardiac syncope, or recurrent, idiopathic syncope.
18. Any clinically significant abnormalities, at the discretion of the Investigator, in rhythm, conduction, or morphology of resting ECG that pose an additional safety risk to patients. This will include patients with any of the following (at Screening Visit or Day -1):
    a) Clinically significant PR (PQ) interval prolongation.
    b) Intermittent second- or third-degree atrioventricular block.
    c) Sustained cardiac arrhythmia including (but not limited to) supraventricular tachycardia, any symptomatic arrhythmia with the exception of isolated extra systoles.
19. Patient with active "severe acute respiratory syndrome coronavirus 2 (SARS-CoV-2)" infection confirmed as per the local testing guidelines at screening.
20. Patient has other significant disease or disorder which, in the opinion of the Investigator, may put the patient at risk because of participation in the study or may influence the result of the study or the patient's ability to participate in the study.
21. Patient has received an investigational product or treated with an investigational device within 90 days prior to first study drug administration.

22. Patient has known or suspected intolerance or hypersensitivity to the study drug, any closely related compound, or any of the stated ingredients.
23. Patient is not to be enrolled into the study if they received any prohibited therapy within 3 months of screening.
    a) Treatment with anticancer therapy (chemotherapy, immunotherapy, radiotherapy, targeted therapy, or gene therapy) at any time during the study.
    b) Administration of any other investigational agent within 3 months before the first administration of study drug or at any time before the patient's completion of the study.
24. Patient is involved in the planning and/or conduct of the study (applies to Sponsor staff, staff at the study site, and third-party vendors).

5.3 Lifestyle Recommendations

As part of SoC, lifestyle considerations are important, and implementing healthy behaviors (e.g., smoking cessation, physical activity, healthy diet, and maintaining a healthy weight) significantly decreases the risk of future CV events.

5.3.1 Diet Recommendations

A dietary pattern high in fruit, vegetables, legumes, fiber, polyunsaturated fats, nuts, and fish is advocated while avoiding or limiting refined carbohydrates, red meat, dairy, and saturated fat. Salt should be limited to 5 to 6 grams per day. Energy-dense foods such as sugar-sweetened soft drinks should be avoided.

5.3.2 Caffeine, Alcohol, and Tobacco

Patients should abstain from ingesting caffeine- or xanthine-containing products (e.g., coffee, tea, cola drinks, and chocolate) for 3 hours before the clinic visit.

Patients should abstain from alcohol for 24 hours before the clinic visit.

Patients who use tobacco products should be instructed that use of nicotine-containing products (including nicotine patches) should not be permitted while they are in the study center. Patients should be advised to quit smoking. Patients should also avoid passive smoking and use of electronic cigarettes.

5.3.3 Activity

Patients should abstain from strenuous exercise for 24 hours before each blood collection for clinical laboratory tests. Patients may participate in light recreational activities during studies (e.g., watching television, reading).

General physical activity recommendations are 30 to 60 minutes of moderate-intensity aerobic activity at least 5 days per week. Investigators should reassure the patient that exercise is beneficial and educate patients regarding what to do if angina symptoms occur while being

active. Resistance exercises maintain muscle mass, strength, and function, and benefit insulin-sensitivity and control of lipids and BP.

5.4 Screen Failures

Screen failures are defined as patients who consent to participate in the clinical study but are not subsequently entered in the study. A minimal set of screen failure information is required to ensure transparent reporting of screen failure patients to meet the Consolidated Standards of Reporting Trials publishing requirements and to respond to queries from regulatory authorities. Minimal information includes demography, screen failure details, eligibility criteria, and any SAEs.

Patients who do not meet the criteria for participation in this study may be rescreened once. Rescreened patients should not be assigned the same patient number as for the initial screening.

6.0 STUDY TREATMENT

Study treatment is defined as any investigational treatment(s), marketed product(s), placebo, or medical device(s) intended to be administered to a patient according to the study protocol.

6.1 Study Treatment(s) Administered

CDR132L or placebo will be administered IV as detailed in Table 3.The Pharmacy Manual contains detailed information regarding the storage, preparation, destruction, and administration of each treatment. The pharmacy staff preparing the study drug will not be blinded to study drug assignment.

The body weight assessed at the last visit prior to dosing should serve as the reference weight for dose calculation.

Table 3 Study Treatment Details

**Study Treatment Name:**	CDR132L	Placebo
**Dosage Formulation:**	CDR132L drug product will be provided in sterile, [redacted]<br>After reconstitution with [redacted] mg/mL CDR132L is obtained which is further diluted to the specific patient-individual concentration.	Commercial sterile [redacted] will be used as formulation for placebo control.
**Unit Dose Strength(s)/Dosage Level(s):**	CDR132L [redacted] mg/kg, [redacted] mg/kg	Sterile [redacted]
**Route of Administration:**	Intravenous infusion	Intravenous infusion
**Flow Rate**<br>(Details will be provided in the Pharmacy Manual)	[redacted]	[redacted]
**Packaging and Labeling:**	Study drug will be provided in a vial and will be labeled as per country requirement. Labeling of study treatment is performed by [redacted]	Locally-approved commercially available sterile [redacted] will be used.
**Manufacturer:**	Drug Substance: [redacted]	Locally-approved commercially available sterile [redacted] will be used.

6.2 Preparation/Handling/Storage/Accountability

The study drug will be supplied by the Sponsor (or designee), along with the batch/lot numbers and Certificates of Analysis. As reconstitution and dilution solvent for preparation of the CDR132L dosing solutions, and for preparation of the placebo dosing solution, commercially available, locally-approved sterile 0.9% NaCl (saline) solution will be used, which will be sourced locally by the study site.

CDR132L study drug is stored at the recommended storage temperature of [redacted]. Based on stability studies, the current shelf-life of CDR132L vials is [redacted].

The pharmacy personnel must confirm appropriate temperature conditions have been maintained during transit for all study treatment received and any discrepancies are reported and resolved before use of the study treatment.

All study treatments must be stored in a secure, environmentally controlled, and monitored (manual or automated) area in accordance with the labeled storage conditions with access limited to the Investigator and authorized site staff.

To obtain the clinical administration solution for IV infusion, CDR132L [redacted]

CDR132L will be administered through a [redacted]. Each vial containing [redacted] mg CDR132L [redacted] into the vial, and the volume needed to match the required dose/bodyweight, plus 8.3% will be injected into the perfusion syringe. Thus, multiple vials will be used to provide the required dose. Subsequently, the [redacted]. Placebo dosing solution will be identically prepared, by filling the [redacted] (without adding CDR132L).

Only patients enrolled in the study may receive study treatment and only authorized site staff may supply or administer study treatment. The Investigator, institution, or the head of the medical institution (where applicable) is responsible for study treatment accountability, reconciliation, and record maintenance (i.e., receipt, reconciliation, and final disposition records).

The Investigator, a member of the study center staff, or a hospital pharmacist must maintain an adequate record of the receipt and distribution of all study medication using the Drug Accountability Form. These forms must be available for inspection at any time.

Further guidance and information for the final disposition of unused study treatment are provided in the Pharmacy Manual.

6.3 Measures to Minimize Bias: Randomization and Blinding

All patients will be centrally randomized using an Interactive Voice/Web Response System (IVRS/IWRS/IXRS). Before the study is initiated, the telephone number and call-in directions for the IWRS and/or the log in information and directions for the IWRS will be provided to each site.

This study drug will be administered in a double-blind fashion whereby patients and clinical study site staff will be blinded to the study treatment up to Month 6, with study personnel having limited access to the randomization code. The investigational treatment and placebo will be identical in physical appearance. The assignment of treatment each patient will receive will not be disclosed to the Investigator, study center staff, patient, Sponsor, or study vendors. During the study, the individual randomization codes will be kept in the site's clinical study pharmacy, accessible to the pharmacy personnel only. Sponsor staff involved in clinical decision-making (such as those involved in DSMB decisions) will be blinded to study drug assignment up to Month 6.

Patients will be randomly assigned in a 1:1:1 ratio to receive study treatment. Study treatment will be administered at the study visits summarized in the SoA (Section 1.3). Investigators and patients will remain blinded to each patient's assigned study treatment up to Month 6. In order to maintain this blind, an otherwise uninvolved 3rd party (i.e., pharmacy staff) will be responsible for the reconstitution and dispensation of all study treatment and will endeavor to ensure that there are no differences in time taken to dispense following randomization. The pharmacy staff preparing the study drug will not be blinded to study drug assignment.

In the event of a Quality Assurance audit, the auditor(s) will be allowed access to unblinded study treatment records at the study center(s) to verify that randomization/dispensing has been done accurately.

Upon completion of Month 6, after the database lock and after the blind is revealed, the randomization list will be filed in the Trial Master File.

6.4 Study Treatment Compliance

The prescribed dosage, timing, and mode of administration may not be changed. Any departures from the intended regimen must be recorded in the electronic case report forms (eCRFs).

Doses of CDR132L or placebo will be administered by IV infusion by the Investigator or designee. Detailed instructions for dose administration will be included in the Pharmacy Manual.

Each dose of study drug will be administered by study personnel at each site and therefore treatment compliance will not be formally evaluated in this study. Each patient will be considered to have received a complete dose of study drug if they receive at least 80% of study drug per infusion.

6.5 Concomitant Therapy

Any medication or vaccine (including over-the-counter or prescription medicines, vitamins, and/or herbal supplements) that the patient is receiving from 30 days prior to the Screening Period, at the time of enrollment, and receives during the study must be recorded on the eCRF along with:

- Reason for use.
- Dates of administration including start and end dates.
- Dosage information including dose and frequency.
- For vaccines (if applicable): include brand name and manufacturer (plus lot number if available).

Medications/treatments for diabetes, atrial fibrillation, and HF according to SoC as per the latest local guidelines should be given during the study if patients are stable on therapy.

Patients using stable doses of prescription or over-the-counter medications for stable conditions may be enrolled at the discretion of the Investigator. During the study, other prescription or over-the-counter medications may be permitted at the discretion of the Investigator. In any case, the Investigator will inform the Sponsor about the concurrent medication given. Other investigational procedures and products while participating in this study are prohibited.

Details of all other prior and concomitant medications will be recorded by the Investigator on the eCRF and source record.

The Medical Monitor should be contacted if there are any questions regarding concomitant or prior therapy.

6.5.1 Prohibited Therapies

The following therapies are not permitted during the study:

- Treatment with anticancer therapy (chemotherapy, immunotherapy, radiotherapy, targeted therapy, or gene therapy) at any time during the study.
- Administration of any other investigational agent within 3 months before the first administration of study drug or at any time before the patient's completion of the study.

Patients are not to be enrolled into the study if they received any prohibited therapy within 3 months of screening. If administration of any prohibited therapy becomes necessary during the study for medical reasons, the patient may be discontinued from the study. The requirements for handling early discontinuations from study drug are described in Section 7.0.

Vaccination with live vaccines while on study is prohibited. Administration of inactivated vaccines and mRNA vaccines is allowed (e.g., inactivated influenza vaccines or SARS-CoV-2 vaccines).

6.6 Dose Modification

Dose modifications will not be allowed. If a dose modification is needed, the patient should be discontinued from treatment and all end of treatment assessments completed.

6.7 Treatment After the End of the Study

The Sponsor will not provide any additional care to patients after they leave the study because such care should not differ from what is normally expected for patients with MI.

7.0 DISCONTINUATION OF STUDY TREATMENT AND PATIENT DISCONTINUATION/WITHDRAWAL

7.1 Discontinuation of Study Treatment

A patient's study treatment may be discontinued for any of the following reasons:

- AE:
  - A patient may be discontinued from the study if, in the judgment of the Investigator, the patient develops an AE such as an intercurrent illness or complication that is not consistent with the protocol requirements or that, in any way, justifies withdrawal from the study.
- Discontinuation of study treatment for abnormal liver function should be considered by the Investigator when a patient meets one of the conditions outlined below or if the Investigator believes that it is in best interest of the patient:
  - ALT > 8 × ULN.
  - ALT > 5 × ULN for > 2 weeks.
  - ALT > 3 × ULN and (total bilirubin > 2 × ULN or international normalized > 1.5 × ULN).
  - ALT > 3 × ULN with the appearance of fatigue, nausea, vomiting, right upper quadrant pain or tenderness, fever, rash, and/or eosinophilia (> 5%).
- If a clinically significant finding is identified (including, but not limited to changes from baseline in QT interval corrected using Fridericia's formula [QTcF]) after enrollment, the Investigator or qualified designee will determine if the patient can continue in the study and if any change in patient management is needed. This review of the ECG printed at the time of collection must be documented. Any new clinically relevant finding will be reported as an AE. A patient who meets either below criterion based on ECG readings will be withdrawn from the study.
  - QTcF > 500 msec OR uncorrected QT > 600 msec
  - Change from baseline of QTcF > 60 msec

For patients with underlying bundle branch block, follow the discontinuation criteria listed below:

Baseline QTcF with Bundle Branch Block	Discontinuation QTcF Threshold with Bundle Branch Block
< 450 msec	> 500 msec
450 to 480 msec	≥ 530 msec

- Noncompliance with study protocol requirements.

- Investigator decision.
- Discontinuation of treatment on account of protocol deviation.
- Withdrawal by patient (see Section 7.2).
- Study termination by Sponsor or regulatory bodies/agency.
- Other, non-AE.

If a patient who does not meet enrollment criteria is inadvertently enrolled, that patient must be discontinued from study treatment and the Sponsor or Sponsor designee must be contacted. An exception may be granted in rare circumstances for which there is a compelling safety reason to allow the patient to continue. In these rare cases, the Investigator must obtain documented approval from the Sponsor or Sponsor designee to allow the patient to continue in the study.

Patients who discontinue treatment will be followed up as patients who do not discontinue treatment unless consent is withdrawn.

The reason for discontinuation of study treatment will be recorded in the clinical records and the patient's eCRF.

Patients who discontinue study treatment will not be replaced.

See the SoA (Section 1.3) for data to be collected at the time of treatment discontinuation and follow-up and for any further evaluations that need to be completed.

7.2 Patient Discontinuation/Withdrawal from the Study

A patient may withdraw from the study at any time at his/her own request or may be withdrawn at any time at the discretion of the Investigator for safety, behavioral, compliance, positive coronavirus disease 2019 (COVID-19) test or suspected SARS-CoV-2 infection, or administrative reasons.

If the patient withdraws consent for disclosure of future information, the Sponsor may retain and continue to use any data collected before such a withdrawal of consent.

If a patient withdraws from the study, he/she may request destruction of any samples taken and not tested, and the Investigator must document this in the study center study records.

7.3 Lost to Follow-up

Discontinuation of specific study centers or of the study are handled as part of Appendix 2.

A patient will be considered lost to follow-up if he/she repeatedly fails to return for scheduled visits and is unable to be contacted by the study site.

The following actions must be taken if a patient fails to return to the clinic for a required study visit:

- The site must attempt to contact the patient and reschedule the missed visit as soon as possible, counsel the patient on the importance of maintaining the assigned visit schedule and ascertain whether or not the patient wishes to and/or should continue in the study.
- In cases in which the patient is deemed lost to follow-up, the Investigator or designee must make every effort to regain contact with the patient (where possible, 3 telephone calls and, if necessary, a certified letter to the patient's last known mailing address or local equivalent methods). These contact attempts should be documented in the patient's medical record.
- Should the patient continue to be unreachable, he/she will be considered to have withdrawn from the study with a primary reason of lost to follow-up.

8.0 STUDY ASSESSMENTS AND PROCEDURES

Study procedures and their timing are summarized in the SoA (Section 1.3).

Protocol waivers or exemptions are not allowed.

Immediate safety concerns should be discussed with the Sponsor immediately upon occurrence or awareness to determine if the patient should continue or discontinue study treatment.

Adherence to the study design requirements, including those specified in the SoA, is essential and required for study conduct.

All screening evaluations must be completed and reviewed to confirm that potential patients meet all eligibility criteria. The Investigator will maintain a screening log to record details of all patients screened and to confirm eligibility or record reasons for screening failure, as applicable.

Procedures conducted as part of the patient's routine clinical management (e.g., blood count) and obtained before signing of the ICF may be utilized for screening or baseline purposes provided the procedures met the protocol-specified criteria and were performed within the time frame defined in the SoA.

The maximum amount of blood collected from each patient over the duration of the study, including any extra assessments that may be required, will not exceed 450 mL (in 12 weeks). Repeat or unscheduled samples may be taken for safety reasons or for technical issues with the samples.

8.1 Efficacy Assessments

Planned time points for all safety assessments are provided in the SoA (Section 1.3).

8.1.1 Echocardiography Central Laboratory

The ECHO results will be read and interpreted for the study purposes centrally as described below. The assessing Investigator will be blinded to the study treatment assignments.

8.1.1.1 Left Ventricular End-systolic Volume Index

Left ventricular end-systolic volume index is considered one of the standard ECHO markers for assessing risks in ischemic HF. The evaluation of change in left ventricular end-systolic volume index (LVESVI) at Month 6 from its value at baseline is the primary efficacy endpoint of this study. The ECHO will be performed to assess LVESVI. Full details of this procedure will be provided in the Imaging Study Manual.

8.1.1.2 Cardiac Function – Left Ventricular Ejection Fraction

Left ventricular ejection fraction is an established method for evaluation of left ventricular systolic function. A 2D ECHO will be performed in the ECHO central laboratory by trained

personnel to assess LVEF. Contrast ECHO may be an available option to enhance image quality. Full details of this procedure will be provided in the Imaging Study Manual.

8.1.1.3 Left Ventricular End-diastolic Volume, Stroke Volume, and Systolic Ejection Time

Cardiac parameters such as left ventricular end-diastolic volume, early (E) and late (A) diastolic transmitral flow velocity, early diastolic mitral annular velocity (e'), left atrial volume index, stroke volume (mL), systolic ejection time (ms), and strain analysis for global longitudinal strain will also be assessed by ECHO. Full details of these procedures will be provided in the Imaging Study Manual.

8.1.1.4 Global Longitudinal Strain

Global longitudinal strain imaging of the LV is a method for objectively assessing the global and regional functions of the LV of the heart. Left ventricular longitudinal systolic function will be analyzed by 2D ECHO.

8.1.2 Patient Well-being

Patient's well-being will be measured by Kansas City Cardiomyopathy Questionnaire (KCCQ).

8.1.2.1 Kansas City Cardiomyopathy Questionnaire

The KCCQ is a 23-item, self-administered questionnaire developed to independently measure the patient's perception of their health status, which includes HF symptoms, impact on physical and social function, and how their HF impacts their quality of life within a 2-week recall period.

8.1.3 Efficacy-related Biomarkers

8.1.3.1 Absolute Changes from Baseline in NT-proBNP

The increase in circulating NT-proBNP will reflect the diminished capacity of the heart to deliver oxygenated blood to the body. A test for NT-proBNP will be used to evaluate the severity of HF.

8.1.3.2 Troponin T

An elevated level of troponin T indicates heart muscle damage or HF and therefore troponin T is used as a biomarker of myocardial injury. Troponin T levels will be measured by hs-cTn.

[redacted]

[redacted]

[redacted]

8.2 Safety Assessments

Planned time points for all safety assessments are provided in the SoA (Section 1.3).

8.2.1 Physical Examinations

The Investigator or designee will perform a complete physical examination at screening. A brief physical examination will be conducted during the study unless a complete physical examination is needed.

A complete physical examination will include, at a minimum, assessments of the CV, Respiratory, Gastrointestinal, and Neurological systems. Height will only be measured at screening and weight will be measured and recorded per the SoA (Section 1.3).

A brief physical examination will include, at a minimum, assessments of the skin, lungs, CV system, and abdomen (liver and spleen).

Investigators should pay special attention to clinical signs related to previous serious illnesses.

8.2.2 Vital Signs

Body temperature, pulse rate, BP, and respiratory rate will be assessed.

Blood pressure and pulse measurements will be assessed in supine position with a completely automated device. Manual techniques will be used only if an automated device is not available.

Blood pressure and pulse measurements should be preceded by at least 5 minutes of rest for the patient in a quiet setting without distractions (e.g., television, cell phones).

Vital signs (to be taken before blood collection for laboratory tests) will consist of 1 pulse and 3 BP measurements (3 consecutive BP readings will be recorded at intervals of at least 1 minute). The average of the 3 BP readings will be recorded on the eCRF.

8.2.3 Electrocardiograms

12-lead ECG will be obtained as outlined in the SoA (see Section 1.3) using an ECG machine that automatically calculates the heart rate and measures PR, QRS, QT, and QTc intervals. 12-lead ECG recordings should be made after the patient is rested in a supine position for at least 10 minutes. The postural changes by patients will be avoided during the ECG recordings and clinical staff will ensure that patients are awake during the ECG recording.

Refer to Section 7.0 for (QTc) withdrawal criteria and any additional QTc readings that may be necessary.

8.2.4 Clinical Safety Laboratory Assessments

See Appendix 3 for the list of clinical laboratory tests to be performed and to the SoA for the timing and frequency.

The Investigator must review the laboratory report, document this review, and record any clinically relevant changes occurring during the study in the AE section of the eCRF. The laboratory reports must be filed with the source documents. Clinically significant abnormal laboratory findings are those that are not associated with the underlying disease, unless judged by the Investigator to be more severe than expected for the patient's condition.

All laboratory tests with values considered clinically significantly abnormal during participation in the study or within EOS should be repeated until the values return to normal or baseline or are no longer considered clinically significant by the Investigator or Medical Monitor.

- If such values do not return to normal/baseline within a period of time judged reasonable by the Investigator, the etiology should be identified and the Sponsor is notified.
- All protocol-required laboratory assessments, as defined in Appendix 3, must be conducted in accordance with the laboratory manual and the SoA.
- The Investigator must determine whether an abnormal value in an individual study patient represents a clinically significant change from the patient's baseline values. In general, abnormal laboratory findings without clinical significance (based on the Investigator's judgment) are not to be recorded as AEs.
- If laboratory values from non-protocol-specified laboratory assessments performed at the institution's local laboratory require a change in patient management or are considered clinically significant by the Investigator (e.g., SAE or AE or dose modification), then the results must be recorded in the eCRF.

For urinalysis, if deemed necessary, based on a clinically significant positive test, microscopic examination of urine will be performed. Microscopic examination result will supersede the urine dip result.

8.3 Adverse Events

The definitions of an AE or SAE can be found in Appendix 4.

Adverse events will be reported by the patient (or, when appropriate, by a caregiver, surrogate, or the participant's legally authorized representative).

The Investigator and any designees are responsible for detecting, documenting, and recording events that meet the definition of an AE or SAE and remain responsible for following up AEs that are serious, considered related to the study treatment or study procedures, or that caused the patient to discontinue the study treatment (see Section 7.0).

8.3.1 Time Period and Frequency for Collecting AE and SAE Information

All SAEs will be collected from the signing of the ICF until the follow-up visit at the time points specified in the SoA (Section 1.3).

All AEs will be collected from the signing of the ICF until the follow-up visit at the time points specified in the SoA (Section 1.3).

Medical occurrences that begin before the start of study treatment but after obtaining informed consent will be recorded on the Medical History/Current Medical Conditions section of the eCRF, not the AE section.

All SAEs will be recorded and reported to the Sponsor or designee immediately and under no circumstance should this exceed 24 hours, as indicated in Appendix 4. The Investigator will submit any updated SAE data to the Sponsor or designee within 24 hours of their awareness of the updated information.

Investigators are not obligated to actively seek AE or SAE after conclusion of the study participation. However, if the Investigator learns of any SAE, including a death, at any time after a patient has been discharged from the study, and he/she considers the event to be reasonably related to the study treatment or study participation, the Investigator must promptly notify the Sponsor or designee.

The method of recording, evaluating, and assessing causality of AE and SAE and the procedures for completing and transmitting SAE reports are provided in Appendix 4.

8.3.2 Method of Detecting AEs and SAEs

Care will be taken not to introduce bias when detecting AEs and/or SAEs. Open-ended and non-leading verbal questioning of the patient is the preferred method to inquire about AE occurrences.

8.3.3 Follow-up of AEs and SAEs

After the initial AE/SAE report, the Investigator is required to proactively follow each patient at subsequent visits/contacts. All SAEs, and non-serious AESIs (as defined in Section 8.3.6), will be followed until resolution, stabilization, the event is otherwise explained, or the patient is lost to follow-up (as defined in Section 7.3). Further information on follow-up procedures is given in Appendix 4.

8.3.4 Regulatory Reporting Requirements for SAEs

Prompt notification by the Investigator to the Sponsor or designee of an SAE is essential so that legal obligations and ethical responsibilities towards the safety of patients and the safety of a study treatment under clinical investigation are met. See also Section 8.3.1.

The Sponsor has a legal responsibility to notify both the local regulatory authority and other regulatory agencies about the safety of a study treatment under clinical investigation. The Sponsor will comply with country-specific regulatory requirements relating to safety reporting to the regulatory authority, Institutional Review Boards (IRB)/IEC, and Investigators.

Investigator safety reports must be prepared for suspected unexpected serious adverse reactions according to local regulatory requirements and Sponsor policy and forwarded to Investigators as necessary.

An Investigator who receives an Investigator safety report describing an SAE or other specific safety information (e.g., summary or listing of SAEs) from the Sponsor will review and then file it along with the IB and will notify the IRB/IEC, if appropriate according to local requirements.

8.3.5 Pregnancy

Details of all pregnancies in (female partners of male patients) will be collected after the start of study treatment and until EOS.

If a pregnancy is reported, the Investigator should inform the Sponsor or designee within 24 hours of learning of the pregnancy and should follow the procedures outlined in Appendix 5.

Abnormal pregnancy outcomes (e.g., spontaneous abortion, fetal death, stillbirth, congenital anomalies, ectopic pregnancy) are considered SAEs.

8.3.6 Adverse Events of Special Interest

An AESI is an AE of scientific or medical concern specific to the Sponsor or the particular product or program, for which ongoing monitoring and rapid communication by the Investigator to the Sponsor may be appropriate. It may require further investigation to characterize and understand them. It could be serious or nonserious and may include events that may be potential precursors or prodromes for more serious medical conditions in susceptible individuals.

The AESI of thrombocytopenia will be reported by the Investigator as this has been reported for oligonucleotides but not so far for CDR132L.

8.4 Treatment of Overdose

An overdose is any dose of study treatment given to a patient that exceeds the dose described in the protocol. There is no information regarding overdose with CDR132L.

Any overdose, with or without associated AEs, must be promptly reported to the Sponsor or designee. In case of any AEs associated with the overdose, these should be reported on relevant AE/SAE sections in the eCRF.

8.5 Pharmacokinetics

PK parameters are not evaluated in this study.

8.6 Pharmacodynamics

Biomarkers for efficacy and exploratory efficacy analyses are provided in Section 8.1.3 and Section 8.1.4.4, respectively.

8.7 Biomarkers

Biomarkers for efficacy and exploratory efficacy analyses are provided in Section 8.1.3 and Section 8.1.4.4, respectively. Instructions for the collection and handling of biological samples will be provided by the Sponsor. The actual date and time (24-hour clock time) of each sample will be recorded.

All efficacy-related and exploratory efficacy-related biomarkers will be analyzed by a central laboratory. Additional details can be found in the Laboratory Manual.

8.8 Genetics

Genetics are not evaluated in this study.

8.9 Health Economics OR Medical Resource Utilization and Health Economics

Health Economics/Medical Resource Utilization and Health Economics parameters are not evaluated in this study.

9.0 STATISTICAL CONSIDERATIONS

This section summarizes the planned statistical analyses of the endpoints, including primary and secondary endpoints. The statistical analysis plan (SAP) will be developed and finalized before database lock for the 6-month primary efficacy and safety analyses and will describe the patient analysis sets to be included in the analyses, and procedures for accounting for missing, unused, and spurious data. Any subsequent amendments to the SAP will be documented, with final amendments completed before unblinding of the data for the final analysis.

Primary analysis of efficacy and safety will be done once the last patient has completed the 6-month time point. Data from the extension period will be analyzed separately once the last patient has completed last visit (Month 12). A clinical study report will be written following the completion of the study (Day 360/Month 12).

9.1 Sample Size Determination

The primary endpoint is defined as percent (%) change from baseline LVESVI at Month 6 compared with baseline (after reperfusion, before treatment start) on top of SoC as measured by ECHO (central laboratory).

For the sample size calculation, the two-sample t-test is used. The test level is set to 2.5% (one-sided). Assumptions are displayed below.

Left ventricular end-systolic volume index	Mean (change in %)	Standard deviation (%)
Placebo response	1	9
[redacted] mg/kg	6	9
[redacted] mg/kg	5	9
N = 90 per group	Step 1	96.0%
	Step 2	84.3%
	Overall	80.9%

<u>Step 1 ([redacted] mg/kg versus placebo):</u>

A sample size of 90 in each group will have 96.0% power to detect a difference in means of 5 (the difference between a Group 1 mean, μ_1, of 6 and a Group 2 mean, μ_2, of 1) assuming that the common standard deviation is 9 using a 2-group t-test with a 2.5% one-sided significance level.

<u>Step 2 ([redacted] mg/kg versus placebo):</u>

A sample size of 90 in each group will have 84.33% power to detect a difference in means of 4 (the difference between a Group 1 mean, μ_1, of 5 and a Group 2 mean, μ_2, of 1) assuming that the common standard deviation is 9 using a 2-group t-test with a 2.5% one-sided significance level.

Including 90 patients per group (270 total) will lead to a power of 96.0% for the first step ([redacted] mg/kg vs placebo, difference in means of 5) and 84.3% for the second step ([redacted] mg/kg vs placebo, difference in means of 4) of the hierarchical test procedure, resulting in an overall power of 80.9%. Ten additional patients should be included to compensate for early dropouts.

It is planned that approximately 280 patients will be enrolled/randomized into the study in a 1:1:1 ratio (approximately 90 patients each in [redacted] mg/kg, [redacted] mg/kg, and placebo groups). Assuming 10 patients will not be evaluable, it is anticipated that 270 patients will be evaluable (90 patients each in [redacted] mg/kg, [redacted] mg/kg, and placebo groups).

9.2 Populations for Analyses

For purposes of analysis, the analysis sets in Table 4 are defined. Additional analysis sets may be defined in the SAP.

Table 4 Analysis Sets

Analysis Set	Description
Entered Analysis Set	All patients who sign the informed consent form.
Intent-to-treat	All patients in the Entered Analysis Set who are randomized to study drug. Patients will be analyzed according to their randomized study treatment.
Safety	All randomized patients who receive at least 1 dose of study drug (CDR132L or placebo) and have at least 1 post-dose safety assessment. Patients will be analyzed according to the treatment they actually received.

9.3 Statistical Analyses

9.3.1 Efficacy Analyses

The Intent-to-treat (ITT) Population will be the primary analysis population. Additionally, sensitivity analysis will be done using the Full Analysis Set and will be described further in the SAP.

The estimand for establishing the efficacy of CDR132L consists of the mean difference in change from baseline in percent change in LVESVI at Month 6 among MI patients randomized to either active treatment or placebo group calculated in the ITT analysis set using analysis of covariance (ANCOVA). This primary analysis of the primary efficacy endpoint will be conducted regardless of intercurrent events, following the treatment policy strategy.[24] Under this strategy imputation of the primary efficacy endpoint among patient dropouts who are known to be alive during the assessment of the primary efficacy endpoint may be imputed. The efficacy of CDR132L based on mortality will be assessed at Month 12.

9.3.1.1 Primary Efficacy Analysis

The primary endpoint is defined as percent (%) change from baseline LVESVI at Month 6 compared with baseline (after reperfusion, before treatment start) on top of SoC as measured by ECHO (central laboratory).

The comparison will be done for the [redacted] mg/kg dose group versus placebo, followed by [redacted] mg/kg versus placebo within a hierarchical two-step test procedure.

The aim is to show superiority of the [redacted] mg/kg dose group (μ_{T10}) and the [redacted] mg/kg dose group (μ_{T5}) in comparison to placebo (μ_P) within a hierarchical test procedure with 2 steps:

Step 1

- H01: $\mu T10 \leq \mu P$
- H11: $\mu T10 > \mu P$

If Step 1 is successful, then proceed to Step 2.

If Step 1 is not successful, then do not proceed to Step 2.

Step 2

- H02: $\mu T5 \leq \mu P$
- H12: $\mu T5 > \mu P$

To show superiority, the effect of the [redacted] mg dose group on the percent change in LVESVI must be larger (i.e., higher decrease) than the effect within the placebo group.

The percent change from baseline in LVESVI will be analyzed using ANCOVA, with placebo acting as the reference. The model will include treatment as a fixed effect and baseline LVESVI as a covariate.

9.3.1.2 Secondary Efficacy Analysis

The change from baseline in continuous secondary endpoints will also be analyzed using ANCOVA, with placebo acting as the reference. The model will include treatment as a fixed effect and baseline value as a covariate.

9.3.1.3 Exploratory Efficacy Analysis

The exploratory analyses will be described in the SAP finalized before database lock.

9.3.2 Safety Analyses

Safety variables include incidence of AEs and TEAEs, laboratory test results, vital signs, ECG results, and physical examination findings. All safety analyses will be based on the Safety Population. No formal statistical analysis of the safety data will be performed.

Treatment-emergent adverse events are defined as AEs that first occurred or worsened in severity after the first administration of study treatment and prior to 30 days after the last administration of study treatment.

Adverse events will be coded using the Medical Dictionary for Regulatory Activities. For each study treatment, numbers of TEAEs and incidence rates will be tabulated by preferred term and system organ class (SOC). Treatment-emergent adverse events by maximum severity, TEAEs by relationship to study treatment, SAEs, TEAEs leading to death, and TEAEs leading to discontinuation of study treatment will be tabulated for each treatment group. Commonly occurring TEAEs, i.e., those that occur in 5% or more of the patients in either treatment group, will be summarized using descriptive statistics.

The AE summary tables will include counts of patients. Therefore, if a patient experiences more than one episode of a particular AE, the patient will be counted only once for that event. If a patient has more than one AE that is coded to the same preferred term, the patient will be

counted only once for that preferred term. Similarly, if a patient has more than one AE within a SOC, the patient will be counted only once in that SOC.

All laboratory test results, vital signs measurements, ECG results, weight, and body mass index will be summarized for each treatment group using descriptive statistics at each visit for raw numbers and change from baseline. The incidence of treatment-emergent abnormal laboratory, vital sign, and ECG values will also be summarized using descriptive statistics.

Laboratory test variables will be summarized by treatment group and visit using descriptive statistics (number of patients, mean, standard deviation, minimum, maximum, and mean change from baseline). Laboratory tests with categorical results (e.g., urinalysis) will be summarized by treatment group and visit by the number of patients in each category. Shift tables (low, normal, high) between baseline and post-baseline time points will be presented by laboratory test and treatment group. Laboratory tests with categorical results that cannot be analyzed by change from baseline or shift table analysis will not be included in these summaries but will be listed. Data obtained from laboratory tests not required by the protocol will not be summarized but will be listed.

Descriptive statistics of vital signs and ECG results at each visit will be presented by treatment group. Physical examination findings will be listed for each patient.

9.3.3 Missing Data

Sites will be instructed to minimize dropouts and if possible, collect to retrieve efficacy data among patients who withdraw from the study early. Such data from retrieved dropouts may be used to impute missing data.

Missing efficacy data (including those due to early discontinuations) may be imputed by having the previous visit value carried forward (last observation carried forward).

No safety data will be imputed.

9.4 Interim Analyses

No interim analysis for efficacy is planned. Primary analysis will be done once the last patient has completed the 6-month time point. Follow-up data after this time point will be analyzed separately once the last patient has completed last visit (Month 12). A clinical study report will be written following the completion of the study (Day 360/Month 12).

9.5 Data Safety Monitoring Board

A DSMB consisting of members who are independent from the Sponsor will be established. The DSMB membership will have a minimum of 3 members, including at least 1 specialist with expertise in Cardiology and an independent statistician, as appropriate. The DSMB is responsible for reviewing and evaluating unblinded safety data collected after the initiation of the study;

however, the frequency could be adjusted based on accrual and pertinent dose information availability. The DSMB may also meet in ad hoc meetings at its discretion as needed in response to events occurring in the study. The DSMB will be responsible for making recommendations as to whether it is scientifically and ethically appropriate to continue enrollment, discontinue treatment groups, or stop the study. The detailed information on how to run or manage the DSMB will be described in the DSMB Charter.

10.0 REFERENCES

1. Ucar A, Gupta SK, Fiedler J, Erikci E, Kardasinski M, Batkai S, et al. The miRNA-212/132 family regulates both cardiac hypertrophy and cardiomyocyte autophagy. Nat Commun 2012;3:1078.
2. Foinquinos A, Batkai S, Genschel C, Viereck J, Rump S, Gyöngyösi M, et al. Preclinical development of a miR-132 inhibitor for heart failure treatment. Nat Commun 2020;11(1):633.
3. Batkai S, Genschel C, Viereck J, Rump S, Bär C, Borchert T, et al. CDR132L improves systolic and diastolic function in a large animal model of chronic heart failure. Eur Heart J. 2021;42(2):192-201.
4. Täubel J, Hauke W, Rump S, Viereck J, Batkai S, Poetzsch J, Rode L, Weigt H, et al. Novel antisense therapy targeting microRNA-132 in patients with heart failure: results of a first-in-human Phase 1b randomized, double-blind, placebo-controlled study. Eur Heart J. 2021;42(2):178-188.
5. Hinkel R, Batkai S, Bähr A, Bozoglu T, Straub S, Borchert T, et al. AntimiR-132 Attenuates Myocardial Hypertrophy in an Animal Model of Percutaneous Aortic Constriction. J Am Coll Cardiol. 2021;77(23):2923-2935.
6. Thum T, Galuppo P, Wolf C, Fiedler J, Kneitz S, Van Laake LW, et al. MicroRNAs in the human heart: A clue to fetal gene reprogramming in heart failure. Circulation 2007;116(3):258-267.
7. Kim GH, Uriel N, Burkhoff D. Reverse remodelling and myocardial recovery in heart failure. Nat Rev Cardiol 2018;15(2):83-96.
8. CDR132L [Investigator's Brochure]. Cardior Pharmaceuticals GmbH. Version 2.0, 15 November 2021.
9. Lippi G, Sanchis-Gomar F. Global epidemiology and future trends of heart failure. AME Med J 2020;5:1.
10. Granger CB, Goldberg RJ, Dabbous OM, Pieper KS, Eagle KA, Cannon CP, et al. Predictors of hospital mortality in the global registry of acute coronary events. Arch Intern Med 2003;163:2345–2353.
11. Bahit MC, Kochar A, Granger CB. Post-myocardial infarction heart failure. JACC Heart Fail 2018;6:179-186.
12. Hellermann JP, Jacobsen SJ, Gersh BJ, Rodeheffer RJ, Reeder GS, Roger VL. Heart failure after myocardial infarction: a review. Am J Med 2002;113:324–330.
13. Hinkel R, Batkai S, Bahr A, Bozoglu T, Straub S, Borchert T, et al. AntimiR-132 attenuates myocardial hypertrophy in an animal model of percutaneous aortic constriction. Am Coll Cardiol 2021;77:2923-35.
14. Van der Meer P, Gaggin HK, Dec GW. ACC/AHA versus ESC guidelines on heart failure JACC guideline comparison. J Am Coll Cardiol 2019;73(21):2756-2768.

15. Braendli-Baiocco A, Festag M, Dumong Erichsen K, Persson R, Mihatsch MJ, Fisker N, et al. From the Cover: The Minipig is a suitable non-rodent model in the safety assessment of single stranded oligonucleotides. Toxicol Sci. 2017;157(1):112-128.
16. Henry, Kim TW, Kramer-Stickland K, Zanardi TA, Fey RA, Levin AA. 2007. Toxicologic properties of 2′-o-methoxyethyl chimeric antisense inhibitors in animals and man. *Antisense Drug Technology: Principles, Strategies, and Applications, Second Edition* (pp. 327–363). CRC Press.
17. Thuilliez C, Tortereau A, Perron-Lepage MF, Howroyd P, Gauthier B. Spontaneous testicular tubular hypoplasia/atrophy in the Göttingen minipig: a retrospective study. Toxicol Pathol. 2014;42(6):1024-31.
18. Lindow M, Kauppinen S. Discovering the first microRNA-targeted drug. J Cell Biol 2012;199(3):407-412.
19. Evers MM, Toonen LJ, van Roon-Mom WM. Antisense oligonucleotides in therapy for neurodegenerative disorders. Adv Drug Deliv Rev 2015;87:90-103.
20. Chi X, Gatti P, Papoian T. Safety of antisense oligonucleotide and siRNA-based therapeutics. Drug Discov Today. 2017;22(5):823-833.
21. Thygesen K, Alpert JS, Jaffe AS, Chaitman BR, Bax JJ, Morrow DA, et al. Fourth universal definition of myocardial infarction (2018). J Am Col Cardiol 2018;72:2231-64.
22. Puymirat E, Simon T, Cayla G, Cottin Y, Elbaz M, Coste P, et al. Acute myocardial infarction: changes in patient characteristics, management, and 6-month outcomes over a period of 20 years in the FAST-MI program (French Registry of Acute ST-Elevation or Non-ST-Elevation Myocardial Infarction) 1995 to 2015. Circulation 2017;136:1908–1919.
23. Thygesen K. 'Ten Commandments' for the Fourth Universal Definition of Myocardial Infarction 2018. Eur Heart J. 2019 Jan 14;40(3):226.
24. E9(R1) Statistical Principles for Clinical Trials: Addendum: Estimands And Sensitivity Analysis in Clinical Trials Guidance for Industry. Retrieved from: https://www.fda.gov/regulatory-information/search-fda-guidance-documents/e9r1-statistical-principles-clinical-trials-addendum-estimands-and-sensitivity-analysis-clinical Accessed on 11 October 2021.

11.0 APPENDICES

Appendix 1 Abbreviations

Abbreviation	Definition
AE	Adverse event
AESI	Adverse event of special interest
ALT	Alanine aminotransferase
AMI	Acute myocardial infarction
ANCOVA	Analysis of covariance
ASO	Antisense oligonucleotide
BP	Blood pressure
C_{max}	Maximum concentration
CNS	Central nervous system
COVID-19	Coronavirus disease 2019
CV	cardiovascular
DSMB	Data safety monitoring board
EC	Ethics Committee
ED	Emergency Department
ECG	Electrocardiogram
eCRF	Electronic Case Report Form
ECHO	Echocardiography
ED	Emergency department
EF	Ejection fraction
EOS	End of Study
EU	European Union
FIH	First-in-human
GCP	Good clinical practice
GDMT	Guideline-directed medical therapy
GLP	Good Laboratory Practice
HF	Heart failure
hs-cTn	high-sensitivity cardiac troponin
IB	Investigator’s brochure
ICF	Informed consent form
ICH	International Council for Harmonisation
IEC	Independent Ethics Committee

Abbreviation	Definition
IRB	Institutional review board
IV	Intravenous
IV(W)RS or IXRS	Interactive voice (web) response system
ITT	Intent-to-treat
KCCQ	Kansas City Cardiomyopathy Questionnaire
LV	Left ventricle
LVEF	Left ventricular ejection fraction
LVESVI	Left ventricular end-systolic volume index
MI	Myocardial infarction
miR-132	MicroRNA-132
MoA	Mechanism of action
NOAEL	No-observed-adverse-effect-level
NSTEMI	Non-ST-segment elevation myocardial infarction
NT-proBNP	N-terminal pro B-type natriuretic peptide
[redacted]	[redacted]
PD	Pharmacodynamic(s)
PK	Pharmacokinetic(s)
QTcF	QT interval corrected using Fridericia's formula
SAE	Serious adverse event
SAP	Statistical analysis plan
SARS-CoV-2	Severe acute respiratory syndrome coronavirus 2
SoA	Schedule of Activities
SoC	Standard of Care
SOC	System organ class
STEMI	ST-segment elevation myocardial infarction
$T_{1/2}$	half-life
US	United States
TEAE	Treatment-emergent adverse event
ULN	Upper limit of normal
WOCBP	Woman of childbearing potential

Appendix 2 Regulatory, Ethical, and Study Oversight Considerations

Regulatory and Ethical Considerations

This study will be conducted in accordance with the protocol and with the following:

- Consensus ethical principles derived from international guidelines including the Declaration of Helsinki and Council for International Organizations of Medical Sciences International Ethical Guidelines.
- Applicable International Council for Harmonisation (ICH) Good Clinical Practice (GCP) Guidelines.
- Applicable laws and regulations.

The protocol, protocol amendments, informed consent form (ICF), Investigator Brochure, and other relevant documents (e.g., advertisements) must be submitted to an Institutional Review Board (IRB)/Independent Ethics Committee (IEC) by the Investigator and reviewed and approved by the IRB/IEC before the study is initiated.

Any amendments to the protocol will require IRB/IEC and regulatory authority approval, when applicable, before implementation of changes made to the study design, except for changes necessary to eliminate an immediate hazard to patients.

The Investigator will be responsible for the following:

- Providing written summaries of the status of the study to the IRB/IEC annually or more frequently in accordance with the requirements, policies, and procedures established by the IRB/IEC.
- Notifying the IRB/IEC of serious adverse events (SAEs) or other significant safety findings as required by IRB/IEC procedures.
- Providing oversight of the conduct of the study at the study center and adherence to requirements of 21 Code of Federal Regulations (CFR), ICH guidelines, the IRB/IEC, European regulation 536/2014 for clinical studies (if applicable), and all other applicable local regulations.

After reading the protocol, each Investigator will sign the protocol signature page and send a copy of the signed page to the Sponsor or representative (Appendix 6). The study will not start at any study center at which the Investigator has not signed the protocol.

Financial Disclosure

Investigators and sub-Investigators will provide the Sponsor with sufficient, accurate financial information as requested to allow the Sponsor to submit complete and accurate financial certification or disclosure statements to the appropriate regulatory authorities. Investigators are responsible for providing information on financial interests during the course of the study and for 1 year after completion of the study.

Insurance

The Sponsor has obtained liability insurance, which covers this study as required by local law and/or national regulations and/or ICH guidelines, whichever is applicable. The terms of the insurance will be kept in the study files.

Informed Consent Process

The Investigator or his/her representative will explain the nature of the study to the patient or his/her legally authorized representative and answer all questions regarding the study.

Patients must be informed that their participation is voluntary. Patients or their legally authorized representative will be required to sign a statement of informed consent that meets the requirements of 21 CFR 50, local regulations, ICH guidelines, Health Insurance Portability and Accountability Act requirements, where applicable, and the IRB/IEC or study center.

The medical record must include a statement that written informed consent was obtained before the patient was entered in the study and the date the written consent was obtained. The authorized person obtaining the informed consent must also sign the ICF.

Patients must be re-consented to the most current version of the ICF(s) during their participation in the study.

A copy of the ICF(s) must be provided to the patient or the patient's legally authorized representative.

Patients who are rescreened are required to sign a new ICF.

Data Protection

Patients will be assigned a unique identifier by the Sponsor. Any patient records or datasets that are transferred to the Sponsor will contain the identifier only; patient names or any information which would make the patient identifiable will not be transferred.

The patient must be informed that his/her personal study-related data will be used by the Sponsor in accordance with local data protection law. The level of disclosure must also be explained to the patient.

The patient must be informed that his/her medical records may be examined by Clinical Quality Assurance auditors or other authorized personnel appointed by the Sponsor, by appropriate IRB/IEC members, and by inspectors from regulatory authorities.

Dissemination of Clinical Study Data

The results of the study should be reported within 1 year from the end of the clinical study. Irrespective of the outcome, the Sponsor will submit to the EU database a summary of the results

of the clinical study within 1 year from the end of the clinical study. It shall be accompanied by a summary written in a manner that is understandable to laypersons.

Data Quality Assurance

All patient data relating to the study will be recorded on printed or electronic case report forms (eCRFs) unless transmitted to the Sponsor or designee electronically (e.g., laboratory data). The Investigator is responsible for verifying that data entries are accurate and correct by physically or electronically signing the eCRF.

The Investigator must maintain accurate documentation (source data) that supports the information entered in the eCRF.

The Investigator must permit study-related monitoring, audits, IRB/IEC review, and regulatory agency inspections and provide direct access to source data documents.

The Sponsor or designee is responsible for the data management of this study including quality checking of the data.

Study monitors will perform ongoing source data verification to confirm that data entered into the eCRF by authorized study center personnel are accurate, complete, and verifiable from source documents; that the safety and rights of patients are being protected; and that the study is being conducted in accordance with the currently approved protocol and any other study agreements, ICH GCP, and all applicable regulatory requirements.

Details of study monitoring, including action required due to severe acute respiratory syndrome coronavirus 2 or coronavirus disease 2019, will be included in a separate Study Monitoring Plan.

Records and documents, including signed ICFs, pertaining to the conduct of this study must be retained by the Investigator for 25 years after study completion unless local regulations or institutional policies require a longer retention period. No records may be destroyed during the retention period without the written approval of the Sponsor. No records may be transferred to another location or party without written notification to the Sponsor.

Source Documents

The Investigator/institution should maintain adequate and accurate source documents and study records that include all pertinent observations on each of the study center's patients. Source data should be attributable, legible, contemporaneous, original, accurate, and complete. Changes to source data should be traceable, should not obscure the original entry, and should be explained if necessary (e.g., via an audit trail).

Source documents provide evidence for the existence of the patient and substantiate the integrity of the data collected. Source documents are filed at the Investigator's study center.

Data reported on the eCRF or entered in the eCRF that are transcribed from source documents must be consistent with the source documents or the discrepancies must be explained. The Investigator may need to request previous medical records or transfer records, depending on the study. Also, current medical records must be available.

Definition of what constitutes source data can be found in the Clinical Monitoring Plan.

Study and Study Center Closure

The Sponsor designee reserves the right to close the study center or terminate the study at any time for any reason at the sole discretion of the Sponsor. Study centers will be closed upon study completion. A study center is considered closed when all required documents and study supplies have been collected and a study center closure visit has been performed.

The Investigator may initiate study center closure at any time, provided there is reasonable cause and sufficient notice is given in advance of the intended termination.

Reasons for the early closure of a study center by the Sponsor or Investigator may include but are not limited to:

- Failure of the Investigator to comply with the protocol, the requirements of the IRB/IEC or local health authorities, the Sponsor's procedures, or GCP guidelines.
- Inadequate recruitment of patients by the Investigator.
- Discontinuation of further study treatment development.

Publication Policy

The data generated by this study are confidential information of the Sponsor. The Sponsor will make the results of the study publicly available. The publication policy with respect to the Investigator and study center will be set forth in the Clinical Trial Agreement.

The results of this study may be published or presented at scientific meetings. If this is foreseen, the Investigator agrees to submit all manuscripts or abstracts to the Sponsor before submission. This allows the Sponsor to protect proprietary information and to provide comments.

The Sponsor will comply with the requirements for publication of study results. In accordance with standard editorial and ethical practice, the Sponsor will generally support publication of multicenter studies only in their entirety and not as individual study center data. In this case, a Coordinating Investigator will be designated by mutual agreement.

Authorship will be determined by mutual agreement and in line with International Committee of Medical Journal Editors authorship requirements.

Appendix 3 Clinical Laboratory Tests

The tests detailed in Table 5 will be performed by the central laboratory. Local laboratory results are only required in the event that the central laboratory results are not available in time for either study treatment administration and/or response evaluation. If a local sample is required, it is important that the sample for central analysis is obtained at the same time. Additionally, if the local laboratory results are used to make either a study treatment decision or response evaluation, the results must be entered into the electronic case report form (eCRF).

Protocol-specific requirements for inclusion or exclusion of patients are detailed in Section 5.0 of the protocol.

Additional tests may be performed at any time during the study as determined necessary by the Investigator or required by local regulations.

Investigators must document their review of each laboratory safety report. The results of each test must be entered into the eCRF.

Laboratory/analyte results that could unblind the study will not be reported to study centers or other blinded personnel until the study has been unblinded.

Table 5 Protocol-required Safety Laboratory Assessments

**Laboratory Assessments**	**Parameters**			
Hematology	Platelet Count	RBC Indices:<br>Mean corpuscular volume (MCV)<br>Mean corpuscular hemoglobin (MCH)<br>%Reticulocytes<br>RBC morphology<br>Red cell distribution (RDW)		White Blood Cell Count with Differential:<br>Neutrophils<br>Lymphocytes<br>Monocytes<br>Eosinophils<br>Basophils
	Red Blood Cell (RBC) Count			
	Hemoglobin			
	Hematocrit			
Clinical Chemistry[a]	Blood Urea Nitrogen/Urea<br>Uric acid<br>Cholecystokinin (CK)	Potassium	Aspartate Aminotransferase (AST)/Serum Glutamic-Oxaloacetic Transaminase (SGOT)	Total, indirect, and direct bilirubin<br>Gamma-glutamyltransferase (GGT)<br>Albumin
	Creatinine (glomerular filtration rate calculated using the Chronic Kidney Disease Epidemiology Collaboration equation)	Sodium<br>Bicarbonate	Alanine Aminotransferase (ALT)/Serum Glutamic-Pyruvic Transaminase (SGPT)	Total Protein<br>Lactate dehydrogenase (LDH)

**Laboratory Assessments**	**Parameters**			
	Glucose<br>Hemoglobin A1c (HbA1c)	Calcium	Alkaline phosphatase	
Coagulation	Activated partial thromboplastin time, prothrombin time			
Routine Urinalysis	• Specific gravity, pH, glucose, protein, blood, ketones, bilirubin, urobilinogen, nitrite, leukocyte esterase by dipstick<br>• Microscopic examination (if blood or protein is abnormal)			
Biomarkers	Efficacy-related biomarkers<br>• High-sensitivity cardiac troponin (hs-cTn)<br>• N-terminal pro B-type natriuretic peptide (NT-proBNP)[b]<br>[redacted]			

NOTES: All blood samples should be collected pre-dose at dosing days.

a Details of liver chemistry stopping criteria and required actions and follow-up assessments after liver stopping or monitoring event are given in Section 7.1. All events of ALT ≥ 3 × upper limit of normal (ULN) and bilirubin ≥ 2 × ULN (> 35% direct bilirubin) or ALT ≥ 3 × ULN and international normalized ratio (INR) > 1.5, if INR measured, which may indicate severe liver injury (possible Hy's Law), must be reported as an SAE (excluding studies of hepatic impairment or cirrhosis).

b NT-proBNP values required for eligibility confirmation may be collected at any time post MI either through medical history (e.g., site has collected it as standard of care once the patient came to the hospital), through a local laboratory assessment, or by sending a sample to the central laboratory (if sites use the standard of care sample or the local laboratory sample for eligibility confirmation, no additional sample for central laboratory assessment is needed).

c The exploratory efficacy-related biomarkers will be analyzed by a central laboratory after completion of the study, and data will be stored outside of the main study database. The results will then be included in a separate report.

Appendix 4 Adverse Events: Definitions and Procedures for Recording, Evaluating, Follow-up, and Reporting

See Section 8.3 for additional information regarding adverse events (AEs) and serious adverse events (SAEs).

Definition of AE

**AE Definition**
• An AE is any untoward medical occurrence in a patient or subject, temporally associated with the use of study treatment, whether or not considered related to the study treatment.<br>• NOTE: An AE can therefore be any unfavorable and unintended sign (including an abnormal laboratory finding), symptom, or disease (new or exacerbated) temporally associated with the use of study treatment.

**Events Meeting the AE Definition**
• Any abnormal laboratory test results (hematology, clinical chemistry, or urinalysis) or other safety assessments (e.g., electrocardiogram [ECG], radiological scans, vital signs measurements), including those that worsen from baseline, considered clinically significant in the medical and scientific judgment of the Investigator (i.e., not related to progression of underlying disease).<br>• Exacerbation of a chronic or intermittent pre-existing condition including either an increase in frequency and/or intensity of the condition.<br>• New conditions detected or diagnosed after study treatment administration even though it may have been present before the start of the study.<br>• Signs, symptoms, or the clinical sequelae of a suspected drug-drug interaction.<br>• Signs, symptoms, or the clinical sequelae of a suspected overdose of either study treatment or a concomitant medication. Overdose per se will not be reported as an AE/SAE unless it is an intentional overdose taken with possible suicidal/self-harming intent. Such overdoses should be reported regardless of sequelae.<br>• "Lack of efficacy" or "failure of expected pharmacological action" per se will not be reported as an AE or SAE. Such instances will be captured in the efficacy assessments. However, the signs, symptoms, and/or clinical sequelae resulting from lack of efficacy will be reported as AE or SAE if they fulfill the definition of an AE or SAE.

**Events NOT Meeting the AE Definition**
• Any clinically significant abnormal laboratory findings or other abnormal safety assessments which are associated with the underlying disease, unless judged by the Investigator to be more severe than expected for the patient's condition.<br>• The disease/disorder being studied or expected progression, signs, or symptoms of the disease/disorder being studied, unless more severe than expected for the patient's condition.<br>• Medical or surgical procedure (e.g., endoscopy, appendectomy): the condition that leads to the procedure is the AE.<br>• Situations in which an untoward medical occurrence did not occur (social and/or convenience admission to a hospital).<br>• Anticipated day-to-day fluctuations of pre-existing disease(s) or condition(s) present or detected at the start of the study that do not worsen.<br>• An elective surgery/procedure scheduled to occur during a study will not be considered an AE if the surgery/procedure is being performed for a pre-existing condition and the surgery/procedure has been pre-planned prior to study entry. However, if the pre-existing condition deteriorates unexpectedly during the study (e.g., surgery performed earlier than planned), then the deterioration of the condition for which the elective surgery/procedure is being done will be considered an AE.

Definition of SAE

If an event is not an AE per definition above, then it cannot be an SAE even if serious conditions are met (e.g., hospitalization for signs/symptoms of the disease under study, death due to progression of disease).

**An SAE is defined as any untoward medical occurrence that, at any dose:**
**a) Results in death**
**b) Is life-threatening**<br>• The term 'life-threatening' in the definition of "serious" refers to an event in which the patient was at risk of death at the time of the event. It does not refer to an event, which hypothetically might have caused death, if it were more severe.
**c) Requires inpatient hospitalization or prolongation of existing hospitalization**<br>• In general, hospitalization signifies that the patient has been detained (usually involving at least an overnight stay) at the hospital or emergency ward for observation and/or treatment that would not have been appropriate in the physician's office or outpatient setting. Complications that occur during hospitalization are AEs. If a complication prolongs hospitalization or fulfills any other serious criteria, the event is serious. When in doubt as to whether "hospitalization" occurred or was necessary, the AE should be considered serious.<br>• Hospitalization for elective treatment of a pre-existing condition that did not worsen from baseline is not considered an AE.
**d) Results in persistent disability/incapacity**<br>• The term disability means a substantial disruption of a person's ability to conduct normal life functions.<br>• This definition is not intended to include experiences of relatively minor medical significance such as uncomplicated headache, nausea, vomiting, diarrhea, influenza, and accidental trauma (e.g., sprained ankle) which may interfere with or prevent everyday life functions but do not constitute a substantial disruption.
**e) Is a congenital anomaly/birth defect**

• The term congenital anomaly/birth defect means there is suspect that exposure to a medical product prior to conception or during pregnancy may have resulted in an adverse outcome in the child.
**f) Other situations:**<br>• Medical or scientific judgment should be exercised in deciding whether SAE reporting is appropriate in other situations such as important medical events that may not be immediately life-threatening or result in death or hospitalization but may jeopardize the patient or may require medical or surgical intervention to prevent one of the other outcomes listed in the above definition. These events should usually be considered serious.<br>• Examples of such events include invasive or malignant cancers, intensive treatment in an emergency room or at home for allergic bronchospasm, blood dyscrasias, or convulsions that do not result in hospitalization, or development of drug dependency or drug abuse.

Recording and Follow-up of AE and/or SAE

**AE and SAE Recording**
• When an AE/SAE occurs, it is the responsibility of the Investigator to review all documentation (e.g., hospital progress notes, laboratory reports, and diagnostics reports) related to the event.<br>• The Investigator will then record all relevant AE/SAE information in the electronic case report form (eCRF). Each event must be recorded separately.<br>• It is **not** acceptable for the Investigator to send photocopies of the patient's medical records to the Sponsor or designee in lieu of completion of the AE/SAE eCRF page.<br>• There may be instances when copies of medical records for certain cases are requested by the Sponsor. In this case, all patient identifiers, with the exception of the patient number, will be redacted on the copies of the medical records before submission to the Sponsor.<br>• The Investigator will attempt to establish a diagnosis of the event based on signs, symptoms, and/or other clinical information. Whenever possible, the diagnosis (not the individual signs/symptoms) will be documented as the AE/SAE.
**Assessment of Intensity**
The Investigator will make an assessment of intensity for each AE and SAE reported during the study and assign it to one of the following categories:<br>• Mild: An event that is easily tolerated by the patient, causing minimal discomfort and not interfering with everyday activities.<br>• Moderate: An event that causes sufficient discomfort and interferes with normal everyday activities.<br>• Severe: An event that prevents normal everyday activities. An AE that is assessed as severe should not be confused with a SAE. Severe is a category utilized for rating the intensity of an event; and both AEs and SAEs can be assessed as severe.<br>The terms "severe" and "serious" are not synonymous. Severity refers to the intensity of an AE (e.g., rated as mild, moderate, or severe); the event itself may be of relatively minor medical significance (such as severe headache without any further findings). Severity and seriousness need to be independently assessed for each AE recorded on the CRF

**Assessment of Causality**
• The Investigator is obligated to assess the relationship between study treatment and each occurrence of each AE/SAE. The AE must be characterized as unrelated, unlikely to be related, possibly related, probably related, or unknown (unable to judge).

o **Not related:** An AE that does not follow a reasonable temporal sequence from administration of study drug(s), and/or that can be reasonably explained by other factors such as the subject's preexisting medical condition, underlying disease, concurrent illness, or concomitant medications/therapies.<br>o **Related:** There is a reasonable possibility that an AE is caused by the study drug(s). A plausible temporal sequence exists between the time of administration of the study drug(s) and the development of the AE, or it follows a known response pattern to the study drug(s). The AE cannot be reasonably explained by the known characteristics of the subject's clinical state or other concomitant therapies, or interventions administered to the subject.<br>• The Investigator will use clinical judgment to determine the relationship.<br>• Alternative causes, such as underlying disease(s), concomitant therapy, and other risk factors, as well as the temporal relationship of the event to study treatment administration will be considered and investigated.<br>• The Investigator will also consult the Investigator's Brochure (IB) and/or Product Information, for marketed products, in his/her assessment.<br>• For each AE/SAE, the Investigator must document in the medical notes that he/she has reviewed the AE/SAE and has provided an assessment of causality.<br>• There may be situations in which an SAE has occurred and the Investigator has minimal information to include in the initial report to the Sponsor. However, it is very important that the Investigator always make an assessment of causality for every event before the initial transmission of the SAE data to the Sponsor.<br>• The Investigator may change his/her opinion of causality in light of follow-up information and send a SAE follow-up report with the updated causality assessment.<br>• The causality assessment is one of the criteria used when determining regulatory reporting requirements.

**Follow-up of AEs and SAEs**
• The Investigator is obligated to perform or arrange for the conduct of supplemental measurements and/or evaluations as medically indicated or as requested by the Sponsor or designee to elucidate the nature and/or causality of the AE or SAE as fully as possible. This may include additional laboratory tests or investigations, histopathological examinations, or consultation with other health care professionals.<br>• If a patient dies during participation in the study or during a recognized follow-up period, the Investigator will provide the Sponsor with a copy of any postmortem findings including histopathology.<br>• New or updated information will be recorded in the originally completed eCRF.<br>• The Investigator will submit any updated SAE data to the Sponsor within 24 hours of receipt of the information.

Reporting of SAEs

**SAE Reporting to the Sponsor via Paper SAE Report Form**
• The primary mechanism for reporting an SAE to the Sponsor or designee will be the paper safety event report form. The site will submit the safety event report form, via email, within 24 hours of the Investigator's awareness of the event. Facsimile transmission may be utilized as an alternative mode of submission, if necessary

- Notification of SAE information via telephone does not replace the need for the Investigator to complete, sign and submit the paper SAE report form to the Sponsor or designee within 24 hours of the Investigator's awareness of the event.
- Safety events should be reported to [redacted] at:
  Email: [redacted]
  Phone: [redacted]
  Fax [redacted]

Appendix 5 Contraceptive Guidance and Collection of Pregnancy Information

Definitions:

Woman of Childbearing Potential (WOCBP)

A woman is considered fertile following menarche and until becoming postmenopausal unless permanently sterile (see below).

Women in the following categories are not considered WOCBP

1. Premenarchal
2. Premenopausal female with one of the following:
   a) Documented hysterectomy.
   b) Documented bilateral salpingectomy.
   c) Documented bilateral oophorectomy.
   Note: Documentation can come from the study center personnel's: review of the patient's medical records, medical examination, or medical history interview.
3. Postmenopausal female:
   a) A postmenopausal state is defined as no menses for 12 months without an alternative medical cause. A high follicle stimulating hormone (FSH) level in the postmenopausal range may be used to confirm a postmenopausal state in women not using hormonal contraception or hormonal replacement therapy (HRT). However, in the absence of 12 months of amenorrhea, a single FSH measurement is insufficient.
   b) Females on HRT and whose menopausal status is in doubt will be required to use one of the non-estrogen hormonal highly effective contraception methods if they wish to continue their HRT during the study. Otherwise, they must discontinue HRT to allow confirmation of postmenopausal status before study enrollment.

Contraception Guidance

Male patients

Male patients with female partners of childbearing potential are eligible to participate if they agree to ONE of the following during the protocol-defined time frame in Section 5.1:

- Are abstinent from penile-vaginal intercourse as their usual and preferred lifestyle (abstinent on a long-term and persistent basis) and agree to remain abstinent.
- Agree to use a male condom plus partner use of a contraceptive method with a failure rate of <1% per year as described in Table 6 when having penile-vaginal intercourse with a WOCBP who is not currently pregnant.

In addition, male patients must refrain from donating sperm for the duration of the study and for 30 days after the last dose of study treatment.

Male patients with a pregnant or breastfeeding partner must agree to remain abstinent from penile-vaginal intercourse or use a male condom during each episode of penile penetration during the protocol-defined time frame.

Table 6 Highly Effective Contraceptive Methods

**Highly Effective Contraceptive Methods That Are User Dependent [a]**<br>*Failure rate of <1% per year when used consistently and correctly.*
Combined (estrogen and progestogen containing) hormonal contraception associated with inhibition of ovulation[b]<br>• Oral.<br>• Intravaginal.<br>• Transdermal.
Progestogen only hormonal contraception associated with inhibition of ovulation<br>• Oral.<br>• Injectable.
**Highly Effective Methods That Are User Independent [a]**
Implantable progestogen only hormonal contraception associated with inhibition of ovulation[b]<br>• Intrauterine device (IUD).<br>• Intrauterine hormone-releasing system (IUS).<br>Bilateral tubal occlusion.
**Vasectomized Partner**<br>*A vasectomized partner is a highly effective birth control method provided that the partner is the sole male sexual partner of the WOCBP and the absence of sperm has been confirmed. If not, an additional highly effective method of contraception should be used.*
**Sexual Abstinence**<br>*Sexual abstinence is considered a highly effective method only if defined as refraining from heterosexual intercourse during the entire period of risk associated with the study treatment. The reliability of sexual abstinence needs to be evaluated in relation to the duration of the study and the preferred and usual lifestyle of the patient.*
NOTES:<br>[a] Typical use failure rates may differ from those when used consistently and correctly. Use should be consistent with local regulations regarding the use of contraceptive methods for patients participating in clinical studies.<br>[b] Hormonal contraception may be susceptible to interaction with the study treatment, which may reduce the efficacy of the contraceptive method. In this case, 2 highly effective methods of contraception should be utilized during the treatment period and for at least 30 days after the last dose of study treatment.

Collection of Pregnancy Information

Male patients with partners who become pregnant

The Investigator will attempt to collect pregnancy information on any male patient’s female partner who becomes pregnant while the male patient is in this study. This applies only to male patients who receive CDR132L.

After obtaining the necessary signed informed consent from the pregnant female partner directly, the Investigator will record pregnancy information on the appropriate form and submit it to the Sponsor within 24 hours of learning of the partner's pregnancy. The female partner will also be followed to determine the outcome of the pregnancy. Information on the status of the mother and child will be forwarded to the Sponsor. Generally, the follow-up will be no longer than 6 to 8 weeks following the estimated delivery date. Any termination of the pregnancy will be reported regardless of fetal status (presence or absence of anomalies) or indication for the procedure.

Reporting Pregnancy Information

Email transmission of the paper Pregnancy Report Form is the preferred method to transmit safety event information to [redacted] with facsimile as a back-up method, if necessary.

Safety events should be reported to [redacted] at:

Email: [redacted]
Phone: [redacted]
Fax: [redacted]

Appendix 6 Signature of Investigator

PROTOCOL TITLE: P**h**ase 2, Multicenter, Randomized, Parallel, 3-arm, Placebo-controlled Study to Assess E**f**ficacy and Safety of CD**R**132L in Pati**e**nts with Reduced Left **V**entricular **E**jection F**r**ac**t**ion (≤ 45%) After Myocardial Infarction (**HF-REVERT**)

PROTOCOL NO: CDR132L – P2-01

VERSION: Original Protocol

This protocol is a confidential communication of Cardior Pharmaceuticals GmbH. I confirm that I have read this protocol, I understand it, and I will work according to this protocol. I will also work consistently with the ethical principles that have their origin in the Declaration of Helsinki and that are consistent with Good Clinical Practices and the applicable laws and regulations. Acceptance of this document constitutes my agreement that no unpublished information contained herein will be published or disclosed without prior written approval from Cardior Pharmaceuticals GmbH.

Instructions to the Investigator: Please SIGN and DATE this signature page. PRINT your name, title, and the name of the study center in which the study will be conducted. Return the signed copy to Cardior Pharmaceuticals GmbH.

I have read this protocol in its entirety and agree to conduct the study accordingly:

Signature of Investigator: ____________________________ Date: ____________

Printed Name: ____________________________

Investigator Title: ____________________________

Name/Address of Center: ____________________________

____

____

Cardior Pharmaceuticals GmbH
Protocol #: CDR132L-P2-01

Phase 2, Multicenter, Randomized, Parallel, 3-arm, Placebo-controlled Study to Assess Efficacy and Safety of CDR132L in Patients with Reduced Left Ventricular Ejection Fraction (≤ 45%) After Myocardial Infarction (HF-REVERT)

Statistical Analysis Plan

Version 2.0
Document last saved: 11-Oct-2024 2:25 AM

Prepared by:

Redacted statistical analysis plan includes redaction of personal identifiable and company confidential information.

Signed by [redacted]
[redacted] | I approve this document
11-Oct-2024 | 05:28:58 EDT
AF08918CD2FC4F6E90CCCCCC535143DE

[redacted]

[redacted]

Approved by:

Signiert von [redacted]
[redacted] | Ich genehmige dieses Dokument
11-Okt-2024 | 03:55:43 PDT
F709870B76EA4403ACE259DFE529BFFD

[redacted]

Signiert von [redacted]
[redacted] | Ich genehmige dieses Dokument
11-Oct-2024 | 02:29:09 PDT
5AA80916C7854806B52E9F545E4CCB02

[redacted]

CONTENTS

Abbreviations 4

I.Introduction 6

A. Background 6
B. Protocol and Amendment History 6
C. Changes to Planned Analyses from Protocol 7

II.Protocol Objectives 7

A. Primary Objective 8
B. Secondary Objectives 8
C. Exploratory Objectives 8

III.Study Endpoints 9

A. Primary Endpoint 9
B. Secondary Endpoints 9
C. Exploratory Endpoints 10

IV.Study Design 11

A. Design Overview 11
B. Sample Size Calculation 15
C. Treatment Randomization 16

V.General Analytical Considerations 16

A. Data Sources 16
B. Definition of Baseline 16
C. Analysis Visit Window 17
D. Missing Data 18
E. Multiple Study Centers 19
F. Covariate Adjustment in Primary Analysis 19
G. Sample Size Reassessment 19
H. Interim Analyses or Timing of Analyses 19
I. Test Sizes 20
J. Multiple Comparisons 20
K. Analysis Populations 21
L. Definition of Subgroups 23
M. Data Display Characteristics 24

VI.Subject Accountability 24

A. Subject Characteristics 24
B. Disposition 26
C. Protocol Deviations 26
D. Visit Attendance 26

VII.Efficacy Analyses 27

A. Efficacy Outcomes 27
B. Primary Efficacy Outcome Analysis 28
C. Secondary Efficacy Analyses 31
D. Exploratory Efficacy Analysis 32
E. Subgroup analyses 34

VIII.Safety Analyses 34

A. Exposure 34
B. Adverse Events 34
C. Clinical Laboratory Results 36
D. Vital Signs 36
E. 12-Lead Electrocardiogram (ECG) 37
F. Physical Examination 37
G. Concomitant Medications 37
H. Immunogenicity Analysis 38

IX.References 39

X.Appendix 40

A. Kansas City Cardiomyopathy Questionnaire (KCCQ) 40

ABBREVIATIONS

Abbreviation	Definition
AE	Adverse Event
ANCOVA	Analysis of Covariance
ATC	Anatomical-Therapeutic-Chemical
BMI	Body Mass Index
CI	Confidence Interval
CM	Concomitant Medication
COVID-19	Coronavirus disease 2019
ECHO	Echocardiography
eCRF	Electronic Case Report Form
EF	Ejection Fraction
EOS	End of Study
EOT	End of Treatment
ET	Early Termination
GDMT	Guideline-Directed Medical Therapy
HDU	High-Dependency Care Units
HF	Heart Failure
ICU	Intensive Care Unit
ITT	Intent-to-treat
IV	Intravenous
IVRS/IWRS/IXRS	Interactive Voice/Web Response System
KCCQ	Kansas City Cardiomyopathy Questionnaire
LAVI	Left atrial volume index
LOCF	Last Observation Carried Forward
LV	Left ventricle
LVEF	Left ventricular ejection fraction
LVESVI	Left ventricular end-systolic volume index
MedDRA	Medical Dictionary for Regulatory Activities
MI	Myocardial infarction
miR-132	MicroRNA-132
MNAR	Missing Not at Random
MRI	Magnetic Resonance Imaging
NSTEMI	Non ST-segment elevation myocardial infarction
NT-proBNP	N-terminal pro B-type natriuretic peptide
[redacted]	[redacted]
PK	Pharmacokinetics

PP	Per-Protocol
PT	Preferred Term
SAE	Serious Adverse Event
SAP	Statistical Analysis Plan
SoA	Schedule of Activities
SOC	System Organ Class
STEMI	ST-segment elevation myocardial infarction
TEAE	Treatment Emergent AE(s)
WHO-DD	World Health Organization Drug Dictionary

I. Introduction

A. Background

Current state-of-the-art Heart Failure (HF) pharmacotherapy is largely focused on symptomatic management. The recommended guideline-directed medical therapy (GDMT) includes diuretics, angiotensin-converting enzyme inhibitors, angiotensin II receptor blockers, beta-blockers, aldosterone antagonists, angiotensin receptor neprilysin inhibitors, and ivabradine. Mechanical circulatory support and heart transplant is reserved for the treatment of patients with severe HF with reduced ejection fraction (EF) who have failed GDMT. Novel, efficient, disease-halting therapeutics that reduce mortality and hospitalization are urgently needed to offer curative hope for these patients. CDR132L is a unique miRNA-based next generation drug in HF, with the potential to improve patient care and thereby reduce the financial burden of HF care.

Several completed pre-clinical studies and the completed first-in-human (FIH) study assessing the pharmacokinetics (PK), pharmacodynamics (PD), pharmacology, safety pharmacology, and toxicological profile of CDR132L have demonstrated that the safety profile is adequate to progress with clinical development.

B. Protocol and Amendment History

This Statistical Analysis Plan (SAP) is based on Study Protocol Amendment 3.1 dated 16 May 2023.

Version	Approval Date	Salient Changes, if any*
Protocol	07 December 2021	n/a
Amendment 1	15 July 2022	n/a
Amendment 2_Greek	03 November 2022	n/a
Amendment 2_UK	03 November 2022	n/a
Amendment 3	30 March 2023	n/a
Amendment 3.1	16 May 2023	n/a

* Changes expected to require accommodation in analysis plan.

This SAP will govern the analysis of data from this study. The plan may be modified until first database lock and unblinding for the 6-month

primary efficacy and safety analyses. Any amendments to the SAP will be documented, with final amendments completed before unblinding of the data for the primary analysis. Any deviations from the analysis plan after unblinding of the data for the primary analysis will be documented as such in the study report. A second database lock will be performed once the last patient has completed last visit (Day 360/Month 12) and data cleaning is complete. The clinical study report will be written following the completion of the study (Day 360/Month 12).

C. Changes to Planned Analyses from Protocol

The following changes from the protocol planned analyses and their rationale are noted in this plan:

- A modified Intent-to-Treat (mITT) analysis set has been defined to include all randomized subjects who receive at least 1 dose of study drug (CDR132L or placebo).
  <u>Rationale:</u>
  For analysis purposes, the mITT is added in the SAP.

- The analysis of the efficacy outcome measure will be performed using the mITT population.
  <u>Rationale:</u>
  From a medical perspective, it makes more sense to include subjects in the primary collective who received the study drug in this early phase trial, especially taking the small patient numbers into consideration.

- Additional test processes are added when the primary endpoints are successful. Details can be seen in Section D.J.Multiple Comparisons.
  <u>Rational:</u>
  Additional tests are added because we want to get the most out of the study in terms of understanding the different elements of efficacy and connected endpoints to finally generate the right hypotheses for the upcoming clinical trials.

II. Protocol Objectives

A. Primary Objective

To assess the efficacy of 2 dose levels ([redacted] and [redacted] mg/kg) of CDR132L compared with placebo administered in 3 single IV doses given 28-days apart in subjects with reduced Left Ventricular Ejection Fraction (LVEF) ≤45% after Myocardial Infarction (MI) (STEMI or NSTEMI) as add-on therapy to Standard of Care treatment.

B. Secondary Objectives

Confirmatory secondary objectives:

- To assess the efficacy of the pooled dose levels ([redacted] and [redacted] mg/kg) of CDR132L compared with placebo in LVEF (%) at month 6.
- To assess the efficacy of the pooled dose levels ([redacted] and [redacted] mg/kg) of CDR132L compared with placebo in NT-proBNP (pg/mL) (on a logarithmic scale) at month 6.
- To assess the efficacy of the pooled dose levels ([redacted] and [redacted] mg/kg) of CDR132L compared with placebo in global longitudinal strain at month 6.
- To assess the efficacy of the pooled dose levels ([redacted] and [redacted] mg/kg) of CDR132L compared with placebo in Overall Summary score in KCCQ at month 6.

Supportive secondary objectives:

- To assess the safety of 2 dose levels ([redacted] and [redacted] mg/kg) of CDR132L compared with placebo
- To assess the effects of CDR132L compared with placebo on cardiac function
- To assess the effects of CDR132L compared with placebo on efficacy-related biomarkers
- To assess the effects of CDR132L compared with placebo on subject well-being

C. Exploratory Objectives

[redacted]

III. Study Endpoints

A. Primary Endpoint

Percent change from baseline (screening to occur at least 3 days (up to 14 days) after MI diagnosis as measured by Echocardiography (ECHO) [central laboratory]) in Left Ventricular End-systolic Volume Index (LVESVI) at Month 6.

B. Secondary Endpoints

Confirmatory secondary endpoints:

- Absolute change from baseline in LVEF (%) at Month 6 with pooled dose levels ([redacted] and [redacted] mg/kg).
- Absolute change from baseline in N-terminal pro B-type natriuretic peptide (NT-proBNP pg/mL) at Month 6 with pooled dose levels ([redacted] and [redacted] mg/kg).
- Absolute change from baseline in global longitudinal strain (%) at Month 6 (absolute) with pooled dose levels ([redacted] and [redacted] mg/kg).
- Absolute change from baseline in Overall Summary score in KCCQ at Month 6 (absolute) with pooled dose levels ([redacted] and [redacted] mg/kg).

Supportive secondary endpoints:

- Change from baseline in LVEF (%) (absolute/relative) at Months 3, 6, and 12
- Change from baseline in absolute/relative values over time for the NT-proBNP (pg/mL) at Days 2 to 5, Months 1, 2, 3, 6, and 12.
- Change from baseline in LVESVI at Month 3 (absolute/relative), Month 6 (absolute), and Month 12 (absolute/relative)
- Frequency and nature of adverse events during the course of the trial, and abnormalities in clinical laboratory assessments, vital signs, physical examination, electrocardiograms (ECGs), and urinalysis at Months 3, 6, and 12

- Change from baseline in absolute/relative Troponin T (ng/L) at Months 3, 6, and 12

- Well-being as evaluated by change from baseline at Months 6 and 12 in the following parameters:
    - Mean Kansas City Cardiomyopathy Questionnaire (KCCQ - overall summary score) score and mean scores of subdomains (symptom burden, physical limitation, and quality of life)

C. Exploratory Endpoints

[redacted]

IV. Study Design

A. Design Overview

This is a Phase 2, multicenter, randomized, parallel, 3-arm, placebo-controlled study to assess efficacy and safety of CDR132L in subjects with reduced LVEF (≤ 45%) after MI. This study consists of a Screening Period (to occur at least 3 days after MI diagnosis), a 6-month Double-blind Period, and a 6-month Prolonged Follow-up Period with the End of Study (EOS) Visit at Day 360/Month 12. All subjects will be required to attend the study visits as described in the Schedule of Activities (SoA).

Cardior Pharmaceuticals GmbH / Protocol: CDR132L-P2-01
[redacted](Confidential) Project # QZA91424

Table 1 Schedule of Activities

Procedures	Screening Period (To occur at least 3 days after MI diagnosis)	Double-blind Period (Day 1 to Day 180/Month 6)							Prolonged Follow-up Period (6 months)
		Study Drug Treatment (Day 1, Day 29, and Day 57)					Follow-up		
**Visit**		Randomization (To occur no later than 14 days after MI diagnosis)	**1**	**2**	**3**	**4**	**5**	**6**	**EOS/ET**
**Study Day**			**Day 1 (pre-dose)**	**Days 2 to 5**	**Day 29**	**Day 57**	**Day 90**	**Day 180**	**Day 360**
**Window (days)**					**± 2**	**± 2**	**± 7**	**± 7**	**± 7**
**Month**					**Month 1**	**Month 2**	**Month 3**	**Month 6**	**Month 12**
Informed consent	X								
Demographic data and medical history[a]	X[a]								
Inclusion/exclusion criteria	X								
Randomization		X							
Study drug administration[b]			X[b]		X	X			
Prior/concomitant medications	X		X	X	X	X	X	X	X
**EFFICACY**									
Echocardiography[c]	X[c]						X	X	X
KCCQ	X						X	X	X
hs-cTn	X		X	X	X	X	X	X	X
NT-proBNP	X[d]		X	X	X	X	X	X	X
**SAFETY**									
Adverse event assessments	X		X	X	X	X	X	X	X

Procedures	Screening Period (To occur at least 3 days after MI diagnosis)	Double-blind Period (Day 1 to Day 180/Month 6)							Prolonged Follow-up Period (6 months)
		Study Drug Treatment (Day 1, Day 29, and Day 57)					Follow-up		
**Visit**		**Randomization (To occur no later than 14 days after MI diagnosis)**	**1**	**2**	**3**	**4**	**5**	**6**	**EOS/ET**
**Study Day**			**Day 1 (pre-dose)**	**Days 2 to 5**	**Day 29**	**Day 57**	**Day 90**	**Day 180**	**Day 360**
**Window (days)**					**± 2**	**± 2**	**± 7**	**± 7**	**± 7**
**Month**					**Month 1**	**Month 2**	**Month 3**	**Month 6**	**Month 12**
Injection site reaction monitoring			X (post-dose)		X	X			
Physical examination	X		X	X	X	X	X	X	X
Vital signs	X		X	X	X	X	X	X	X
12-lead electrocardiogram	X		X		X	X	X	X	X
Body weight	X			X	X	X	X	X	X
Hematology, biochemistry, coagulation[e]	X		X	X	X	X	X	X	
Urinalysis[e]	X		X		X		X	X	
**EXPLORATORY EFFICACY**									

[redacted]

Procedures	Screening Period (To occur at least 3 days after MI diagnosis)	Double-blind Period (Day 1 to Day 180/Month 6)							Prolonged Follow-up Period (6 months)
		Study Drug Treatment (Day 1, Day 29, and Day 57)					Follow-up		
Visit		Randomization (To occur no later than 14 days after MI diagnosis)	1	2	3	4	5	6	EOS/ET
Study Day			Day 1 (pre-dose)	Days 2 to 5	Day 29	Day 57	Day 90	Day 180	Day 360
Window (days)					± 2	± 2	± 7	± 7	± 7
Month					Month 1	Month 2	Month 3	Month 6	Month 12

[redacted]

Abbreviations: COVID-19 = coronavirus disease 2019; ECHO = echocardiography; ED = emergency department; EOS = end of study; ET = early termination; hs-cTn=high-sensitivity cardiac troponin; IV = intravenous; KCCQ = Kansas City Cardiomyopathy Questionnaire; NT-proBNP = N-terminal pro B-type natriuretic peptide; NYHA = New York Heart Association; MI = myocardial infarction; SoC = Standard of Care.

Note: All blood samples should be collected pre-dose at dosing days.

a. Events and diseases will be recorded for up to 6 months prior to screening. COVID-19 infections and vaccinations will be recorded regardless of their time of occurrence.

b. On Day 1 (preferably within one day after randomization but no later than 4 days after randomization) patients will receive CDR132L 5 mg/kg, CDR132L 10 mg/kg, or placebo IV. The second IV dose of the patient's assigned treatment will be administered on Day 29 ± 2 days, and the third IV dose on Day 57 ± 2 days. For doses administered in an outpatient setting, the patient should be observed for at least 30 minutes after dosing.

c. The screening ECHO will be performed at least 3 days after MI diagnosis; all eligibility criteria must be confirmed no later than 14 days after MI diagnosis.

d. At screening, sample is to be collected as early as possible preferably on the same day as the ECHO. NT-proBNP values required for eligibility confirmation may be collected at any time post MI either through medical history (e.g., site has collected it as SoC once the patient came to the hospital), through a local laboratory assessment, or by sending a sample to the central laboratory (if sites use the SoC sample or the local laboratory sample for eligibility confirmation, no additional sample for central laboratory assessment is needed).

e. See Appendix 3 in protocol for more information.

Approximately 70 Investigators and study sites participated in this study. Approximately 280 subjects were enrolled/randomized into the study in a 1:1:1 ratio (approximately 90 subjects each, in [redacted] mg/kg, [redacted] mg/kg, and placebo groups). The study schema is presented in Figure 1.

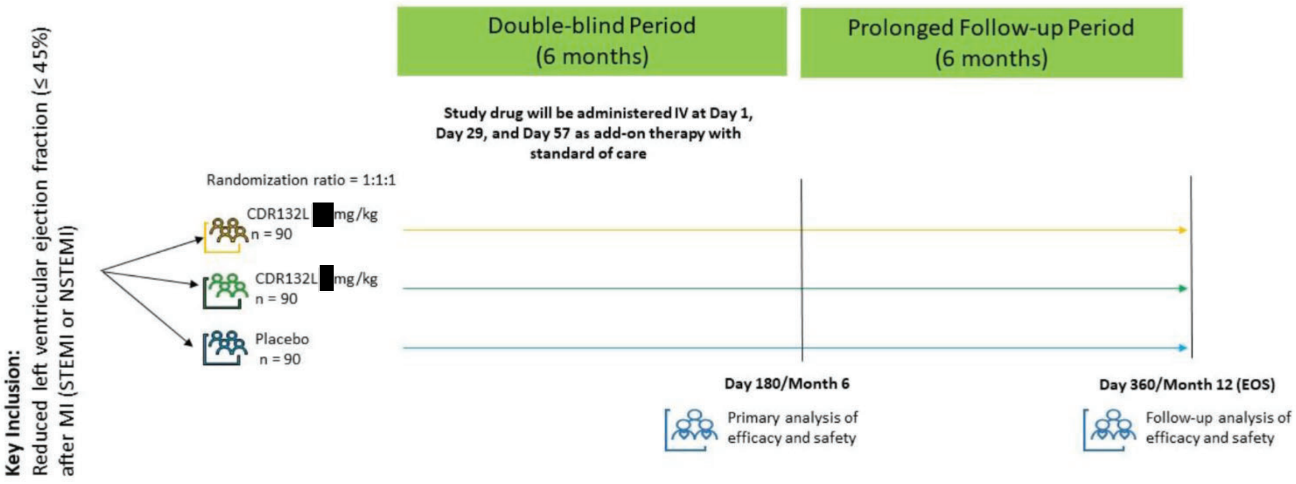

Figure 1 Study Schema

B. Sample Size Calculation

For the primary endpoint of percent (%) change from baseline LVESVI at month 6, a sample size calculation for the comparison of the two treatment arms ([redacted] and [redacted] mg/kg CDR132L v.s. Placebo) is performed by using the two-sample t-test. The test level is set to 2.5% (one-sided). Assumptions are displayed in Table 2 below.

Test 1 (CDR132L [redacted] mg/kg v.s. Placebo)

H_{01}: $\mu_{T10} \le \mu_P$ versus H_{11}: $\mu_{T10} > \mu_P$

Test 2 (CDR132L [redacted] mg/kg v.s. Placebo)

H_{02}: $\mu_{T5} \le \mu_P$ versus H_{12}: $\mu_{T5} > \mu_P$

**Left ventricular end-systolic index**	**Mean (change in %)**	**Standard deviation (%)**	**Power**
Placebo response	1	9	
[redacted] mg/kg	6	9	
[redacted] mg/kg	5	9	
N = 90 per group	Step 1		96.0%
	Step 2		84.3%
	Overall		80.9%

Table 2 Sample Size Calculation Assumptions

Step 1 ([redacted] mg/kg versus placebo):
A sample size of 90 in each group will have 96.0% power to detect a difference in means of 5 (the difference between a Group 1 mean, μ_1, of 6 and a Group 2 mean, μ_2, of 1) assuming that the common standard deviation is 9 using a 2-group t-test with a 2.5% one-sided significance level.

Step 2 ([redacted] mg/kg versus placebo):
A sample size of 90 in each group will have 84.33% power to detect a difference in means of 4 (the difference between a Group 1 mean, μ_1, of 5 and a Group 2 mean, μ_2, of 1) assuming that the common standard deviation is 9 using a 2-group t-test with a 2.5% one-sided significance level.

Including 90 subjects per group (270 total) will lead to a power of 96.0% for the first step ([redacted] mg/kg vs placebo, difference in means of 5) and 84.3% for the second step ([redacted] mg/kg vs placebo, difference in means of 4). In of the hierarchical test procedure, resulting in an overall power of at least 80 %. Ten additional subjects should be included to compensate for early dropouts.

C. Treatment Randomization

All subjects will be centrally randomized using an Interactive Voice/Web Response System (IVRS/IWRS/IXRS). Subjects will be randomly assigned in a 1:1:1 ratio to receive study treatment.

Randomization will be stratified by:
- Age groups (<60 years, ≥60 years)
- Location of infarction (anterior, non-anterior)

V. General Analytical Considerations

A. Data Sources

Data are recorded on electronic case report forms (eCRFs). Central laboratory data and ECHO data results will be provided via electronic data transfers (details in Protocol Section 8.2.1).

B. Definition of Baseline

Unless otherwise specified, baseline is defined as the last non-missing measurement taken prior to reference start date (first administration of study medication), including unscheduled assessments. In the case where the last non-missing measurement and the reference start date coincide, collection time, where available, will be compared with the first dose time to determine whether the measurement is pre-baseline or post-baseline. If time is not available, the measurement will be considered pre-baseline, but injection site reaction commencing on the reference start date will be considered post-baseline. If first dose date is not available, the randomization date for untreated subjects (or day 1 visit date for treated subjects) will be used as the proxy reference start date in defining baseline assessments.

Change from baseline is calculated as post-baseline values minus baseline values. Percentage (relative) change from baseline is calculated as ratio of change from baseline and baseline expressed as a percentage.

C. Analysis Visit Window

All efficacy and safety endpoints will be analyzed according to the nominal/assigned visits (as described in Protocol Section 1.3) except for assessments collected on early termination and unscheduled visits. Early termination (ET) and unscheduled visits will be re-numbered to analysis visits based on their windowed visits defined by actual study day in Table 3. If more than one visit or assessment (scheduled or unscheduled) occurs within a single visit window day and if one visit is scheduled and one is unscheduled, the assessment will take the scheduled one. If the two visits are equidistant from the target day and if both visits are scheduled or both are unscheduled, the visit with later date and time will be used.

Visit	Target Day	Analysis Window
Day 1	1	On or prior to Day 1
Days 2-5	3	Day 2 – Day 16
Day 29 / Month 1	29	Day 17 – Day 43
Day 57 / Month 2	57	Day 44 – Day 73
Day 90 / Month 3	90	Day 74 – Day 135
Day 180 / Month 6	180	Day 136 – Day 270
Day 360 / Month 12	360	Day 271 – Day 360

Table 3 Analysis Visit Window

Certain efficacy and safety endpoints are not scheduled to be assessed at each post-baseline. If the date of an ET or unscheduled visit does not fall into the analysis window of any scheduled visit, the assessment will be

mapped to the scheduled post-baseline visit closest to the assessment date.

D. Missing Data

1. Partial and Missing Dates in AE and CM

Partial dates may be entered on the CRF for Adverse Events (AEs) and prior and concomitant medications (CMs). Dates from these forms will be reported in listings as collected without any imputation. However, reported partial information will be utilized in classification of TEAEs and prior and concomitant medications according to the rules described below.

- AEs that cannot be definitely determined as occurring prior to study drug administration will be counted as TEAEs unless either the partial start date/time or a partial or complete end date/time documents the AE as occurring prior to treatment.
- AE onset dates with missing day and non-missing month will be assumed to occur on the first day of the non-missing month, except for AEs occurring in the month and year of first dosing of study drug, in which case the date will be assumed to be the date of first dosing of study drug and the AE will be considered as TEAE.
- AE onset dates with missing day and month will be assumed to occur on the first day of the non-missing year (i.e., January 1), except for AEs occurring in the year of first dosing of the study drug, in which case the date will be assumed to be the date of the first dosing of study drug and the AE will be considered as TEAE.

Partial dates entered in the prior and CM form will be imputed for the purposes of determining whether the record is a concomitant or prior medication based on the following:

- Medications that are not ongoing and have a medication stop date with a missing day and non-missing month will be assumed to occur on the last day of the non-missing month
- Medications that are not ongoing and have a medication stop date with missing day and month will be assumed to occur on the last day of the non-missing year (i.e., December 31)

2. Missing Safety Data

Sites will be instructed to especially pay attention to complete the safety section of the eCRF and if possible, collect to retrieve safety data among subjects who withdraw from the study early.

Missing safety data will not be replaced with imputed values, with the exception of missing severity or relationship to study medication for AE:

- Missing relationship to study medication will not be imputed and will be summarized as a separate "missing" category where applicable.
- Missing intensity will be summarized as a separate "missing" category.

3. Missing Efficacy Data

Sites will be instructed to minimize dropouts and if possible, collect to retrieve efficacy data among subjects who withdraw from the study early.

Missing efficacy data (including those due to early discontinuations) will be imputed by having the last non-missing visit value carried forward (LOCF) as detailed below.

For the primary efficacy endpoint and both LVEF (%) and NT-proBNP (pg/mL) secondary endpoints, multiple imputation (MI) based sensitivity analysis will be performed as detailed in Section VII.B.3.

E. Multiple Study Centers

The centers will be considered as a covariance factor and added into the model for all analysis. The list of center groups for pooling the very small centers will be determined before unblinding.

F. Covariate Adjustment in Primary Analysis

Covariate adjustment will be addressed in relevant sections below, as appropriate.

G. Sample Size Reassessment

Not applicable.

H. Interim Analyses or Timing of Analyses

No interim analysis for efficacy is planned.

Primary analysis will be done once the last subject has completed the 6-month time point (first database lock). Follow-up data after this time point will be analyzed separately once the last subject has completed last visit (Month 12; second database lock). A clinical study report will be written following the completion of the study (Day 360/Month 12).

A DSMB is implemented for this study and reviews unblinded safety data in format and frequency as described in a separate DSMB Charter.

I. Test Sizes

Any tested hypotheses will be tested against 1-sided alternatives, using procedures that provide an expected probability of Type I error (α) of 0.025 to show superiority.

J. Multiple Comparisons

For the primary efficacy endpoint, the comparison will be done for the [redacted] mg/kg dose group versus placebo, followed by [redacted] mg/kg versus placebo within a hierarchical two-step test procedure. The aim is to show superiority of the [redacted] mg/kg dose group (μ_{T10}) and the [redacted] mg/kg dose group (μ_{T5}) in comparison to placebo (μ_P) within a hierarchical test procedure with 2 steps:

Step 1
H_{01}: $\mu_{T10} \leq \mu_P$ versus H_{11}: $\mu_{T10} > \mu_P$,
where μ indicates the treatment effect in percent change from baseline. (i.e H_{01}: $PCHG_{T10} \geq PCHG_P$ versus H_{11}: $PCHG_{T10} < PCHG_P$)

If Step 1 is successful, then proceed to Step 2. If Step 1 is not successful, then do not proceed to Step 2.

Step 2
H_{02}: $\mu_{T5} \leq \mu_P$ versus H_{12}: $\mu_{T5} > \mu_P$,
where μ indicates the treatment effect in percent change from baseline. (i.e H_{02}: $PCHG_{T5} \geq PCHG_P$ versus H_{12}: $PCHG_{T5} < PCHG_P$)

To show superiority, the effect of CDR132L dose groups on the percent change in LVESVI must be larger (i.e., higher decrease in percent change from baseline) than the effect within the placebo group.

If both Step 1 and Step 2 are successful (i.e. statistically significant effects of both doses on the primary efficacy endpoint were established), then the following confirmatory secondary efficacy endpoints are tested on the

pooled data of both doses (5 mg/kg and 10 mg/kg) against placebo to show the superiority:

- Change from baseline in LVEF (%) at Month 6
  (H_{03a}: $CHG_T \leq CHG_P$ versus H_{13a}: $CHG_T > CHG_P$)

- Change from baseline in NT-proBNP (pg/mL) (on a logarithmic scale) at Month 6
  (H_{03b}: $CHG_T \geq CHG_P$ versus H_{13b}: $CHG_T < CHG_P$)

[redacted]

- Well-being as evaluated by change from baseline at Month 6 in the following parameters:
  - Mean Kansas City Cardiomyopathy Questionnaire (KCCQ) score (i.e. Overall Summary score)

  (H_{03d}: $CHG_T \leq CHG_P$ versus H_{13d}: $CHG_T > CHG_P$)

Control of the type I error rate across these secondary efficacy endpoints is achieved by application of the Hochberg procedure.

K. Analysis Populations

Three analysis populations will be used for various analyses. Table 4 Analysis Sets illustrates the different populations and how these are defined.

**Analysis Set**	**Description**
Entered Analysis Set	All subjects who sign the informed consent form.
Intent-to-treat (ITT)	All subjects in the Entered Analysis Set who are randomized to study drug. Subjects will be analyzed according to their randomized study treatment.
Modified Intent-to-treat (mITT)	All randomized subjects who receive at least 1 dose of study drug (CDR132L or placebo). Subjects will be analyzed according to the treatment they were randomized to.

Safety	All randomized subjects who receive at least 1 dose of study drug (CDR132L or placebo) and have at least 1 post-dose safety assessment. Subjects will be analyzed according to the treatment they actually received.
Per-protocol (PP)	All subjects from ITT population that have completed treatment and 6-month visit without any major protocol deviations that could affect the validity of primary and secondary efficacy assessments. Subjects may be excluded from PP population due to any of the following criteria:<br>• Not compliant (in terms of study drug and study schedule)<br>• Used prohibited therapies (as per protocol section 6.5.1)<br>• Had other protocol violation that might affect the efficacy assessment<br><br>Per-protocol population will be determined by blinded review of data prior to unblinding. Subjects will be analyzed according to the treatment they actually received.

Table 4 Analysis Sets

The following table illustrates the relationship between each population and the analyses for which the data from the population will be used.

Analysis Population	Type of Analysis				
	Subject Disposition	Baseline	Efficacy	Safety	Exposure/ Compliance
Entered Analysis Set	V				
ITT	V	V			
mITT	V	V	V		
Safety	V			V	V
Per Protocol	V	V	V		

Two observation periods are defined for each subject:

- In-trial: The in-trial period is defined as the time from the randomization to the date of last contact with the trial site.

- On-treatment period: The on-treatment period is defined as the duration from the initial administration of the study treatment to the final administration. For the evaluation of adverse events (AEs), this period extends from the first administration of the study treatment to 30 days after the last administration.

L. Definition of Subgroups

A subgroup analysis for the primary endpoint of % change from baseline at Month 6 will be performed. The changes in LVESVI, LVEF (%), and NT-proBNP (pg/mL) from baseline (relative/absolute) at Month 6 and month 12 will be performed.

LVESVI, LVEF (%), and NT-proBNP (pg/mL) will be summarized descriptively by treatment group for the following prespecified subgroups. Further subgroups might be defined for additional exploratory analyses post hoc.

- Age group from CRF (less than 60 years, 60 years and older)
- Sex (Female, Male)
- MI type (STEMI, NSTEMI)
- Time of first treatment after MI event (d3-7 versus d7-14)
- Patients with and without treatment of SGLT2 inhibitors
- Disease severity acc. to [redacted]
    - LVEF (%) (<= median, > median from mITT)
    - NT-proBNP (pg/mL) (<= median, > median from mITT)
- Location of infarction (anterior, non-anterior)
- Diabetes Status (Diabetes/non-Diabetes)
- Troponin central baseline results (<= median, > median from mITT)
- Chronic HF status (Patients with, Patients without)
    - Patients with chronic heart failure or reduced LVEF in medical history prior to MI index event.
- Renal function
    - eGFR (mL/min/1.72m^2) Group 1 (“<=45”, “>45, <=60”, “>60”)
    - eGFR (mL/min/1.72m^2) Group 2 (“<=60”, “>60”)
- Atrial fibrillation status (Patients with, Patients without)
- Septum thickness (<= median, > median from mITT)
    - The value will be obtained from an [redacted] with the assessment ‘Parasternal long axis view: Interventricular septum thickness.”

M. Data Display Characteristics

Data displays produced for this study will include three types: summary tables, data listings, and figures. Unless stated otherwise, data listings will be produced for all recorded data. Summary tables will be produced as specified in following sections. Additional data listings will be produced for outcome measures that involve extensive procedures to derive the analyzed outcomes. Figures will be produced when specified in sections to follow.

Data listings will simply list the data recorded on the CRF or derived for each subject. They will be ordered by treatment, site, subject number, and time of assessment. When expedient, additional levels of ordering hierarchy may reflect subsets of assessments within subject. Data listings will not display subject initials.

The summary statistics displayed will be a function of the type of data associated with the summarized assessment. Unless stated otherwise in relevant sections to follow, continuous data will be summarized with the number of non-missing values, mean, standard deviation, minimum, median, and maximum. Categorical data will be summarized with the number of non-missing values and the numbers of values equal to each of the possible values. Percentages of subjects with each of the possible values will be calculated from the number of subjects in the corresponding analysis population, unless stated otherwise. Some continuous variables may also be grouped into categorical levels and evaluated in frequency tables.

VI. Subject Accountability

A. Subject Characteristics

Demography and Baseline Characteristics. The data collected on the following characteristics will be summarized for ITT, mITT and PP populations by each treatment group:

- Age (years)
- Age group: <60, ≥60 years
- Sex at birth
- Race: White, Native Hawaiian or Other Pacific Islander, Black or African American, Asian, American Indian or Alaska Native, Not Reported
- Height (cm): Height measured at Screening visit will be reported.

- Weight (kg): Weight measured at Screening visit will be reported.
- Body Mass Index (BMI): Calculated as body mass (kg) divided by the square of their height (m) with the unit of kg/m^2
- Troponin I (ng/L) and Troponin T (ng/L) local results
- Time between MI diagnosis to Troponin local results (days)

[redacted]

- LVEF (%) (<= median, > median)
- NT-proBNP (pg/mL) (<= median, > median)
- Stratification factors:
  - Age group at randomization: <60, ⩾60 years
  - Location of infarction at randomization: anterior, non-anterior
- Diabetes Status (Diabetes/non-Diabetes)
- Troponin central baseline results (<= median, > median)
- Chronic HF status (Patients with, Patients without)
- Renal function
  - eGFR (mL/min/1.72m^2) Group 1 ("<=45", ">45, <=60", ">60")
  - eGFR (mL/min/1.72m^2) Group 2 ("<=60", ">60")
- Atrial fibrillation status (Patients with, Patients without)
- Septum size (hypertrophy) (<= median, > median)

A listing of the above characteristics will be presented for the ITT, mITT and PP population.

Myocardial Infarction history. The following MI related history will be summarized for the ITT population:

- Time between MI diagnosis to Randomization (days)
- Location of MI (anterior, posterior, lateral, other)
- Type (STEMI, NSTEMI)
- Number of previous infarctions (0, 1, 2, ≥3)

A listing of the above characteristics will be presented for the ITT population.

Medical History. Medical history (MH) will be coded using Medical Dictionary for Regulatory Activities (MedDRA) version 25.0 or later. System Organ Class (SOC) and Preferred Term (PT) will be summarized by number and percentage of subjects having at least one occurrence of the event, for ITT population.

COVID-19 Infection and Vaccination. The following information related to COVID-19 will be summarized for the ITT population:

- COVID-19 (Y, N) and if Yes:
  - Hospitalization due to COVID-19 (Y, N)
  - Long COVID-19 or post COVID-19 condition (Y, N)
- COVID-19 vaccination (Y, N)

B. Disposition

A summary of subjects will display the numbers (and percentages of the population) of subjects by treatment (as applicable) for the following:

- Subjects who provided informed consent (Entered Analysis Set)
- Subjects who fail screening, overall and by reason for screen failure
- Subjects randomized
- Subjects randomized who received at least one dose of study drug
- Subjects who complete treatment
- Subjects who discontinue treatment, overall and by reason for discontinuation
- Subjects who complete study
- Subjects who discontinue study, overall and by reason for discontinuation
- Subjects included in each analysis population (ITT, mITT, Safety, PP)
  - For PP population, the number of subjects who were excluded from PP (overall and by reason for exclusion) will also be summarized.

A listing by subject will display the details of screen failure reason of each screen failed subject. A listing of treatment and study completion information, including the reason for discontinuation, if applicable, will also be presented for all subjects in the ITT population.

C. Protocol Deviations

Major protocol deviations will be summarized for the ITT population with number (and percentage) of subjects in each protocol deviation category by treatment. In addition, a by-subject listing will be presented for details of all protocol deviations.

D. Visit Attendance

Subject visit attendance will be summarized at each visit, for the following:

- Number and percentage of subjects who miss the visit

- Number and percentage of subjects who miss the visit due to incompliance

VII. Efficacy Analyses

A. Efficacy Outcomes

Primary, secondary, and exploratory efficacy endpoints are described in Section III. Additional definitions and derivations are as follows:

- Kansas City Cardiomyopathy Questionnaire (KCCQ)

  The KCCQ is a patient reported 23-item questionnaire, which includes HF symptoms, impact on physical and social function, and how their HF impacts their quality of life within a 2-week recall period.

  For the secondary efficacy outcome of change from baseline to Months 6 and 12 months in mean KCCQ score and mean scores of subdomains, the Overall Summary Score and subdomain scores (symptom burden, physical limitation, and quality of life) will be derived based on formula in Appendix.A.

[redacted]

[redacted]

B. Primary Efficacy Outcome Analysis

1. Primary Estimand

The primary estimand for establishing the efficacy of CDR132L consists of the mean difference in percent change from baseline in LVESVI at Month 6 among MI subjects randomized to either active treatment or placebo group calculated in the mITT analysis set, defined by the following attributes:

Attribute	Description
Treatments	CDR132L ([redacted] mg/kg, [redacted] mg/kg) as an add-on to SoC vs placebo as an add-on to SoC
Population	Population of subjects with HF and reduced LVEF after MI, defined through appropriate inclusion/exclusion criteria to reflect the targeted subject population for approval
Variable	Percent change from baseline in LVESVI at Month 6
Population-level summary	Mean difference in percent change from baseline in LVESVI at Month 6
Intercurrent events	Treatment discontinuation, study discontinuation, death, (hospitalization for) CRT implants and heart transplantation; the treatment policy strategy will be used for the intercurrent events.

2. Primary Efficacy Analysis

The mITT Population will be the primary analysis population. The primary efficacy endpoint will also be analyzed using the PP population as supportive analysis.

The comparison of percent (%) change from baseline LVESVI at Month 6 will be done for the [redacted] mg/kg dose group versus placebo, followed by [redacted] mg/kg versus placebo within a hierarchical two-step test procedure. The aim is to show superiority of the [redacted] mg/kg dose group (μT10) and the [redacted] mg/kg dose group (μT5) in comparison to placebo (μP). The hierarchical testing procedure is described in section V.J Multiple Comparisons.

The percent change from baseline in LVESVI will be analyzed using an analysis of covariance (ANCOVA), with placebo acting as the reference. Individual ANCOVA models will be used for each test step, i.e. the [redacted] mg/kg and [redacted] mg/kg dose groups will be compared to placebo in separate models. The model will include treatment as a fixed effect and baseline LVESVI as a covariate. The analysis will also be adjusted for center group and the stratification factors of age group (<60 years, ≥60 years) and location of infarction (anterior, non-anterior). The least squares mean (LSM) estimates for each treatment arm will be displayed together with standard errors (SEs) and their corresponding 95% CIs. Treatment differences with 95% CIs will also be produced. The LSM line plot with ± one SE from the corresponding point estimate of the LS mean will be presented.

All observed data will be included in analysis regardless of intercurrent events. Missing post-baseline data through Month 6 will be imputed using the last observation (including baseline) carried forward (LOCF) approach. In addition, multiple imputations as described above will be used for sensitivity analyses.

Supportive analyses will be performed for PP population in a similar manner to the primary efficacy analysis.

For LVESVI, absolute change and % change from baseline will be summarized by treatment group and respective timepoint. A listing will present this information for each subject.

3. Sensitivity Analysis

Sensitivity Analysis 1: Analysis using Multiple imputation

Multiple imputation based sensitivity analysis will be performed. A reference (control) based imputation model will be applied based on the missing not at random (MNAR) assumption, according to the following steps:

- All the missing values will be multiply-imputed using the imputation model built from the placebo group, i.e., assuming the missing data in the treatment groups will have a profile that equals the profile of the placebo group for all timepoints. The missing data imputation will be implemented using PROC MI in SAS 9.4 with the MNAR statement. The variables to be used in the imputation model are treatment, center group, age (<60 years, ⩾60 years), location of

infarction (anterior, non-anterior), baseline LVESVI, and LVESVI values observed at post-baseline visits.

```
Proc mi data= indata seed=&seed nimpute=100 out= outdata;
  class trt01pn stratum1 stratum2 stratum3;
  var stratum1 stratum2 stratum3 base timepoint1 timepoint2;
  fcs reg(/details);
  mnar model(timepoint1 timepoint2/ modelobs=(trt01pn='0'));
run;
```

Note: `trt01pn='0'` indicates placebo subjects.

- Once the competed datasets are formed, the same ANCOVA model as specified for the primary analysis will be applied to each completed set and inference drawn using PROC MIANALYZE.

A pre-specified seed number of 10272022 will be used in all imputation procedures as described above. Alternative model specifications may be used based on the actual data if there is an issue in model convergence.

Sensitivity Analysis 2: Analysis using Mixed model Repeated Measures (MMRM)

A MMRM model will be used to analyze the percent change from baseline using treatment group, analysis timepoint (Month 3 and Month 6), treatment group-by-month interaction, center group, age (<60 years, ≥60 years), location of infarction (anterior, non-anterior), baseline LVESVI as factors. An unstructured covariance matrix will be used to model the within-patient variance-covariance errors. If the unstructured covariance structure matrix results in a lack of convergence, the heterogenous first-order autoregressive covariance structure will be used as the first alternative, followed by the compound symmetry covariance structure and variance components covariance structure. The Kenward-Roger approximation will be used to estimate the denominator degrees of freedom. Any missing data are assumed to be missing at random.
The LSM for each treatment group with SE, LSM difference against placebo group with SE, 95% CI for the differences and the associated p-values will be presented at each analysis timepoint.

Sensitivity Analysis 3: Analysis using ANCOVA with pooled CDR132L group

Additionally, an ANCOVA analysis with the LOCF method will be performed for the pooled CDR132L group and the placebo group.

C. Secondary Efficacy Analyses

For the following continuous change from baseline (absolute/relative) secondary endpoints:

- Change in LVEF (%) at Months 3, 6, and 12
- Change in NT-proBNP (pg/mL) (on a logarithmic scale) at Days 2 to 5, Months 1, 2, 3, 6, and 12
  - The Geometric Mean will be calculated by exponentiating the mean for each treatment, and the ratio will be also calculated by exponentiating the mean of the treatment difference.
- Change in LVESVI at Month 3, 6, and 12
- Change in Troponin T (ng/L) at Months 3, 6, and 12
- Change in KCCQ score (Overall Summary score) and subdomain scores (Physical Limitation, Symptom Burden, Quality of Life) at Months 6 and 12

A similar ANCOVA will be used, with placebo acting as the reference. The model will include treatment as a fixed effect and baseline value as a covariate. Also, the analysis will be adjusted for center group and the stratification factors of age group (<60 years, ⩾60 years) and location of infarction (anterior, non-anterior).

Similar to the primary endpoint analysis, LOCF approach will be used for subjects that have missing data. No imputation will be performed for visits after Month 6. The PP population will also be used for LVEF (%) and NT-proBNP (pg/mL) secondary endpoints analysis.

Trellis plots and spider plots will be provided for NT-proBNP (pg/mL).

C.3. Testing strategy for the confirmatory secondary efficacy endpoints

Similar to the primary endpoint analysis, ANCOVA model with LOCF method will be used for absolute change from baseline in confirmatory secondary endpoints below at Month 6. The testing procedure is described in section V.J. Multiple Comparisons.

- Absolute change from baseline in LVEF (%) at Month 6
- Absolute change from baseline in NT-proBNP (pg/mL) (on a logarithmic scale) at Month 6

[redacted]

- Well-being as evaluated by absolute change from baseline at Month 6 in the following parameters:
  - Mean Kansas City Cardiomyopathy Questionnaire (KCCQ) score (i.e. Overall Summary score)

C.4. Sensitivity Analysis

Sensitivity Analysis 1: Analysis using Multiple Imputation

Similar to the primary endpoint analysis, MI method will be used for change from baseline at Month 6 with the endpoints below.
For the LVEF (%) endpoint, the variables to be used in the imputation model are treatment, center group, age (<60 years, ≥60 years), location of infarction (anterior, non-anterior), baseline LVEF (%), and LVEF (%) values observed at post-baseline visits.
For the NT-proBNP (pg/mL) endpoint, the variables to be used in the imputation model are treatment, center group, age (<60 years, ≥60 years), location of infarction (anterior, non-anterior), baseline NT-proBNP (pg/mL), and NT-proBNP (pg/mL) values observed at post-baseline visits.

Sensitivity Analysis 2: Analysis using Mixed model Repeated Measures (MMRM)

Similar to the primary endpoint analysis, MMRM model will be used for change from baseline with the endpoints below.
The treatment group, analysis timepoint (Month 3 and Month 6), treatment group-by-month interaction, center group, age (<60 years, ≥60 years), location of infarction (anterior, non-anterior), baseline LVEF (%) will be included in the MMRM model for LVEF (%) analysis.
The treatment group, analysis timepoint (Month 1, Month 2, Month 3 and Month 6), treatment group-by-month interaction, center group, age (<60 years, ≥60 years), location of infarction (anterior, non-anterior), baseline NT-proBNP (pg/mL) will be included in the MMRM model for NT-proBNP (pg/mL) analysis.

D. Exploratory Efficacy Analysis

[redacted]

[redacted]

[redacted]

[redacted]

E. Subgroup analyses

Subgroup analyses based on age group, sex, MI type, time of first treatment after MI event, patients with and without treatment of SGLT2 inhibitors and disease severity as defined in section V.L may also be performed in an exploratory manner for secondary [LVESVI, LVEF (%) and NT-proBNP (pg/mL)] endpoints.

VIII. Safety Analyses

All safety analyses will be based on the Safety Population. No formal statistical analysis of the safety data will be performed.

A. Exposure

Exposure to the treatment will be summarized by group, for each dosing visit and overall, in terms of the following:

- number of doses administered
- administered dose (mL)
- % planned dose administered
    - (administered dose/ planned dose (24 mL))*100

Cases where reduced dose has been infused, as well as cases of infusion interruption, will be summarized along with the reason that has been reported (AE, other). This information will also be presented as a by-subject listing.

B. Adverse Events

Adverse events will be coded using the Medical Dictionary for Regulatory Activities (MedDRA) version 25.0 or higher.

Treatment-emergent adverse events (TEAEs) are defined as AEs that first occurred or worsened in severity after the first administration of study treatment and prior to 30 days after the last administration of study treatment.

AE is considered treatment-related if the relationship to the study drug is “related”; if relationship to study treatment is missing, the event will be summarized as a separate “missing” category. Missing severity will be summarized as a separate “missing” category.

The following AE information will be summarized by treatment group:

- All TEAEs
- Treatment related TEAEs
- SAEs
- Serious TEAEs
- Serious Treatment-related TEAEs
- TEAEs leading to discontinuation of study treatment
- TEAEs leading to death
- Adverse events of special interest (AESIs)
- Injection site reactions

The following AE summaries will be produced by SOC (alphabetical order) and PT (descending order of the overall frequency):

- TEAEs
- TEAEs by maximum severity (mild, moderate, severe). TEAEs missing a severity grade will be reported in a separate “missing” category.
- Treatment Related TEAEs
- SAEs
- TEAEs leading to discontinuation of study treatment
- TEAEs leading to deaths

AEs will be summarized with the number of patients, percentage of patients, number of events, and the rate of events.
The rate of events is calculated as (number of events / patient years of exposure), where patient years of exposure = (last dose date – first dose date + 31) / 365.25.

At each level of summarization, a patient will be counted once if he/she reported one or more events. The severity grade and relationship to study drug will be summarized in a similar manner.

All AEs will be presented in a by-subject listing, detailing in the verbatim term given by the investigator, PT, SOC, start date/time, end date/time or ongoing, seriousness, intensity grade, outcome, relationship to study drug, action taken with study drug, and other action taken.

C. Clinical Laboratory Results

Quantitative laboratory test variables (including hematology, coagulation, serum chemistry, and urinalysis) will be summarized by treatment group and visit (as per SOA in Protocol Section 1.3) using descriptive statistics; number and percentage of subjects with normality status (low, normal and high) will also be summarized. Laboratory tests with categorical results (e.g., urinalysis) will be summarized by treatment group and visit by the number of subjects in each category.

Shift tables (low, normal, high according to the normal range criteria) between baseline and post-baseline time points will be presented by laboratory test and treatment group.

Laboratory tests with categorical results that cannot be analyzed by change from baseline or shift table analysis will not be included in these summaries but will be listed. Data obtained from laboratory tests not required by the protocol will not be summarized but will be listed.

Trellis plots (line plots over time) and scatter plots (Baseline v.s Max post-baseline) will be provided for chemistry, hematology, and coagulation. Additionally, spider plots will be provided for kidney tests, liver tests, and thrombocytes.

D. Vital Signs

Descriptive statistics of vital signs will be summarized for each treatment group using descriptive statistics at each visit for absolute values and change from baseline values (as per SOA in Protocol Section 1.3). Additionally, spider plots will be provided.

The following variables will be included:

- Body temperature (°C)
- Systolic blood pressure (mmHg)
- Diastolic blood pressure (mmHg)
- Respiratory rate (beats/min)
- Pulse rate (beats/min)

- Normality status (normal, abnormal with clinical significance, or abnormal without clinical significance)

All vital sign variables will be presented in a by-subject listing for all recorder visits.

E. 12-Lead Electrocardiogram (ECG)

Descriptive statistics of ECG will be summarized for each treatment group using descriptive statistics at each visit for absolute values and change from baseline values (as per SOA in Protocol Section 1.3). Additionally, spider plots will be provided.

The following variables will be included:
- Heart rate (beats/min)
- PR (msec)
- QRS (msec)
- QT uncorrected (msec)
- QTcF interval (msec)

F. Physical Examination

Clinically important abnormal physical examination results will be recorded as MH or AEs and analyzed accordingly. Subjects with abnormal physical examination will be presented in a by-subject listing.

G. Concomitant Medications

For analysis purposes, the start and stop dates of the medications will be used to determine the prior and concomitant medications based on the definition below. Prior medications are defined as medications with start and stop times prior to the time of study drug administration. Concomitant medications are defined as medications with a start time at or after the time of study drug administration or medications with a start time prior to study drug administration but continuing after treatment.

Prior and Concomitant Medications will be coded using the WHO Drug version B3 Global March 2022 or later. The number and percentage of subjects with prior or concomitant medications will be summarized by WHO-DD Anatomical-Therapeutic-Chemical (ATC) classification level 4 or highest level available, and preferred term (PT) for each treatment.

Concomitant Medication(s) information will be presented in a by-subject listing for all recorder visits.

H. Immunogenicity Analysis

Immunogenicity evaluation will be performed by a specialty laboratory after completion of the study. This analysis and results will be covered in a separate report.

IX. References

Rubin DB. Multiple imputation for nonresponse in surveys. John Wiley & Sons; 9 June 2004.

E9(R1) Statistical Principles for Clinical Trials: Addendum: Estimands And Sensitivity Analysis in Clinical Trials Guidance for Industry

Multiple Endpoints in Clinical Trials Guidance for Industry. October 2022.

X. Appendix

A. Kansas City Cardiomyopathy Questionnaire (KCCQ)

The 23-item KCCQ questionnaire contains 7 domains which include the following:
Physical Limitation (6 items)
Symptom Stability (1 item)
Symptom Frequency (4 items)
Symptom Burden (3 items)
Self-Efficacy (2 items)
Quality of Life (3 items)
Social Limitations (4 items)

All items are measured on a Likert scale with 5–7 response options. In addition, three summary scores are calculated:

Total Symptom score (average of Symptom Frequency and Symptom Burden)
Clinical Summary score (average of Physical Limitation and Total Symptoms)
Overall Summary score (average of Physical Limitation, Total Symptoms, Quality of Life, and Social Limitation)

The individual item responses and domains scores will be calculated per the table below:

Scale	Item Number	Original response
Physical Limitation	1a to 1f	Extremely limited = 1<br>Quite a bit limited = 2<br>Moderately limited = 3<br>Slightly limited = 4<br>Not at all limited = 5<br>Limited for other reasons or did not do = missing
	Step 1:<br>The original response needs to be transferred to the score value using the formula below:<br>S = [(Questions) - 1]/4	
	Step 2:<br>If at least three of scores are not missing:<br>Physical Limitation Score = 100*(mean of available S results)	
Symptom Stability	2	Much worse = 1<br>Slightly worse = 2<br>Not changed = 3<br>Slightly better = 4<br>Much better = 5<br>I've had no symptoms over the last 2 weeks = 3
	Step 1:	

	The original response needs to be transferred to the score value using the formula below:<br>S = [(Questions) - 1]/4	
	Step 2:<br>If the score is not missing:<br>Symptom Stability Score = 100*(mean of available S result)	
Symptom Frequency	3,5,7,9	<u>Question 3</u><br>Every morning = 1<br>3 or more times a week but not every day = 2<br>1-2 times a week = 3<br>Less than once a week = 4<br>Never over the past 2 weeks = 5<br><u>Questions 5 and 7</u><br>All of the time = 1<br>Several times a day = 2<br>At least once a day = 3<br>3 or more times a week but not every day = 4<br>1-2 times a week = 5<br>Less than once a week = 6<br>Never over the past 2 weeks = 7<br><u>Question 9</u><br>Every night = 1<br>3 or more times a week but not every day = 2<br>1-2 times a week = 3<br>Less than once a week = 4<br>Never over the past 2 weeks = 5
	<u>Step 1:</u><br>S3 = [(Question 3) – 1]/4<br>S5 = [(Question 5) – 1]/6<br>S7 = [(Question 7) – 1]/6<br>S9 = [(Question 9) – 1]/4	
	Step 2:<br>If at least two of scores are not missing:<br>Symptom Frequency Score = 100*(mean of S3, S5, S7 and S9)	
Symptom Burden	4,6,8	Extremely bothersome = 1<br>Quite a bit bothersome = 2<br>Moderately bothersome = 3<br>Slightly bothersome = 4<br>Not at all bothersome = 5<br>I've had no swelling/fatigue/shortness of breath = 5
	Step 1:<br>The original response needs to be transferred to the score value using the formula below:<br>S = [(Questions) - 1]/4	
	Step 2:<br>If at least one of scores is not missing:<br>Symptom Burden Score = 100*(mean of S4, S6 and S8)	
Self-Efficacy	10, 11	<u>Question 10</u>

		Not at all sure = 1<br>Not very sure = 2<br>Somewhat sure = 3<br>Mostly sure = 4<br>Completely sure = 5<br><u>Question 11</u><br>Do not understand at all = 1<br>Do not understand very well = 2<br>Somewhat understand = 3<br>Mostly understand = 4<br>Completely understand = 5
	Step 1:<br>The original response needs to be transferred to the score value using the formula below:<br>S = [(Questions) - 1]/4	
	Step 2:<br>If at least one of score is not missing:<br>Self-Efficacy Score = 100*(mean of S10 and S11)	
Quality of Life	12, 13, 14	<u>Question 12</u><br>It has extremely limited my enjoyment of life = 1<br>It has limited my enjoyment of life quite a bit = 2<br>It has moderately limited my enjoyment of life = 3<br>It has slightly limited my enjoyment of life = 4<br>It has not limited my enjoyment of life at all = 5<br><u>Question 13</u><br>Not at all satisfied = 1<br>Mostly dissatisfied = 2<br>Somewhat satisfied = 3<br>Mostly satisfied = 4<br>Completely satisfied = 5<br><u>Question 14</u><br>I felt that way all of the time = 1<br>I felt that way most of the time = 2<br>I occasionally felt that way = 3<br>I rarely felt that way = 4<br>I never felt that way = 5
	Step 1:<br>The original response needs to be transferred to the score value using the formula below:<br>S = [(Questions) - 1]/4	
	Step 2:<br>If at least one of scores is not missing:<br>Quality of Life Score = 100*(mean of S12, S13 and S14)	
Social Limitations	15a to 15d	Severely limited = 1<br>Limited quite a bit = 2<br>Moderately limited = 3<br>Slightly limited = 4<br>Did not limit at all = 5

		Does not apply or did not do for other reasons = Missing
	Step 1:<br>The original response needs to be transferred to the score value using the formula below:<br>S = [(Questions) - 1]/4	
	Step 2:<br>If at least two of scores are not missing:<br>Social Limitations Score = 100*(mean of S15a to 15d)	

DocuSign

Certificate Of Completion

Envelope Id: 5EE2D1AC325247D5B25E456DBC938FF2 — Status: Completed

Subject: Complete with Docusign: Cardior_Statistical_Analysis_Plan_v2.0_20241011_clean.docx

Project Code (Enter 0 for non-billable projects): QZA91424

[redacted] (Login ID): [redacted]

Business Unit:

[redacted]

Source Envelope:

Document Pages: 43	Signatures: 3	Envelope Originator:
Certificate Pages: 5	Initials: 0	[redacted]
AutoNav: Enabled		
EnvelopeId Stamping: Disabled		
Time Zone: (UTC-05:00) Eastern Time (US & Canada)		IP Address: [redacted]

Record Tracking

Status: Original<br>10/11/2024 5:25:43 AM	Holder: [redacted]	Location: DocuSign

Signer Events	Signature	Timestamp
[redacted]<br>Security Level: Email, Account Authentication (Required)	[redacted]<br><br>Signature Adoption: Pre-selected Style<br>Signature ID:<br>AF08918C-D2FC-4F6E-90CC-CCCC535143DE<br>Using IP Address: [redacted]<br><br>With Signing Authentication via DocuSign password<br>With Signing Reasons (on each tab):<br>I approve this document	Sent: 10/11/2024 5:27:52 AM<br>Viewed: 10/11/2024 5:28:24 AM<br>Signed: 10/11/2024 5:29:03 AM
**Electronic Record and Signature Disclosure:**<br>Not Offered via DocuSign		
[redacted]<br>Security Level: Email, Account Authentication (Required)	[redacted]<br><br>Signature Adoption: Pre-selected Style<br>Signature ID:<br>F709870B-76EA-4403-ACE2-59DFE529BFFD<br>Using IP Address: [redacted]<br>Signed using mobile<br>With Signing Authentication via DocuSign password<br>With Signing Reasons (on each tab):<br>Ich genehmige dieses Dokument	Sent: 10/11/2024 5:27:53 AM<br>Viewed: 10/11/2024 6:55:18 AM<br>Signed: 10/11/2024 6:55:52 AM
**Electronic Record and Signature Disclosure:**<br>Accepted: 10/11/2024 6:55:18 AM<br>ID: 369a9de3-1371-4865-a521-401fe4312da0<br>Company Name: [redacted]		

Signer Events	Signature	Timestamp
[redacted]<br>Security Level: Email, Account Authentication (Required)	Signiert von [redacted]<br>[redacted] Ich genehmige dieses Dokument<br>11-Oct-2024 | 02:29:09 PDT<br>5AA80916C7854806B52E9F545E4CCB02<br><br>Signature Adoption: Pre-selected Style<br>Signature ID:<br>5AA80916-C785-4806-B52E-9F545E4CCB02<br>Using IP Address: [redacted]<br><br>With Signing Authentication via DocuSign password<br>With Signing Reasons (on each tab):<br>Ich genehmige dieses Dokument	Sent: 10/11/2024 5:27:53 AM<br>Viewed: 10/11/2024 5:28:52 AM<br>Signed: 10/11/2024 5:29:12 AM

Electronic Record and Signature Disclosure:
Accepted: 10/11/2024 5:28:52 AM
ID: 5286651c-45c5-4209-affc-1a49286fed2e
Company Name: [redacted]

In Person Signer Events	Signature	Timestamp

Editor Delivery Events	Status	Timestamp

Agent Delivery Events	Status	Timestamp

Intermediary Delivery Events	Status	Timestamp

Certified Delivery Events	Status	Timestamp

Carbon Copy Events	Status	Timestamp

Witness Events	Signature	Timestamp

Notary Events	Signature	Timestamp

Envelope Summary Events	Status	Timestamps
Envelope Sent	Hashed/Encrypted	10/11/2024 5:27:54 AM
Certified Delivered	Security Checked	10/11/2024 5:28:52 AM
Signing Complete	Security Checked	10/11/2024 5:29:12 AM
Completed	Security Checked	10/11/2024 6:55:52 AM

Payment Events	Status	Timestamps

Electronic Record and Signature Disclosure

CONSENT TO ELECTRONIC DELIVERY AND EXECUTION OF DOCUMENTS

From time to time, [redacted] ("we" or "us") may provide you certain written contracts, notices, disclosures, authorizations, acknowledgements or other documents (collectively, the "Documents") electronically. Please read this consent form carefully. It explains the terms and conditions under which such Documents are provided by us and executed by you electronically through your DocuSign, Inc. ("DocuSign") user account. If you consent to the delivery and execution of such Documents electronically, please click the "I Agree" button.

Documents will be sent to you electronically

If you consent to electronic delivery, Documents will be sent to your DocuSign user account. You may request a paper copy of documents previously made available through your DocuSign user account, but an additional charge may be incurred. Alternatively, you can download and print documents sent to your DocuSign user account. Unless otherwise noted, you can access a Document up to 30 days from the date we first sent the Document to you.

Withhold Consent or Withdrawing Consent to Electronic Delivery

If you withhold consent to electronic delivery or execution, or withdraw your consent at a later date, all Documents will be sent to your mailing address following our receipt of notice of such action. The following sections explain the consequences of withholding or withdrawing your consent to electronic delivery and execution of Documents, and also the procedures you must follow in order to effectuate delivery to your mailing address.

Consequences of Withdrawing Consent

By electing to only receive and execute Documents sent to your mailing address, we will not be able to carry out transactions or services as efficiently. For instance, some transactions or services require your express consent. We can perform these transaction or services only if we first receive an acknowledgement that indicates you received and consent to the Document related to the proposed transaction or service.

To withhold consent now or withdraw consent at a later date, please sign DocuSign's "Withdraw Consent" form on the signing page of your DocuSign user account. This will indicate that you have withdrawn your consent to receive Documents electronically. Once you sign the "Withdraw Consent" form, you will no longer be able to use your DocuSign user account to execute Documents electronically and we will send Documents to your mailing address. Withdrawal of consent does not affect the validity of any Documents previously executed electronically prior to such withdrawal of Consent. In addition, should you execute any Documents electronically, your execution of such Documents shall indicate your continued consent to execute such Documents electronically.

How to contact [redacted]:

If you would like us to send the Documents to a different e-mail address, request paper copies of Documents you have previously received electronically, or withdraw your consent to receive electronic documents, please follow the instructions below. If you have any other questions, please contact: [redacted]

1. To advise [redacted] of your new e-mail address

If you would like your Documents sent to a different e-mail address, you must send an e-mail message to [redacted]. In the body of the e-mail please state the following: (i) your previous e-mail address, and (ii) your new e-mail address. No other information is required.

In addition, you must notify DocuSign of your new e-mail address. Please log into your DocuSign user account, and follow the instructions to update your e-mail address.

2. To request paper copies from [redacted]

To request paper copies of Documents you have received previously through your DocuSign user account, send an e-mail to [redacted]

In the body of the e-mail please state the following: (i) your e-mail address, (ii) full name, (iii) U.S. Postal address, and (iv) telephone number. Additional charges may apply for such paper copies.

3. To withdraw your consent with [redacted]

To withdraw your consent to receiving and executing Documents in an electronic format, you may do one of the following:

> i. decline to sign a document from within your DocuSign user account, and on the subsequent page, select the check-box indicating you wish to withdraw your consent; or
> ii. send us an e-mail to [redacted] and in the body of such request you must state your e-mail, full name, US Postal Address, telephone number, and account number. No additional information is necessary.

Required hardware and software

Operating Systems:	Windows® 2000, Windows® XP, Windows Vista®; Mac OS® X
Browsers:	• Internet Explorer (Windows Only) 8.0 or above – compatibility mode is supported only for 9.0 and above.<br>• Windows Edge Current Version<br>• Mozilla Firefox Current Version<br>• Safari (Mac OS only) 6.2 or above<br>• Google Chrome Current Version
PDF Reader:	Acrobat® or similar software may be required to view and print PDF files
Screen Resolution:	1024 x 768 Recommended
Enabled Security Settings:	Allow per session cookies
Mobile Signing:	• Apple iOS 7.0 or above<br>• Android 4.0 or above

** These minimum requirements are subject to change. If these requirements change, we will provide you with an e-mail message at the e-mail address we have on file for you at the time the hardware and software requirements are revised.

Pre-release (e.g. beta) versions of operating systems and browsers are not supported.

Acknowledging your access and consent to receive materials electronically

To confirm you can access this information electronically and that you consent to receiving and executing Documents electronically on the terms and conditions described above, please let us know by clicking the "I Agree" button.

By clicking the "I Agree" button, you confirm that

- You can access and read this Consent To Electronic Delivery and Execution of Documents; and
- You can print on paper the disclosure or save or send the disclosure to a place where you can print it, for future reference and access; and
- Until or unless you notify [redacted] as described above, you consent to the delivery and execution of Documents electronically.
