# Supplementary material for: The microRNA inhibitor CDR132L in patients with reduced left ventricular ejection fraction after myocardial infarction: a randomized phase 2 trial
Source: Nat Med. 2026 May 10;32(7):2654–61. doi: 10.1038/s41591-026-04408-4 (PMC13375544; doi:10.1038/s41591-026-04408-4)
Supplement: Supplementary file 2 — Reporting Summary [file 41591_2026_4408_MOESM2_ESM.pdf]

nature portfolio

Corresponding author(s): Thomas Thum

Last updated by author(s): Apr 14, 2026

Reporting Summary

Nature Portfolio wishes to improve the reproducibility of the work that we publish. This form provides structure for consistency and transparency in reporting. For further information on Nature Portfolio policies, see our Editorial Policies and the Editorial Policy Checklist.

Statistics

For all statistical analyses, confirm that the following items are present in the figure legend, table legend, main text, or Methods section.

n/a	Confirmed	
☐	☒	The exact sample size (*n*) for each experimental group/condition, given as a discrete number and unit of measurement
☐	☒	A statement on whether measurements were taken from distinct samples or whether the same sample was measured repeatedly
☐	☒	The statistical test(s) used AND whether they are one- or two-sided<br>*Only common tests should be described solely by name; describe more complex techniques in the Methods section.*
☐	☒	A description of all covariates tested
☐	☒	A description of any assumptions or corrections, such as tests of normality and adjustment for multiple comparisons
☐	☒	A full description of the statistical parameters including central tendency (e.g. means) or other basic estimates (e.g. regression coefficient) AND variation (e.g. standard deviation) or associated estimates of uncertainty (e.g. confidence intervals)
☐	☒	For null hypothesis testing, the test statistic (e.g. *F*, *t*, *r*) with confidence intervals, effect sizes, degrees of freedom and *P* value noted<br>*Give P values as exact values whenever suitable.*
☒	☐	For Bayesian analysis, information on the choice of priors and Markov chain Monte Carlo settings
☐	☒	For hierarchical and complex designs, identification of the appropriate level for tests and full reporting of outcomes
☒	☐	Estimates of effect sizes (e.g. Cohen's *d*, Pearson's *r*), indicating how they were calculated

Our web collection on statistics for biologists contains articles on many of the points above.

Software and code

Policy information about availability of computer code

Data collection	EDC Tool: IBM Clinical Development. Initial software version 2022.2.0.1. EDC Designer: IQVIA Biotech
Data analysis	SAS version 9.4

For manuscripts utilizing custom algorithms or software that are central to the research but not yet described in published literature, software must be made available to editors and reviewers. We strongly encourage code deposition in a community repository (e.g. GitHub). See the Nature Portfolio guidelines for submitting code & software for further information.

Data

Policy information about availability of data

All manuscripts must include a data availability statement. This statement should provide the following information, where applicable:

- Accession codes, unique identifiers, or web links for publicly available datasets
- A description of any restrictions on data availability
- For clinical datasets or third party data, please ensure that the statement adheres to our policy

Researchers can request access to clinical trial data by submitting a research proposal for review and approval by Novo Nordisk and an internal independent review panel. Requests are considered after the research is concluded or completed and the main results have been published. If the research supports a regulatory application, requests will be considered after the product and its intended use are approved in both the EU and the USA. Participants' clinical data will be

anonymized, following an approved internal process, before data are shared to external third parties. For details on how to request access to clinical data, visit novonordisk-trials.com.

Research involving human participants, their data, or biological material

Policy information about studies with human participants or human data. See also policy information about sex, gender (identity/presentation), and sexual orientation and race, ethnicity and racism.

Reporting on sex and gender	13% of participants were women, based on sex at birth. Data on gender were not collected. Some further exploratory analyses were performed stratified by sex for key endpoints, including LVESVI and LVEF, but the small number of treated women does not allow a meaningful conclusion.
Reporting on race, ethnicity, or other socially relevant groupings	96% of participants were White, 1% were Black or African American, 1% were Asian, 1% were Native Hawaiian or Other Pacific Islander. Race was not reported for 2% of participants.
Population characteristics	In the mITT population, the median age was 61.0 years (range: 35–80), 13% were women, and 81% presented with ST-segment elevation MI (STEMI). The median time from MI to randomization was 11.0 days (range: 4–15). Median LVEF at baseline was 36% (range 13.1–54.9) and median NT-proBNP concentration was 1307 pg ml^{-1} (range: 55.0-17451.0). Background therapy was consistent with contemporary post-MI guideline-directed care, with >90% of patients receiving beta-blockers, angiotensin-converting enzyme inhibitors, angiotensin receptor blockers or angiotensin receptor–neprilysin inhibitors, as well as 84% of patients receiving mineralocorticoid receptor antagonists. A high number of patients (79%) additionally received sodium-glucose co-transporter 2 (SGLT2) inhibitors on top of current standard of care.
Recruitment	Between July 2022 and March 2024, a total of 427 patients with acute MI were screened across 54 international sites, of whom 294 met eligibility criteria and were randomized to CDR132L 5 mg kg^{-1} (n = 98), CDR132L 10 mg kg^{-1} (n = 98) or placebo (n = 98).
Ethics oversight	The study protocol, all study protocol amendments, written study participant information, informed consent form (ICF), Investigator's Brochure and any other relevant documents were reviewed and approved by an independent ethics committee (IEC) or institutional review board (IRB) at each study site.<br>This study was conducted in accordance with the study protocol and all approved amendments; the International Council for Harmonisation (ICH) Guideline for Good Clinical Practice E6(R2); the ethical principles of the Declaration of Helsinki; the Council for International Organizations of Medical Sciences, International Ethical Guidelines; all applicable local laws and regulations; and ICH requirements for archiving and retention of essential documents.

Note that full information on the approval of the study protocol must also be provided in the manuscript.

Field-specific reporting

Please select the one below that is the best fit for your research. If you are not sure, read the appropriate sections before making your selection.

☒ Life sciences ☐ Behavioural & social sciences ☐ Ecological, evolutionary & environmental sciences

For a reference copy of the document with all sections, see nature.com/documents/nr-reporting-summary-flat.pdf

Life sciences study design

All studies must disclose on these points even when the disclosure is negative.

Sample size	Sample size was determined using a one-sided two-sample t-test with a significance level of 2.5%. Based on assumed mean LVESVI changes of 1% for placebo, 5% for CDR132L 5 mg kg^{-1} and 6% for CDR132L 10 mg kg^{-1}, each with a common SD of 9%, a sample size of 90 patients per group provided 96.0% power to detect a 5 percentage-point difference between 10 mg kg^{-1} and placebo (step 1), and 84.3% power to detect a 4 percentage-point difference between 5 mg kg^{-1} and placebo (step 2), within a hierarchical testing procedure. This resulted in an overall power of 80.9%. To account for early dropouts, the planned sample size was increased to 294 patients.
Data exclusions	Of 427 patients screened, 294 were randomized (ITT population). The mITT population (N = 280) comprised all randomized patients who received at least one dose of study drug. Fourteen randomized patients did not receive study treatment and were therefore excluded from the mITT population. The PP population (N = 250) included patients from the ITT population who completed treatments and the 6-month visit without any major protocol deviations that could affect efficacy assessments. Exclusions were primarily due to treatment discontinuation, missing data, or post-randomization events including cardiac resynchronization therapy [CRT] implantation, heart transplantation, or death. A CONSORT diagram is provided in the manuscript.
Replication	Not applicable in the context of a clinical trial
Randomization	Patients were randomized centrally using a computer-generated schedule stratified by site.
Blinding	Investigators, patients, core lab personnel and the sponsor remained blinded throughout the study. The study drug was administered in a double-blind fashion whereby patients and clinical study site staff were blinded to the study treatment. The pharmacy staff who prepared the study drug were not blinded to study drug assignment.

Reporting for specific materials, systems and methods

We require information from authors about some types of materials, experimental systems and methods used in many studies. Here, indicate whether each material, system or method listed is relevant to your study. If you are not sure if a list item applies to your research, read the appropriate section before selecting a response.

Materials & experimental systems

n/a	Involved in the study
☒	☐ Antibodies
☒	☐ Eukaryotic cell lines
☒	☐ Palaeontology and archaeology
☒	☐ Animals and other organisms
☐	☒ Clinical data
☒	☐ Dual use research of concern
☒	☐ Plants

Methods

n/a	Involved in the study
☒	☐ ChIP-seq
☒	☐ Flow cytometry
☒	☐ MRI-based neuroimaging

Clinical data

Policy information about clinical studies

All manuscripts should comply with the ICMJE guidelines for publication of clinical research and a completed CONSORT checklist must be included with all submissions.

Clinical trial registration: NCT05350969

Study protocol: Included in Supplementary Information

Data collection: Transthoracic echocardiograms were obtained at baseline and at months 3, 6 and 12 using a standardized imaging protocol. All study sonographers at study site were sufficiently trained and had to be certified before study start. All echocardiograms obtained at study site were transferred to the Brigham/Harvard core laboratory for blinded analysis.

Plasma miR-132 levels were quantified at Biotype GmbH (Dresden, Germany) using the CardiorHealth miR-132 Plasma PCR Kit (Cardior Pharmaceuticals GmbH, Hannover, Germany) in accordance with the manufacturer's instructions.

Outcomes: The primary endpoint was the percent change in LVESVI from baseline to month 6. The primary analysis used an analysis of covariance (ANCOVA) model that included treatment as a fixed effect and baseline LVESVI as a covariate.

Secondary endpoints (changes from baseline to month 6 and 12 in LVEF, NT-proBNP, troponin T, GLS, KCCQ, and LVESVI to month 12) were analyzed using ANCOVA, with change scores expressed as LSMs with standard errors and 95% CIs. No adjustment for multiple comparisons was prespecified.

Predefined subgroup analyses of the primary endpoints were performed at month 6 and month 12 in the mITT and PP populations. Subgroup analyses of the secondary endpoints (i.e. absolute and relative change from baseline in LVESVI, LVEF and NT-proBNP) were also performed at months 6 and 12 in the mITT and PP populations.

Plants

Seed stocks: *Report on the source of all seed stocks or other plant material used. If applicable, state the seed stock centre and catalogue number. If plant specimens were collected from the field, describe the collection location, date and sampling procedures.*

Novel plant genotypes: *Describe the methods by which all novel plant genotypes were produced. This includes those generated by transgenic approaches, gene editing, chemical/radiation-based mutagenesis and hybridization. For transgenic lines, describe the transformation method, the number of independent lines analyzed and the generation upon which experiments were performed. For gene-edited lines, describe the editor used, the endogenous sequence targeted for editing, the targeting guide RNA sequence (if applicable) and how the editor was applied.*

Authentication: *Describe any authentication procedures for each seed stock used or novel genotype generated. Describe any experiments used to assess the effect of a mutation and, where applicable, how potential secondary effects (e.g. second site T-DNA insertions, mosiacism, off-target gene editing) were examined.*
